# MIL-100(Fe)-Enabled Oral Delivery of Syringic Acid with Enhanced Pharmacokinetics

**DOI:** 10.3390/pharmaceutics17101282

**Published:** 2025-10-01

**Authors:** Joshua H. Santos, Hannah Jean Victoriano, Mary Sepulveda, Hung-En Liu, Shierrie Mae N. Valencia, Rikkamae Zinca Marie L. Walde, Emelda A. Ongo, Chia-Her Lin

**Affiliations:** 1 Department of Science and Technology—Central Office, General Santos Avenue, Upper Bicutan, Taguig City 1631, Philippines; eaongo@itdi.dost.gov.ph; 2 Department of Science and Technology—Industrial Technology Development Institute, General Santos Avenue, Upper Bicutan, Taguig City 1631, Philippines; victorianohannahjean@gmail.com (H.J.V.); msmarysepulveda@gmail.com (M.S.); smnvalencia@itdi.dost.gov.ph (S.M.N.V.); rzmlwalde@itdi.dost.gov.ph (R.Z.M.L.W.); 3 Department of Chemistry, National Taiwan Normal University, No. 162, Section 1, Heping E Rd, Da’an District, Taipei City 10610, Taiwan; ikea940155@gmail.com; 4 Department of Chemistry, National Tsing Hua University, 101, Section 2, Kuang-Fu Road, Hsinchu 300044, Taiwan; chiaher@mx.nthu.edu.tw

**Keywords:** syringic acid, bioavailability, MIL-100(Fe), AUC, MOF

## Abstract

**Background/Objectives:** Plant-derived bioactive compounds like syringic acid, a phenolic acid from the shikimic acid pathway, have shown potential against chronic diseases, including diabetes, cardiovascular disorders, cancer, and cerebral ischemia. However, its poor water solubility and rapid systemic elimination result in low oral bioavailability, limiting therapeutic potential. This study aimed to enhance its oral bioavailability using MIL-100(Fe), a metal–organic framework (MOF) known for high surface area and drug-loading capacity. **Methods:** MIL-100(Fe) was synthesized using an optimized method and loaded with syringic acid through impregnation at 12, 24, 36, and 48 h. Characterization included PXRD, FTIR, BET, SEM, and DLS. Acute oral toxicity was evaluated following OECD 423 guidelines, and bioavailability was assessed in Sprague Dawley rats. **Results:** The 1:2 MIL-100(Fe) to syringic acid ratio achieved the highest drug loading at 64.42 ± 0.03% (12 h). PXRD and FTIR confirmed successful loading (notably at 1239.2 cm^−1^), and TGA indicated thermal stability at ~350 °C. SEM revealed octahedral particles with an average size of 270.67 ± 2.60 nm. BET showed reduced surface area post-loading. In vitro drug release exhibited media-dependent profiles. Toxicity tests indicated no adverse effects at 2000 mg/kg. Oral administration of SYA@MIL-100(Fe) resulted in a 10.997-fold increase in relative bioavailability versus oral syringic acid and a 12.82-fold increase compared to intraperitoneal administration. **Conclusions:** MIL-100(Fe) is a safe and effective oral carrier for syringic acid, significantly enhancing its bioavailability. This platform shows strong potential for delivering phenolic compounds in pharmaceutical applications.

## 1. Introduction

**Figure 1:**
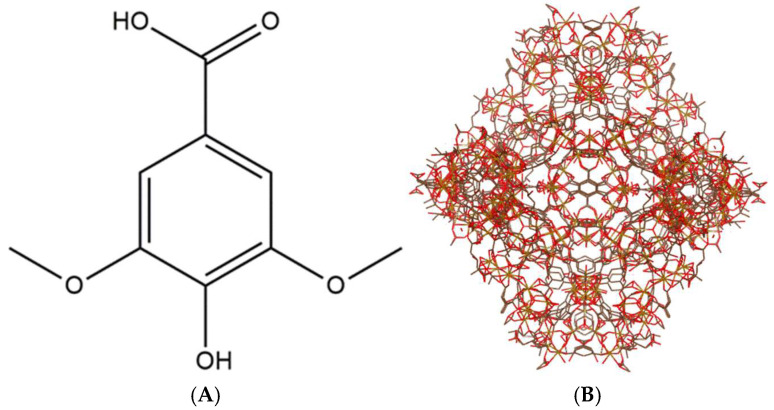
Structure of the (**A**) phenolic acid, syringic acid, and 3,5-Dimethoxy-4-hydroxybenzoic acid, as well as (**B**) MIL-100(Fe) (as visualized using VESTA, version 2025, Japan).

Natural compounds have long served as valuable lead structures in drug discovery, with approximately 60% of commercial pharmaceuticals derived from natural sources. Recent advancements in drug development have renewed interest in natural products for the treatment of various diseases, including multidrug-resistant infections [1]. Despite their broad pharmacological potential, many natural compounds suffer from low bioavailability due to unfavorable physicochemical properties and poor gastrointestinal disposition, with factors such as poor solubility, chemical and plasma instability, transformation by gut microbiota, limited permeability, and extensive metabolism contributing to reduced systemic availability [2]. Among natural products, plant-derived phenolic acids exhibit notable antioxidant properties linked to protective effects against oxidative stress-related conditions, including cancer, diabetes, liver disorders, and cardiovascular diseases [3,4], neuroprotective effects against neurodegenerative diseases like Alzheimer’s disease [5], and antimicrobial potential [6]. These secondary metabolites, biosynthesized from L-phenylalanine or L-tyrosine, typically feature one or more hydroxyl groups attached to an aromatic ring [7,8], with phenolic or phenol-carboxylic acids characterized by at least one carboxylic acid group and commonly found in plants as esters, amides, or glycosides [9]. Syringic acid, a naturally occurring phenolic compound (see Figure 1A found in olives, dates, grapes, acai palm, spices, and honey [10,11], exhibits diverse pharmacological effects, including antioxidant, anti-inflammatory, antimicrobial, antidiabetic, and anticancer activities, with protective roles in the liver, heart, and brain [4]; however, like many phenolic acids, it has poor aqueous solubility and is rapidly eliminated from the body, resulting in low bioavailability [12], as evidenced by its reduced absolute bioavailability (86.27%) when administered intravenously in rabbits, lower than the expected 100% [13]. Formulation studies for syringic acid achieved higher area under the curve values (120.58 ± 2.92 and 338.08 ± 3.65 g min mL^−1^ for pristine syringic acid and SA-liposome, respectively) but still exhibited the same Cmax and Tmax (4.50 ± 0.04 g mL^−1^ and 8 min, respectively) [12].

To overcome such limitations, various strategies have been explored to enhance the bioavailability of natural compounds, including salt formation, particle size reduction, solid dispersion, complexation, surfactant use, and crystal engineering [14]. Among these, drug delivery systems offer significant promise by improving drug solubility, modifying release profiles, enhancing permeability across biological barriers, and optimizing pharmacokinetics and biodistribution [15]. Despite progress, many conventional delivery systems still face formulation challenges. As a result, novel materials and approaches are being explored, including the use of inorganic and metallic-based frameworks. These highly porous materials offer advantages such as high drug-loading capacity, encapsulation efficiency, controlled release behavior, and improved tissue targeting, making them promising candidates for next-generation drug delivery platforms [16].

**Figure 2:**
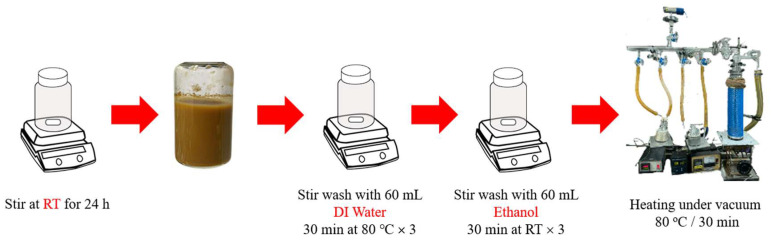
Schematic diagram of the MIL-100(Fe) synthesis using the method of Luo et al. [42].

Among these highly porous materials are metal–organic frameworks (MOFs). These are novel polymers made of metal ions or may come in metal clusters that form multidentate organic porous ligands [17]. They are widely used in biological sensing, catalysis, and gas storage due to the following characteristics: (1) high surface area; (2) tunable pore size; (3) three-dimensional rigid skeleton; (4) organic–inorganic hybrid nature; and (5) versatile crystal morphology. Different kinds of MOFs exist, such as ZIF-8(Zn), HKUST-1(Cu), Uio-66(Zr), Hf-MOF-888, Zn-MOF-3, CD-MOF-1, CD-MOF-2, Uio-66(Zr)-NO2, MIL-53(Fe), MIL-88(Fe), and MIL-100(Fe) [18]. Some of the drugs formulated with the MOF as a drug delivery system include ibuprofen, azidothymidine, cidofovir, gemcitabine, topotecan, isoniazid, doxycycline, tetracycline, docetaxel, lamivudine, cidofovir, exthoxysuccinatocisplatin, oridonin, caffeine, 5-fluorouracil, mitoxantrone, etilefrine, cisplatin, ketoprofen, lansoprazole, azilsartan, budesonide, valsartan, doxorubicin, and many more [19,20,21,22,23,24,25,26,27,28,29,30,31,32,33,34,35,36,37,38,39,40,41]. In particular, MIL-100(Fe) (Figure 1 and Figure 2) was used as the drug carrier in this study.

Therefore, this study aims to address the limited bioavailability of syringic acid. Specifically, this project aims to prepare and synthesize MIL-100(Fe), a MOF, as a drug delivery system for the natural product, syringic acid. The characterization of the prepared drug delivery system was performed based on its particle size, drug loading, surface morphology, BET surface area, and FT-IR. The in vitro drug release study of the drug delivery system was determined in four release media, namely, simulated gastric fluid pH 1.2, simulated intestinal fluid pH 6.8, phosphate-buffered saline pH 7.4, and water. The possible release mechanism of the preparation was determined using five drug release models. The toxicity of the preparation was tested based on the Organization for Economic Cooperation and Development (OECD) guidelines 423. Likewise, the relative bioavailability of the preparation was determined in the murine model. A comparison of the area under the curve using the pristine and incorporated syringic acid was used as a point of comparison to determine whether the use of the drug delivery system achieved its intended purpose. This project aims to enhance the oral bioavailability of syringic acid.

## 2. Materials and Methods

### 2.1. Materials and Reagents

Syringic acid (≥95%) was purchased from Sigma-Aldrich (St. Louis, MO, USA). Trimesic acid (benzene-1,3,5-tricarboxylic acid, ≥98%) was obtained from standard commercial suppliers. Iron (II) chloride tetrahydrate (FeCl_2_·4H_2_O), sodium hydroxide (NaOH), and ethanol (95%) were purchased locally. Deionized water was prepared using a Milli-Q ultrapure water system. All reagents and solvents were used without further purification.

### 2.2. Animals

Male Sprague Dawley (SD) rats (200–250 g) were obtained from the Laboratory Animal Facility of the Department of Science and Technology–Industrial Technology Development Institute (DOST–ITDI, Taguig City, Philippines). All animal test procedures were conducted at the Laboratory Animal Facility of the Department of Science and Technology–Industrial Technology Development Institute (DOST–ITDI, Taguig City, Philippines). The animals were housed under standard laboratory conditions with a controlled temperature of 25 ± 2 °C and relative humidity of 45 ± 5% and maintained on a 12 h light/dark cycle. Rats were given distilled water and standard laboratory chow ad libitum and were acclimatized for 7 days prior to experimentation. All animal procedures were reviewed and approved by the Institutional Animal Care and Use Committee (IACUC) of DOST–ITDI, in accordance with the guidelines set by the Bureau of Animal Industry (BAI) of the Philippines (AR-2024-0138). Prior to drug administration, animals were fasted for 12 h with free access to distilled water. All animal test procedures were supervised by a licensed veterinarian.

### 2.3. Methods

#### 2.3.1. Synthesis of MIL-100

The synthesis of MIL-100(Fe) was performed following the procedure outlined by Luo and co-workers [42]. The reaction was performed by dissolving 1.14 mmol (226.64 mg) of iron (II) chloride tetrahydrate, 0.79 mmol (166.01 mg) of trimesic acid, and 2.28 mmol (91.2 mg) of sodium hydroxide in 60 mL of deionized water. The mixture was stirred at room temperature for 24 h. The solution was centrifuged at 5000 rpm for 10 min to collect the MOF powder [43]. The MOF powder was washed with 60 mL of deionized water for 30 min at 80 °C three times, subjecting the MIL-100(Fe) powder to centrifugation at 5000 rpm for 10 min. The MOF powder was washed with 95% ethanol at 80 °C for 30 min, subjecting the MIL-100(Fe) powder to centrifugation at 5000 rpm for 10 min. The slurry pellet mixture was dried under vacuum at 80 °C for 30 min (as illustrated in Figure 2).

#### 2.3.2. Syringic Acid Impregnation and Quantification

The loading of the syringic acid to MIL-100(Fe) was performed through simple drug impregnation, as reported in separate studies by Cunha and co-workers and by Singco and co-workers [21,44].

Approximately 100 mg of MIL-100(Fe) was stirred with the syringic acid–ethanol solution (1 MOF:1 syringic acid) (100 µL solution per 1 mg MOF). The syringic acid entrapment protocol was carried out in 4 time frames (12 h, 24 h, 36 h, and 48 h) with a stirring speed of 75 rpm. After the given period of entrapment, the mixture was centrifuged at 10,000 rpm for 10 min. The supernatant liquid was separated and stored for further analysis. The MOF particle was washed with 12 mL of 95% ethanol to remove the superficially adsorbed syringic acid. The mixture was re-centrifuged at 10,000 rpm for 10 min to separate the particles from the supernatant liquid; the procedure of washing and centrifugation was performed three times. The preparation containing syringic acid impregnated into the MIL-100(Fe) was labeled as “SYA@MIL-100(Fe)”. The preparation was dried at 80 °C and stored for further analysis [44]. The MIL-100(Fe): syringic acid ratio was changed to 1:2 to determine the effect of increasing the syringic acid for entrapment.

The amount of syringic acid impregnated into MIL-100(Fe) was calculated by determining the unabsorbed syringic acid found in the supernatant liquid using high-performance liquid chromatography method using an HPLC Shimadzu 2050C PDA model with InertSustain C18 5 µm, 150 × 4.6 mm ID column (GL Science, Shinjuku-ku, Tokyo, 163-1130 Japan) equipped with a photodiode array (PDA) (Shimadzu, Suita, Japan). The detection wavelength was optimized at 272 nm with the flow rate of 1.0 mL min^−1^ using a 70:30 ratio of water: methanol with 0.1% acetic acid [44,45].

(1) $$Drug\;Loading\;Percent=\frac{TA-SA}{C}\;\times \;100$$

where *TA* is the total amount of syringic acid (mg), *SA* is the amount of syringic acid in the supernatant (mg), and *C* is the amount of MIL-100(Fe) used (mg).

#### 2.3.3. Characterization of SYA@MIL-100(Fe)

The sample with the highest percentage of drug loading across the 1:1 and 1:2 ratios was used for further characterization.

##### Nitrogen Sorption Isotherms

Samples were degassed under high vacuum at a final temperature of 350 °C for 6 h, following an initial evacuation at 90 °C for 1 h at a controlled temperature ramp rate of 10 °C min^−1^ under a vacuum using the nitrogen adsorption–desorption equipment to remove residual solvents adhered inside the MIL-100(Fe) and 77.35 K (−195.796 °C) for nitrogen adsorption using the Micromeritics ASAP 2020 Accelerated Surface Area and Porosimetry System (Micromeritics, Taipei, Taiwan). The Brunauer–Emmett–Teller (BET) specific surface area (S_BET_, m^2^ g^−1^) was determined from the linear region of the BET plot within the relative pressure range of 0.05–0.30 using N_2_ adsorption–desorption isotherms at −196 °C. The total pore volume (V_total_, cm^3^ g^−1^) was calculated from the amount of nitrogen adsorbed at a relative pressure (P/P_0_) of approximately 0.99, assuming complete pore filling [46].

##### Thermogravimetric Analysis (TGA)

Samples (5–10 mg) of the pristine MIL-100(Fe) and SYA@MIL-100(Fe) were placed into the ceramic pans and heated from 50 to 800 °C with a heat rate of 10 °C min^−1^ under a nitrogen atmosphere (20 mL min^−1^). Temperature vs. percent weight was graphed to determine the change between the pristine MIL-100(Fe) and SYA@MIL-100(Fe) [47].

##### Powder X-Ray Diffraction (PXRD)

Samples were subjected to PXRD to check the identity and structural integrity of MIL-100(Fe) after exposure to syringic acid for different loading times. PXRD analysis was performed using a Bruker D8 Phase diffractometer (Bruker, Taipei City, Taiwan) operating at 30 kV and 10 mA with monochromated Cu Kα radiation. Scans were conducted over a 2θ range of 5° to 50°, with a step size of 0.03° and a scan speed of 0.5–3.5 s per step [48,49].

##### Fourier Transform Infrared Spectroscopy (FTIR)

Samples were scanned at 400 to 4000 cm^−1^ using potassium bromide pellets. The functional groups of the samples were compared to check the presence of syringic acid in the framework.

##### Scanning Electron Microscopy (SEM)

Samples were dried under a vacuum and mounted on carbon double adhesive tape. The samples were coated with platinum under an argon atmosphere and at reduced pressure to increase the conductivity of the sample. The analysis was performed at 20,000× magnification with an accelerating voltage of 15,000 V using field emission scanning electron microscopy (JEOL JEM-700F) (JEOL, Hsinchu, Taiwan). Micropictographs were obtained for every sample [45,50].

##### Particle Size Determination

Samples were reconstituted with ultrapure water with a refractive index of 1.33 at 25 °C and 78,304 dielectric constants to make a 100 ppm concentration and sonicated for 10 min at 40 kHz to facilitate the distribution of the particles. Particle size was determined using the dynamic light scattering method (Nanopartica nanoparticle analyzer SZ-100V2) (Horiba Scientific, Ltd., Kyoto, Japan) [45,50].

#### 2.3.4. In Vitro Drug Release Study

Syringic acid and SYA@MIL-100(Fe) were subjected to an in vitro drug release study using the sample and separation method [45,51]. The release media used were ultrapure water, 0.1 N phosphate-buffered saline (PBS) pH 7.4 for simulated blood pH, PBS pH 6.8 for simulated intestinal fluid, and 0.1 N HCl pH 2.0 for simulated gastric fluid. An appropriate amount of the sample (the amount of sample did not exceed one-third of its solubility (g per mL) to reach the sink condition) was prepared for 100 mL of the release media. The setup was stirred at 75 rpm at 37.5 ± 0.5 °C. An aliquot of 1000 µL was taken at the time points of 5, 15, 30, 60, 120, 180, 240, 360, 1440, 1680, and 1800 min. A fresh amount of the media was used to compensate for the amount of the release media taken at every time point. The aliquots were centrifuged at 5000 rpm for 10 min. The supernatant liquid was stored for syringic acid quantification using HPLC analysis, while the pellets were returned to the setup. The cumulative amount of syringic acid released from the MIL-100(Fe) was utilized to predict and correlate the behavior of the in vitro release. The experimental data were fitted to five predictable models: zero-order, first-order, Higuchi, Korsmeyer–Peppas, and Hixson–Crowell models [52]. Data fitting was performed by linear regression using Microsoft Excel. The correlation coefficient (r^2^) was utilized as a criterion for selecting the best model that describes the release profile in the three media. An r^2^ value close to 1 signifies the best correlation.

#### 2.3.5. Acute Oral Toxicity

Acute oral toxicity was analyzed in accordance with the OECD guidelines for testing chemical compounds using the acute toxic class method (OECD procedure 423). Prior to dosing, 1 mL of the blood was extracted from the test animals (Male Sprague Dawley Rats, 200–250 g) using the tail vein method and was submitted for alanine aminotransferase (ALT), aspartate aminotransferase (AST), and blood urea nitrogen (BUN), and creatinine level determinations for signs of liver and kidney damages, respectively. A dose of 2000 mg kg^−1^ was given to three test animals, and they were observed for one week. Since no test animals were recorded to show any signs of toxicity or death, an additional three test animals were given the same dose. The test animals were observed for 14 days post-administration for signs of toxicity and death. After the 14 days of observation, the test animals were sedated using Zoletil 50 (50 mg tiletamine base, 50 mg zolazepam base, and 57.7 mg mannitol per mL) at a dose of 50 mg kg^−1^ prior to blood extraction using the cardiac puncture method. The animals were euthanized using a carbon dioxide chamber. The blood samples were analyzed for liver and kidney functions. The major organs, such as the liver and kidneys of the test animals, were harvested for tissue mounting and histopathological analysis.

#### 2.3.6. Oral Bioavailability and Tissue Distribution

The bioavailability study of syringic acid and tissue distribution in the liver and kidney was conducted according to the method of Ding et al., Sun et al., and Santos et al. [45,53,54]. The test animals were grouped into 10 groups (n = 3) for every time point (15, 30, 60, 120, 240, 480, 1080, 1440, 2880, and 4320 min). The test animals were dosed at 100 mg kg^−1^ bodyweight of syringic acid and an equivalent amount of syringic acid in SYA@MIL-100(Fe) orally, while 25 mg kg^−1^ bodyweight of syringic acid and 3 mg kg^−1^ bodyweight of SYA@MIL-100(Fe) were administered to a fasted test animal. The reduction in the dosing for the intraperitoneal route is to prevent abdominal distress in the test animal post-administration.

##### Collection and Processing of Blood Samples

After the given time points, the test animals were sedated using Zoletil 50 (50 mg tiletamine base, 50 mg zolazepam base, and 57.7 mg mannitol per mL) at a dose of 1 mg kg^−1^ before blood extraction using the cardiac puncture method. The blood was allowed to clot and centrifuged at 4000 rpm at 4 °C for 5 min to obtain the serum. The sera were mixed with an equal amount of methanol to precipitate proteins and centrifuged again to obtain the deproteinized sera. The procedure was repeated until no precipitation with methanol occurred. The protein-free sera were dried under reduced pressure for prior analysis using HPLC.

##### Collection and Processing of Organ Samples

After the blood collection from the test animals, the animals were euthanized, and the organs, such as the liver and kidneys, were taken. The organs were pat-dried and weighed to determine the amount of NSS to be used for homogenization (1 mL of NSS per 700 mg of organ). The organs were homogenized and mixed with equal amounts of absolute ethanol. The mixture was centrifuged at 5000 rpm for 10 min. The serum samples were supplemented with an equal amount of absolute ethanol to facilitate the precipitation of proteins. This procedure was performed until no precipitation occurred. The supernatant was allowed to dry under reduced pressure before HPLC analysis.

##### Syringic Acid Quantification

Samples were prepared using the mobile phase consisting of 80:20 water with 0.1% acetic acid: methanol. HPLC analysis was performed using a linear gradient elution at a flow rate of 1.0 mL min^−1^. The gradient program started at 80:20 (water with 0.1% acetic acid: methanol) and linearly progressed to 40:60 over 10 min. The column oven temperature was maintained at 30 °C. Detection was carried out using a PDA detector set at 218 nm.

#### 2.3.7. Statistical Analysis

All experiments were conducted in triplicate. Statistical analysis of significance was performed using SPSS version 20 statistical software (SPSS Inc., Chicago, IL, USA). Differences within the group were evaluated using a paired sample *t*-test, while differences between various groups were evaluated using one-way analysis of variance (ANOVA), and a *p*-value < 0.05 indicated statistical significance. Post hoc analysis was performed using Tukey HSD.

## 3. Results

### 3.1. Syringic Acid Impregnation and Quantification

**Figure 3:**
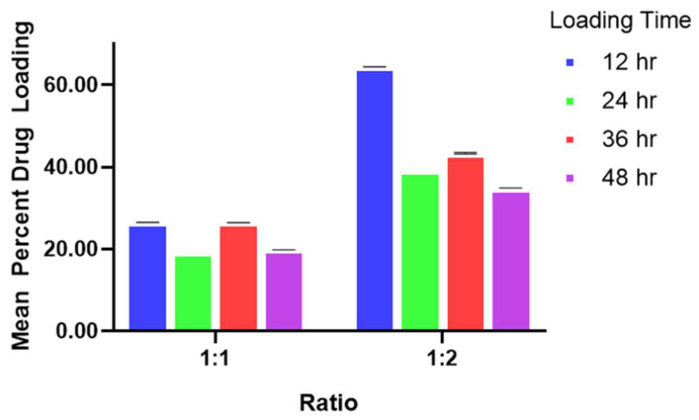
Mean drug loading efficiency of syringic acid at four different time points. Data presented as mean ± S.E.M. Error bars ± 1 S.E.M.

Syringic acid was impregnated into MIL-100(Fe) at 12, 24, 36, and 48 h at 1:1 (1 mg MIL-100(Fe): 1 mg syringic acid) and 1:2 (1 mg MIL-100(Fe): 2 mg syringic acid) ratios, as illustrated in Figure 3. The mean drug loading was highest at 12 h loading (26.48% ± 0.03%) (*p*-value less than 0.001), followed by 36 h (26.38 ± 0.02%), 48 h (19.79 ± 0.02%), and 24 h (19.28 ± 0.02%), with *p*-values of less than 0.001. Interestingly, the mean percent drug loading of syringic acid decreased with the constant value of the ratio of the MIL-100(Fe) to syringic acid, except for 12 h; although the change was significantly different (with a *p*-value of less than 0.001), the difference was not remarkable (deviation of 1.76%). The mean drug loading values of the 1:2 ratio are as follows: 64.42 ± 0.03% (12 h) > 43.30 ± 0.17% (36 h) > 39.12 ± 0.03% (24 h) > 34.78 ± 0.04% (48 h). In congruence with the entrapment efficiency data of the 1:2 ratio, the mean drug loading at 24 h and 48 h was deemed as non-significant, with a *p*-value of 0.922. Two-way ANOVA data suggested that the loading ratio and loading time have a significant effect on the drug loading of syringic acid in MIL-100(Fe), with a *p*-value of less than 0.001. Likewise, the combined effects of loading ratio and loading time on the drug loading were significant, with a *p*-value of less than 0.001. With the highest drug loading, this study utilized the 12 h loading time at a 1:2 ratio.

### 3.2. Powder X-Ray Diffraction (PXRD)

**Figure 4:**
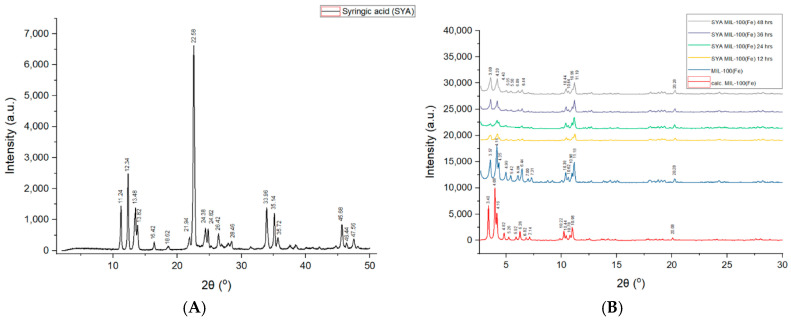
Powder X-ray diffraction (pXRD) patterns of (**A**) syringic Acid and (**B**) MIL-100(Fe) and syringic acid-loaded MIL-100(Fe) (SYA@MIL-100(Fe)) at 4 time points.

PXRD analysis of the synthesized MIL-100(Fe) confirmed the successful synthesis of the intended MOF, as evidenced by the presence of characteristic Bragg peaks at 2θ values of 3.40, 4.00, 4.16, 4.82, 5.26, 5.92, 6.82, 7.14, 10.22, 10.44, 10.78, 10.98, and 20.08. These Bragg peaks align well with previously reported patterns for MIL-100(Fe), consistent with its simulated crystalline structure [55], although minor deviations from the expected Bragg positions were observed. Importantly, these Bragg peaks were conserved after drug loading at different time points (12, 24, 36, and 48 h), suggesting that the MIL-100(Fe) structure remains stable and crystalline despite exposure to syringic acid, as shown in Figure 4B. The preservation of these diffraction patterns confirms that the framework does not collapse upon syringic acid incorporation.

Additionally, the absence of any new diffraction peaks corresponding to crystalline syringic acid (compared to the pure drug pattern in Figure 4A) suggests that no unentrapped or recrystallized drug is present on the surface of MIL-100(Fe). This absence of free syringic acid peaks supports the conclusion that syringic acid is successfully encapsulated within the MIL-100(Fe) pores, rather than simply being adsorbed or precipitated externally. These findings align with previous studies demonstrating the capability of MIL-100(Fe) to retain its structure during the encapsulation of guest molecules due to its high porosity and robust framework stability.

### 3.3. Fourier Transform Infrared Spectroscopy (FTIR)

**Figure 5:**
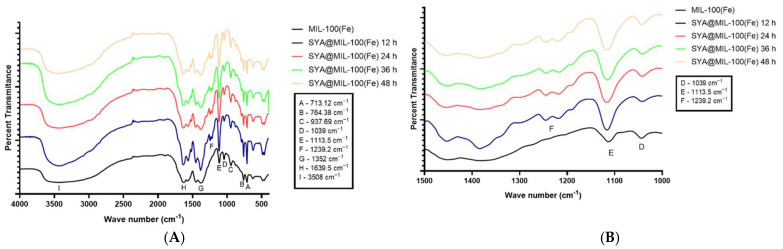
FTIR Spectra of MIL-100(Fe) and syringic acid-loaded MIL-100(Fe) (SYA@MIL-100(Fe)) at 4 time points (**A**) illustrated at 400 to 4000 cm^−1^ and (**B**) at 1000 to 1500 cm^−1^.

The FTIR spectra of MIL-100(Fe), SYA@MIL-100(Fe), and syringic acid were noted, as illustrated in Figure 5. The spectra of MIL-100(Fe) and SYA@MIL-100(Fe) at different time points displayed prominent peaks at 3508 cm^−1^ (Peak I)(O–H stretching from hydrogen-bonded dimers), 1639.5 cm^−1^ (Peak H) (C=C stretching), 1352 cm^−1^ (Peak G) (C–H rocking), 1113.5 cm^−1^ (Peak E) (C–O stretching), 1039 (Peak D) and 937.69 cm^−1^ (Peak C) (O–H bending), and 764.38 (Peak B) and 713.12 cm^−1^ (Peak A) (C–H out-of-plane bending). These peaks correspond to functional groups present in trimesic acid, the organic ligand of MIL-100(Fe).

The functional groups of syringic acid and trimesic acid are notably similar; however, syringic acid contains unique para-hydroxyl and ortho-methoxy substituents on the aromatic ring. The ortho-methoxy substituent was detected at 1239.2 cm^−1^ (Peak F). As shown in Figure 5A, a key difference between MIL-100(Fe) and SYA@MIL-100(Fe) is the appearance of a distinct peak in the 1239.2 cm^−1^ (Peak F) (expanded at 1000–1500 cm^−1^), which is observed only in the SYA-loaded samples (from 12 to 48 h of loading time).

This band corresponds to the C–O stretching of an aryl–OCH_3_ group, indicative of the methoxy substituents on syringic acid. Since this functional group is absent in trimesic acid, its presence in SYA@MIL-100(Fe) suggests a possibility of successful loading of syringic acid into the framework.

### 3.4. Nitrogen Adsorption–Desorption

**Figure 6:**
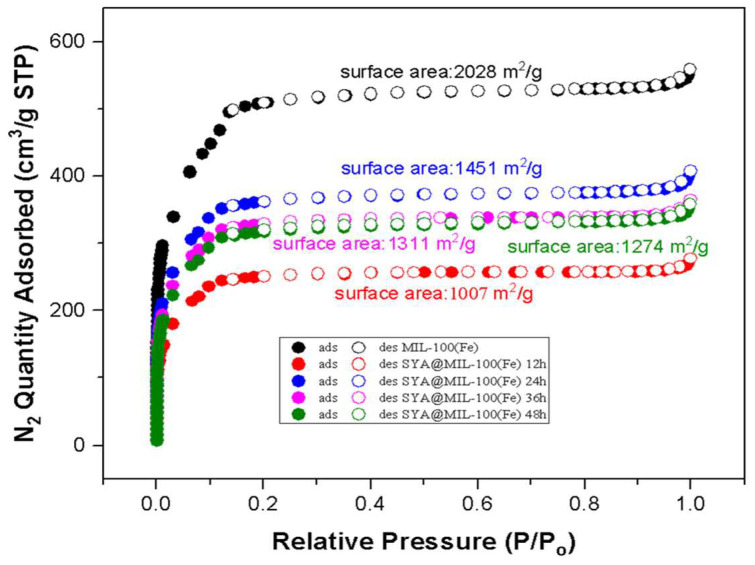
Nitrogen adsorption–desorption isotherm MIL-100(Fe) and syringic acid-loaded MIL-100(Fe) (SYA@MIL-100(Fe)) at 4 time points.

Changes in the BET surface area of the unloaded MIL-100(Fe) and syringic acid-loaded MIL-100(Fe), from 2028 m^2^ g^1^ to 1451 m^2^ g^1^ (12 h), 1311 m^2^ g^1^ (24 h), 1274 m^2^ g^1^ (36 h), and 1007 m^2^ g^1^ (48 h), indicated the successful impregnation of syringic acid into the framework, as depicted in Figure 6.

Changes in the BET surface area of the unloaded MIL-100(Fe) and SYA@MIL-100(Fe) were noted from 2028 m^2^ g^1^ to 1451 m^2^ g^1^ (12 h) (28.45% reduction), 1311 m^2^ g^1^ (24 h) (35.36% reduction), 1274 m^2^ g^1^ (36 h) (37.18% reduction), and 1007 m^2^ g^1^ (48 h) (50.35% reduction), indicating the successful impregnation of syringic acid into the internal surface or a blockage of the pore opening, as depicted in Figure 5. Table 1 summarizes the other textural characteristics, including total pore volume (cm^3^ g^−1^), micropore volume (cm^3^ g^−1^), mesoporous volume (cm^3^ g^−1^), and pore width (Å) of the MIL-100(Fe) and SYA@MIL-100(Fe). Reductions in the total pore volume were also noted in the samples, from 0.855916 cm^3^ g^−1^ (MIL-100(Fe)) to 0.422034 cm^3^ g^−1^ (12 h) (50.69% reduction), 0.619344 cm^3^ g^−1^ (24 h) (27.64% reduction), 0.555394 cm^3^ g^−1^ (36 h) (35.11% reduction), and 0.546359 cm^3^ g^−1^ (48 h) (36.17% reduction).

Changes in the micropore volume between the MIL-100(Fe) and SYA@MIL-100(Fe) were also noted, from 0.297397 cm^3^ g^−1^ (MIL-100(Fe)) to 0.231148 cm^3^ g^−1^ (12 h) (22.28% reduction), 0.400067 cm^3^ g^−1^ (24 h) (34.52% increase), 0.373316 cm^3^ g^−1^ (36 h) (25.53% increase), and 0.339412 cm^3^ g^−1^ (48 h) (14.13% increase). Reductions in the mesopore volume of MIL-100(Fe) after syringic acid loading were also noted, from 0.558519 cm^3^ g^−1^ (MIL-100(Fe)) to 0.190886 cm^3^ g^−1^ (12 h) (65.82% reduction), 0.219277 cm^3^ g^−1^ (24 h) (60.74% reduction), 0.182078 cm^3^ g^−1^ (36 h) (67.40% reduction), and 0.206947 cm^3^ g^−1^ (48 h) (62.95% reduction). Increases in the pore width of the MIL-100(Fe) were noted after the incorporation of syringic acid at all time points, from 29.717 Å to 35.259 Å (12 h), 37.399 Å (24 h), 35.937 Å (36 h), and 37.102 Å (48 h), with corresponding percent increases of 35.26%, 37.40%, 35.94%, and 37.10%, respectively. (See Supplementary Material for the raw data of the nitrogen adsorption–desorption analysis). 

### 3.5. Thermogravimetric Analysis (TGA)

**Figure 7:**
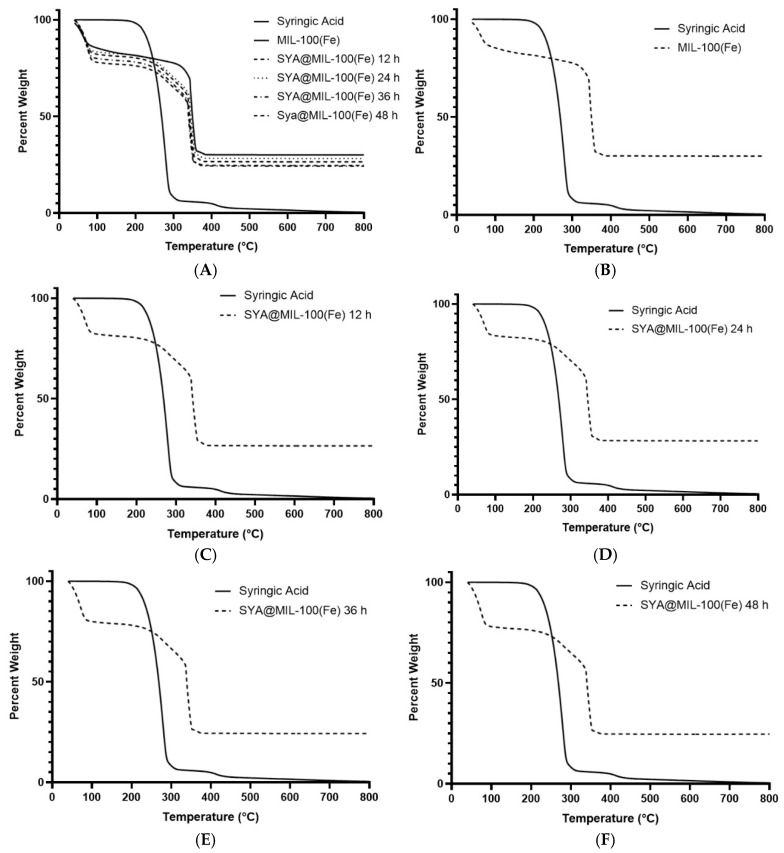
Thermograms comparing the following: (**A**) syringic acid, MIL-100(Fe), and SYA@MIL-100(Fe) at four time points; (**B**) syringic acid and MIL-100(Fe); (**C**) syringic acid and SYA@MIL-100(Fe) at 12 h; (**D**) syringic acid and SYA@MIL-100(Fe) at 24 h; (**E**) syringic acid and SYA@MIL-100(Fe) at 36 h; and (**F**) syringic acid and SYA@MIL-100(Fe) at 48 h.

The thermogram of the syringic acid with MIL-100(Fe) and SYA@MIL-100(Fe) at different time points is illustrated in Figure 7A. Specifically, a comparative thermogram of syringic acid with MIL-100(Fe) (Figure 7B) and SYA@MIL-100(Fe) at different time points is shown (Figure 7C–F). Lastly, the residual weight noted with MIL-100(Fe) after the analysis was 30.08%, while lower residual weights were noted with SYA@MIL-100(Fe), specifically 26.53% (12 h), 28.23% (24 h), 24.29% (36 h), and 24.68% (48 h).

### 3.6. Surface Morphology and Particle Size Analysis

**Figure 8:**
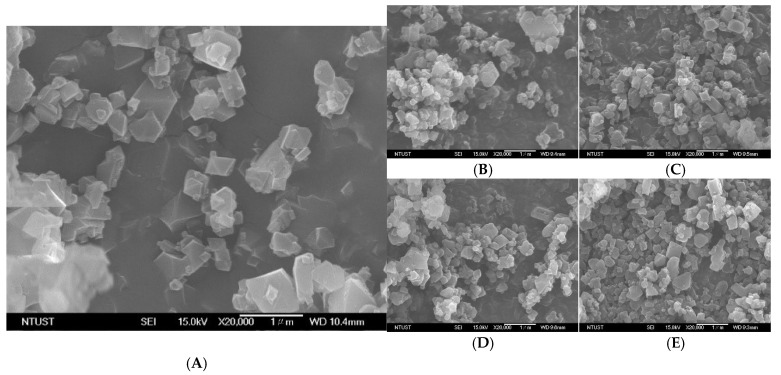
Micropictographs of (**A**) MIL-100(Fe) and (**B**) SYA@MIL-100(Fe) at 12 h; (**C**) SYA@MIL-100(Fe) at 24 h; (**D**) SYA@MIL-100(Fe) at 36 h; and (**E**) SYA@MIL-100(Fe) at 48 h taken at 10 kV and 20,000× magnification.

The results of the SEM are presented in Figure 8A–E. MIL-100(Fe) exhibits a triangular-based pyramid shape that enhances drug absorption by maximizing surface area for drug loading, thus potentially improving therapeutic efficacy (Figure 8A). After the impregnation of syringic acid at 12, 24, 36, and 48 h, the surface morphology of the particle did not significantly change in congruence with the PXRD data (Figure 8B–E). The mean particle size of MIL-100(Fe) was reported as 270.67 nm ± 2.60 nm, but upon loading with syringic acid, the mean particle sizes were 272.03 nm ± 1.33 nm (12 h post-loading), 269.67 nm ± 3.07 nm (24 h post-loading), 268.30 nm ± 0.40 nm (36 h post-loading), and 176.97 nm ± 4.67 nm (48 h post-loading). 

### 3.7. In Vitro Drug Release

**Figure 9:**
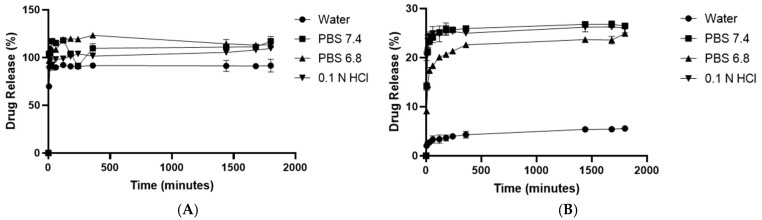
Cumulative drug release of (**A**) syringic acid and (**B**) SYA@MIL-100(Fe) in 4 different media. Water; HCl—hydrochloric acid; and PBS—phosphate-buffered saline at pH 7.4 and 6.8. Data presented as mean ± S.E.M.

The in vitro drug release of syringic acid was carried out using water, phosphate-buffered saline (PBS) at pH 7.4 and pH 6.8, and 0.1 N hydrochloric acid, as shown in Figure 9. After 30 h, the cumulative release percentages of syringic acid were as follows: in water (94.67%), PBS pH 7.4 (117.55%), PBS pH 6.8 (110.93%), and 0.1 N HCl (110.99%). Similarly, for SYA@MIL-100(Fe), the cumulative releases were 5.57% (water), 27.57% (PBS pH 7.4), 55.42% (PBS pH 6.8), and 25.80% (0.1 N HCl). With these cumulative values of syringic acid released, it was deduced that there was a very slow release of syringic acid in the simulated release media. The data were plotted to determine the release kinetic profile at zero-order kinetics, first-order kinetics, the Higuchi model, the Korsmeyer–Peppas model, and the Hixson–Crowell model.

### 3.8. Acute Oral Toxicity

Acute oral toxicity was measured to check the preliminary toxicity of syringic acid based on the OECD 423 guidelines. The result indicates that the test samples at 2000 mg kg^−1^ were noted to be safe, as the test animals survived the 14 days of observation. Serological test revealed significant reduction in the SGPT values after the administration of syringic acid (87.96 ± 14.9 to 46.73 ± 3.66 units per liter at *p* = 0.02), but there was no significant difference after the administration of SYA@MIL-100(Fe) (63.57 ± 8.53 to 47.33 ± 1.88 units per liter at *p* = 0.099). Significant reductions in the SGOT levels were noted for the both samples after administration (syringic acid: 337.30 ± 34.87 to 200.63 ± 19.17 units per liter at *p* = 0.01; and SYA@MIL-100(Fe): 268.32 ± 21.37 to 168.43 ± 25.33 units per liter at *p* = 0.01). Non-significant changes in the BUN levels were noted for both samples after administration (syringic acid: 14.71 ± 0.51 to 15.57 ± 1.59 mg per dL at *p* = 0.548; and SYA@MIL-100(Fe): 13.74 ± 0.63 to 14.42 ± 1.33 mg per dL at *p* = 0.683). Likewise, the same observation was noted with serum creatinine levels after administration for both samples (syringic acid: 0.58 ± 0.01 to 0.62 ± 0.12 mg per dL at *p* = 0.753; and SYA@MIL-100(Fe): 0.50 ± 0.02 to 0.61 ± 0.10 mg per dL at *p* = 0.299). This indicates that the sample of syringic acid (pristine and loaded) has a positive effect on the liver enzyme functions but not on the kidney functions. The serological analysis suggests that the samples were not toxic at the serological level. Histopathological analysis revealed distinct histopathological changes in liver and kidney structures across various rat models, with findings including mild to moderate hepatocellular degeneration and bile duct proliferation in the liver and mild to moderate glomerular morphology and tubular degeneration in the kidneys. The cellular damage was determined not to be induced by the test sample due to the notable decrease in the serological analysis, which might be due to the sample preparation of the organs. With the OECD guidelines, the preparation was classified as a “low-toxicity” class.

### 3.9. Bioavailability

The bioavailability of syringic acid was determined by administering syringic acid alone and SYA@MIL-100(Fe). The pharmacokinetic parameters of syringic acid and SYA@MIL-100(Fe) were computed based on the non-compartment model and are shown in Table 2. Figure 6 shows the mean plasma concentration–time curve of syringic acid in Sprague Dawley rats following both oral (100 mg kg^−1^) and intraperitoneal (3 mg kg^−1^) administration (data used in the graph are equivalent to 100 mg kg^−1^). To gain insights into the plasma concentration of syringic acid present in a certain amount of time and to show systemic exposure to syringic acid, the area under the curve (AUC) was determined. Table 2 summarizes the pharmacokinetic parameters of syringic acid and SYA@MIL-100(Fe) obtained from the experimental data.

**Figure 10:**
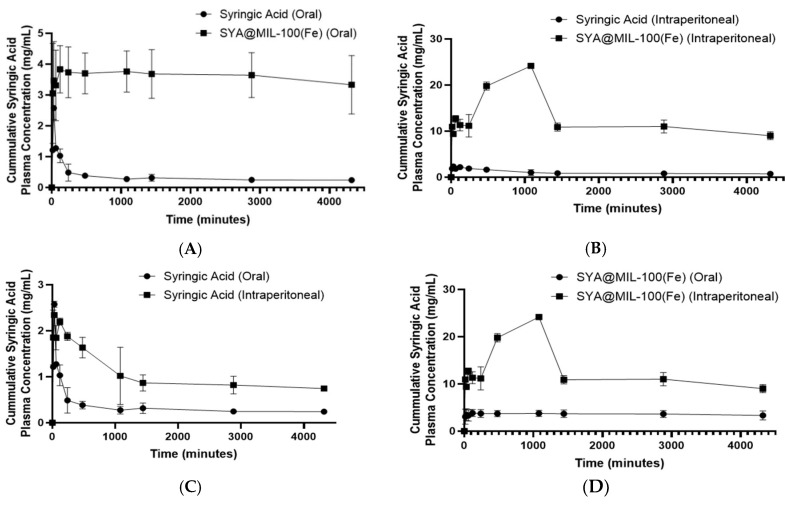
Area under the curve (AUC_0–72_) graph of (**A**) syringic acid (oral) and SYA@MIL-100(Fe) (oral), (**B**) syringic acid (intraperitoneal) and SYA@MIL-100(Fe) (intraperitoneal), (**C**) syringic acid (oral) and syringic acid (intraperitoneal), and (**D**) SYA@MIL-100(Fe) (oral) and SYA@MIL-100(Fe) (intraperitoneal) in blood samples. Data plotted as mean ± S.E.M. for time (min) versus mean plasma concentration of syringic acid (mg mL^−1^).

The area under the curve for the 0 to 72 h time points (AUC_0–72_) of the syringic acid plasma concentration following oral administration of syringic acid and SYA@MIL-100(Fe) and the intraperitoneal route of administration is shown in Figure 10A–D. The AUC_0–72_ of administration was significantly higher in SYA@MIL-100(Fe) (15,606 ± 1936.03 mg min mL^−1^) compared to syringic acid (1419 ± 142.15 mg min mL^−1^) alone when given through the oral route, with a *p*-value of 0.02. In contrast, the AUC for the 0 to infinity time points (AUC_0–∞_) showed no significant difference between SYA@MIL-100(Fe) and syringic acid (131,269.97 ± 61,666.27 mg min mL^−1^ and 1460 ± 143.84 mg min mL^−1^, respectively), with a *p*-value of 0.103. The area under the curve for the 0 to 72 h time points (AUC_0–72_) determined in the samples administered orally in the blood was significantly higher in SYA@MIL-100(Fe) (15,606 ± 1936.03 mg min mL^−1^) compared to syringic acid (1419 ± 142.15 mg min mL^−1^) alone, with a *p*-value of 0.02. In contrast, the AUC for the 0 to infinity time points (AUC_0–∞_) showed no significant difference between SYA@MIL-100(Fe) and syringic acid (131,269.97 ± 61,666.27 mg min mL^−1^ and 1460 ± 143.84 mg min mL^−1^, respectively), with a *p*-value of 0.103. In congruence with the data of AUC_0–72_, significant differences were observed at the maximum concentration (C_max_) and the time to reach the maximum concentration (T_max_), with *p*-values of 0.036 and 0.039, respectively. Between the two test groups, SYA@MIL-100(Fe) showed a significantly higher C_max_ (3.79 ± 0.43 mg mL^−1^) and T_max_ (549.02 ± 159.41 min) compared to syringic acid alone (2.33 ± 0.19 mg mL^−1^—C_max_ and 66.78 ± 7.56 min). There was no significant difference in the elimination half-life (T_1/2_) between the two groups, with a *p*-value of 0.84 (SYA@MIL-100(Fe): 24,392.75 ± 10,593.37 min vs. syringic acid: 118.77 ± 30.76 min). The intraperitoneal route of administration showed a significant difference in all pharmacokinetic parameters (*p*-values less than 0.05). The AUC_0–72_ of samples administered through the intraperitoneal route in the blood was significantly higher in SYA@MIL-100(Fe) (56,022.33 ± 2240.13 mg min mL^−1^) compared to syringic acid (4368.33 ± 489.25 mg min mL^−1^) alone when given through the oral route, with a *p*-value less than 0.05. Likewise, the AUC_0–∞_ of SYA@MIL-100(Fe) (206,758.55 ± 31,210.34 mg min mL^−1^) was significantly higher compared to syringic acid alone (5400.84 ± 964.81 mg min mL^−1^), with a *p*-value of 0.03. SYA@MIL-100(Fe) showed significantly higher values for C_max_, T_max_, and T_1/2_ (26.54 ± 0.55 mg mL^−1^, 1236.64 ± 91.50 min, and 11,504.68 ± 2306 min, respectively) compared to syringic acid alone (2.55 ± 0.04 mg mL^−1^, 5.597 ± 2.11 min, and 999.64 ± 410 min, respectively).

**Figure 11:**
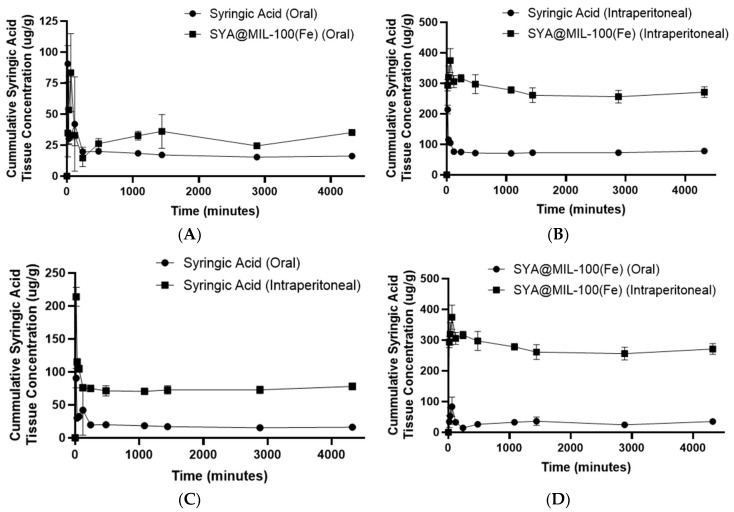
Area under the curve (AUC_0–72_) graph of (**A**) syringic acid (oral) and SYA@MIL-100(Fe) (oral), (**B**) syringic acid (intraperitoneal) and SYA@MIL-100(Fe) (intraperitoneal), (**C**) syringic acid (oral) and syringic acid (intraperitoneal), and (**D**) SYA@MIL-100(Fe) (oral) and SYA@MIL-100(Fe) (intraperitoneal) in kidney samples. Data plotted as mean ± S.E.M. for time (min) versus mean tissue concentration of syringic acid (µg g^−1^).

The area under the curve for the 0 to 72 h time points (AUC_0–72_) of the syringic acid kidney concentration following oral administration of syringic acid and SYA@MIL-100(Fe) and the intraperitoneal route of administration is shown in Figure 11A–D. The AUC0–72 determined in the samples administered orally in the kidney was significantly higher in SYA@MIL-100(Fe) (130,698 ± 7713.74 µg min g^−1^) compared to syringic acid (77,103.33 ± 2531.03 µg min g^−1^) alone, with a *p*-value of 0.03. Likewise, the AUC for the 0 to infinity time points (AUC_0–∞_) showed a significant difference between SYA@MIL-100(Fe) and syringic acid (144,112.76 ± 8494.44 µg min g^−1^ and 78,035.27 ± 2458.81 µg min g^−1^, respectively), with a *p*-value of 0.02. No significant difference in the maximum concentration (C_max_) was observed between syringic acid (105.36 ± 9.79 µg g^−1^) and SYA@MIL-100(Fe) (89.27 ± 17.80 µg g^−1^), with a *p*-value of 0.473. In congruence with the AUC data, the time to reach the maximum concentration (T_max_) and elimination half-life (T_1/2_) were significantly higher in SYA@MIL-100(Fe) (97.30 ± 9.65 min and 265.21 ± 22.01 min, respectively) compared to syringic acid alone (28.21 ± 0.33 min and 37.56 ± 4.69 min, respectively), with *p*-values less than 0.05.

For the intraperitoneal route of administration using the kidney samples, significant differences were noted with AUC_0–72_, C_max_, and T_max_, with *p*-values of less than 0.05. SYA@MIL-100(Fe) showed significantly higher pharmacokinetic parameters (1,169,999.33 ± 36,457.71 µg min g^−1^, 393.47 ± 14.44 µg g^−1^, and 118.08 ± 0.86 min, respectively) compared to syringic acid alone (321,104.67 ± 11,949.32 mg min mL^−1^, 218.21 ± 8.73 µg g^−1^, and 24.15 ± 0.96 min, respectively). No significant differences in AUC_0–∞_ and T_1/2_ were noted between SYA@MIL-100(Fe) (2,971,634.10 ± 1,163,138.16 µg min g^−1^ and 2799.01 ± 1717.60 min, respectively) and syringic acid alone (808,296.10 ± 429,472.17 µg min g^−1^ and 4805.81 ± 3271.08 min, respectively), with *p*-values of more than 0.05.

**Figure 12:**
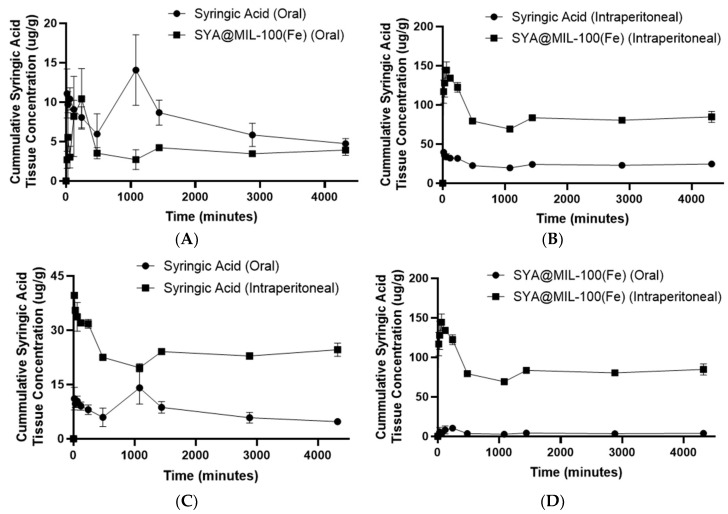
Area under the curve (AUC_0–72_) graph of (**A**) syringic acid (oral) and SYA@MIL-100(Fe) (oral), (**B**) syringic acid (intraperitoneal) and SYA@MIL-100(Fe) (intraperitoneal), (**C**) syringic acid (oral) and syringic acid (intraperitoneal), and (**D**) SYA@MIL-100(Fe) (oral) and SYA@MIL-100(Fe) (intraperitoneal) in liver samples. Data plotted as mean ± S.E.M. for time (min) versus mean tissue concentration of syringic acid (µg g^−1^).

The area under the curve for the 0 to 72 h time points (AUC_0–72_) of the syringic acid liver concentration following oral administration of syringic acid and SYA@MIL-100(Fe) and the intraperitoneal route of administration is shown in Figure 12A–D. The AUC_0–72_ determined in the samples administered orally in the liver was significantly higher in syringic acid (32,000.33 ± 3544.16 µg min g^−1^) compared to SYA@MIL-100(Fe) (17,309.33 ± 1351.33 µg min g^−1^) alone, with a *p*-value of 0.018. Likewise, the AUC for the 0 to infinity time points (AUC_0–∞_) showed a significant difference between the syringic acid and SYA@MIL-100(Fe) (47,401.91 ± 8515.77 µg min g^−1^ and 22,623.03 ± 2085.19 µg min g^−1^, respectively), with a *p*-value of 0.048. No significant difference in the maximum concentration (C_max_) and elimination half-time (T_1/2_) was observed with between syringic acid (162,757 ± 3.54 µg g^−1^, and 2288.81 ± 812.6 min, respectively) and SYA@MIL-100(Fe) (11.42 ± 2.30 µg g^−1^, and 913.33 ± 223.58 min, respectively), with *p*-values of 0.31 and 0.18, respectively. Similarly, the maximum concentration (T_max_) was significantly higher for syringic acid (515.96 ± 4.44 min) compared to SYA@MIL-100(Fe) alone (385.57 ± 42.40 min), with a *p*-value of 0.038.

For the intraperitoneal route of administration using the liver samples, significant differences in AUC_0–72_, AUC_0–∞_, C_max_, and T_max_ were noted, with *p*-values of less than 0.001 and 0.003. SYA@MIL-100(Fe) showed significantly higher pharmacokinetic parameters (363,982.67 ± 5429.14 µg min g^−1^, 522,988.13 ± 44,624.56 µg min g^−1^, 150.26 ± 6.65 µg g^−1^, and 111.01 ± 4.91 min, respectively) compared to syringic acid alone (102,784.67 ± 1510.75 µg min g^−1^, 166,522.15 ± 34,487.20 µg min g^−1^, 40.13 ± 0.56 µg g^−1^, and 24.70 ± 0.21 min, respectively). No significant difference in T_1/2_ was noted for syringic acid (1852.40 ± 1105.09 min) and SYA@MIL-100(Fe) (1309.25 ± 359.93 min), with a *p*-value of 0.67.

## 4. Discussion

**Figure 13:**
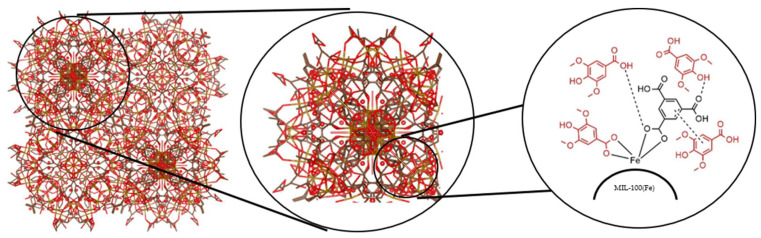
Predicted binding of syringic acid to the MIL-100(Fe) framework using hydrogen bonding, π-π interaction, and iron complexation.

This is the first study to utilize an iron-based MOF as a drug carrier for syringic acid. MIL-100(Fe) exhibited a sudden capture of syringic acid in its framework, accounting for 64.42 ± 0.03% (~0.6442 mg of syringic acid per 1 mg of MIL-100(Fe)) during the 12 h loading time, but it decreased during the succeeding hours: 43.30 ± 0.17% (36 h) > 39.12 ± 0.03% (24 h) > 34.78 ± 0.04% (48 h) at a 1:2 ratio of MIL-100(Fe) to syringic acid. This phenomenon may be due to the potential framework collapse with the interaction with the nucleophilic center found in syringic acid [45,56], leading to ligand exchange or competition between the organic linker, trimesic acid, and syringic acid [57]. Decreased drug loading (12 h loading (26.48% ± 0.03%) > 36 h (26.38% ± 0.02%) > 48 h (19.79% ± 0.02%) > 24 h (19.28% ± 0.02%)) was observed using the 1:1 ratio, mainly due to the lower concentration of syringic acid available for impregnation. Research has confirmed that increasing the available drug or guest molecule can increase the drug loading into a carrier [58,59]. The remarkable drug loading of syringic acid with MIL-100(Fe) may be due to the physical properties of MIL-100(Fe) and the interaction of syringic acid with the framework. MIL-100(Fe) has been experimentally determined at 2028 m^2^ g^1^ (BET surface area), 0.855916 cm^3^ g^−1^ (total pore volume), and 29.717 Å (pore width). This phenomenon has been supported by numerous studies [60,61,62,63,64]. Considering the particle diameter of syringic acid is 7.17 Å (as calculated via semi-empirical optimization and energy minimization, using Chem3D Pro, version 9.0), this smaller particle diameter permits its entry into the pore window of MIL-100(Fe), having a 29.717 Å pore size, as computed using the nitrogen desorption–adsorption isotherm [65,66]. Lastly, the internal environment within the MIL-100(Fe) is considered amphiphilic, thus allowing the incorporation of both hydrophilic and hydrophobic drugs [45,67]. Figure 13 shows the possible guest interaction, which includes hydrogen bonding, π-π interaction, and iron complexation with phenolic rings [45,68,69,70]. Hydrogen bonding can occur with the hydroxyl and carboxyl groups present in syringic acid, along with the carboxyl group of trimesic acid. The π-π interaction can occur between syringic acid and the organic ligand. Finally, complexation of the iron moiety from MIL-100(Fe) with the nucleophilic oxygen in syringic acid occurs. This is evident by the change in color observed after exposing MIL-100(Fe) to syringic acid (pristine MIL-100(Fe), which has a reddish brown color, compared to SYA@MIL-100(Fe), which has a dark to bluish reddish brown color). Another possible mechanism is through electrostatic interaction [71].

Other drugs that utilize MIL-100(Fe) as a framework are the following: oxaliplatin [72], curcumin [73], cyclophosphamide [74], chloroquine [75], doxycycline, tetracycline [39], cephalexin [76], lamivudine [77], and aceclofenac [78]. These drugs are bigger molecules compared to syringic acid. Thus, syringic acid, having a smaller particle diameter, can be entrapped inside MIL-100(Fe). These findings are in congruence with the study of Santos and co-workers [45]. Compared to the other drug delivery systems reported for syringic acid, the entrapment efficiency of the preparation was lower compared to that of Lin and co-workers (2020), who utilized mPEG-PLGA-PLL nanoparticles (EE of 92.69 ± 2.73%) [79], that of Sun and co-workers (2021), who utilized SMEDDS (EE of 98.04 ± 1.39%) [54], and that of Liu and co-workers (2019), who utilized TPGS Liposome (96.48 ± 0.76%) [12]. However, the studies did not report the use of percent drug loading, which measures the capacity of a given nanoparticle to carry or entrap a given drug. Shen and co-workers (2017) reported that varying the amount of drug during the drug loading affects the drug loading and entrapment efficiency [80]. The increase in the loading time also affects the amount of drug that can be incorporated into the framework, up to a certain degree, before the drug can alter the crystallinity of the framework [45].

A change in the intensity of the Bragg peaks in the PXRD after the encapsulation of syringic acid was observed in this research. The observed redistribution of the main peak intensities indeed provides strong evidence for the successful encapsulation of syringic acid molecules within the pores of the MIL-100(Fe). The presence of these guest molecules alters the electron density distribution within the unit cell, which, in turn, changes the structural factors (Fhkl) for different crystallographic planes. This directly affects the relative intensities of the diffraction peaks, while the unchanged peak positions confirm the retention of the framework’s crystallinity and structural integrity. This result is in congruence with the previous reported research using MIL-100(Fe), where drug impregnation (e.g., of 5-fluorouracil or caffeine) led to noticeable redistribution of main peak intensities (e.g., changes in the (022):(357) ratio), while peak positions remained constant, implying that structural factors changed due to electron density shifts induced by guest molecules, while the framework retained full crystallinity [81]. The observed discrepancy between the calculated and synthesized MIL-100(Fe) was observed in this study. The difference in relative intensities between the experimentally obtained pattern and the theoretically calculated one, particularly for the (220) and (311) reflections, is a well-documented phenomenon in powder diffraction of porous materials, which is attributed to the residual molecules present inside the pores. The calculated pattern assumes a perfectly “empty” and activated framework. Our as-synthesized MIL-100(Fe), even after activation, may still contain a small amount of residual solvent or water molecules trapped within the pores. Similar to the syringic acid loading, these residual guests will also modulate the peak intensities compared to the ideal calculated pattern [43].

While FTIR provides strong evidence for the incorporation of syringic acid, it cannot fully confirm whether the molecule is encapsulated within the pores of MIL-100(Fe) or merely adsorbed onto its external surface. Despite extensive washing to remove unbound syringic acid, additional characterization techniques, such as TGA, BET surface area measurements, or PXRD, are required to confirm the mode of interaction and verify true encapsulation.

The observed reduction in the total pore volume indicates that syringic acid occupies the pore space, which reduces the pore volume. The observed reduction in the micropore volume between the unloaded MIL-100(Fe) and SYA@MIL-100(Fe) at 12 h suggests that syringic acid was successfully impregnated into the microporous framework of MIL-100(Fe), indicating that syringic acid occupied the inner cavities or blocked the micropore entrances. On the contrary, increases in the micropore volume were observed for SYA@MIL-100(Fe) at 24 h, 36 h, and 48 h. This increase can be attributed to the displacement of residual solvents or moisture, pore opening or framework rearrangement, partial degradation or surface etching, and improved loading of syringic acid deeper into the framework. It is important to note that the increase in micropore volume at later time points does not imply reduced loading but may reflect improved pore accessibility or partial redistribution of the guest molecules.

The findings on the reduction in the mesoporous volume support the earlier discussion that syringic acid selectively impregnated the mesoporous cavities rather than the smaller microporous cavities. Although slight increases in mesopore volume were noted with extended loading times (24–48 h), the mesoporous volume remained significantly lower than that of the unloaded MIL-100(Fe). This trend suggests that at early stages (12 h), SYA primarily fills mesoporous cavities, while at later stages, molecules may reorganize or diffuse deeper into the framework, balancing mesopore and micropore occupancy. This sustained decrease suggests that syringic acid molecules predominantly occupied the larger internal cages of MIL-100(Fe), which are accessible through smaller microporous windows but offer sufficient space to host bulkier guest molecules. Such occupancy would explain the partial recovery of micropore volume seen in later time points, as syringic acid may have redistributed deeper into the mesoporous cavities, thereby freeing the micropore entrances. These findings are consistent with the framework’s hierarchical porosity and reinforce the idea of internal encapsulation rather than surface-level adsorption.

The substantial decrease from MIL-100(Fe) to SYA@MIL-100(Fe) across all loading time points for the mesoporous volume supports the hypothesis that syringic acid specifically impregnated the mesoporous cavities of MIL-100(Fe), rather than the smaller micropore spaces. The reductions in mesopore volumes, even after prolonged loading durations, indicate that the syringic acid is retained within the internal mesoporous cages rather than being superficially adsorbed. This behavior suggests that syringic acid preferentially fills the larger internal cavities, which are accessible through microporous windows and are capable of accommodating bulkier molecules. The data further reinforce the notion of internal encapsulation within the hierarchical pore structure of MIL-100(Fe), highlighting successful guest molecule confinement rather than simple surface adsorption. This result is in congruence with the discussion on the loading of syringic acid, having a particle diameter of 7.17 Å, which can fit inside the framework.

Interestingly, a slight increase in the average pore width was observed in SYA@MIL-100(Fe) preparations following the loading of syringic acid. This could be attributed to framework relaxation or partial swelling induced by host–guest interactions between syringic acid and the MIL-100(Fe) structure. Another possibility is the displacement of residual guest molecules or modulators during loading, which freed the previously constricted pore pathways, leading to an increase in the average pore width, as calculated using the BJH method. These results align with the known structural flexibility of MIL-100(Fe) and support the notion that the loading process not only introduces guest molecules but also subtly modifies the internal environment of the host matrix. However, it should be noted that pore volume measurements using nitrogen adsorption may be influenced by the flexibility of the MIL-100(Fe) framework and the presence of residual solvents, potentially affecting the accuracy of the calculated values.

The difference in the degradation temperature of MIL-100(Fe) (~300 °C) and SYA@MIL-100(Fe) (~253–263 °C) suggests that the melting point of syringic acid is at 205–209 °C, but possibly due to the interaction with the framework, the melting point shifted to ~253–263 °C. This is in congruence with the study of Mileo and co-workers [82]. Another key finding from the TGA data was the difference in the residual weights after thermal analysis, which indicates that the 30% residual weight of MIL-100(Fe) accounted for the iron (III) oxide, while a lower residual weight (<30%) for SYA@MIL-100(Fe) indicates a higher organic content, indicative of the presence of syringic acid in the framework [83].

The particle sizes of all MIL-100(Fe) preparations were particularly within the range of nanoparticles (1–1000 nm) [84]. Another key consideration for the use of nanoparticles as drug delivery systems is their ability to enhance solubility, increase bioavailability, and, to a greater extent, pass the blood–brain barrier [84,85]. A smaller particle size is favorable for absorption due to a larger surface area [86]. In addition, MIL-100(Fe), ranging from 100 to 300 nm, is transported through the cell through clathrin or caveolin-mediated endocytosis [87]. The surface morphology of MIL-100(Fe), in conformity with the previous studies, shows that octahedral nanoparticles are easily taken up by the cells through clathrin-mediated endocytosis [88]. Thus, MIL-100(Fe) is widely used as a drug carrier for different drugs [89].

The release of syringic acid in the four media resulted in 5.57% (ultrapure water), 27.57% (PBS pH 7.4), 55.42% (PBS pH 6.8), and 25.80% (0.1 N HCl) after 30 h of release. These data indicate SYA@MIL-100(Fe) preparation was pH-dependent, as greater release in the PBS pH 6.8 media was noted. It can be noted that the release of syringic acid is higher for the PBS pH 7.4 and pH 6.8 and for 0.1 N HCl, compared to distilled water. This is due to the presence of a nucleophilic moiety found on these media, i.e., the phosphate group in PBS media and chloride in diluted hydrochloric acid solution. The presence of these nucleophilic moieties destabilizes the structural integrity of MIL-100(Fe), which would lead to structural integrity and release of syringic acid [45,57,90,91]. Santos and co-workers cited that the presence of chloride ions with MIL-100(Fe) leads to the protonation of the carboxylate group in the trimesic acid, resulting in incomplete reversible structural breakdown, while the presence of the phosphate group leads to competitive complexation with iron, thus displacing trimesic acid complexed with the iron moiety [45]. It is also noteworthy that chloride and phosphate anions are present in the blood, thus providing a mechanism of gradual release inside the body.

The data were fitted to five mathematical models to determine the mechanism of syringic acid release, including the Korsmeyer–Peppas model for water (r^2^ = 0.9788) and 0.1N HCl (r^2^ = 0.9273), the Higuchi model for PBS pH 7.4 (r^2^ = 0.9162), and the first-order release for PBS pH 6.8 (r^2^ = 0.9998). Drug release studies of SYA@MIL-100(Fe) demonstrated that both water (*n* = 0.1796) and simulated gastric fluid (*n* = 0.0824) followed a quasi-Fickian diffusion mechanism, as indicated by their *n*-values below 0.45. This suggests that the release process was primarily governed by passive diffusion through the MOF’s porous structure, with minimal influence from swelling or erosion. Comparable findings have been reported in other MOF-based systems, such as a porous Zn-based MOF, where similarly low *n*-values reflected restricted molecular transport within rigid nanoporous matrices [92]. These results reinforce the potential of MIL-100(Fe) as a viable carrier for controlled diffusion-based drug release under physiological conditions. The Higuchi model describes a drug release mechanism primarily governed by diffusion through a porous matrix. It assumes that the drug is uniformly dispersed within the carrier and that release occurs as the drug diffuses out over time, driven by a concentration gradient. This model is particularly applicable to non-swelling systems like MOFs, where structural stability and pore uniformity enable controlled diffusion-based release. Several MOFs have demonstrated a Higuchi-type release behavior. For instance, the release of ketoprofen from Sr/PTA MOF [93], ibuprofen from UiO-66-NH_2_ [94], and doxorubicin from an MOF [Cu_3_(BTC)_2_] [95] showed sustained release consistent with diffusion through its mesoporous framework. The release kinetics of SYA@MIL-100(Fe) followed a first-order model, which is characterized by a concentration-dependent release rate. In this model, the rate of drug release decreases over time as the concentration of the encapsulated drug within the matrix decreases. This type of kinetic behavior is typical of systems where drug diffusion is the primary release mechanism and no significant burst release occurs. The sustained and controlled release profile provided by the MIL-100(Fe) matrix ensures gradual diffusion of syringic acid into the surrounding medium. Other MOFs that have demonstrated first-order release kinetics include folic acid from CD-MOF@SiO_2_ nanocomposite [96] and ibuprofen and captopril from Zr-MOF [64], both of which similarly showed prolonged concentration-dependent drug release behavior, reinforcing the applicability of this model in MOF-based drug delivery platforms.

The acute oral toxicity result of the syringic acid and SYA@MIL-100(Fe) based on the OECD 423 guidelines at 2000 mg kg^−1^ aligns with the individual safety profile of syringic acid (generally not considered as an acutely toxic agent based on the Safe Work Australia’s Code of Practice; no toxic effect at 14 days 1000 mg kg^−1^ daily administration for 14 days [97]) and MIL-100(Fe) (non-cytotoxic in human normal liver cells (HL-7702) and hepatocellular carcinoma (HepG2) [98]; non-toxic material [99]). It has been noted by Santos and co-workers that the collapse of the MOF with the presence of a more nucleophilic moiety can lead to framework destabilization and collapse, releasing the secondary building unit (SBU) complexed with the nucleophilic moiety, which is generally water-soluble [45].

The results of the serological data from the acute oral toxicity align with previously documented hepatoprotective properties of syringic acid, which include antioxidant activity, inhibition of lipid peroxidation, and modulation of inflammatory responses in the liver. However, the more pronounced reduction observed in our MIL-100(Fe) preparation suggests an added advantage: the sustained and targeted delivery enabled by the MIL-100(Fe) matrix likely enhanced tissue availability and therapeutic retention of syringic acid at hepatocellular sites. Compared to the free form—which is rapidly eliminated and limited by poor solubility—the MIL-100(Fe)-encapsulated syringic acid demonstrated slower systemic clearance and improved bioavailability, as supported by the pharmacokinetic data, particularly significantly higher AUC values of syringic acid in the liver compared to the blood (summarized in Table 2). This prolonged exposure may have allowed for more consistent antioxidant activity within liver tissue, thus minimizing cellular injury and enzyme leakage. The reduced AST and ALT levels in our experimental animals, therefore, not only reaffirm the intrinsic protective effect of syringic acid, as seen in CCl_4_ [100]- and APAP [101]-induced models, thioacetamide-induced hepatic encephalopathy [102], and non-alcoholic fatty liver [103], but also highlight the efficacy of MIL-100(Fe) as a delivery platform in enhancing the hepatoprotective potential of natural phenolic compounds. Overall, this supports the conclusion that the integration of syringic acid into the MIL-100(Fe) framework does not compromise its inherent safety and that the resulting formulation remains biocompatible under controlled conditions. While minimal liver changes, such as mild hepatocellular degeneration, were noted, there were no signs of severe toxicity or irreversible damage. These observations suggest that SYA@MIL-100(Fe) is generally safe for oral administration within the studied dose range. The results are consistent with the existing literature on the parent compound. Syringic acid is widely recognized for its antioxidant and hepatoprotective properties.

Syringic acid is known to exhibit nephroprotective effects by lowering key kidney function markers such as BUN and creatinine, as demonstrated in diabetic nephropathy induced by streptozotocin [104,105], chronic hyperglycemia, renal damage [106], renal oxidative stress, and mitochondrial biogenesis [107]. This outcome supports the formulation’s renal safety and aligns with the antioxidant properties of syringic acid.

The use of MIL-100(Fe) has been noted to increase the AUC_0–72_ values of syringic acid in both the oral (syringic acid: 1419 ± 142.15 mg min mL^−1^ vs. SYA@MIL-100(Fe): 15,606 ± 1936.03 mg min mL^−1^) and intraperitoneal routes (syringic acid: 4368.33 ± 489.25 mg min mL^−1^ vs. SYA@MIL-100(Fe): 56,022.33 ± 2240.13 mg min mL^−1^). Using the formula of the relative bioavailability and syringic acid as the reference, the relative bioavailability (F_rel_) of SYA@MIL-100(Fe) compared to syringic acid was 10.997 (10.997 times greater), while the F_rel_ of the intraperitoneally administered SYA@MIL-100(Fe) compared to syringic acid was 12.82 (12.82 times greater). In comparison, the F_rel_ using the AUC_0–∞_ increased to 89.91 and 38.28 (oral and intraperitoneal administration of SYA@MIL-100(Fe) compared to syringic acid, respectively). These increases in the F_rel_ can be attributed to the use of the MIL-100(Fe) as a carrier. This is noted with a longer elimination half-life estimated using the non-compartment method (syringic acid: 118.77 ± 30.76 min (oral) and 999.64 ± 410 min (IP) versus SYA@MIL-100(Fe): 24,392.75 ± 10,593.37 (oral) and 11,504.68 ± 2306 min (IP)). This extended presence is consistent with the slow and sustained release characteristics of the MOF delivery system. As a result, less frequent dosing may be required to maintain therapeutic levels, reducing the potential for side effects associated with frequent administration. This lower dosing frequency also contributes to improved patient adherence and compliance, which are critical factors in chronic or long-term therapies. Thus, the prolonged half-life not only supports the sustained-release nature of SYA@MIL-100(Fe) but also offers practical clinical advantages in terms of safety, convenience, and therapeutic consistency [108,109,110].

In congruence with the data of the in vitro drug release, the T_max_ for SYA@MIL-100(Fe) (oral: 549.02 ± 159.41 min and IP: 1236.64 ± 91.50 min) is longer compared to the syringic acid (oral: 66.78 ± 7.56 min and IP: 55.97 ± 2.11 min) in both the oral and intraperitoneal routes. The delayed T_max_ reflects the sustained release properties conferred by the MIL-100(Fe) structure, which acts as a reservoir that gradually releases the encapsulated compound into systemic circulation. This behavior is consistent with the intended function of MIL-100(Fe) as a controlled-release drug delivery system. Another key evidence in the use of MIL-100(Fe) is that the C_max_ for both routes of SYA@MIL-100(Fe) (oral: 3.79 ± 0.43 mg mL^−1^ and IP: 26.54 ± 0.55 mg mL^−1^) is greater compared to syringic acid alone (oral —2.33 ± 0.19 mg mL^−1^ and IP—2.55 ± 0.04 mg mL^−1^). This suggests that the MOF effectively protects the active compound during gastrointestinal transit and promotes its gradual release and absorption across biological membranes. In particular, MIL-100(Fe) can activate clathrin-mediated endocytosis. Interestingly, the use of the MIL-100(Fe) as a drug carrier provided a greater AUC_0–72_ of orally administered SYA@MIL-100(Fe) compared to intraperitoneally administered syringic acid, suggesting that the use of MIL-100(Fe) can promote the oral administration of drugs over the intraperitoneal route of administration. The same findings were noted in the study of Santos and co-workers, with an increase in the systemic availability of magnolol using UiO-66(Zr) as a carrier [45]. The F_rel_ of SYA@MIL-100(Fe) is greater than that observed in other research on the development of a drug delivery system for syringic acid, such as TPGS/F127/F68 (2.3-fold) [111], TPGS (2.8-fold) [12], and SMEDDS (2.1-fold) [54].

Both the experimental (0–72) and calculated (0–∞) AUCs are far greater in SYA@MIL-100(Fe) compared to syringic acid alone for the oral as well as the intraperitoneal route of administration in the liver and kidney samples. Greater AUCs were observed in the kidney samples compared to the liver samples, suggesting possible organ-specific accumulation of syringic acid, which may be attributed to active uptake mechanisms or prolonged tissue retention [112]. Such accumulation of the drug in the liver and kidney is consistent with the existing literature on phenolic compounds, which are known to localize preferentially in metabolically active tissues, particularly the liver and kidney, due to their roles in detoxification, metabolism, and excretion processes [113,114,115]. Another potential reason for the increase in the concentration of syringic acid in the liver and kidney after the use of MIL-100(Fe) as a carrier is the internalization of the hepatic phagocytes and renal phagocytes; once inside the cells, the MIL-100(Fe) degrades due to the presence of a nucleophilic moiety, such as phosphate and others [18].

This study’s research design, while suitable for achieving the objectives and consistent with established methodologies, such as those cited in this paper, may limit the generalizability of the findings. The chosen approach was based on feasibility and resource considerations, but future work using alternative or complementary designs could help validate and extend these results.

## 5. Conclusions

This study explores the potential of MIL-100(Fe) as a drug carrier for syringic acid. Experimental data show successful loading of syringic acid into the framework while still maintaining the structural integrity. The slow release of syringic acid from MIL-100(Fe) is noted from the in vitro drug release study, supported by the T_max_ when administered orally and intraperitoneally to the Sprague Dawley rats. The increased AUC_0–72_, AUC_0–∞_, and C_max_ suggest that the systemic availability of syringic acid is increased, coupled with a prolonged elimination half-life. MIL-100(Fe) serves as a new avenue for a drug delivery system for syringic acid and potentially other drugs with low bioavailability.

## 6. Patents

An ongoing patent application is being filed for this study.

## Figures and Tables

**Table 1:** Textural characteristics of the MIL-100(Fe) and SYA@MIL-100(Fe).

	Total Pore Volume (cm^3^ g^−1^) *	Micropore Volume (cm^3^ g^−1^) **	Mesopore Volume (cm^3^ g^−1^) ***	Pore Width (Å) ****
MIL-100(Fe)	0.855916	0.297397	0.558519	29.717
SYA@MIL-100(Fe)—12 h	0.422034	0.231148	0.190886	35.259
SYA@MIL-100(Fe)—24 h	0.619344	0.400067	0.219277	37.399
SYA@MIL-100(Fe)—36 h	0.555394	0.373316	0.182078	35.937
SYA@MIL-100(Fe)—48 h	0.546359	0.339412	0.206947	37.102

* Single-point adsorption total pore volume of pores less than 3873.040 Å width at P/Po = 0.995000000; ** t-plot micropore volume; *** calculated by subtracting the total pore volume from the micropore volume; **** BJH adsorption average pore width (4V/A).

**Table 2:** Summary of the pharmacokinetic parameters for the oral and intraperitoneal route of syringic acid and SYA@MIL-100(Fe).

Test Compound	Biological Sample	Route	AUC_0–72_^*^	AUC_0–∞_ *	C_max_ **	T_max_ ***	T_1/2_ ***
Syringic Acid	Blood	Oral	1419 ± 142.15	1460 ± 143.84	2.33 ± 0.19	66.78 ± 7.56 ^‡^	118.77 ± 30.76
		Intraperitoneal	4368.33 ± 489.25 ^‡^	5400.84 ± 964.81 ^‡^	2.55 ± 0.04 ^‡^	55.97 ± 2.11	999.64 ± 410 ^‡^
	Liver	Oral	32,000.33 ± 3544.16 ^‡^	47,401.91 ± 8515.77 ^‡^	16.28 ± 3.54	515.96 ± 4.44 ^‡^	2288.81 ± 812.66
		Intraperitoneal	102,784.67 ± 1510.75	166,522.15 ± 34,487.20	40.13 ± 0.56 ^‡^	24.70 ±0.21	1852.40 ± 1105.09
	Kidney	Oral	77,103.33 ± 2531.03 ^‡^	78,035.27 ± 2458.81 ^‡^	105.36 ± 9.79	28.21 ± 0.33 ^‡^	37.56 ± 4.69 ^‡^
		Intraperitoneal	321,104.67 ± 11,949.32	808,296.73 ± 429,472.17	218.21 ± 8.73 ^‡^	24.15 ± 0.96	4805.81 ± 3271.08
SYA@MIL-100(Fe)	Blood	Oral	15,606 ± 1936.03	131,269.97 ± 61,666.27	3.79 ± 0.43	549.02 ± 159.41	24,392.75 ± 10,593.37
		Intraperitoneal	56,022.33 ± 2240.13 ^‡^	206,758.55 ± 31,210.34 ^‡^	26.54 ± 0.55 ^‡^	1236.64 ± 91.50 ^‡^	11,504.68 ± 2306 ^‡^
	Liver	Oral	17,309.33 ± 1351.33 ^‡^	22,623.03 ± 2085.19 ^‡^	11.42 ±2.30	385.57 ± 42.44 ^‡^	913.33 ± 223.58
		Intraperitoneal	363,982.67 ± 5429.14	522,988.13 ± 44,624.56	150.25 ± 6.65 ^‡^	111.01 ± 4.91	1309.25 ± 359.93
	Kidney	Oral	130,698 ± 7713.74 ^‡^	144,112.76 ± 8494.44 ^‡^	89.27 ± 17.80	97.30 ± 9.65 ^‡^	265.21 ± 22.01 ^‡^
		Intraperitoneal	1,169,999.33 ± 36,457.71	2,971,634.10 ± 1,163,138.16	393.47 ± 14.44 ^‡^	118.08 ± 0.86	2799.01 ± 1717.60

* Units of area under the curve are mg min mL^−1^ (for blood sample) and μg min g^−1^ (for kidney and liver samples); ** unit of C_max_ is mg mL^−1^; *** unit of T_max_ and T_1/2_ is min; ^‡^ significant differences at *p*-value < 0.05.

## Data Availability

The raw data supporting the conclusions of this article will be made available by the authors on request.

## Supplementary Materials

The following supporting information can be downloaded at: https://www.mdpi.com/article/10.3390/pharmaceutics17101282/s1, supplementary material containing the raw data and analysis plot of the nitrogen sorption isotherm was provided.

## Abbreviations

The following abbreviations are used in this manuscript:

MIL	Materials of Institut Lavoisier
MOF	Metal–Organic Framework
AUC	Area under the Curve
C_max_	Maximum Concentration
T_max_	Time to Reach Maximum Concentration
T_1/2_	Half-life
SD	Sprague Dawley
SYA@MIL-100(Fe)	Syringic Acid-Loaded MIL-100(Fe)
PXRD	Powder X-Ray Diffraction
FTIR	Fourier Transform Infrared Spectroscopy
h	Hour
min	Minute
s	Second
mL	Milliliter
Mcg	Microgram
