# Supplementary material for: MIL-100(Fe)-Enabled Oral Delivery of Syringic Acid with Enhanced Pharmacokinetics
Source: Pharmaceutics. 2025 Oct 1;17(10):1282. doi: 10.3390/pharmaceutics17101282 (PMC12567248; doi:10.3390/pharmaceutics17101282)
Supplement: Supplementary file 1 [file pharmaceutics-17-01282-s001.zip › pharmaceutics-3791310-supplementary.pdf]

pharmaceutics

MDPI

MIL-100(Fe)-Enabled Oral Delivery of Syringic Acid with Enhanced Pharmacokinetics

Joshua Santos[1,2]*, Hannah Jean Victoriano[2], Mary Sepulveda[2], Hung-En Liu[3], Shierrie Mae N. Valencia[2], Rikkamae Zinca Marie L. Walde[2], Emelda A. Ongo[1,2], Chia-Her Lin[4]

Nitrogen Sorption Isotherms

The data obtained from Micromeritics (MicroActive for ASAP 2020 6.01). Samples degassed at 150°C for 48 hours, then 77K for Nitrogen Adsorption.

Raw data for MIL-100(Fe)

Summary Report Surface Area

Single point surface area at P/Po = 0.249552124: 1,677.4791 m²/g

BET Surface Area: 2,028.3473 m²/g Langmuir Surface Area: 2,536.761 m²/g

t-Plot Micropore Area: 912.9137 m²/g

t-Plot external surface area: 1,115.4336 m²/g

BJH Adsorption cumulative surface area of pores

between 17.000 Å and 3,000.000 Å width: 235.8991 m²/g

BJH Desorption cumulative surface area of pores
between 17.000 Å and 3,000.000 Å width: 196.8770 m²/g

Pore Volume

Single point adsorption total pore volume of pores
less than 3,873.040 Å width at P/Po = 0.995000000: 0.855916 cm³/g

Single point desorption total pore volume of pores
less than 3,873.040 Å width at P/Po = 0.995000000: 0.863617 cm³/g

t-Plot micropore volume:

0.297397 cm³/g BJH Adsorption cumulative volume

of pores
between 17.000 Å and 3,000.000 Å width: 0.175253 cm³/g

BJH Desorption cumulative volume of pores
between 17.000 Å and 3,000.000 Å width: 0.168875 cm³/g

Pore Size

Adsorption average pore diameter (4V/A by BET): 16.879 Å Desorption average pore diameter

(4V/A by BET): 17.03

Pore Size

BJH Adsorption average pore width (4V/A):

29.717 Å BJH Desorption average pore

pharmaceutics

MDPI

width (4V/A): 34.311 Å

Horvath-Kawazoe

Maximum pore volume at P/Po = 0.100099740: 0.693162 cm³/g

Median pore width: 15.368 Å

Isotherm Tabular Report

Relative Pressure (P/Po)	Absolute Pressure (mmHg)	Quantity Adsorbed (cm³/g STP)	Elapsed Time (h:min)	Saturation Pressure (mmHg)
			03:42	760.643921
0.000001338	0.001018	8.1019	04:35	
0.000002165	0.001647	16.2014	05:27	
0.000004633	0.003523	24.2925	06:20	
0.000009575	0.007281	32.3643	07:07	
0.000016515	0.012558	40.4105	07:51	
0.000024975	0.018990	48.4272	08:33	
0.000034851	0.026498	56.4286	09:11	
0.000046310	0.035209	64.4213	09:46	
0.000059922	0.045556	72.4146	10:23	
0.000076280	0.057990	80.4181	10:55	
0.000096347	0.073242	88.5047	11:20	
0.000120513	0.091611	96.5861	11:44	
0.000149407	0.113572	104.6618	12:08	
0.000183357	0.139374	112.7323	12:32	
0.000223609	0.169966	120.7977	12:54	
0.000270570	0.205655	128.8591	13:16	
0.000325035	0.247046	136.9485	13:39	
0.000387728	0.294688	144.9996	14:02	
0.000460016	0.349619	153.0418	14:24	
0.000542792	0.412518	161.0782	14:47	
0.000638270	0.485067	169.1035	15:09	
0.000747327	0.567930	177.1193	15:32	
0.000873026	0.663437	185.1199	15:54	
0.001017166	0.772951	193.1052	16:16	
0.001185251	0.900654	201.0656	16:38	
0.001380596	1.049064	209.0008	17:00	
0.001608409	1.222136	216.9016	17:22	
0.001878364	1.427219	224.7534	17:44	
0.002199330	1.671045	232.5449	18:07	
0.002584359	1.963539	240.2531	18:27	
0.003054696	2.320828	247.9809	18:48	
0.003625452	2.754385	255.6243	19:10	
0.004320907	3.282668	263.1058	19:29	
0.005157876	3.918426	270.3543	19:49	
0.006182077	4.696388	277.4538	20:09	

pharmaceutics

MDPI

Isotherm Tabular Report

Relative Pressure (P/Po)	Absolute Pressure (mmHg)	Quantity Adsorbed (cm³/g STP)	Elapsed Time (h:min)	Saturation Pressure (mmHg)
			20:14	759.673218
0.007455339	5.663941	283.8144	20:27	
0.008954308	6.803174	290.6052	20:42	
0.010703736	8.132682	297.0715	20:52	
0.030260686	22.993618	340.5778	21:08	
0.060990218	46.347710	406.8620	21:29	
0.085021355	64.613663	433.9851	21:44	
0.100099740	76.076408	448.1264	21:55	
0.117405081	89.233215	469.1824	22:07	
0.135017688	102.626274	495.3306	22:22	
			22:24	760.101624
0.164621874	125.132080	504.1176	22:31	
0.187882777	142.815338	507.6461	22:36	
0.205982011	156.575058	509.8880	22:40	
0.250314610	190.276978	514.0809	22:45	
0.301809172	229.423492	517.6091	22:49	
0.352256146	267.773804	520.1516	22:52	
0.399912230	304.004211	522.0140	22:56	
0.450022577	342.099091	523.5662	22:58	
0.500095014	380.166809	524.8322	23:01	
0.550236850	418.287994	525.7666	23:04	
0.599996362	456.117828	526.5860	23:06	
0.650125967	494.231018	527.2673	23:09	
0.700031570	532.172974	527.8721	23:11	
0.750134533	570.267212	528.4744	23:14	
0.799996186	608.176819	529.1949	23:16	
0.820052966	623.430298	529.7130	23:19	
0.850330829	646.452515	530.3252	23:21	
0.874684004	664.970825	531.0439	23:23	
0.900059535	684.268738	531.9831	23:26	
0.924844585	703.115906	533.2591	23:28	
0.949736437	722.046753	535.1919	23:31	
0.974245606	740.687012	539.2448	23:34	
0.980406383	745.375488	541.5653	23:36	
0.990367839	752.955933	546.4965	23:39	
0.994377647	756.013916	550.9975	23:43	

Isotherm Tabular Report

Relative Pressure (P/Po)	Absolute Pressure (mmHg)	Quantity Adsorbed (cm³/g STP)	Elapsed Time (h:min)	Saturation Pressure (mmHg)
0.996619010	757.741577	559.4560	23:53	
0.978317217	743.838074	546.6718	23:58	
0.960027810	729.941284	539.8266	24:02	
0.933479485	709.762329	535.6354	24:05	
0.907302274	689.865173	533.4891	24:08	
0.881831960	670.503052	532.2604	24:10	
0.856664989	651.371338	531.4069	24:12	
0.831343309	632.123718	530.7315	24:15	
0.806290465	613.078247	530.2070	24:17	
0.781254469	594.045349	529.7926	24:19	
0.749938577	570.238892	529.3437	24:22	
0.699940478	532.224609	528.7903	24:24	
			24:26	760.390259
0.650125692	494.349243	528.1379	24:29	
0.600213762	456.396698	527.4659	24:32	
0.550270889	418.420624	526.6955	24:34	
0.499802299	380.044800	525.6666	24:37	
0.449784014	342.011383	524.3134	24:40	
0.399583480	303.839386	522.7075	24:43	
0.350046323	266.171814	520.7036	24:46	
0.300004158	228.120239	518.0940	24:50	
0.250148155	190.210220	514.6170	24:54	
0.200308808	152.312866	509.7542	25:00	
0.141825900	107.843033	499.4481	25:10	

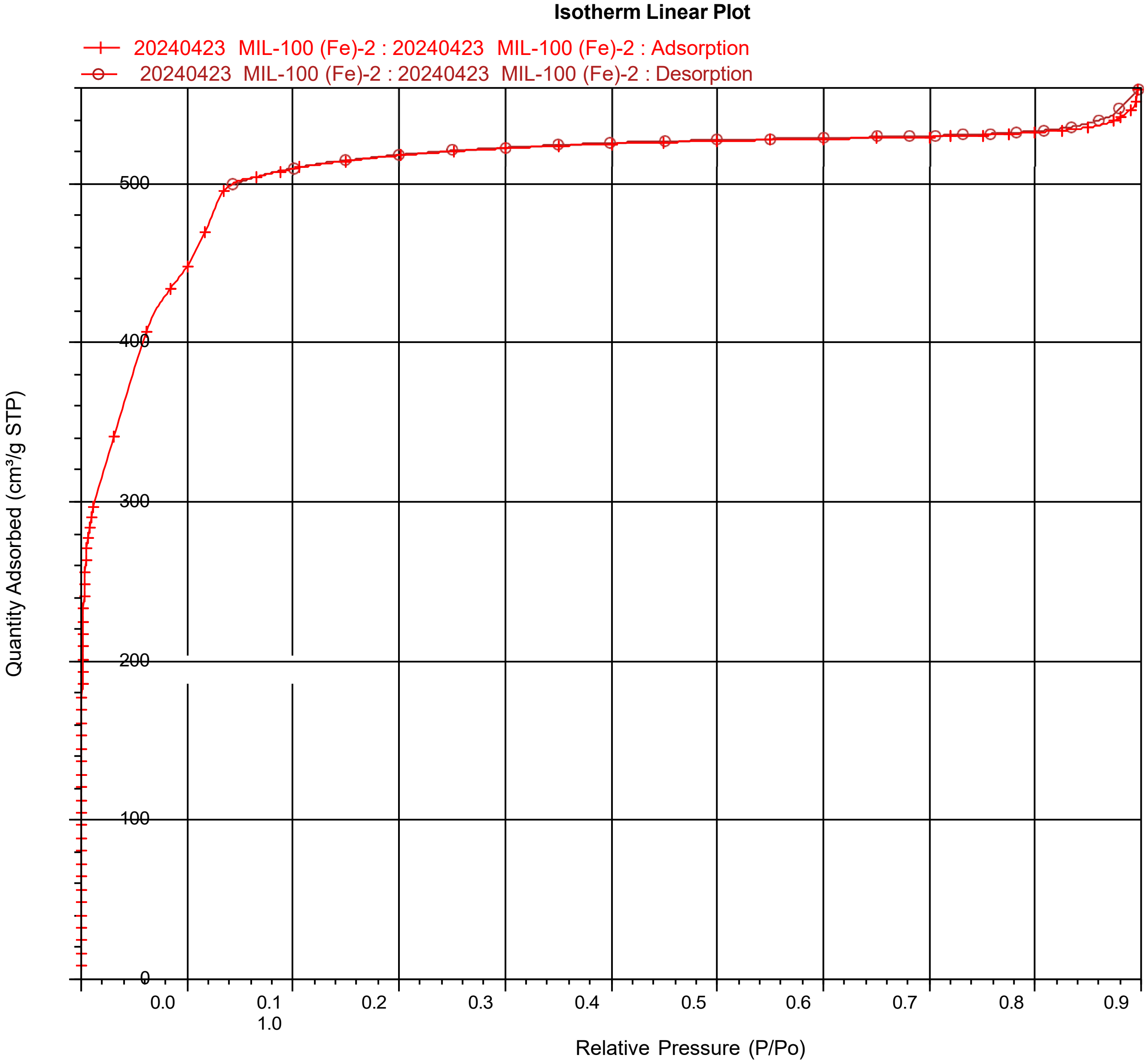
Isotherm Linear Plot
20240423 MIL-100 (Fe)-2 : 20240423 MIL-100 (Fe)-2 : Adsorption
20240423 MIL-100 (Fe)-2 : 20240423 MIL-100 (Fe)-2 : Desorption
Quantity Adsorbed (cm³/g STP)
500
400
300
200
100
0
0.0
0.1
1.0
0.2
0.3
0.4
0.5
0.6
0.7
0.8
0.9
Relative Pressure (P/Po)

pharmaceutics

MDPI

BET Report

BET surface area: 2028.3473 ± 57.4186 m²/g Slope: 0.002112 ± 0.000060 g/cm³ STP
Y-intercept: 0.000034 ± 0.000006 g/cm³ STP C: 64.042250
Qm: 466.0105 cm³/g STP Correlation coefficient: 0.9987748
Molecular cross-sectional area: 0.1620 nm²

Relative Pressure (P/Po)	Quantity Adsorbed (cm³/g STP)	1/[Q(Po/P - 1)]
0.060990218	406.8620	0.000160
0.085021355	433.9851	0.000214
0.100099740	448.1264	0.000248
0.117405081	469.1824	0.000284
0.135017688	495.3306	0.000315

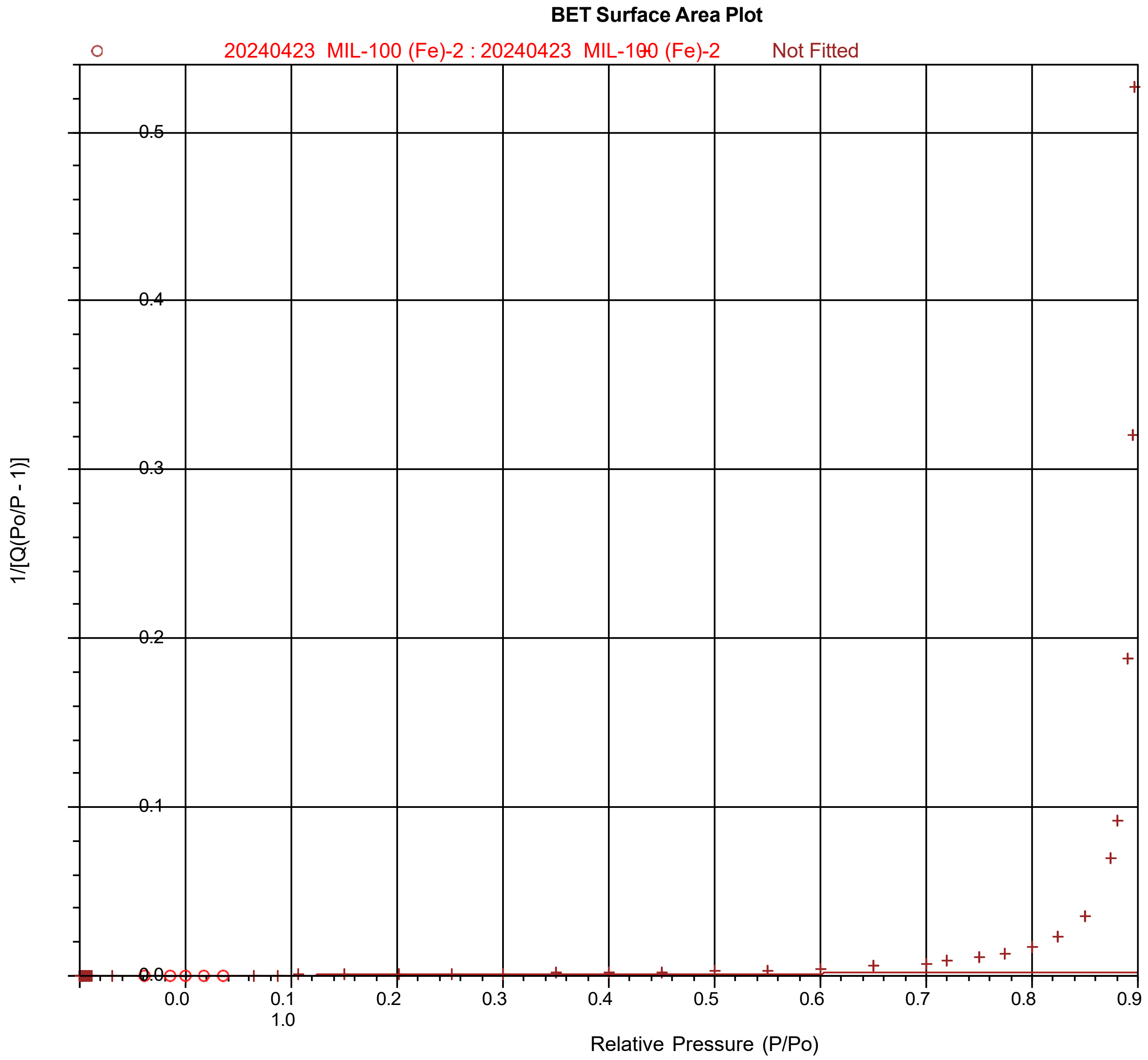
BET Surface Area Plot
20240423 MIL-100 (Fe)-2 : 20240423 MIL-100 (Fe)-2
Not Fitted
1/[Q(Po/P - 1)]
0.0
0.1
0.2
0.3
0.4
0.5
0.0
0.1
0.2
0.3
0.4
0.5
0.6
0.7
0.8
0.9
1.0
Relative Pressure (P/Po)

pharmaceutics

MDPI

t-Plot Report

Micropore volume:
0.297397 cm³/g
Micropore area:
912.9137 m²/g
External surface area: 1115.4336 m²/g
Slope: 72.112337 ± 13.680458 cm³/g·Å STP
Y-intercept: 192.265842 ± 55.811848 cm³/g
STP Correlation coefficient: 0.906868
Surface area correction factor:
1.000 Density conversion
factor: 0.0015468
Total surface area (BET): 2028.3473
m²/g Thickness range: 3.5000
Å to 5.0000 Å
Thickness equation: Harkins and Jura

Thickness Curve

t = [13.99 / (0.034 - log(P/Po))] ^ 0.5

t-Plot Report - Data

Relative Pressure (P/Po)	Statistical Thickness (Å)	Quantity Adsorbed (cm³/g STP)	Fitted
0.000001338	1.5389	8.1019	
0.000002165	1.5668	16.2014	
0.000004633	1.6143	24.2925	
0.000009575	1.6640	32.3643	
0.000016515	1.7044	40.4105	
0.000024975	1.7371	48.4272	
0.000034851	1.7648	56.4286	
0.000046310	1.7896	64.4213	
0.000059922	1.8130	72.4146	
0.000076280	1.8357	80.4181	
0.000096347	1.8585	88.5047	
0.000120513	1.8813	96.5861	
0.000149407	1.9039	104.6618	
0.000183357	1.9262	112.7323	
0.000223609	1.9486	120.7977	
0.000270570	1.9709	128.8591	
0.000325035	1.9930	136.9485	
0.000387728	2.0150	144.9996	
0.000460016	2.0371	153.0418	
0.000542792	2.0592	161.0782	

pharmaceutics MDPI

t-Plot Report - Data

Relative Pressure (P/Po)	Statistical Thickness (Å)	Quantity Adsorbed (cm³/g STP)	Fitted
0.000638270	2.0815	169.1035	
0.000747327	2.1039	177.1193	
0.000873026	2.1268	185.1199	
0.001017166	2.1500	193.1052	
0.001185251	2.1739	201.0656	
0.001380596	2.1987	209.0008	
0.001608409	2.2243	216.9016	
0.001878364	2.2513	224.7534	
0.002199330	2.2798	232.5449	
0.002584359	2.3101	240.2531	
0.003054696	2.3427	247.9809	
0.003625452	2.3777	255.6243	
0.004320907	2.4152	263.1058	
0.005157876	2.4548	270.3543	
0.006182077	2.4975	277.4538	
0.007455339	2.5441	283.8144	
0.008954308	2.5922	290.6052	
0.010703736	2.6419	297.0715	
0.030260686	3.0013	340.5778	
0.060990218	3.3471	406.8620	
0.085021355	3.5590	433.9851	*
0.100099740	3.6791	448.1264	*
0.117405081	3.8089	469.1824	*
0.135017688	3.9348	495.3306	*
0.164621874	4.1368	504.1176	*
0.187882777	4.2901	507.6461	*
0.205982011	4.4075	509.8880	*
0.250314610	4.6919	514.0809	*
0.301809172	5.0240	517.6091	
0.352256146	5.3590	520.1516	
0.399912230	5.6905	522.0140	
0.450022577	6.0615	523.5662	
0.500095014	6.4628	524.8322	
0.550236850	6.9046	525.7666	
0.599996362	7.3946	526.5860	
0.650125967	7.9563	527.2673	

pharmaceutics

MDPI

t-Plot Report - Data

Relative Pressure (P/Po)	Statistical Thickness (Å)	Quantity Adsorbed (cm³/g STP)	Fitted
0.700031570	8.6062	527.8721	

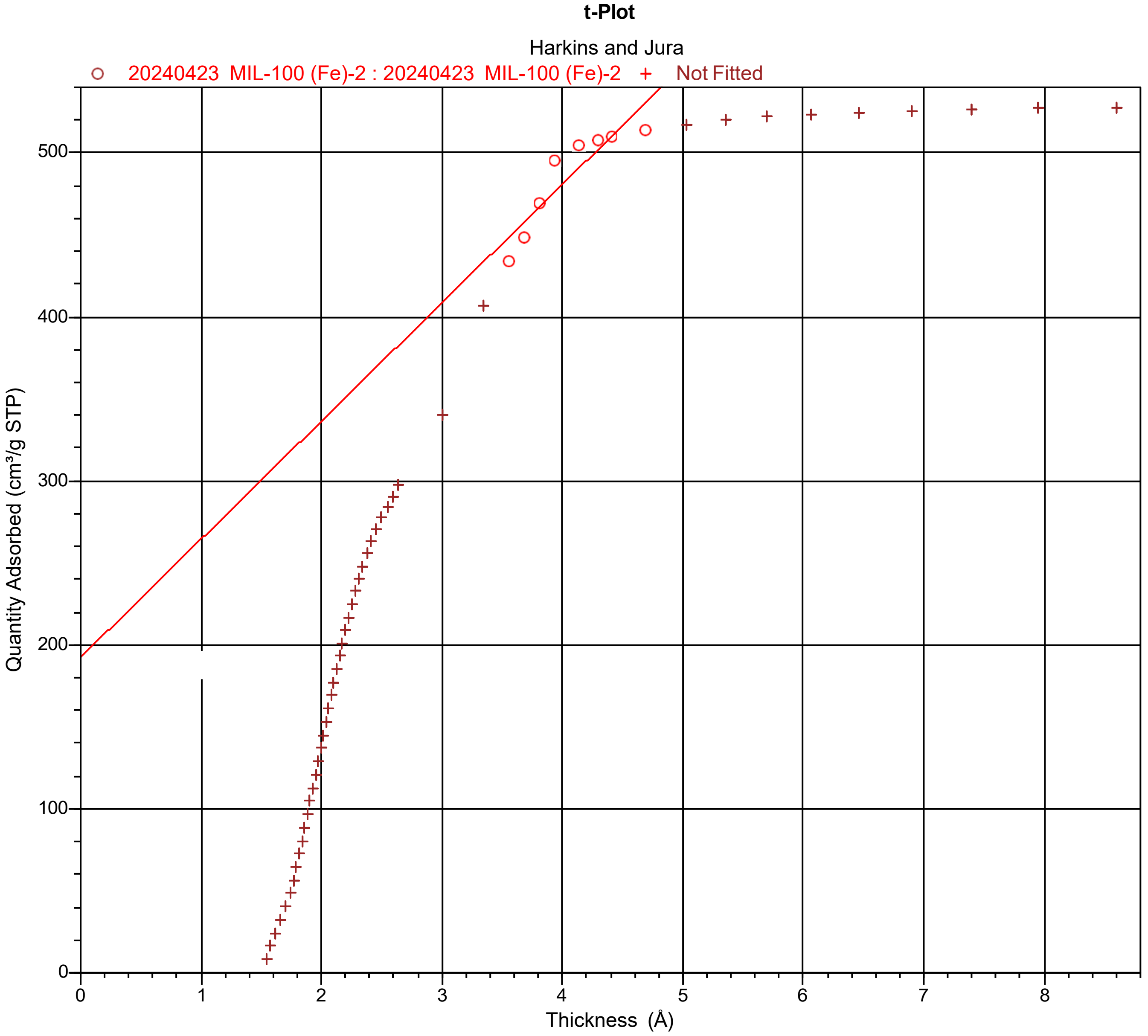
t-Plot
Harkins and Jura
20240423 MIL-100 (Fe)-2 : 20240423 MIL-100 (Fe)-2
Not Fitted
Quantity Adsorbed (cm³/g STP)
Thickness (Å)
0
100
200
300
400
500
0
1
2
3
4
5
6
7
8

pharmaceutics

MDPI

BJH Adsorption Pore Distribution Report

Faas Correction

Halsey

t = 3.54 [-5 / ln(P/Po)] ^ 0.333

Width range: 17.000 Å to 3,000.000 Å

Adsorbate property factor: 9.53000 Å

Density conversion factor: 0.0015468

Fraction of pores open at both ends: 0.00

Pore Width Range (Å)	Average Width (Å)	Incremental Pore Volume (cm³/g)	Cumulative Pore Volume (cm³/g)	Incremental Pore Area (m²/g)	Cumulative Pore Area (m²/g)
3448.4 - 2025.9	2386.7	0.007228	0.007228	0.121	0.121
2025.9 - 1007.9	1204.9	0.008064	0.015292	0.268	0.389
1007.9 - 771.2	857.7	0.003846	0.019138	0.179	0.568
771.2 - 402.1	477.6	0.006892	0.026030	0.577	1.145
402.1 - 272.2	311.8	0.003305	0.029334	0.424	1.569
272.2 - 206.6	230.0	0.002225	0.031559	0.387	1.956
206.6 - 166.0	181.6	0.001664	0.033223	0.367	2.323
166.0 - 139.7	150.4	0.001298	0.034521	0.345	2.668
139.7 - 116.8	126.0	0.001088	0.035609	0.345	3.013
116.8 - 105.4	110.4	0.001021	0.036630	0.370	3.383
105.4 - 84.6	92.4	0.001329	0.037959	0.575	3.958
84.6 - 70.5	76.1	0.001156	0.039115	0.608	4.566
70.5 - 60.3	64.5	0.001270	0.040384	0.788	5.354
60.3 - 52.4	55.7	0.001582	0.041967	1.136	6.490
52.4 - 46.3	48.9	0.002102	0.044068	1.720	8.209
46.3 - 41.2	43.4	0.002567	0.046636	2.369	10.578
41.2 - 36.9	38.8	0.003827	0.050463	3.951	14.529
36.9 - 33.3	34.8	0.005007	0.055470	5.748	20.277
33.3 - 30.2	31.5	0.006412	0.061881	8.133	28.410
30.2 - 27.3	28.6	0.009353	0.071235	13.101	41.511
27.3 - 24.6	25.8	0.013964	0.085199	21.671	63.182
24.6 - 22.5	23.4	0.017795	0.102994	30.420	93.602
22.5 - 21.6	22.0	0.010024	0.113018	18.223	111.825
21.6 - 20.5	21.0	0.016418	0.129435	31.254	143.080
20.5 - 19.1	19.7	0.045817	0.175253	92.819	235.899

pharmaceutics

MDPI

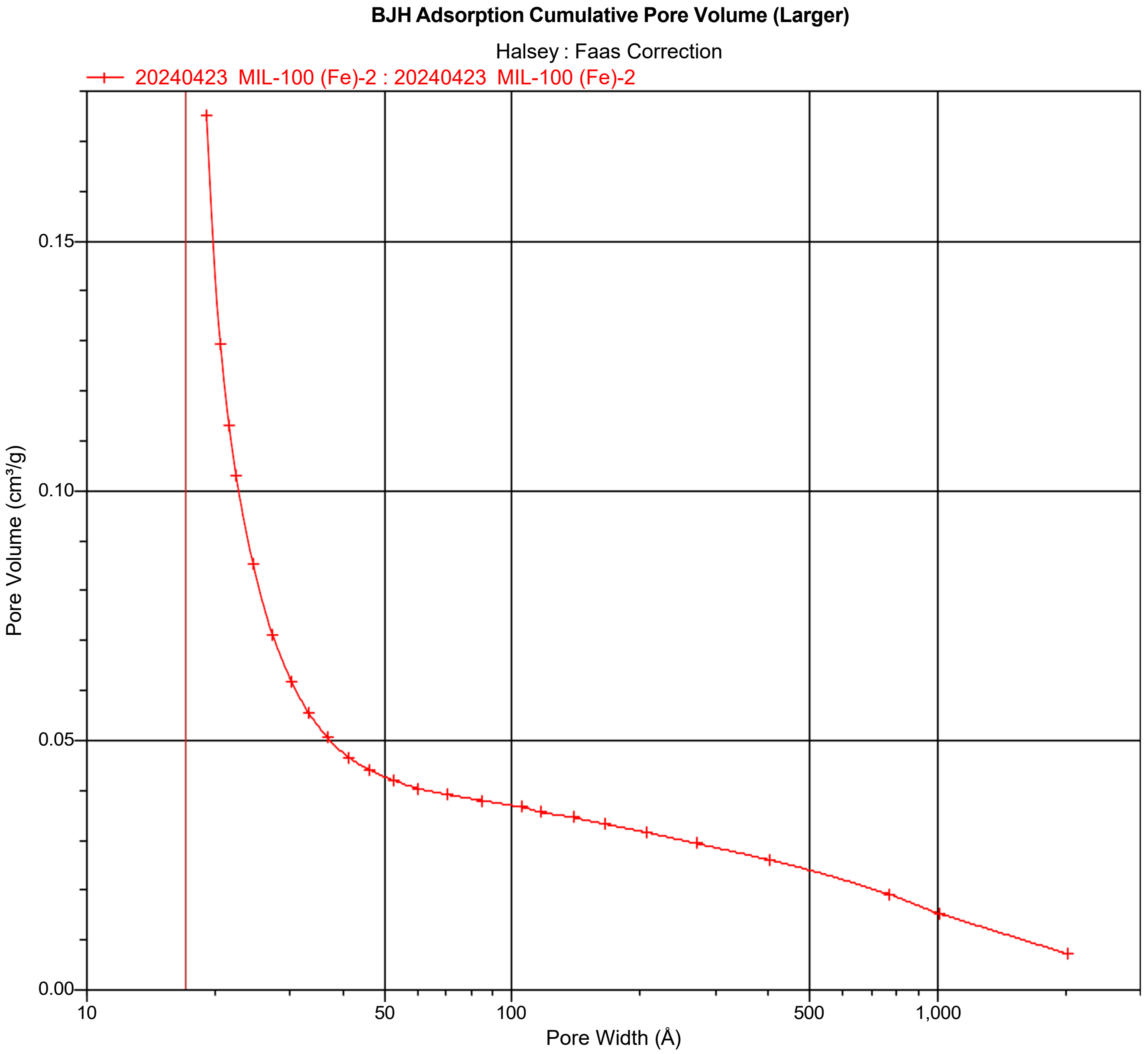

pharmaceutics

MDPI

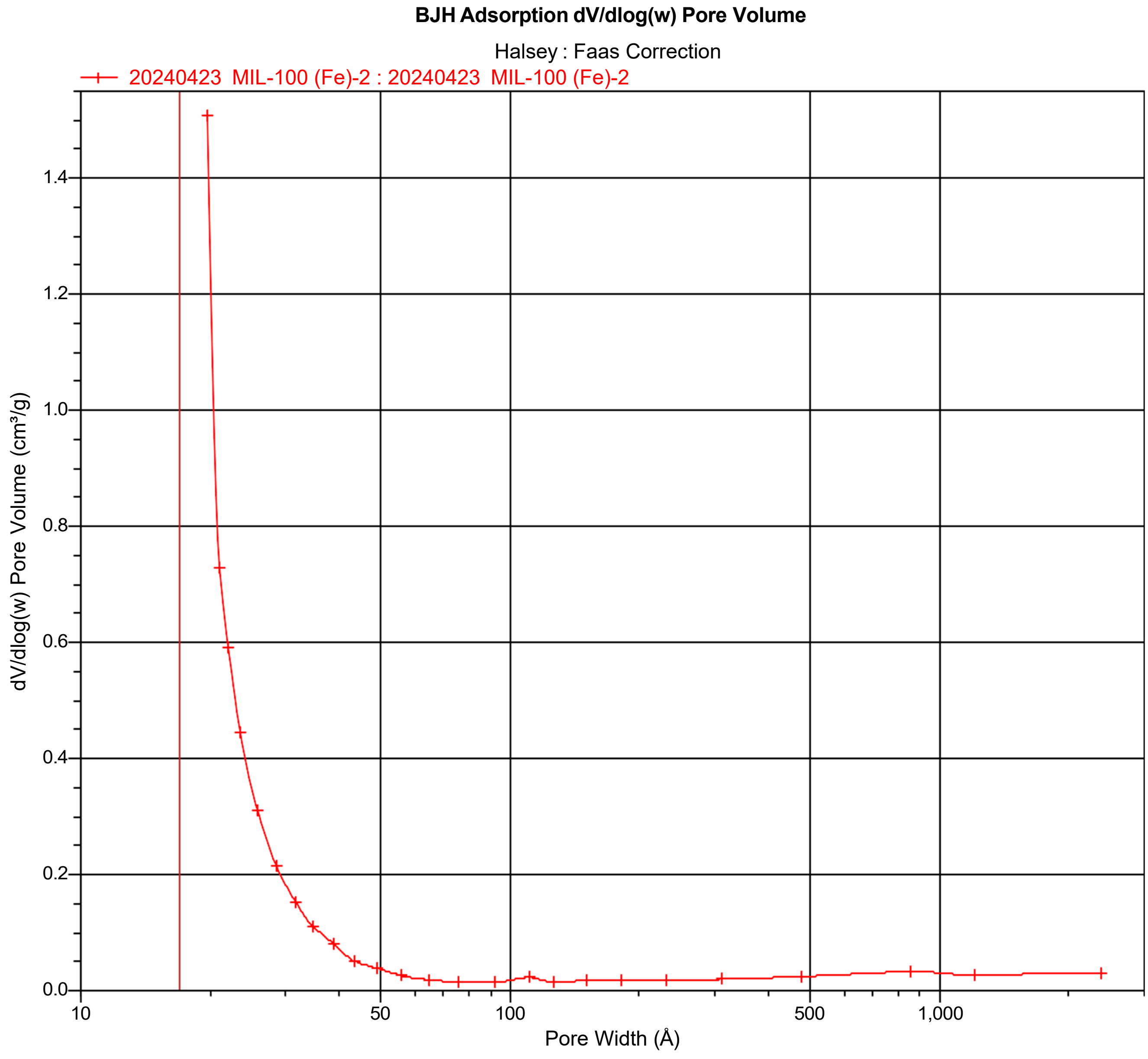

pharmaceutics

MDPI

BJH Desorption Pore Distribution Report

Faas Correction
Halsey
t = 3.54 [-5 / ln(P/Po)] ^ 0.333

Width range: 17.000 Å to 3,000.000 Å
Adsorbate property factor: 9.53000 Å
Density conversion factor: 0.0015468
Fraction of pores open at both ends: 0.00

Pore Width Range (Å)	Average Width (Å)	Incremental Pore Volume (cm³/g)	Cumulative Pore Volume (cm³/g)	Incremental Pore Area (m²/g)	Cumulative Pore Area (m²/g)
5708.3 - 912.7	1025.3	0.021552	0.021552	0.841	0.841
912.7 - 502.3	594.6	0.011590	0.033142	0.780	1.620
502.3 - 306.4	357.5	0.007184	0.040326	0.804	2.424
306.4 - 222.2	250.4	0.003683	0.044009	0.588	3.013
222.2 - 175.7	193.2	0.002082	0.046092	0.431	3.444
175.7 - 145.7	157.7	0.001443	0.047534	0.366	3.810
145.7 - 124.4	133.2	0.001153	0.048687	0.346	4.156
124.4 - 108.7	115.4	0.000891	0.049579	0.309	4.465
108.7 - 96.5	101.8	0.000693	0.050271	0.272	4.737
96.5 - 84.6	89.6	0.000765	0.051037	0.342	5.079
84.6 - 70.5	76.0	0.000931	0.051968	0.490	5.569
70.5 - 60.3	64.5	0.001321	0.053289	0.820	6.389
60.3 - 52.5	55.7	0.001482	0.054772	1.064	7.453
52.5 - 46.3	48.9	0.001882	0.056654	1.540	8.992
46.3 - 41.2	43.3	0.002824	0.059478	2.606	11.598
41.2 - 36.9	38.7	0.004074	0.063552	4.207	15.806
36.9 - 33.2	34.8	0.005135	0.068686	5.899	21.704
33.2 - 30.1	31.4	0.006865	0.075551	8.733	30.438
30.1 - 27.2	28.4	0.009571	0.085121	13.458	43.896
27.2 - 24.6	25.7	0.013695	0.098816	21.288	65.184
24.6 - 22.2	23.2	0.020667	0.119484	35.596	100.780
22.2 - 19.4	20.6	0.049392	0.168875	96.097	196.877

pharmaceutics

MDPI

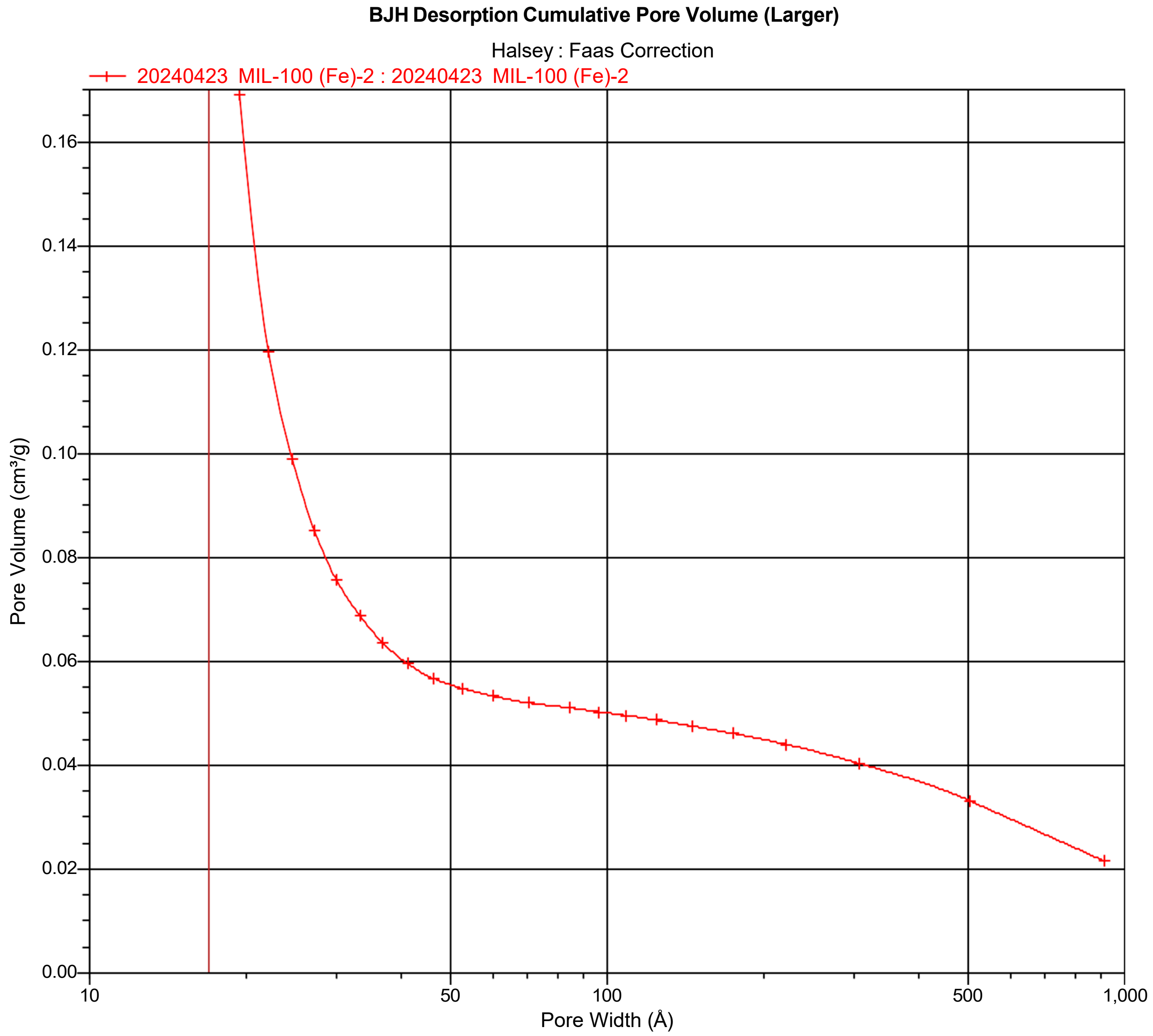

pharmaceutics

MDPI

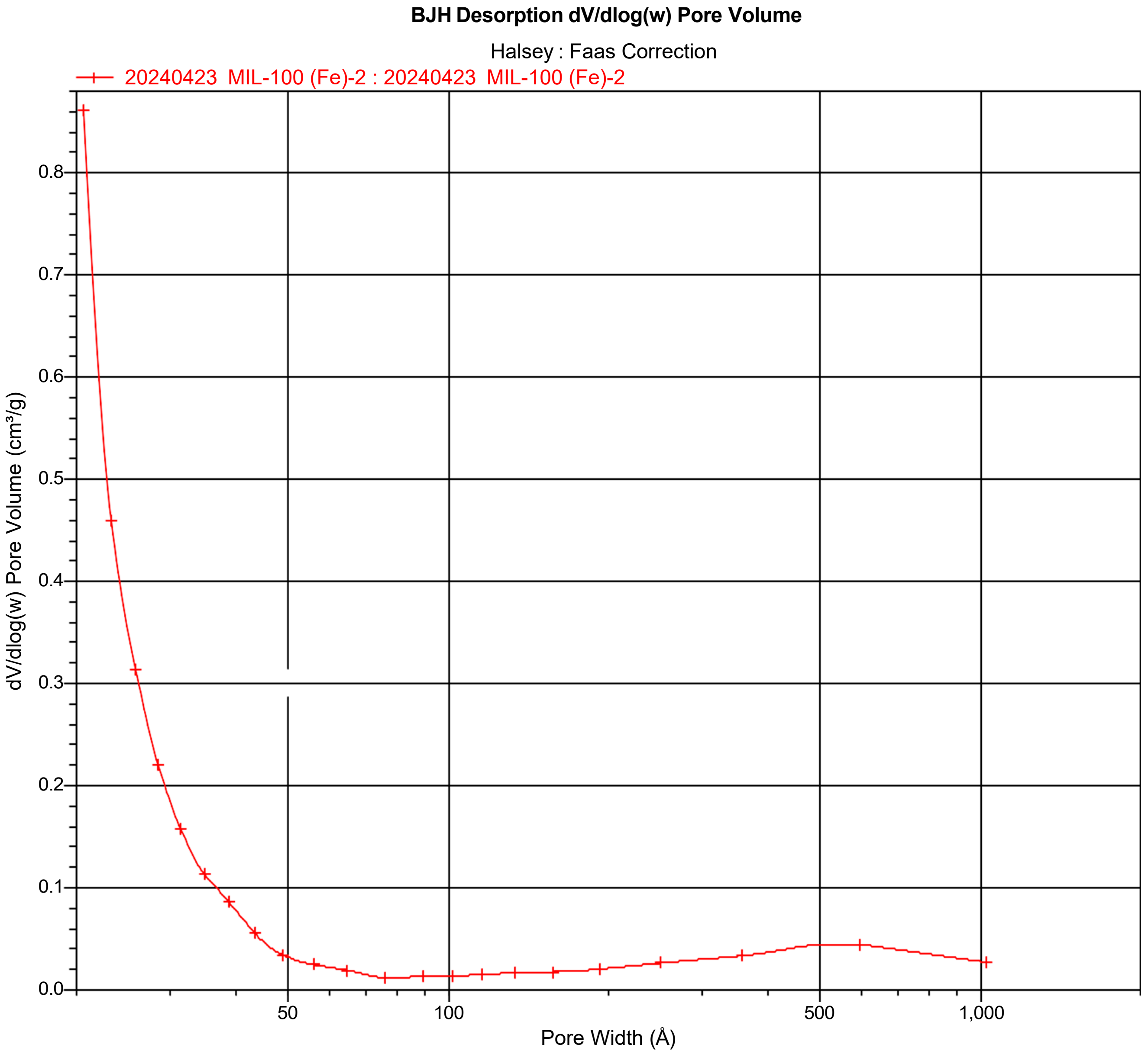

pharmaceutics

MDPI

Porosity Distribution by
Model: N2 - Tarazona NLDFT, Esf = 30.0K
Method: Non-negative Regularization: 0.01000
Standard Deviation of Fit: 5.97618 cm³/g STP

Volume in Pores	<	4.86 Å	:	0.00215 cm³/g
Total Volume in Pores	<=	448.83 Å	:	0.84900 cm³/g
Total Area in Pores	>=	4.86 Å	:	3,158.341 m²/g

Pore Size Table

Pore Width (Å)	Cumulative Volume (cm³/g)	Incremental Volume (cm³/g)	Cumulative Area (m²/g)	Incremental Area (m²/g)
4.86	0.01004	0.00789	64.919	64.919
5.22	0.02749	0.01745	198.660	133.741
5.93	0.05874	0.03125	409.340	210.680
6.29	0.10130	0.04256	679.926	270.586
6.65	0.15019	0.04889	974.049	294.123
7.36	0.20091	0.05072	1249.554	275.505
7.72	0.24474	0.04383	1476.628	227.074
8.44	0.28311	0.03837	1658.551	181.923
8.79	0.32196	0.03886	1835.303	176.752
9.51	0.36973	0.04777	2036.254	200.951
10.22	0.42151	0.05178	2238.856	202.602
10.94	0.46410	0.04259	2394.599	155.743
11.65	0.49026	0.02615	2484.377	89.778
12.37	0.49344	0.00318	2494.661	10.284
13.08	0.49344	0.00000	2494.661	0.000
13.80	0.49344	0.00000	2494.661	0.000
14.87	0.49344	0.00000	2494.661	0.000
15.59	0.49344	0.00000	2494.661	0.000
16.66	0.49344	0.00000	2494.661	0.000
17.73	0.51892	0.02548	2552.145	57.484
18.44	0.57845	0.05953	2681.240	129.095
19.87	0.63595	0.05750	2796.973	115.733
20.95	0.68283	0.04688	2886.497	89.524
22.02	0.73508	0.05225	2981.413	94.916
23.45	0.79689	0.06181	3086.847	105.434
24.52	0.83304	0.03614	3145.803	58.956
25.95	0.84069	0.00765	3157.600	11.797

pharmaceutics

MDPI

Sample: 20240423 MIL-100 (Fe)-2
Operator:
Submitter: Micromeritics
File: C:\MicroActive for ASA...\20240423 MIL-100 (Fe)-2.SMP

Started: 4/23/2024 7:59:48 PM
Completed: 4/24/2024 9:36:46 PM
Report time: 5/3/2024 12:50:11 PM
Sample mass: 0.0750 g
Cold free space: 84.0611 cm³
Low pressure dose: 8.0000 cm³/g STP
Automatic degas: No

Analysis adsorptive: N2
Analysis bath temp.: -195.796 °C
Thermal correction: No
Warm free space: 26.9173 cm³ Measured
Equilibration interval: 20 s
Sample density: 1.000 g/cm³

Comments: Use sample tube with isothermal jacket and filler rod. Follow the instructions on the Silica-Alumina sample data sheet for sample preparation and special instructions. Be sure to use specifications for the current lot.

Pore Size Table

Pore Width (Å)	Cumulative Volume (cm³/g)	Incremental Volume (cm³/g)	Cumulative Area (m²/g)	Incremental Area (m²/g)
27.38	0.84069	0.00000	3157.600	0.000
29.17	0.84069	0.00000	3157.600	0.000
30.60	0.84069	0.00000	3157.600	0.000
32.39	0.84069	0.00000	3157.600	0.000
34.17	0.84069	0.00000	3157.600	0.000
35.96	0.84069	0.00000	3157.600	0.000
38.11	0.84069	0.00000	3157.600	0.000
40.25	0.84069	0.00000	3157.600	0.000
42.39	0.84069	0.00000	3157.600	0.000
44.54	0.84069	0.00000	3157.600	0.000
47.04	0.84069	0.00000	3157.600	0.000
49.54	0.84069	0.00000	3157.600	0.000
52.05	0.84069	0.00000	3157.600	0.000
54.91	0.84069	0.00000	3157.600	0.000
57.77	0.84069	0.00000	3157.600	0.000
60.98	0.84069	0.00000	3157.600	0.000
64.20	0.84069	0.00000	3157.600	0.000
67.42	0.84069	0.00000	3157.600	0.000
70.99	0.84069	0.00000	3157.600	0.000
74.57	0.84069	0.00000	3157.600	0.000
78.50	0.84069	0.00000	3157.600	0.000
82.79	0.84069	0.00000	3157.600	0.000
87.08	0.84069	0.00000	3157.600	0.000
91.37	0.84069	0.00000	3157.600	0.000
96.37	0.84069	0.00000	3157.600	0.000
101.38	0.84069	0.00000	3157.600	0.000
106.38	0.84069	0.00000	3157.600	0.000
112.10	0.84069	0.00000	3157.600	0.000
117.82	0.84069	0.00000	3157.600	0.000
123.90	0.84069	0.00000	3157.600	0.000
130.33	0.84069	0.00000	3157.600	0.000
136.76	0.84069	0.00000	3157.600	0.000
143.91	0.84069	0.00000	3157.600	0.000
151.06	0.84069	0.00000	3157.600	0.000
158.93	0.84069	0.00000	3157.600	0.000
167.15	0.84069	0.00000	3157.600	0.000

pharmaceutics

Pore Size Table

Pore Width (Å)	Cumulative Volume (cm³/g)	Incremental Volume (cm³/g)	Cumulative Area (m²/g)	Incremental Area (m²/g)
175.73	0.84069	0.00000	3157.600	0.000
184.66	0.84069	0.00000	3157.600	0.000
193.96	0.84069	0.00000	3157.600	0.000
203.97	0.84069	0.00000	3157.600	0.000
214.33	0.84069	0.00000	3157.600	0.000
225.06	0.84069	0.00000	3157.600	0.000
236.50	0.84069	0.00000	3157.600	0.000
248.29	0.84069	0.00000	3157.600	0.000
261.16	0.84069	0.00000	3157.600	0.000
274.39	0.84069	0.00000	3157.600	0.000
287.97	0.84069	0.00000	3157.600	0.000
302.63	0.84069	0.00000	3157.600	0.000
318.00	0.84069	0.00000	3157.600	0.000
334.08	0.84069	0.00000	3157.600	0.000
350.88	0.84069	0.00000	3157.600	0.000
368.76	0.84069	0.00000	3157.600	0.000
387.34	0.84069	0.00000	3157.600	0.000
406.65	0.84069	0.00000	3157.600	0.000
427.38	0.84069	0.00000	3157.600	0.000
448.83	0.84900	0.00831	3158.341	0.741

pharmaceutics

MDPI

Porosity Distribution by
Model: N2 - Tarazona NLDFT, Esf = 30.0K
Method: Non-negative Regularization: 0.01000

Standard Deviation of Fit: 5.97618 cm³/g STP

Isotherm Table

Relative Pressure (P/Po)	Experimental Quantity Adsorbed (cm³/g STP)	Fitted Quantity Adsorbed (cm³/g STP)	Absolute Residual (cm³/g STP)	Relative Residual
0.000001585	11.1819	14.6082	-3.4263	-0.306414
0.000001995	15.1867	16.2457	-1.0590	-0.069733
0.000002512	17.7394	18.1208	-0.3814	-0.021501
0.000003162	20.3041	20.1800	0.1241	0.006111
0.000003981	22.8627	22.4561	0.4066	0.017785
0.000005012	24.9582	25.0255	-0.0673	-0.002698
0.000006310	27.2698	27.8774	-0.6076	-0.022281
0.000007943	30.0463	30.9201	-0.8738	-0.029082
0.000010000	32.8810	34.1622	-1.2812	-0.038964
0.000012589	36.0327	37.6590	-1.6262	-0.045132
0.000015849	39.7310	41.2744	-1.5434	-0.038846
0.000019953	43.8000	44.8293	-1.0294	-0.023502
0.000025119	48.5502	48.4108	0.1393	0.002870
0.000031623	53.9384	52.3841	1.5544	0.028818
0.000039811	60.0400	57.4980	2.5420	0.042338
0.000050119	66.8012	64.1285	2.6727	0.040010
0.000063096	74.0812	72.3868	1.6945	0.022873
0.000079433	81.7856	81.2917	0.4940	0.006040
0.000100000	89.8125	87.6790	2.1336	0.023756
0.000125892	98.1839	95.0255	3.1584	0.032168
0.000158490	106.9427	107.6268	-0.6841	-0.006397
0.000199526	116.1291	121.6421	-5.5130	-0.047473
0.000251188	125.6746	128.5043	-2.8297	-0.022516
0.000316228	135.7232	135.2219	0.5013	0.003694
0.000398107	146.2185	150.5748	-4.3563	-0.029793
0.000501187	157.1794	157.8998	-0.7204	-0.004583
0.000630958	168.5293	172.7161	-4.1869	-0.024844
0.000794328	180.2404	181.5724	-1.3320	-0.007390
0.001000000	192.2199	194.7998	-2.5799	-0.013422

pharmaceutics

MDPI

Isotherm Table

Relative Pressure (P/Po)	Experimental Quantity Adsorbed (cm³/g STP)	Fitted Quantity Adsorbed (cm³/g STP)	Absolute Residual (cm³/g STP)	Relative Residual
0.001258925	204.1964	203.5968	0.5996	0.002936
0.001584895	216.1504	214.1578	1.9926	0.009219
0.001995263	227.7553	221.6002	6.1551	0.027025
0.002511882	238.9223	228.8981	10.0242	0.041956
0.003162276	249.5383	237.4676	12.0707	0.048372
0.003981066	259.6537	244.7139	14.9398	0.057537
0.005011868	269.1967	252.0868	17.1100	0.063559
0.006309579	278.1369	260.7235	17.4134	0.062607
0.007943276	286.0774	275.7848	10.2927	0.035979
0.010000000	294.9199	294.5959	0.3240	0.001098
0.012355640	300.9041	313.5923	-12.6882	-0.042167
0.015186320	307.3687	329.8877	-22.5190	-0.073264
0.018485530	314.7681	340.0100	-25.2419	-0.080192
0.022294740	323.1802	347.5940	-24.4138	-0.075542
0.026653420	332.7084	353.9490	-21.2406	-0.063842
0.031598160	343.5117	359.5643	-16.0525	-0.046731
0.037162240	355.9032	364.7234	-8.8203	-0.024783
0.043374470	369.8471	374.0147	-4.1677	-0.011269
0.050259210	385.0051	391.5913	-6.5862	-0.017107
0.057835260	400.7480	396.6105	4.1375	0.010324
0.066115920	415.1854	401.2743	13.9111	0.033506
0.075109080	425.1353	418.3869	6.7484	0.015874
0.084815920	433.7849	433.4008	0.3840	0.000885
0.095232370	443.3458	437.5219	5.8239	0.013136
0.106348200	455.2402	454.5836	0.6566	0.001442
0.118147500	470.1599	474.6016	-4.4417	-0.009447
0.130609100	490.5150	477.9971	12.5179	0.025520
0.143706600	499.9751	490.8565	9.1187	0.018238
0.157410500	503.1167	495.8064	7.3103	0.014530
0.171685500	505.2351	498.2627	6.9724	0.013800
0.186492100	507.4639	500.5118	6.9521	0.013700
0.201792100	509.4099	502.5830	6.8269	0.013402
0.217539500	511.1064	504.4983	6.6082	0.012929
0.233689500	512.6683	506.2754	6.3929	0.012470
0.250196100	514.0716	507.9289	6.1427	0.011949

pharmaceutics

MDPI

Isotherm Table

Relative Pressure (P/Po)	Experimental Quantity Adsorbed (cm³/g STP)	Fitted Quantity Adsorbed (cm³/g STP)	Absolute Residual (cm³/g STP)	Relative Residual
0.267011800	515.3471	509.4711	5.8760	0.011402
0.284089500	516.5311	510.9123	5.6189	0.010878
0.301380300	517.5851	512.2613	5.3238	0.010286
0.318838200	518.5381	513.5260	5.0122	0.009666
0.336417100	519.4303	514.7131	4.7172	0.009082
0.354071100	520.2288	515.8289	4.3999	0.008458
0.371757900	520.9630	516.8786	4.0844	0.007840
0.389435500	521.6453	517.8672	3.7781	0.007243
0.407065800	522.2526	518.7992	3.4534	0.006612
0.424610500	522.8156	519.6787	3.1368	0.006000
0.442034200	523.3393	520.5094	2.8299	0.005407
0.459305300	523.8234	521.2951	2.5283	0.004827
0.476393400	524.2798	522.0402	2.2396	0.004272
0.493271100	524.6863	522.7529	1.9334	0.003685
0.509911800	525.0291	523.4422	1.5870	0.003023
0.526293400	525.3413	524.0801	1.2612	0.002401
0.542394700	525.6308	524.6793	0.9515	0.001810
0.558200000	525.9032	525.2450	0.6582	0.001252
0.573690800	526.1671	525.7801	0.3870	0.000735
0.588853900	526.4153	526.2867	0.1286	0.000244
0.603677600	526.6401	526.7666	-0.1266	-0.000240
0.618153900	526.8473	527.2216	-0.3743	-0.000710
0.632272400	527.0399	527.6531	-0.6132	-0.001163
0.646028900	527.2169	528.0626	-0.8457	-0.001604
0.659417100	527.3803	528.4513	-1.0710	-0.002031
0.672435500	527.5382	528.8204	-1.2822	-0.002431
0.685081600	527.6914	529.1712	-1.4798	-0.002804
0.697355300	527.8398	529.5048	-1.6650	-0.003154
0.709256600	527.9834	529.8222	-1.8388	-0.003483
0.720789500	528.1220	530.1248	-2.0028	-0.003792
0.731953900	528.2560	530.4138	-2.1578	-0.004085
0.742756600	528.3857	530.6921	-2.3064	-0.004365
0.753200000	528.5116	530.9747	-2.4631	-0.004660
0.763289500	528.6385	531.2468	-2.6083	-0.004934
0.773030300	528.7696	531.4893	-2.7198	-0.005144

pharmaceutics

MDPI

Isotherm Table

Relative Pressure (P/Po)	Experimental Quantity Adsorbed (cm³/g STP)	Fitted Quantity Adsorbed (cm³/g STP)	Absolute Residual (cm³/g STP)	Relative Residual
0.782430300	528.9061	531.7169	-2.8109	-0.005314
0.791496100	529.0490	531.9322	-2.8832	-0.005450
0.800232900	529.1991	532.1364	-2.9373	-0.005550
0.808648700	529.4027	532.3304	-2.9276	-0.005530
0.816752600	529.6313	532.5149	-2.8837	-0.005445
0.824552600	529.8099	532.6906	-2.8807	-0.005437
0.832053900	529.9553	532.8581	-2.9028	-0.005477
0.839267100	530.0903	533.0180	-2.9277	-0.005523
0.846200000	530.2307	533.1710	-2.9403	-0.005545
0.852860500	530.3884	533.3179	-2.9295	-0.005523
0.859257900	530.5635	533.4607	-2.8971	-0.005460
0.865398700	530.7478	533.6072	-2.8594	-0.005388
0.871292100	530.9342	533.7744	-2.8402	-0.005349
0.876947400	531.1185	533.9031	-2.7847	-0.005243
0.882369700	531.3060	534.0221	-2.7160	-0.005112
0.887569700	531.4962	534.1341	-2.6380	-0.004963
0.892553900	531.6861	534.2404	-2.5542	-0.004804
0.897328900	531.8738	534.3416	-2.4678	-0.004640
0.901905300	532.0599	534.4384	-2.3785	-0.004470
0.906286800	532.2601	534.5312	-2.2711	-0.004267
0.910484200	532.4710	534.6211	-2.1501	-0.004038
0.914501300	532.6853	534.7098	-2.0245	-0.003801
0.918347400	532.8974	534.8392	-1.9419	-0.003644
0.922026300	533.1026	534.9375	-1.8349	-0.003442
0.925547400	533.2986	535.0176	-1.7190	-0.003223
0.928915800	533.5058	535.0920	-1.5862	-0.002973
0.932136800	533.7288	535.1626	-1.4337	-0.002686
0.935218400	533.9610	535.2306	-1.2696	-0.002378
0.938163200	534.1966	539.8346	-5.6381	-0.010554
0.940978900	534.4315	539.8880	-5.4565	-0.010210
0.943669700	534.6622	539.9388	-5.2766	-0.009869
0.946242100	534.8861	539.9872	-5.1011	-0.009537
0.948700000	535.1013	540.0333	-4.9320	-0.009217
0.951048700	535.3112	540.0772	-4.7660	-0.008903
0.953292100	535.5374	540.1191	-4.5817	-0.008555

Isotherm Table

Relative Pressure (P/Po)	Experimental Quantity Adsorbed (cm³/g STP)	Fitted Quantity Adsorbed (cm³/g STP)	Absolute Residual (cm³/g STP)	Relative Residual
0.955435500	535.7808	540.1589	-4.3781	-0.008171
0.957482900	536.0395	540.1969	-4.1574	-0.007756
0.959438200	536.3112	540.2330	-3.9219	-0.007313
0.961305300	536.5940	540.2675	-3.6735	-0.006846
0.963088200	536.8860	540.3003	-3.4143	-0.006359
0.964789500	537.1854	540.3315	-3.1461	-0.005857
0.966414500	537.4908	540.3613	-2.8706	-0.005341
0.967965800	537.8005	540.3898	-2.5893	-0.004815
0.969447400	538.1133	540.4168	-2.3035	-0.004281
0.970860500	538.4274	540.4425	-2.0151	-0.003743
0.972209200	538.7419	540.4671	-1.7252	-0.003202
0.973496100	539.0557	540.4905	-1.4348	-0.002662
0.974725000	539.3748	540.5128	-1.1380	-0.002110
0.975897400	539.7499	540.5340	-0.7842	-0.001453
0.977015800	540.1659	540.5542	-0.3883	-0.000719
0.978082900	540.5970	540.5735	0.0235	0.000044
0.979101300	541.0231	540.5919	0.4312	0.000797
0.980072400	541.4285	540.6093	0.8192	0.001513
0.980998700	541.8112	540.6260	1.1852	0.002187
0.981882900	542.1963	540.6419	1.5544	0.002867
0.982726300	542.5817	540.6571	1.9246	0.003547
0.983530300	542.9634	540.6715	2.2919	0.004221
0.984297400	543.3387	540.6853	2.6534	0.004883
0.985028900	543.7052	540.6984	3.0068	0.005530
0.985727600	544.0617	540.7108	3.3508	0.006159
0.986392100	544.4055	540.7227	3.6828	0.006765
0.987027600	544.7375	540.7341	4.0034	0.007349
0.987632900	545.0558	540.7449	4.3109	0.007909
0.988209200	545.3600	540.7552	4.6048	0.008444
0.988760500	545.6513	540.7650	4.8863	0.008955
0.989285500	545.9284	540.7744	5.1540	0.009441
0.989785500	546.1916	540.7832	5.4084	0.009902
0.990263200	546.4419	540.7917	5.6501	0.010340
0.990718400	546.6405	540.7998	5.8407	0.010685
0.991151300	546.7447	540.8075	5.9372	0.010859

pharmaceutics

MDPI

Isotherm Table

Relative Pressure (P/Po)	Experimental Quantity Adsorbed (cm³/g STP)	Fitted Quantity Adsorbed (cm³/g STP)	Absolute Residual (cm³/g STP)	Relative Residual
0.991565800	546.8220	540.8149	6.0071	0.010985
0.991959200	546.9252	540.8218	6.1033	0.011159

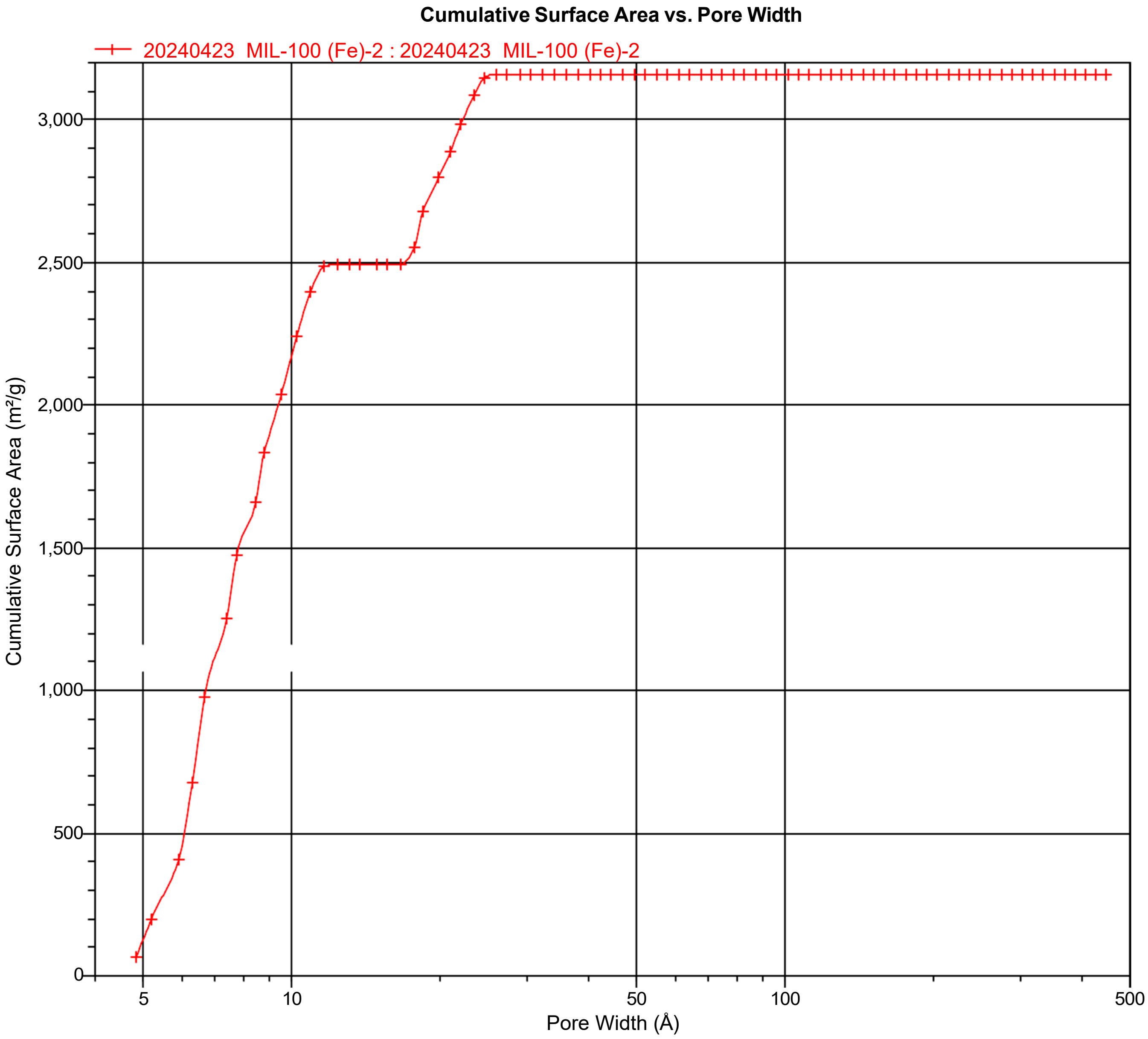
Cumulative Surface Area vs. Pore Width
20240423 MIL-100 (Fe)-2 : 20240423 MIL-100 (Fe)-2
Cumulative Surface Area (m²/g)
3,000
2,500
2,000
1,500
1,000
500
0
5
10
50
100
500
Pore Width (Å)

pharmaceutics

MDPI

.

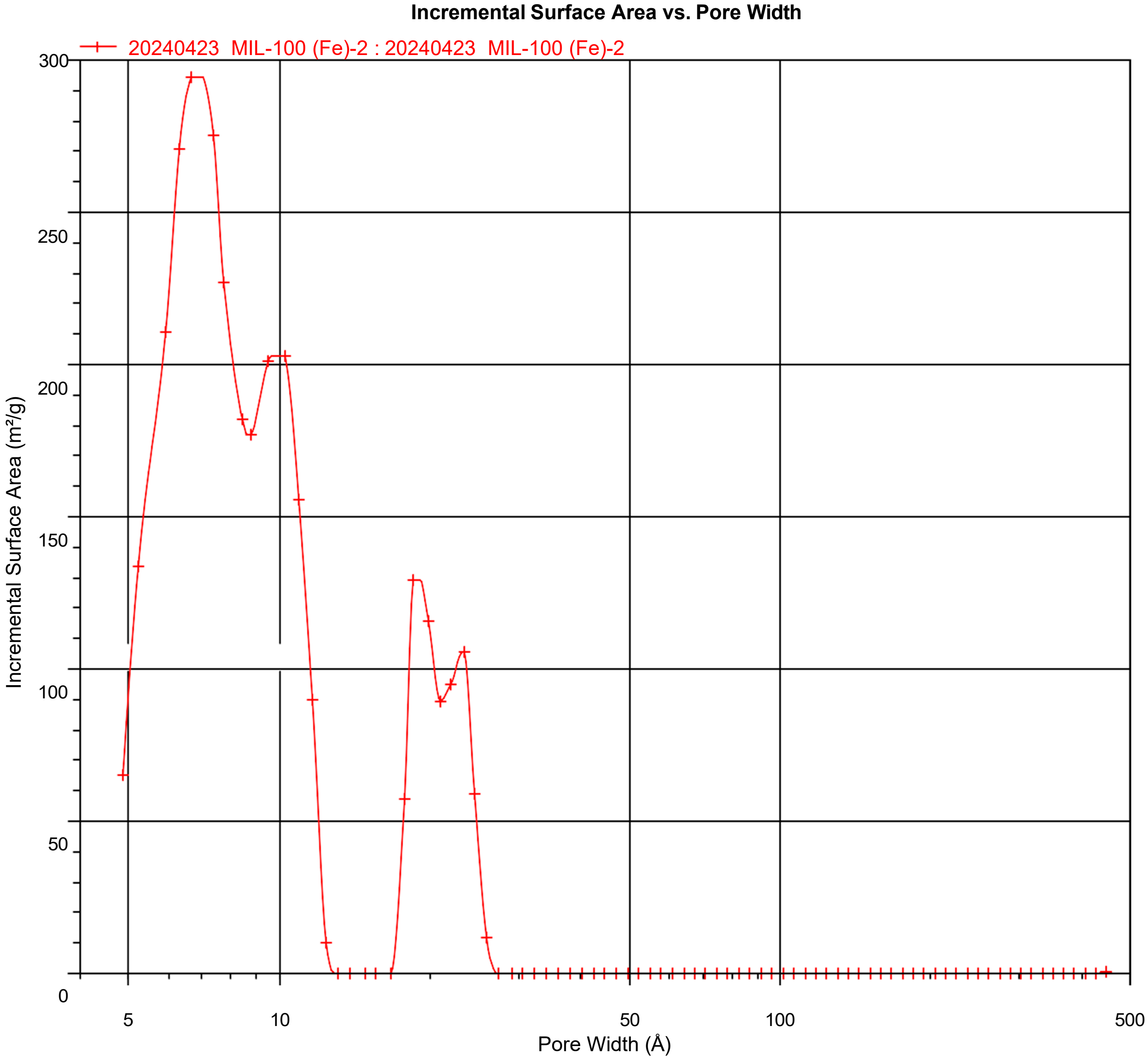

pharmaceutics

MDPI

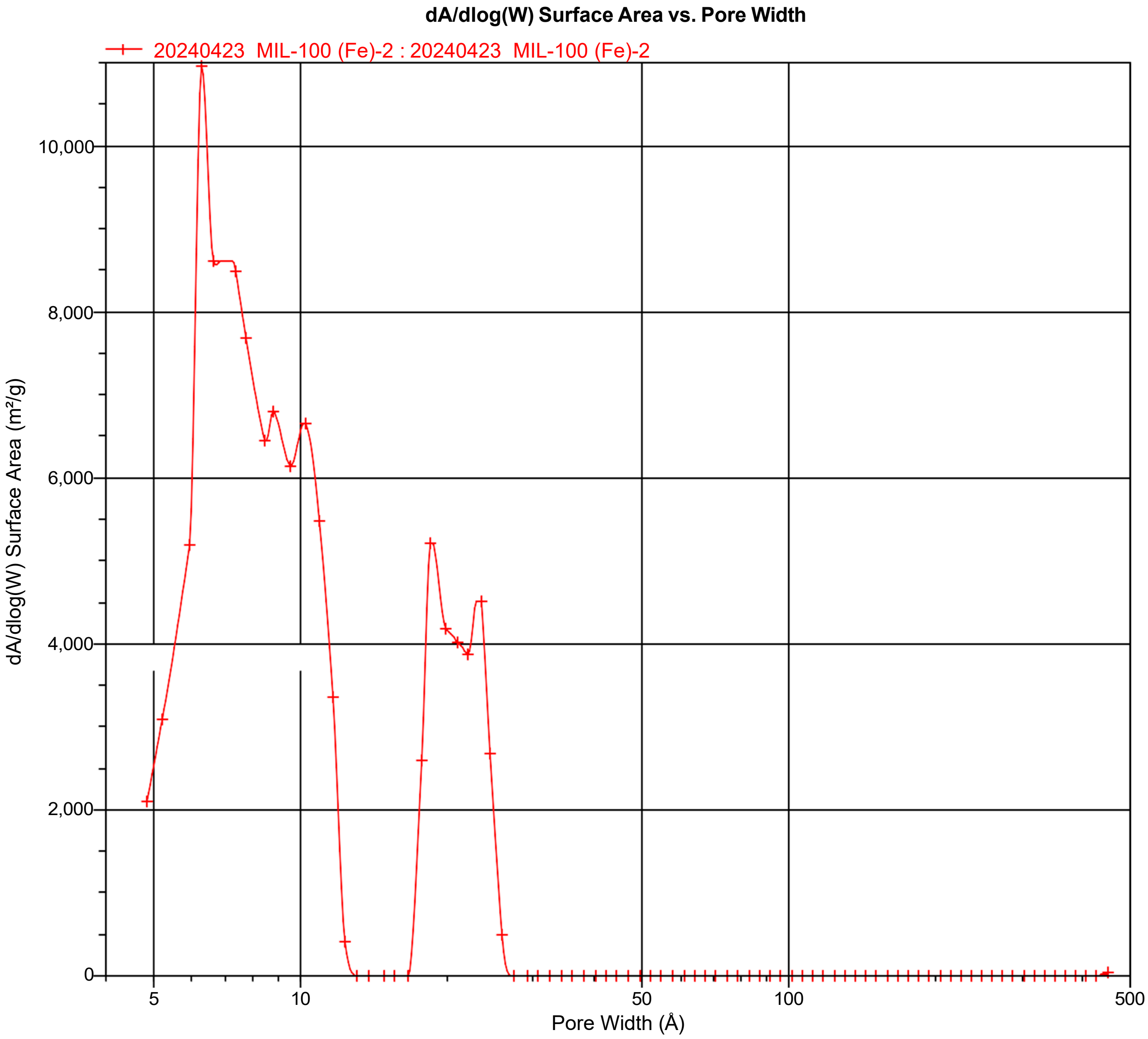

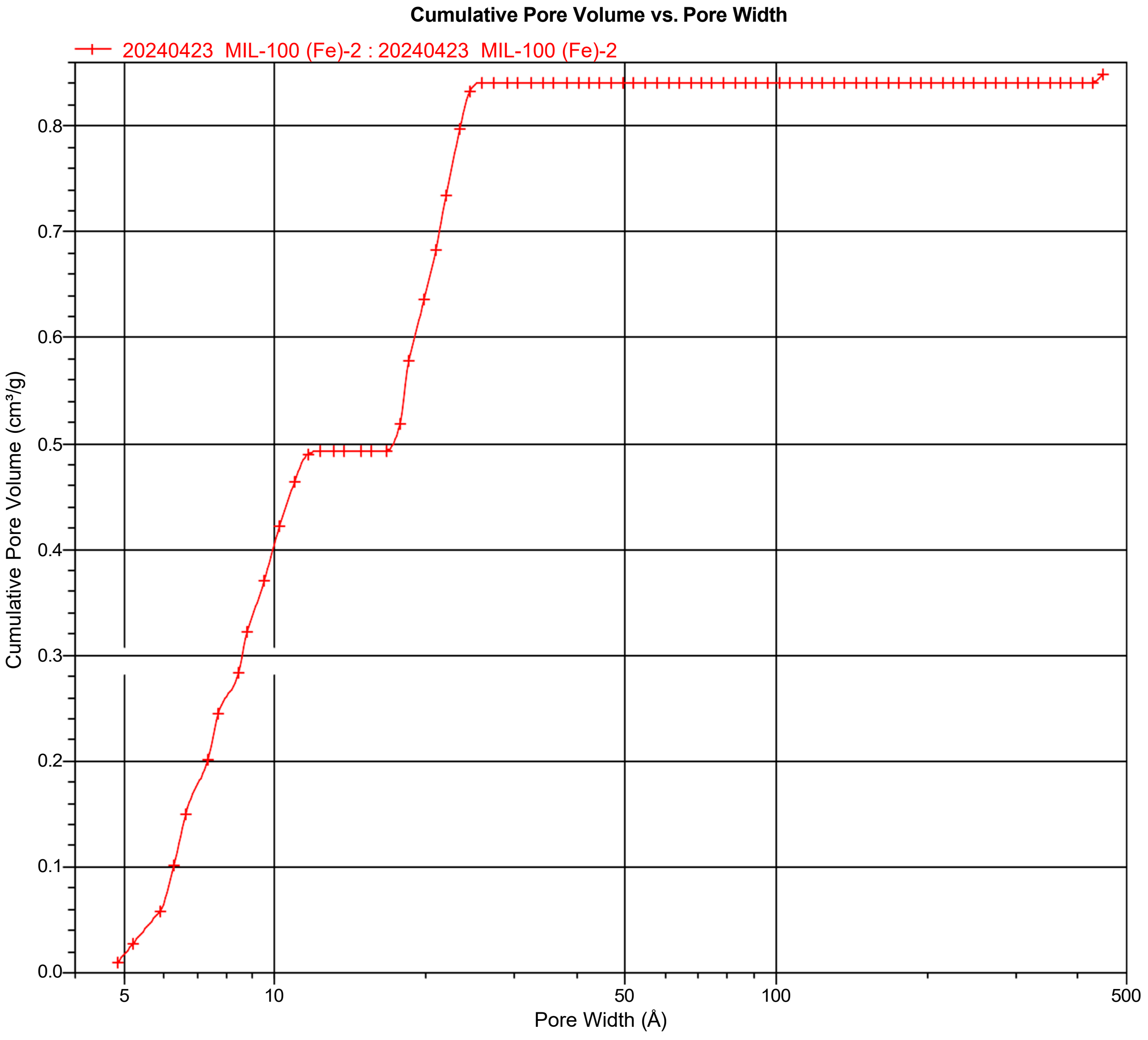
Cumulative Pore Volume vs. Pore Width
20240423 MIL-100 (Fe)-2 : 20240423 MIL-100 (Fe)-2
Cumulative Pore Volume (cm³/g)
0.0
0.1
0.2
0.3
0.4
0.5
0.6
0.7
0.8
5
10
50
100
500
Pore Width (Å)

pharmaceutics

MDPI

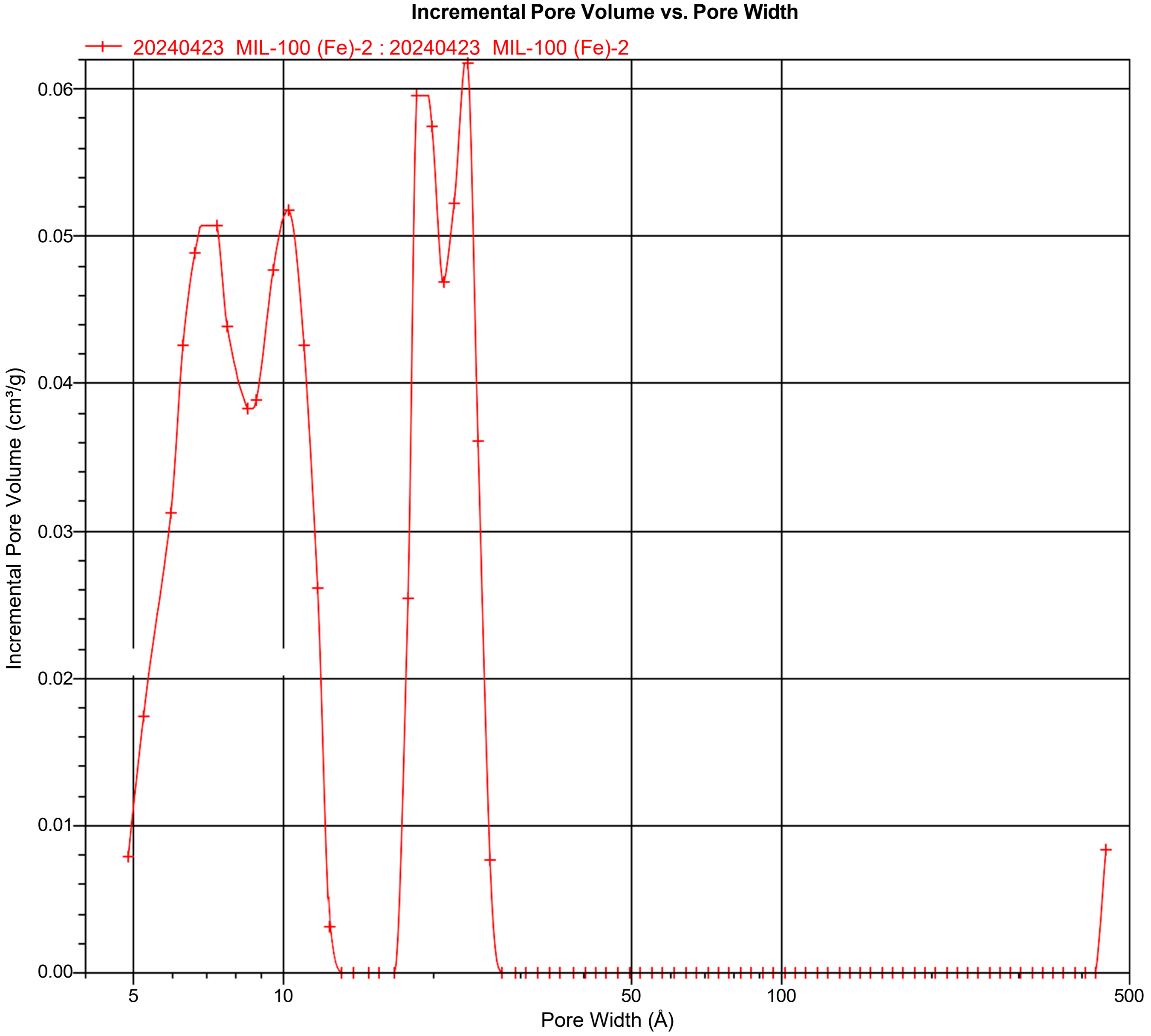

pharmaceutics

MDPI

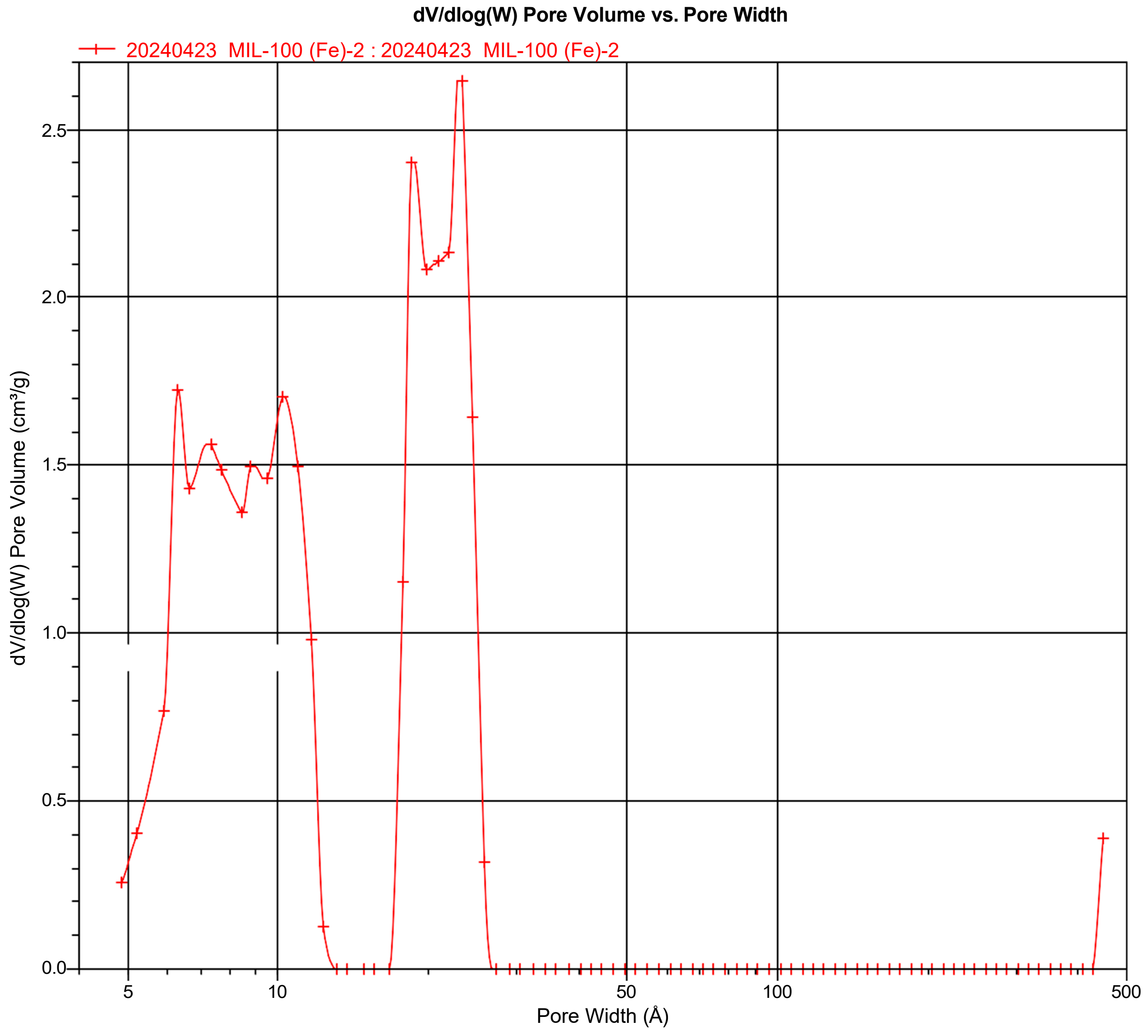

pharmaceutics

MDPI

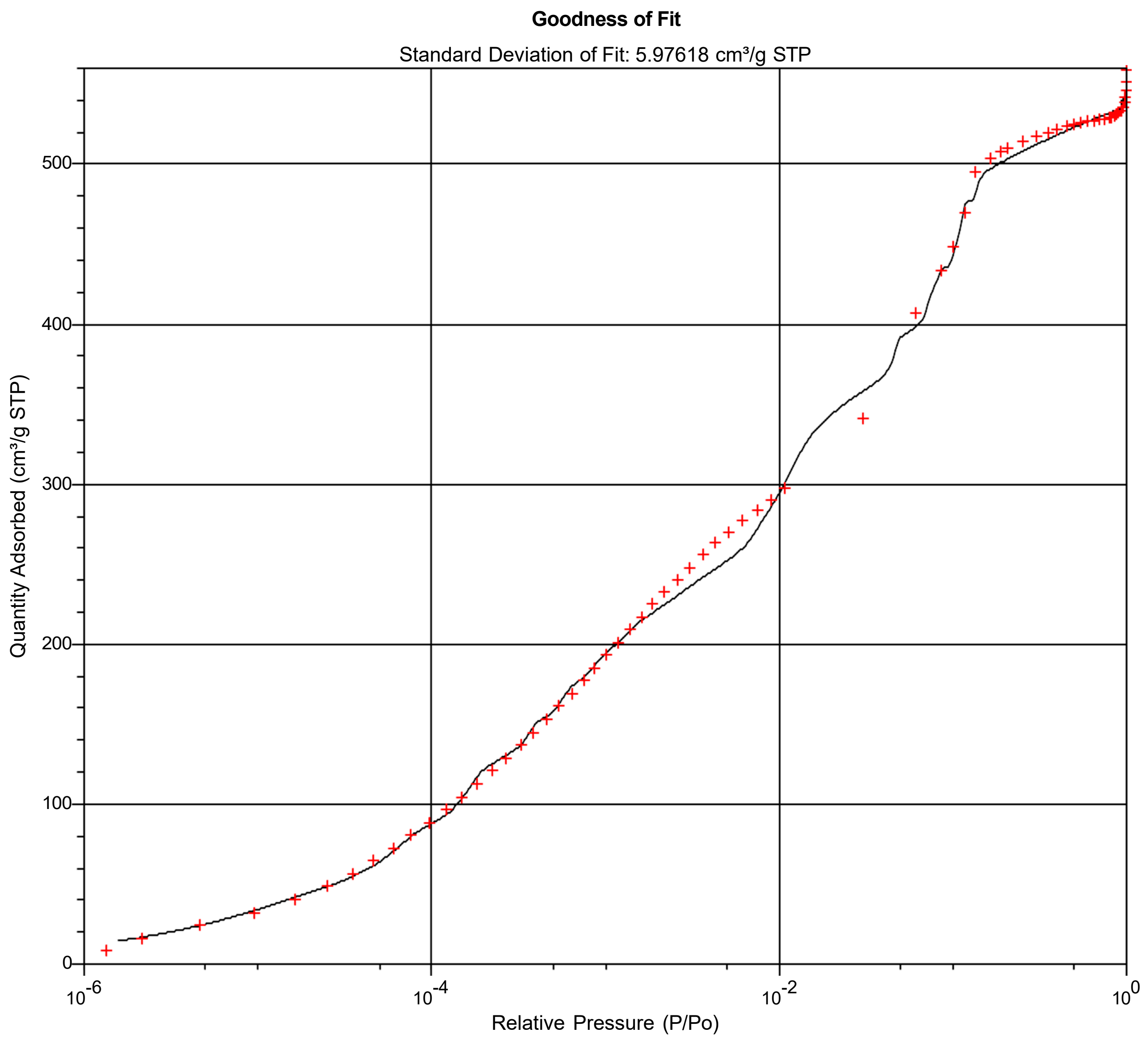

pharmaceutics

MDPI

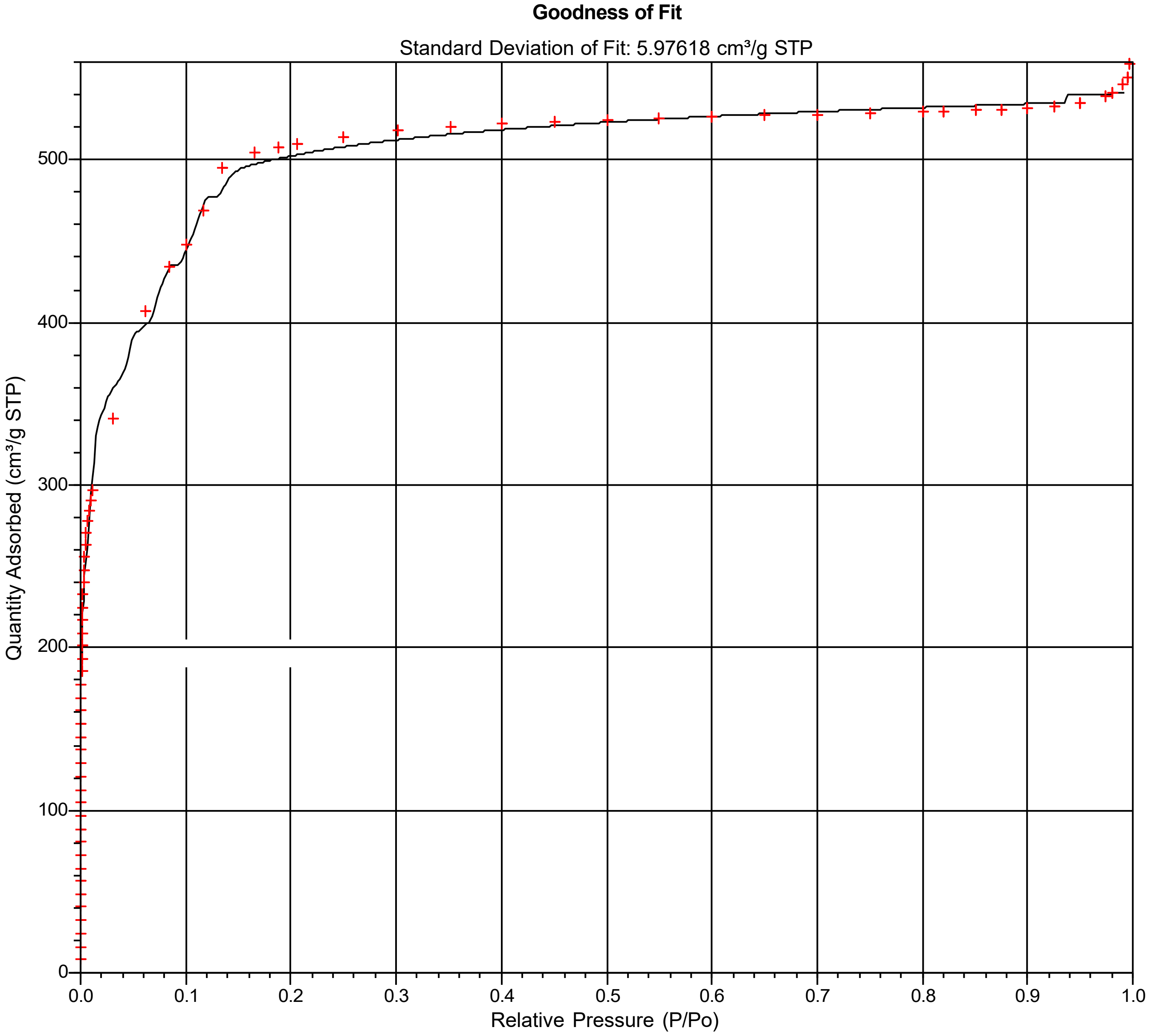

Sample Information

Method: Default
Sample: 20240423 MIL-100 (Fe)-2
Operator:
Submitter: Micromeritics
Mass type: Calculated
Empty tube: 38.3235 g
Sample + tube: 38.3985 g
Sample mass: 0.0750 g
Density: 1.000 g/cm³
Type of data: Automatically collected
Instrument type: 2020
Original instrument type: 2020
Comments: Use sample tube with isothermal jacket and filler rod. Follow the instructions on the Silica-Alumina sample data sheet for sample preparation and special instructions. Be sure to use specifications for the current lot.

Sample Tube

Sample tube: Sample Tube
Warm free space: 1.0000 cm³
Cold free space: 1.0000 cm³
Non-ideality factor: 0.0000620
Use isothermal jacket: Yes
Use filler rod: Yes
Vacuum seal type: Seal Frit

Degas Conditions

Degas conditions: Degas Conditions

Evacuation Phase

Temperature ramp rate: 10.0 °C/min
Target temperature: 90 °C
Evacuation rate: 5.0 mmHg/s
Unrest. evacuation from: 5.0 mmHg
Vacuum level: 1.000000e-002 mmHg
Evacuation time: 60 min

pharmaceutics

MDPI

Heading Phase

Ramp rate: 10.0 °C/min
Hold temp: 350 °C
Hold time: 360 min

Evacuation and Heating Phases

Hold pressure: 10 mmHg

Backfill

Backfill sample tube: Yes

Analysis Conditions

Analysis conditions: Silica Alumina, nitrogen @ 77.35 K
Absolute pressure dosing: No

Pressure Table

Relative Pressure (P/Po)	Rel. Pressure Increment (P/Po)
0.010000000	
0.030000000	
0.060000000	
0.080000000	
0.100000000	
0.120000000	
0.140000000	
0.160000000	
0.180000000	
0.200000000	
0.250000000	
0.300000000	
0.350000000	
0.400000000	
0.450000000	
0.500000000	
0.550000000	
0.600000000	
0.650000000	
0.700000000	
0.750000000	

Pressure Table

Relative Pressure (P/Po)	Rel. Pressure Increment (P/Po)
0.800000000	
0.820000000	
0.850000000	
0.875000000	
0.900000000	
0.925000000	
0.950000000	
0.975000000	
0.980000000	
0.990000000	
0.995000000	
0.998000000	
0.990000000	
0.980000000	
0.975000000	
0.950000000	
0.925000000	
0.900000000	
0.875000000	
0.850000000	
0.825000000	
0.800000000	
0.750000000	
0.700000000	
0.650000000	
0.600000000	
0.550000000	
0.500000000	
0.450000000	
0.400000000	
0.350000000	
0.300000000	
0.250000000	
0.200000000	
0.140000000	

Preparation

Fast evacuation: No
Unrestricted evacuation from: 5.0 mmHg
Vacuum setpoint: 10 μmHg
Evacuation time: 1.00 h

Leak test: No
Use TranSeal: No

Free Space

Measured before analysis
Lower Dewar for evacuation: No
Evacuation time: 2.00 h
Outgas test: No

Po and Temperature

Po type: Measured at intervals in Psat tube
Measurement interval: 120 min
Temperature type: Calculated from Po or Psat

Dosing

Use first pressure fixed dose: No
Use maximum volume increment: No
Target tolerance: 5.0% or 5.000 mmHg
Low pressure dosing: Yes
Dose amount: 8.0000 cm^3/g STP
Minimum equilibration delay: 0.00 h
Maximum equilibration delay: 2.00 h
Maximum number of decants: 6

Equilibration

	Relative Pressure (P/Po)	Equilibration Interval (s)
1	0.010000000	20
2	0.990000000	10

Minimum equilibration delay at P/Po >= 0.995: 600 s

Sample Backfill

Backfill at start of analysis: Yes
Backfill at end of analysis: Yes

pharmaceutics

MDPI

Sample Backfill

Backfill gas: N2

Adsorptive Properties

Adsorptive: Nitrogen @ 77.35 K (N2)
Non-condensing adsorptive: No
Maximum manifold pressure: 925.00 mmHg
Therm. tran. hard-sphere diameter: 3.8600 Å
Molecular cross-sectional area: 0.162 nm²
Adsorbate molecular weight: 28.01
Ideal gas law with non-ideality correction
Non-ideality factor: 0.0000660
Density conversion factor: 0.0015468
Dosing method: Normal

Psat vs. Temperature Table

	Saturation Pressure (mmHg)	Temperature (°C)
1	600.193	-197.750
2	634.512	-197.300
3	674.383	-196.800
4	720.420	-196.250
5	742.119	-196.000
6	759.833	-195.800
7	777.867	-195.600
8	805.525	-195.300
9	853.268	-194.800
10	903.122	-194.300

pharmaceutics

MDPI

Raw data for SYA@MIL-100(Fe) at 12 h

Summary Report

Surface Area

Single point surface area at P/Po = 0.249552124: 828.0805 m²/g

BET Surface Area: 1,006.6398 m²/g

Langmuir Surface Area: 1,196.2282 m²/g

t-Plot Micropore Area: 643.3374 m²/g

t-Plot external surface area: 363.3024 m²/g

BJH Adsorption cumulative surface area of pores
between 17.000 Å and 3,000.000 Å width: 73.8041 m²/g

BJH Desorption cumulative surface area of pores
between 17.000 Å and 3,000.000 Å width: 77.9085 m²/g

Pore Volume

Single point adsorption total pore volume of pores
less than 3,873.040 Å width at P/Po = 0.995000000: 0.422034 cm³/g

Single point desorption total pore volume of pores
less than 3,873.040 Å width at P/Po = 0.995000000: 0.429009 cm³/g

t-Plot micropore volume: 0.231148 cm³/g

BJH Adsorption cumulative volume of pores
between 17.000 Å and 3,000.000 Å width: 0.065057 cm³/g

BJH Desorption cumulative volume of pores
between 17.000 Å and 3,000.000 Å width: 0.077304 cm³/g

Pore Size

Adsorption average pore diameter (4V/A by BET): 16.770 Å

Desorption average pore diameter (4V/A by BET): 17.047 Å

pharmaceutics

Pore Size

BJH Adsorption average pore width (4V/A): 35.259 Å

BJH Desorption average pore width (4V/A): 39.690 Å

Horvath-Kawazoe

Maximum pore volume at P/Po = 0.097082779: 0.366316 cm³/g

Median pore width: 14.955 Å

pharmaceutics

MDPI

Isotherm Tabular Report

Relative Pressure (P/Po)	Absolute Pressure (mmHg)	Quantity Adsorbed (cm³/g STP)	Elapsed Time (h:min)	Saturation Pressure (mmHg)
			03:43	758.929199
0.000006256	0.004747	8.1595	04:45	
0.000010307	0.007819	16.3087	05:37	
0.000015745	0.011941	24.4393	06:28	
0.000023195	0.017587	32.5445	07:16	
0.000033953	0.025740	40.6111	08:02	
0.000050622	0.038372	48.7871	08:30	
0.000076145	0.057712	56.9539	08:57	
0.000116473	0.088268	65.1053	09:21	
0.000181253	0.137349	73.2291	09:42	
0.000281976	0.213655	81.3140	10:02	
0.000435813	0.330195	89.3418	10:19	
0.000667544	0.505730	97.3201	10:36	
0.001010192	0.765264	105.1213	10:53	
0.001518466	1.150221	112.7015	11:10	
0.002262674	1.713840	119.9454	11:25	
0.003345552	2.533894	126.9069	11:40	
0.004813362	3.645369	133.3870	11:55	
			12:04	757.314575
0.006971538	5.279452	139.6279	12:12	
0.009202873	6.968794	145.2675	12:25	
0.011729800	8.881999	150.4726	12:32	
0.029285420	22.173971	180.8872	12:46	
0.065299574	49.440666	214.9600	12:55	
0.078115104	59.141853	221.7687	13:02	
0.097082779	73.499443	236.8215	13:11	
0.121872144	92.264420	245.3698	13:17	
0.143596729	108.709206	247.8334	13:21	
0.164029183	124.175774	249.4710	13:24	
0.181283093	137.236313	250.6439	13:26	
0.200610839	151.865845	251.6882	13:29	
0.250191350	189.396515	253.7322	13:32	
0.300651680	227.593216	255.1703	13:34	
0.351507954	266.087708	256.1876	13:37	
0.400532818	303.194794	256.8561	13:40	
0.450326101	340.884094	257.3184	13:42	

pharmaceutics

MDPI

Isotherm Tabular Report

Relative Pressure (P/Po)	Absolute Pressure (mmHg)	Quantity Adsorbed (cm³/g STP)	Elapsed Time (h:min)	Saturation Pressure (mmHg)
0.500397878	378.783508	257.6590	13:44	
0.550599114	416.778290	257.7877	13:47	
0.619669861	469.057343	257.8410	13:49	
0.669675390	506.901855	257.8909	13:52	
0.719694743	544.758301	257.9243	13:54	
0.769740028	582.630981	257.9011	13:57	
0.819554467	620.330750	258.0769	13:59	
0.820471881	621.019409	258.1733	14:01	
0.850671562	643.868774	258.3414	14:04	
			14:05	756.891174
0.894297473	676.885864	258.7899	14:08	
0.900282520	681.415894	258.9920	14:10	
0.925148608	700.236816	259.6072	14:12	
0.949827291	718.915894	260.7963	14:15	
0.974672252	737.720825	263.3648	14:17	
0.980427961	742.077271	264.9264	14:19	
0.990442633	749.657288	268.3270	14:22	
0.994604184	752.807129	271.7032	14:25	
0.996955303	754.586670	278.4744	14:35	
0.977396569	739.782837	267.2507	14:40	
0.958779783	725.691956	262.9327	14:42	
0.932662656	705.924133	260.6361	14:45	
0.906947275	686.460388	259.6294	14:47	
0.881488166	667.190613	259.0693	14:49	
0.856518536	648.291321	258.7754	14:52	
0.831305699	629.207947	258.6080	14:54	
0.806416467	610.369507	258.4604	14:56	
0.781231773	591.307434	258.3896	14:59	
0.731172201	553.417786	258.3778	15:01	
0.681156819	515.561584	258.3677	15:03	
0.631072289	477.653046	258.3883	15:05	
0.581076059	439.811340	258.3140	15:08	
0.531205948	402.065094	258.2132	15:10	
0.481009692	364.071991	257.9827	15:13	
0.450310965	340.836395	257.6987	15:15	
0.399794934	302.601257	257.2041	15:17	

Isotherm Tabular Report

Relative Pressure (P/Po)	Absolute Pressure (mmHg)	Quantity Adsorbed (cm³/g STP)	Elapsed Time (h:min)	Saturation Pressure (mmHg)
0.350032034	264.936157	256.5181	15:19	
0.300006814	227.072510	255.5005	15:22	
0.249995363	189.219284	254.0439	15:25	
0.200122148	151.470688	251.9592	15:29	
0.140639141	106.448524	247.8512	15:34	

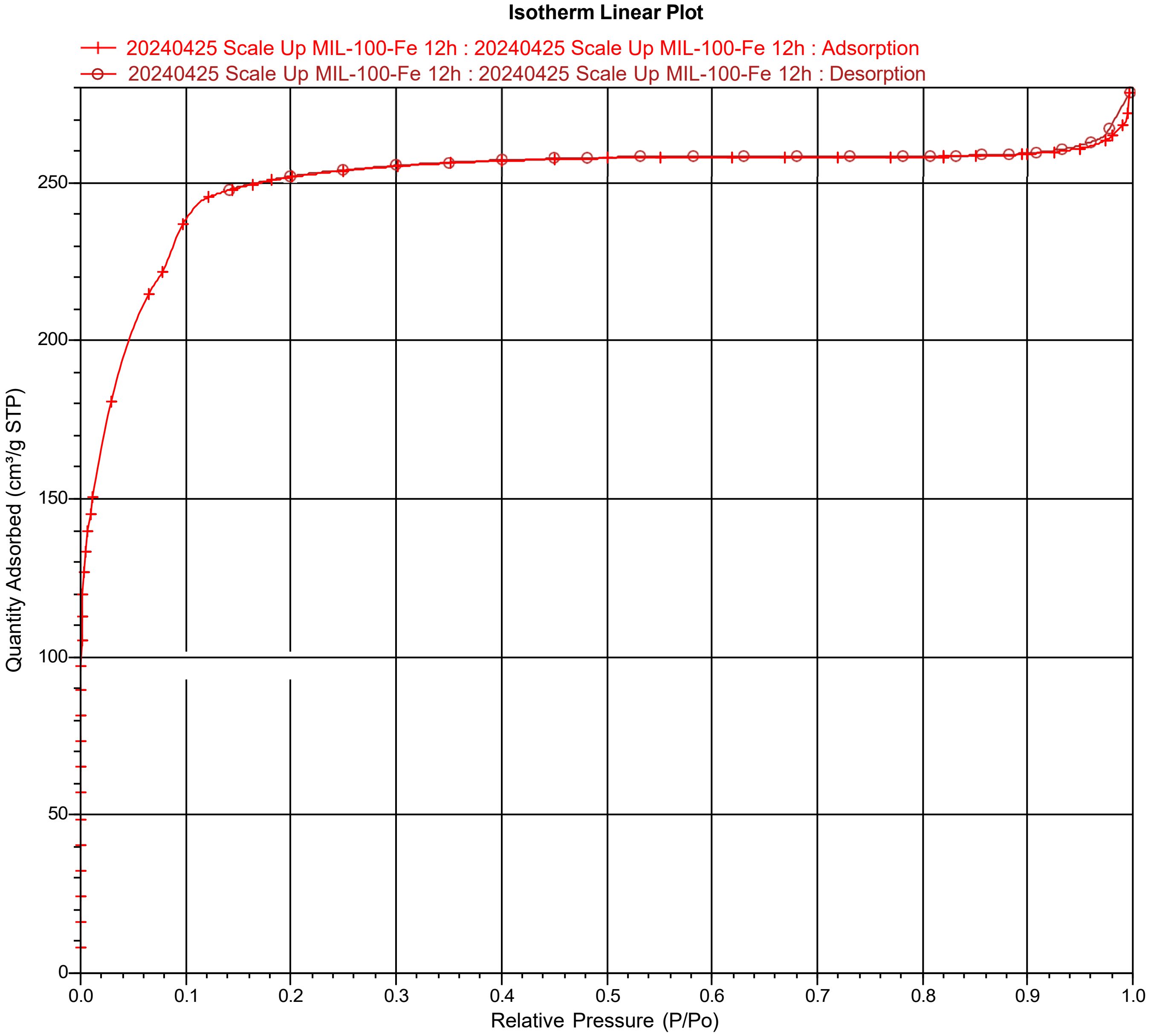
Isotherm Linear Plot
20240425 Scale Up MIL-100-Fe 12h : 20240425 Scale Up MIL-100-Fe 12h : Adsorption
20240425 Scale Up MIL-100-Fe 12h : 20240425 Scale Up MIL-100-Fe 12h : Desorption
Quantity Adsorbed (cm³/g STP)
Relative Pressure (P/Po)
0
50
100
150
200
250
0.0
0.1
0.2
0.3
0.4
0.5
0.6
0.7
0.8
0.9
1.0

pharmaceutics

MDPI

BET Report

BET surface area: 1006.6398 ± 13.9488 m²/g
Slope: 0.004280 ± 0.000060 g/cm³ STP
Y-intercept: 0.000043 ± 0.000005 g/cm³ STP
C: 99.610233
Qm: 231.2744 cm³/g STP
Correlation coefficient: 0.9997083
Molecular cross-sectional area: 0.1620 nm²

Relative Pressure (P/Po)	Quantity Adsorbed (cm³/g STP)	1/[Q(Po/P - 1)]
0.029285420	180.8872	0.000167
0.065299574	214.9600	0.000325
0.078115104	221.7687	0.000382
0.097082779	236.8215	0.000454
0.121872144	245.3698	0.000566

pharmaceutics

MDPI

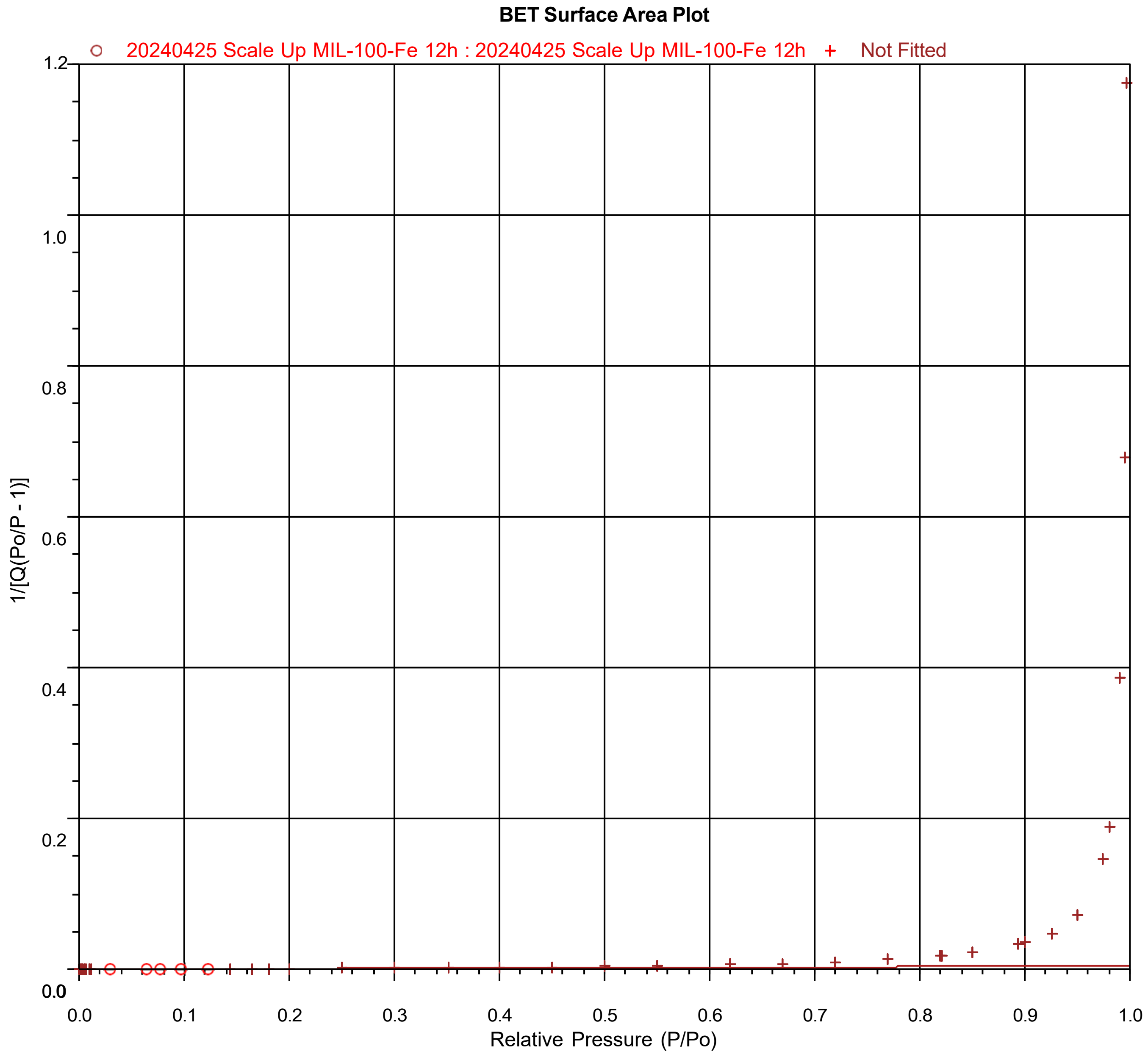

pharmaceutics

MDPI

t-Plot Report

Micropore volume: 0.231148 cm³/g
Micropore area: 643.3374 m²/g
External surface area: 363.3024 m²/g
Slope: 23.487355 ± 5.593135 cm³/g·Å STP
Y-intercept: 149.436096 ± 22.769035 cm³/g STP
Correlation coefficient: 0.863789
Surface area correction factor: 1.000
Density conversion factor: 0.0015468
Total surface area (BET): 1006.6398 m²/g
Thickness range: 3.5000 Å to 5.0000 Å
Thickness equation: Harkins and Jura

Thickness Curve

t = [13.99 / (0.034 - log(P/Po))] ^ 0.5

t-Plot Report - Data

Relative Pressure (P/Po)	Statistical Thickness (Å)	Quantity Adsorbed (cm³/g STP)	Fitted
0.000006256	1.6343	8.1595	
0.000010307	1.6692	16.3087	
0.000015745	1.7007	24.4393	
0.000023195	1.7311	32.5445	
0.000033953	1.7626	40.6111	
0.000050622	1.7976	48.7871	
0.000076145	1.8355	56.9539	
0.000116473	1.8777	65.1053	
0.000181253	1.9249	73.2291	
0.000281976	1.9758	81.3140	
0.000435813	2.0301	89.3418	
0.000667544	2.0878	97.3201	
0.001010192	2.1489	105.1213	
0.001518466	2.2146	112.7015	
0.002262674	2.2850	119.9454	
0.003345552	2.3611	126.9069	
0.004813362	2.4391	133.3870	
0.006971538	2.5271	139.6279	
0.009202873	2.5997	145.2675	
0.011729800	2.6685	150.4726	

pharmaceutics

MDPI

t-Plot Report - Data

Relative Pressure (P/Po)	Statistical Thickness (Å)	Quantity Adsorbed (cm³/g STP)	Fitted
0.029285420	2.9876	180.8872	
0.065299574	3.3876	214.9600	
0.078115104	3.5012	221.7687	*
0.097082779	3.6557	236.8215	*
0.121872144	3.8413	245.3698	*
0.143596729	3.9943	247.8334	*
0.164029183	4.1328	249.4710	*
0.181283093	4.2470	250.6439	*
0.200610839	4.3728	251.6882	*
0.250191350	4.6911	253.7322	*
0.300651680	5.0164	255.1703	
0.351507954	5.3539	256.1876	
0.400532818	5.6949	256.8561	
0.450326101	6.0638	257.3184	
0.500397878	6.4653	257.6590	
0.550599114	6.9080	257.7877	
0.619669861	7.6058	257.8410	
0.669675390	8.1985	257.8909	
0.719694743	8.8941	257.9243	

.

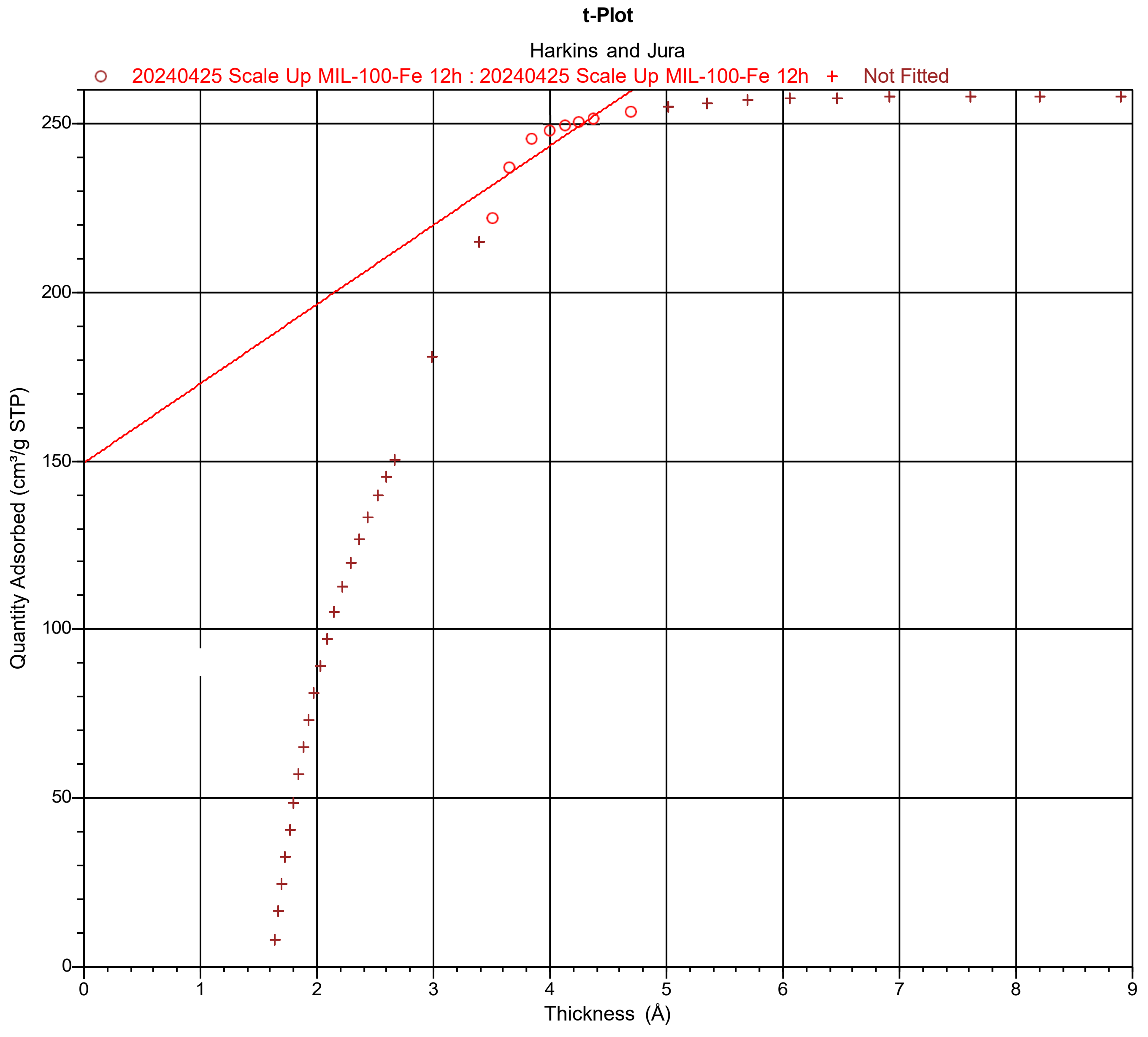

t-Plot
Harkins and Jura
20240425 Scale Up MIL-100-Fe 12h : 20240425 Scale Up MIL-100-Fe 12h
Not Fitted
Quantity Adsorbed (cm³/g STP)
Thickness (Å)
0
50
100
150
200
250
1
2
3
4
5
6
7
8
9

BJH Adsorption Pore Distribution Report

Faas Correction
Halsey
t = 3.54 [-5 / ln(P/Po)] ^ 0.333

Width range: 17.000 Å to 3,000.000 Å
Adsorbate property factor: 9.53000 Å
Density conversion factor: 0.0015468
Fraction of pores open at both ends: 0.00

Pore Width Range (Å)	Average Width (Å)	Incremental Pore Volume (cm^3/g)	Cumulative Pore Volume (cm^3/g)	Incremental Pore Area (m^2/g)	Cumulative Pore Area (m^2/g)
3589.2 - 2039.2	2413.1	0.005403	0.005403	0.090	0.090
2039.2 - 1006.6	1204.3	0.005527	0.010930	0.184	0.273
1006.6 - 781.5	865.4	0.002572	0.013502	0.119	0.392
781.5 - 400.4	476.5	0.004294	0.017796	0.360	0.752
400.4 - 270.9	310.4	0.001985	0.019780	0.256	1.008
270.9 - 204.7	228.2	0.001002	0.020782	0.176	1.184
204.7 - 193.3	198.6	0.000358	0.021140	0.072	1.256
193.3 - 137.6	155.7	0.000664	0.021804	0.171	1.426
137.6 - 114.7	123.9	0.000214	0.022018	0.069	1.496
114.7 - 114.1	114.4	0.000207	0.022225	0.072	1.568
114.1 - 89.3	98.4	0.000187	0.022412	0.076	1.644
89.3 - 41.2	43.4	0.000302	0.022714	0.278	1.922
41.2 - 36.9	38.8	0.001064	0.023778	1.097	3.019
36.9 - 33.3	34.9	0.001548	0.025326	1.776	4.795
33.3 - 30.2	31.5	0.002410	0.027736	3.058	7.853
30.2 - 27.2	28.5	0.003930	0.031666	5.516	13.370
27.2 - 24.6	25.7	0.005924	0.037590	9.203	22.573
24.6 - 22.2	23.2	0.008999	0.046590	15.494	38.066
22.2 - 21.3	21.7	0.004801	0.051391	8.843	46.909
21.3 - 20.5	20.9	0.005615	0.057006	10.767	57.677
20.5 - 19.5	20.0	0.008051	0.065057	16.127	73.804

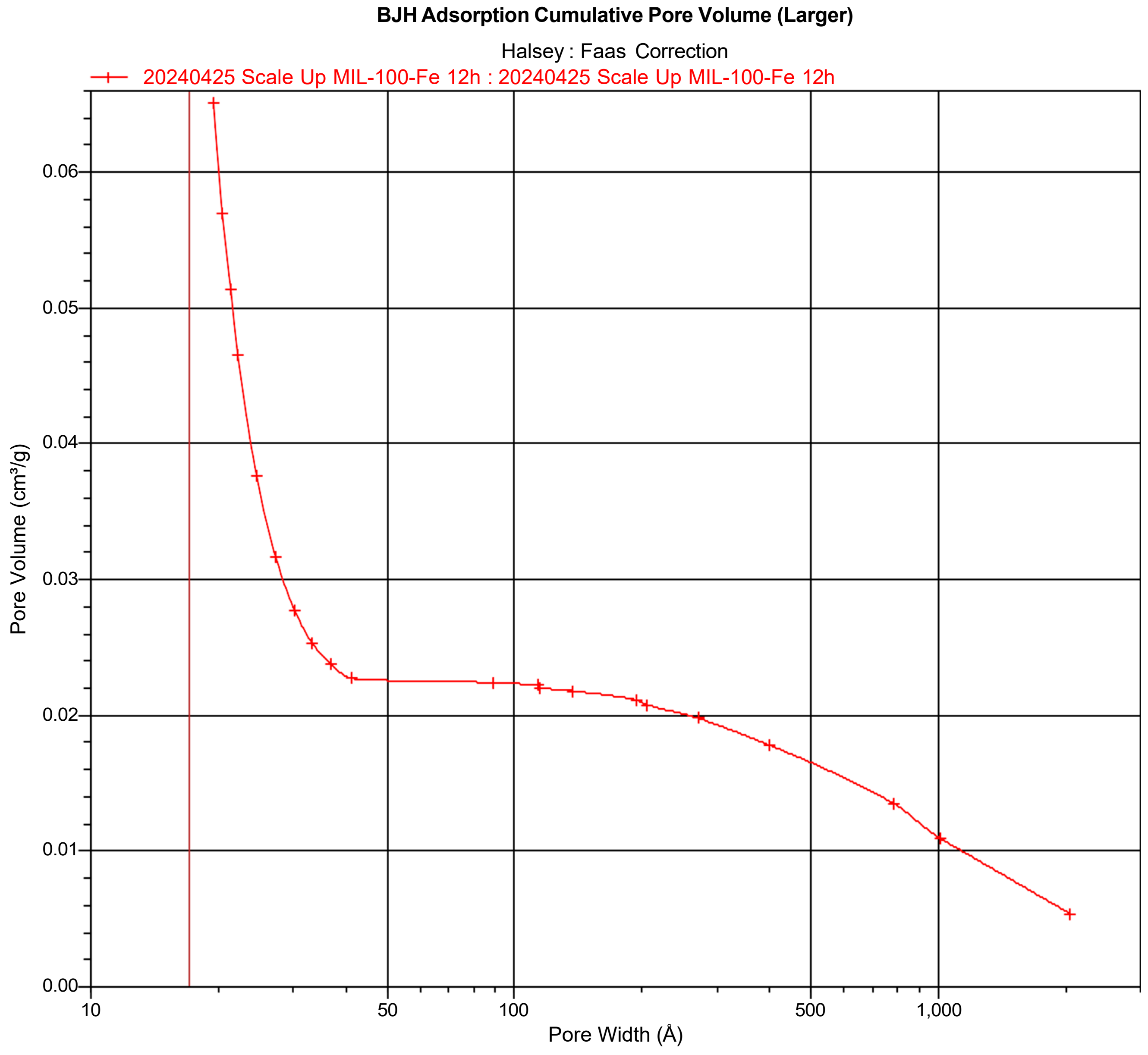

BJH Adsorption Cumulative Pore Volume (Larger)
Halsey : Faas Correction
20240425 Scale Up MIL-100-Fe 12h : 20240425 Scale Up MIL-100-Fe 12h
Pore Volume (cm³/g)
0.06
0.05
0.04
0.03
0.02
0.01
0.00
10
50
100
500
1,000
Pore Width (Å)

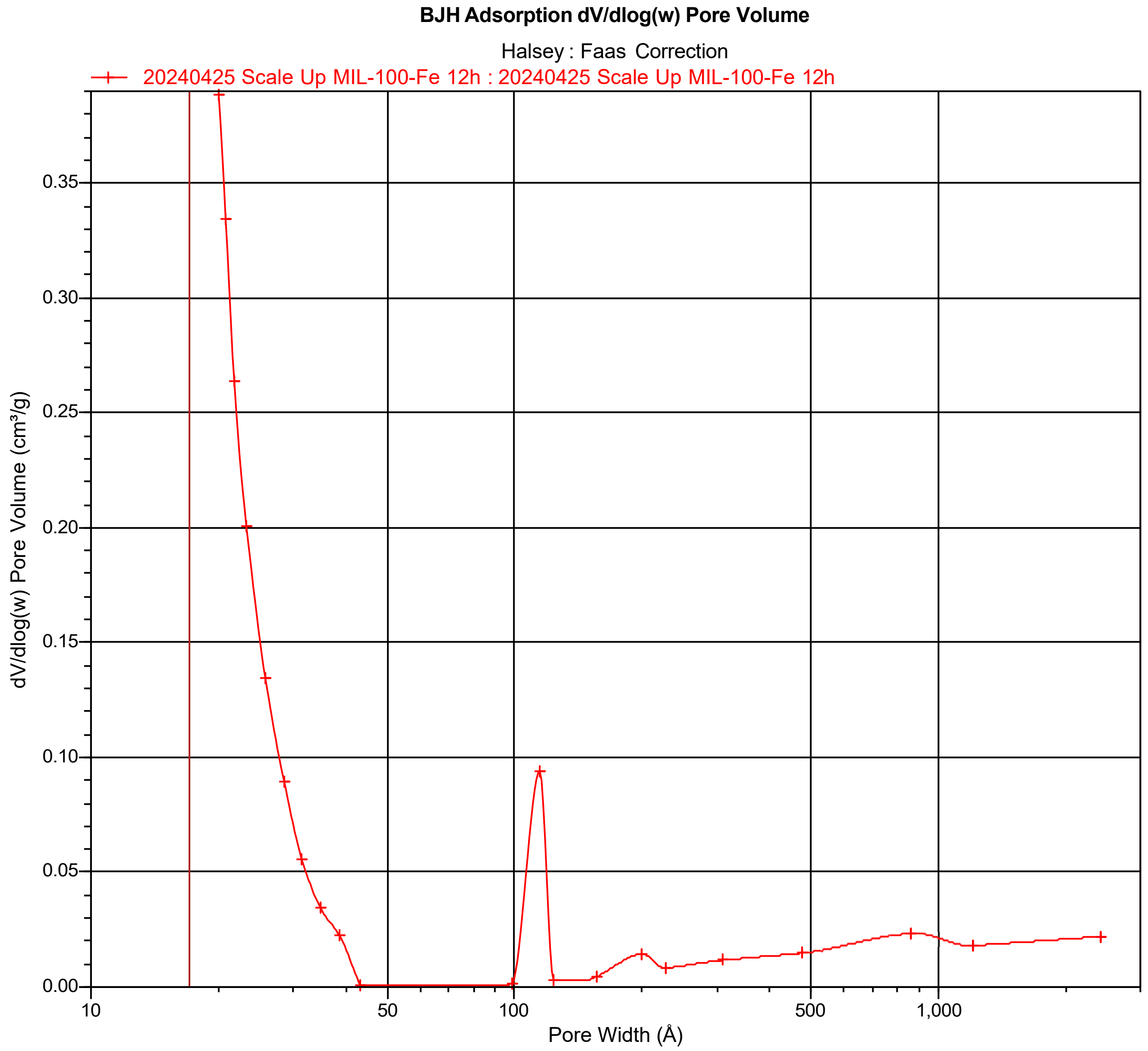
BJH Adsorption dV/dlog(w) Pore Volume
Halsey : Faas Correction
20240425 Scale Up MIL-100-Fe 12h : 20240425 Scale Up MIL-100-Fe 12h
dV/dlog(w) Pore Volume (cm³/g)
0.35
0.30
0.25
0.20
0.15
0.10
0.05
0.00
10
50
100
500
1,000
Pore Width (Å)

pharmaceutics

MDPI

BJH Desorption Pore Distribution Report

Faas Correction
Halsey
t = 3.54 [-5 / ln(P/Po)] ^ 0.333

Width range: 17.000 Å to 3,000.000 Å
Adsorbate property factor: 9.53000 Å
Density conversion factor: 0.0015468
Fraction of pores open at both ends: 0.00

Pore Width Range (Å)	Average Width (Å)	Incremental Pore Volume (cm³/g)	Cumulative Pore Volume (cm³/g)	Incremental Pore Area (m²/g)	Cumulative Pore Area (m²/g)
6330.4 - 872.8	969.1	0.018854	0.018854	0.778	0.778
872.8 - 484.1	573.0	0.007146	0.026000	0.499	1.277
484.1 - 299.4	348.9	0.003761	0.029761	0.431	1.708
299.4 - 218.0	245.5	0.001583	0.031343	0.258	1.966
218.0 - 171.8	189.2	0.000838	0.032182	0.177	2.143
171.8 - 142.2	154.0	0.000380	0.032562	0.099	2.242
142.2 - 121.0	129.7	0.000163	0.032725	0.050	2.292
121.0 - 105.4	112.0	0.000157	0.032882	0.056	2.348
105.4 - 93.1	98.4	0.000012	0.032893	0.005	2.353
93.1 - 44.2	46.7	0.000149	0.033042	0.128	2.481
44.2 - 39.5	41.5	0.000607	0.033650	0.585	3.066
39.5 - 36.9	38.1	0.000895	0.034544	0.939	4.005
36.9 - 33.2	34.8	0.001611	0.036155	1.849	5.855
33.2 - 30.1	31.4	0.002419	0.038574	3.077	8.932
30.1 - 27.2	28.4	0.003883	0.042457	5.460	14.392
27.2 - 24.6	25.7	0.005956	0.048413	9.260	23.651
24.6 - 22.2	23.2	0.009127	0.057540	15.726	39.377
22.2 - 19.4	20.5	0.019764	0.077304	38.531	77.909

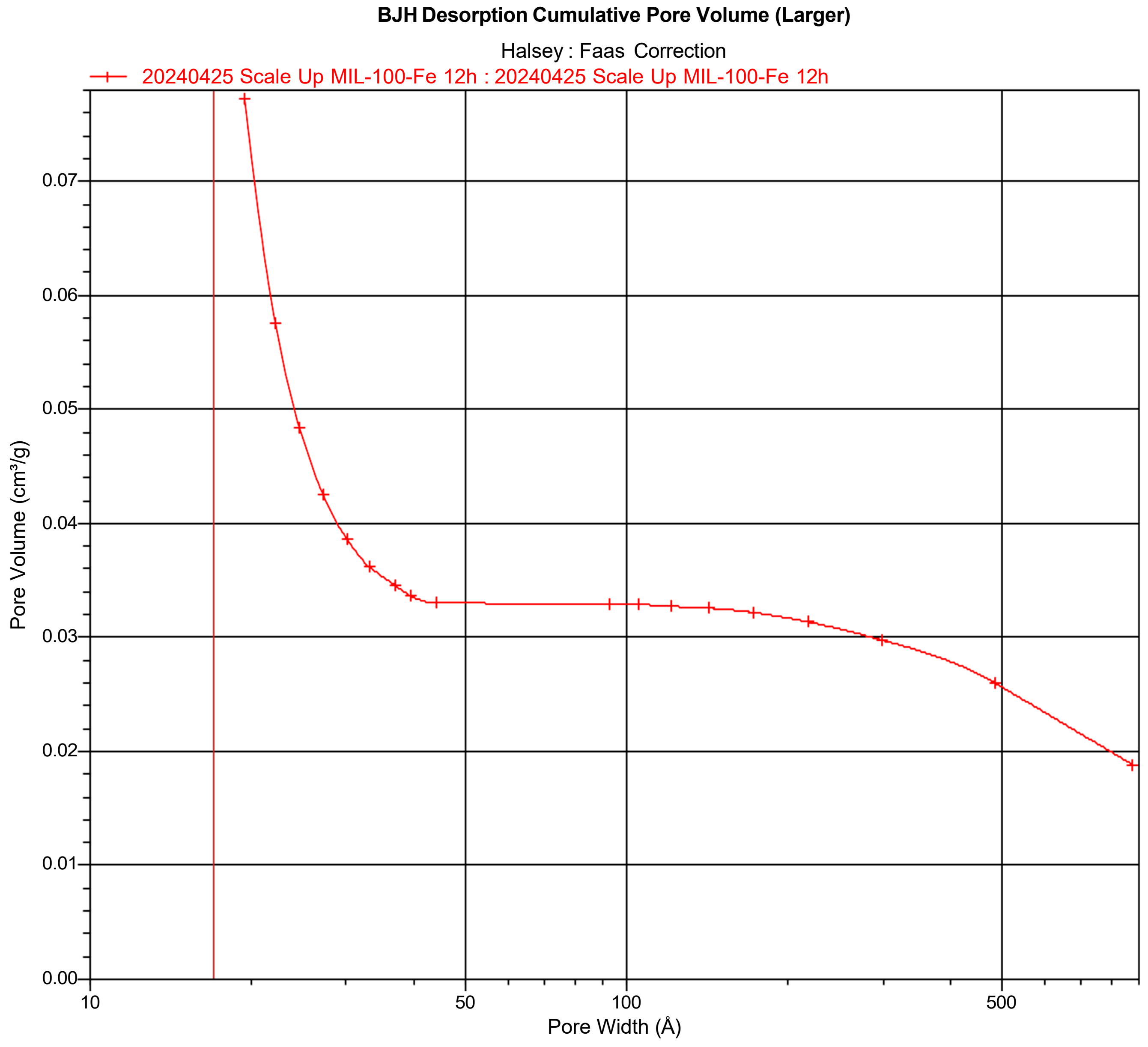

BJH Desorption Cumulative Pore Volume (Larger)
Halsey : Faas Correction
20240425 Scale Up MIL-100-Fe 12h : 20240425 Scale Up MIL-100-Fe 12h
Pore Volume (cm³/g)
0.07
0.06
0.05
0.04
0.03
0.02
0.01
0.00
10
50
100
500
Pore Width (Å)

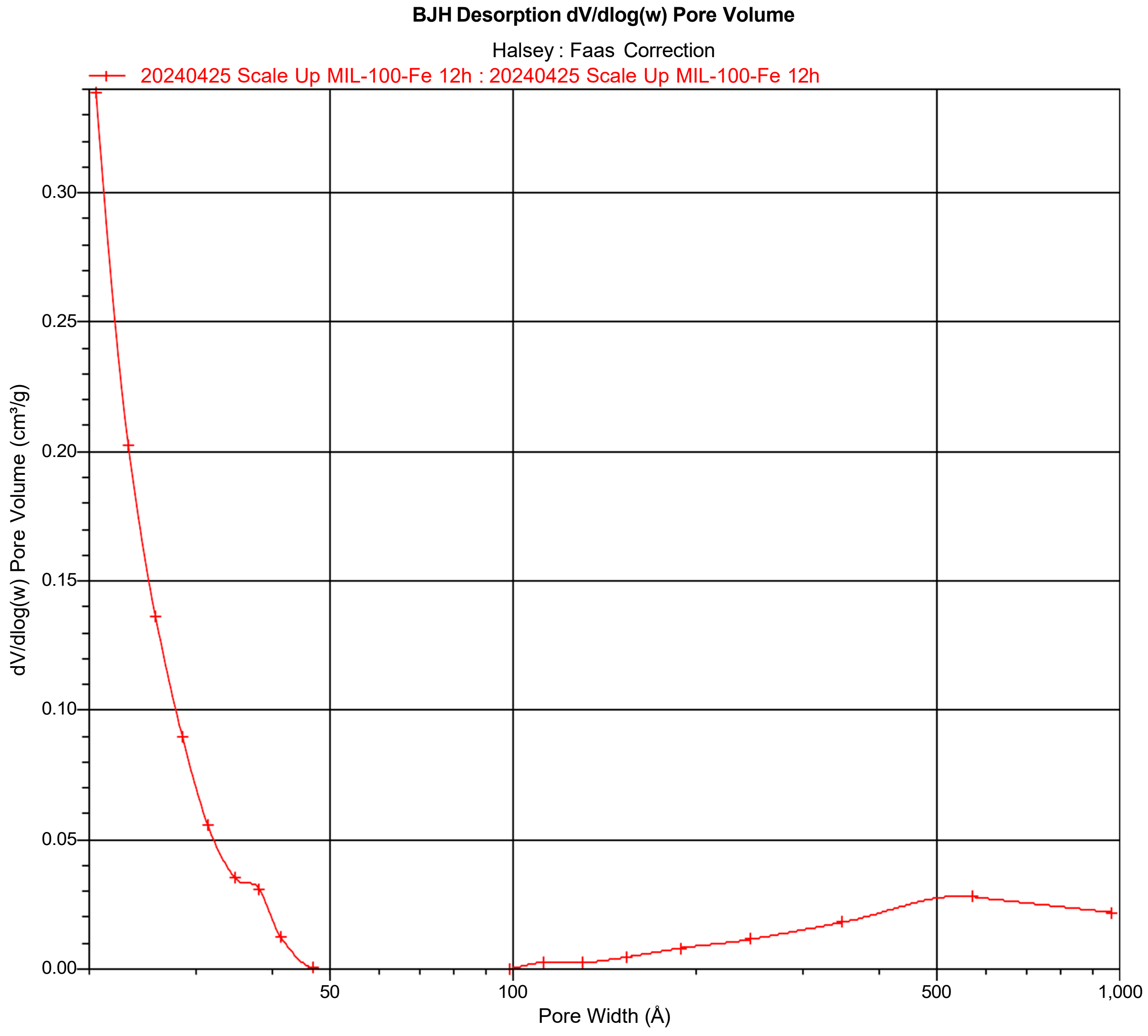
BJH Desorption dV/dlog(w) Pore Volume
Halsey : Faas Correction
20240425 Scale Up MIL-100-Fe 12h : 20240425 Scale Up MIL-100-Fe 12h
dV/dlog(w) Pore Volume (cm³/g)
0.30
0.25
0.20
0.15
0.10
0.05
0.00
50
100
500
1,000
Pore Width (Å)

pharmaceutics

MDPI

Porosity Distribution by
Model: N2 - Tarazona NLDFT, Esf = 30.0K
Method: Non-negative Regularization: 0.01000
Standard Deviation of Fit: 3.34606 cm³/g STP

Volume in Pores	<	5.22 Å	:	0.00094 cm³/g
Total Volume in Pores	<=	448.83 Å	:	0.42034 cm³/g
Total Area in Pores	>=	5.22 Å	:	1,702.738 m²/g

Pore Size Table

Pore Width (Å)	Cumulative Volume (cm³/g)	Incremental Volume (cm³/g)	Cumulative Area (m²/g)	Incremental Area (m²/g)
5.22	0.00776	0.00682	52.280	52.280
5.93	0.02735	0.01959	184.326	132.046
6.29	0.06143	0.03408	401.020	216.694
6.65	0.10512	0.04369	663.856	262.837
7.36	0.14933	0.04421	903.989	240.133
7.72	0.18113	0.03180	1068.757	164.768
8.44	0.19921	0.01808	1154.483	85.726
8.79	0.20948	0.01027	1201.178	46.695
9.51	0.22024	0.01076	1246.438	45.260
10.22	0.23252	0.01228	1294.483	48.045
10.94	0.24321	0.01069	1333.591	39.108
11.65	0.24920	0.00599	1354.146	20.556
12.37	0.24920	0.00000	1354.146	0.000
13.08	0.24920	0.00000	1354.146	0.000
13.80	0.24920	0.00000	1354.146	0.000
14.87	0.24920	0.00000	1354.146	0.000
15.59	0.25060	0.00140	1357.748	3.602
16.66	0.26381	0.01321	1389.463	31.714
17.73	0.29143	0.02762	1451.775	62.312
18.44	0.33033	0.03890	1536.134	84.359
19.87	0.36577	0.03544	1607.469	71.335
20.95	0.39371	0.02794	1660.823	53.354
22.02	0.41161	0.01790	1693.339	32.516
23.45	0.41695	0.00533	1702.435	9.096
24.52	0.41695	0.00000	1702.435	0.000
25.95	0.41695	0.00000	1702.435	0.000
27.38	0.41695	0.00000	1702.435	0.000

pharmaceutics

MDPI

Pore Size Table

Pore Width (Å)	Cumulative Volume (cm³/g)	Incremental Volume (cm³/g)	Cumulative Area (m²/g)	Incremental Area (m²/g)
29.17	0.41695	0.00000	1702.435	0.000
30.60	0.41695	0.00000	1702.435	0.000
32.39	0.41695	0.00000	1702.435	0.000
34.17	0.41695	0.00000	1702.435	0.000
35.96	0.41695	0.00000	1702.435	0.000
38.11	0.41695	0.00000	1702.435	0.000
40.25	0.41695	0.00000	1702.435	0.000
42.39	0.41695	0.00000	1702.435	0.000
44.54	0.41695	0.00000	1702.435	0.000
47.04	0.41695	0.00000	1702.435	0.000
49.54	0.41695	0.00000	1702.435	0.000
52.05	0.41695	0.00000	1702.435	0.000
54.91	0.41695	0.00000	1702.435	0.000
57.77	0.41695	0.00000	1702.435	0.000
60.98	0.41695	0.00000	1702.435	0.000
64.20	0.41695	0.00000	1702.435	0.000
67.42	0.41695	0.00000	1702.435	0.000
70.99	0.41695	0.00000	1702.435	0.000
74.57	0.41695	0.00000	1702.435	0.000
78.50	0.41695	0.00000	1702.435	0.000
82.79	0.41695	0.00000	1702.435	0.000
87.08	0.41695	0.00000	1702.435	0.000
91.37	0.41695	0.00000	1702.435	0.000
96.37	0.41695	0.00000	1702.435	0.000
101.38	0.41695	0.00000	1702.435	0.000
106.38	0.41695	0.00000	1702.435	0.000
112.10	0.41695	0.00000	1702.435	0.000
117.82	0.41695	0.00000	1702.435	0.000
123.90	0.41695	0.00000	1702.435	0.000
130.33	0.41695	0.00000	1702.435	0.000
136.76	0.41695	0.00000	1702.435	0.000
143.91	0.41695	0.00000	1702.435	0.000
151.06	0.41695	0.00000	1702.435	0.000
158.93	0.41695	0.00000	1702.435	0.000
167.15	0.41695	0.00000	1702.435	0.000
175.73	0.41695	0.00000	1702.435	0.000

pharmaceutics

MDPI

Pore Size Table

Pore Width (Å)	Cumulative Volume (cm³/g)	Incremental Volume (cm³/g)	Cumulative Area (m²/g)	Incremental Area (m²/g)
184.66	0.41695	0.00000	1702.435	0.000
193.96	0.41695	0.00000	1702.435	0.000
203.97	0.41695	0.00000	1702.435	0.000
214.33	0.41695	0.00000	1702.435	0.000
225.06	0.41695	0.00000	1702.435	0.000
236.50	0.41695	0.00000	1702.435	0.000
248.29	0.41695	0.00000	1702.435	0.000
261.16	0.41695	0.00000	1702.435	0.000
274.39	0.41695	0.00000	1702.435	0.000
287.97	0.41695	0.00000	1702.435	0.000
302.63	0.41695	0.00000	1702.435	0.000
318.00	0.41695	0.00000	1702.435	0.000
334.08	0.41695	0.00000	1702.435	0.000
350.88	0.41695	0.00000	1702.435	0.000
368.76	0.41695	0.00000	1702.435	0.000
387.34	0.41695	0.00000	1702.435	0.000
406.65	0.41695	0.00000	1702.435	0.000
427.38	0.41695	0.00000	1702.435	0.000
448.83	0.42034	0.00340	1702.738	0.303

pharmaceutics

MDPI

Porosity Distribution by
Model: N2 - Tarazona NLDFT, Esf = 30.0K
Method: Non-negative Regularization: 0.01000

Standard Deviation of Fit: 3.34606 cm³/g STP

Isotherm Table

Relative Pressure (P/Po)	Experimental Quantity Adsorbed (cm³/g STP)	Fitted Quantity Adsorbed (cm³/g STP)	Absolute Residual (cm³/g STP)	Relative Residual
0.000006310	8.2803	15.4154	-7.1351	-0.861688
0.000007943	11.8200	17.7632	-5.9431	-0.502801
0.000010000	15.7721	20.3111	-4.5390	-0.287790
0.000012589	20.0324	23.1017	-3.0693	-0.153218
0.000015849	24.5687	26.0016	-1.4329	-0.058323
0.000019953	29.3592	28.8369	0.5223	0.017789
0.000025119	34.2067	31.6682	2.5385	0.074212
0.000031623	39.1624	34.7967	4.3657	0.111478
0.000039811	43.8547	38.8474	5.0073	0.114179
0.000050119	48.5968	44.0852	4.5116	0.092838
0.000063096	53.2053	50.3312	2.8741	0.054020
0.000079433	57.7335	56.8304	0.9031	0.015642
0.000100000	62.2410	61.2640	0.9770	0.015697
0.000125892	66.4714	65.7638	0.7076	0.010646
0.000158490	70.8010	72.4146	-1.6135	-0.022790
0.000199526	74.8986	77.9545	-3.0559	-0.040800
0.000251188	79.2209	81.3459	-2.1251	-0.026825
0.000316228	83.3398	84.5013	-1.1615	-0.013937
0.000398107	87.6990	89.4671	-1.7681	-0.020161
0.000501187	91.8752	92.5314	-0.6562	-0.007142
0.000630958	96.2956	97.3076	-1.0120	-0.010509
0.000794328	100.5473	100.6236	-0.0762	-0.000758
0.001000000	104.9425	105.0214	-0.0789	-0.000752
0.001258925	109.2140	108.2756	0.9384	0.008593
0.001584895	113.4455	111.8888	1.5567	0.013722
0.001995263	117.6982	114.7156	2.9826	0.025341
0.002511882	121.7451	117.6210	4.1240	0.033874
0.003162276	125.9269	120.9534	4.9735	0.039495
0.003981066	130.0436	124.1907	5.8529	0.045007

pharmaceutics

MDPI

Isotherm Table

Relative Pressure (P/Po)	Experimental Quantity Adsorbed (cm³/g STP)	Fitted Quantity Adsorbed (cm³/g STP)	Absolute Residual (cm³/g STP)	Relative Residual
0.005011868	134.0039	128.0679	5.9359	0.044297
0.006309579	137.8352	134.0346	3.8007	0.027574
0.007943276	142.1693	144.0666	-1.8974	-0.013346
0.010000000	147.0132	154.7874	-7.7743	-0.052882
0.012355640	151.6838	163.3694	-11.6857	-0.077040
0.015186320	157.1081	168.8801	-11.7720	-0.074929
0.018485530	163.2563	172.8959	-9.6396	-0.059046
0.022294740	169.9981	176.4378	-6.4397	-0.037881
0.026653420	177.0559	179.4097	-2.3538	-0.013294
0.031598160	184.0086	184.1208	-0.1122	-0.000610
0.037162240	190.8713	186.7824	4.0890	0.021423
0.043374470	197.5556	194.0732	3.4824	0.017627
0.050259210	203.9249	204.9984	-1.0736	-0.005265
0.057835260	209.8962	207.6864	2.2097	0.010528
0.066115920	215.4618	210.1272	5.3346	0.024759
0.075109080	220.0057	220.2113	-0.2056	-0.000934
0.084815920	227.0694	228.7388	-1.6694	-0.007352
0.095232370	235.6901	230.7014	4.9887	0.021167
0.106348200	241.2482	236.9755	4.2727	0.017711
0.118147500	244.7649	239.9684	4.7965	0.019596
0.130609100	246.4768	241.3918	5.0850	0.020631
0.143706600	247.8424	242.6849	5.1575	0.020810
0.157410500	248.9689	243.8683	5.1006	0.020487
0.171685500	250.0128	244.9569	5.0559	0.020223
0.186492100	250.9521	245.9626	4.9895	0.019882
0.201792100	251.7440	246.8949	4.8491	0.019262
0.217539500	252.4601	247.7614	4.6986	0.018611
0.233689500	253.1304	248.5689	4.5615	0.018020
0.250196100	253.7323	249.3228	4.4095	0.017379
0.267011800	254.2706	250.0279	4.2427	0.016686
0.284089500	254.7591	250.6885	4.0706	0.015978
0.301380300	255.1869	251.3082	3.8787	0.015200
0.318838200	255.5729	251.8902	3.6828	0.014410
0.336417100	255.9261	252.4374	3.4887	0.013632
0.354071100	256.2279	252.9524	3.2755	0.012784

pharmaceutics

MDPI

Isotherm Table

Relative Pressure (P/Po)	Experimental Quantity Adsorbed (cm³/g STP)	Fitted Quantity Adsorbed (cm³/g STP)	Absolute Residual (cm³/g STP)	Relative Residual
0.371757900	256.4942	253.4376	3.0565	0.011917
0.389435500	256.7298	253.8951	2.8347	0.011042
0.407065800	256.9237	254.3269	2.5969	0.010107
0.424610500	257.0950	254.7346	2.3604	0.009181
0.442034200	257.2500	255.1201	2.1299	0.008280
0.459305300	257.3901	255.4850	1.9051	0.007402
0.476393400	257.5190	255.8311	1.6879	0.006555
0.493271100	257.6245	256.1620	1.4625	0.005677
0.509911800	257.6970	256.4814	1.2156	0.004717
0.526293400	257.7471	256.7779	0.9693	0.003761
0.542394700	257.7784	257.0568	0.7216	0.002799
0.558200000	257.7939	257.3205	0.4735	0.001837
0.573690800	257.8055	257.5700	0.2354	0.000913
0.588853900	257.8161	257.8064	0.0097	0.000038
0.603677600	257.8271	258.0305	-0.2034	-0.000789
0.618153900	257.8396	258.2431	-0.4035	-0.001565
0.632272400	257.8535	258.4447	-0.5912	-0.002293
0.646028900	257.8675	258.6362	-0.7687	-0.002981
0.659417100	257.8810	258.8179	-0.9370	-0.003633
0.672435500	257.8934	258.9906	-1.0972	-0.004254
0.685081600	257.9035	259.1548	-1.2513	-0.004852
0.697355300	257.9115	259.3109	-1.3994	-0.005426
0.709256600	257.9184	259.4594	-1.5411	-0.005975
0.720789500	257.9243	259.6010	-1.6767	-0.006501
0.731953900	257.9243	259.7363	-1.8120	-0.007025
0.742756600	257.9204	259.8662	-1.9459	-0.007544
0.753200000	257.9123	259.9972	-2.0849	-0.008084
0.763289500	257.9046	260.1232	-2.2185	-0.008602
0.773030300	257.9011	260.2363	-2.3352	-0.009055
0.782430300	257.9086	260.3427	-2.4341	-0.009438
0.791496100	257.9297	260.4434	-2.5138	-0.009746
0.800232900	257.9623	260.5390	-2.5768	-0.009989
0.808648700	258.0050	260.6298	-2.6248	-0.010173
0.816752600	258.0567	260.7162	-2.6596	-0.010306
0.824552600	258.2077	260.7985	-2.5909	-0.010034

pharmaceutics

MDPI

Isotherm Table

Relative Pressure (P/Po)	Experimental Quantity Adsorbed (cm³/g STP)	Fitted Quantity Adsorbed (cm³/g STP)	Absolute Residual (cm³/g STP)	Relative Residual
0.832053900	258.2467	260.8770	-2.6302	-0.010185
0.839267100	258.2729	260.9518	-2.6789	-0.010372
0.846200000	258.3066	261.0234	-2.7168	-0.010518
0.852860500	258.3613	261.0921	-2.7308	-0.010570
0.859257900	258.4137	261.1586	-2.7449	-0.010622
0.865398700	258.4604	261.2263	-2.7659	-0.010701
0.871292100	258.5068	261.3021	-2.7953	-0.010813
0.876947400	258.5571	261.3618	-2.8048	-0.010848
0.882369700	258.6144	261.4172	-2.8028	-0.010838
0.887569700	258.6812	261.4695	-2.7883	-0.010779
0.892553900	258.7590	261.5190	-2.7600	-0.010666
0.897328900	258.8843	261.5662	-2.6819	-0.010360
0.901905300	259.0382	261.6114	-2.5732	-0.009934
0.906286800	259.1525	261.6546	-2.5021	-0.009655
0.910484200	259.2523	261.6964	-2.4442	-0.009428
0.914501300	259.3441	261.7375	-2.3934	-0.009229
0.918347400	259.4332	261.7950	-2.3618	-0.009104
0.922026300	259.5236	261.8395	-2.3159	-0.008924
0.925547400	259.6186	261.8765	-2.2579	-0.008697
0.928915800	259.7309	261.9108	-2.1799	-0.008393
0.932136800	259.8620	261.9434	-2.0815	-0.008010
0.935218400	260.0048	261.9749	-1.9700	-0.007577
0.938163200	260.1538	263.8611	-3.7074	-0.014251
0.940978900	260.3043	263.8862	-3.5819	-0.013760
0.943669700	260.4529	263.9101	-3.4572	-0.013274
0.946242100	260.5969	263.9329	-3.3360	-0.012801
0.948700000	260.7340	263.9545	-3.2205	-0.012352
0.951048700	260.8648	263.9752	-3.1104	-0.011923
0.953292100	260.9990	263.9948	-2.9959	-0.011478
0.955435500	261.1396	264.0136	-2.8739	-0.011005
0.957482900	261.2877	264.0314	-2.7438	-0.010501
0.959438200	261.4437	264.0484	-2.6047	-0.009963
0.961305300	261.6077	264.0646	-2.4569	-0.009391
0.963088200	261.7798	264.0801	-2.3003	-0.008787
0.964789500	261.9593	264.0947	-2.1354	-0.008152

pharmaceutics

MDPI

Isotherm Table

Relative Pressure (P/Po)	Experimental Quantity Adsorbed (cm³/g STP)	Fitted Quantity Adsorbed (cm³/g STP)	Absolute Residual (cm³/g STP)	Relative Residual
0.966414500	262.1460	264.1088	-1.9628	-0.007487
0.967965800	262.3391	264.1221	-1.7831	-0.006797
0.969447400	262.5380	264.1348	-1.5969	-0.006083
0.970860500	262.7416	264.1470	-1.4053	-0.005349
0.972209200	262.9494	264.1585	-1.2091	-0.004598
0.973496100	263.1605	264.1695	-1.0090	-0.003834
0.974725000	263.3743	264.1800	-0.8057	-0.003059
0.975897400	263.6231	264.1900	-0.5669	-0.002150
0.977015800	263.9141	264.1995	-0.2854	-0.001081
0.978082900	264.2229	264.2085	0.0143	0.000054
0.979101300	264.5306	264.2172	0.3134	0.001185
0.980072400	264.8225	264.2254	0.5971	0.002255
0.980998700	265.0942	264.2333	0.8610	0.003248
0.981882900	265.3643	264.2407	1.1235	0.004234
0.982726300	265.6321	264.2479	1.3842	0.005211
0.983530300	265.8955	264.2546	1.6409	0.006171
0.984297400	266.1532	264.2611	1.8921	0.007109
0.985028900	266.4039	264.2673	2.1366	0.008020
0.985727600	266.6470	264.2731	2.3738	0.008902
0.986392100	266.8809	264.2787	2.6022	0.009750
0.987027600	267.1064	264.2841	2.8224	0.010566
0.987632900	267.3225	264.2891	3.0334	0.011347
0.988209200	267.5288	264.2940	3.2348	0.012092
0.988760500	267.7265	264.2986	3.4278	0.012804
0.989285500	267.9145	264.3030	3.6115	0.013480
0.989785500	268.0932	264.3072	3.7860	0.014122
0.990263200	268.2633	264.3112	3.9521	0.014732
0.990718400	268.4056	264.3150	4.0906	0.015240
0.991151300	268.4720	264.3186	4.1534	0.015471
0.991565800	268.5070	264.3221	4.1849	0.015586
0.991959200	268.5509	264.3254	4.2256	0.015735

pharmaceutics

MDPI

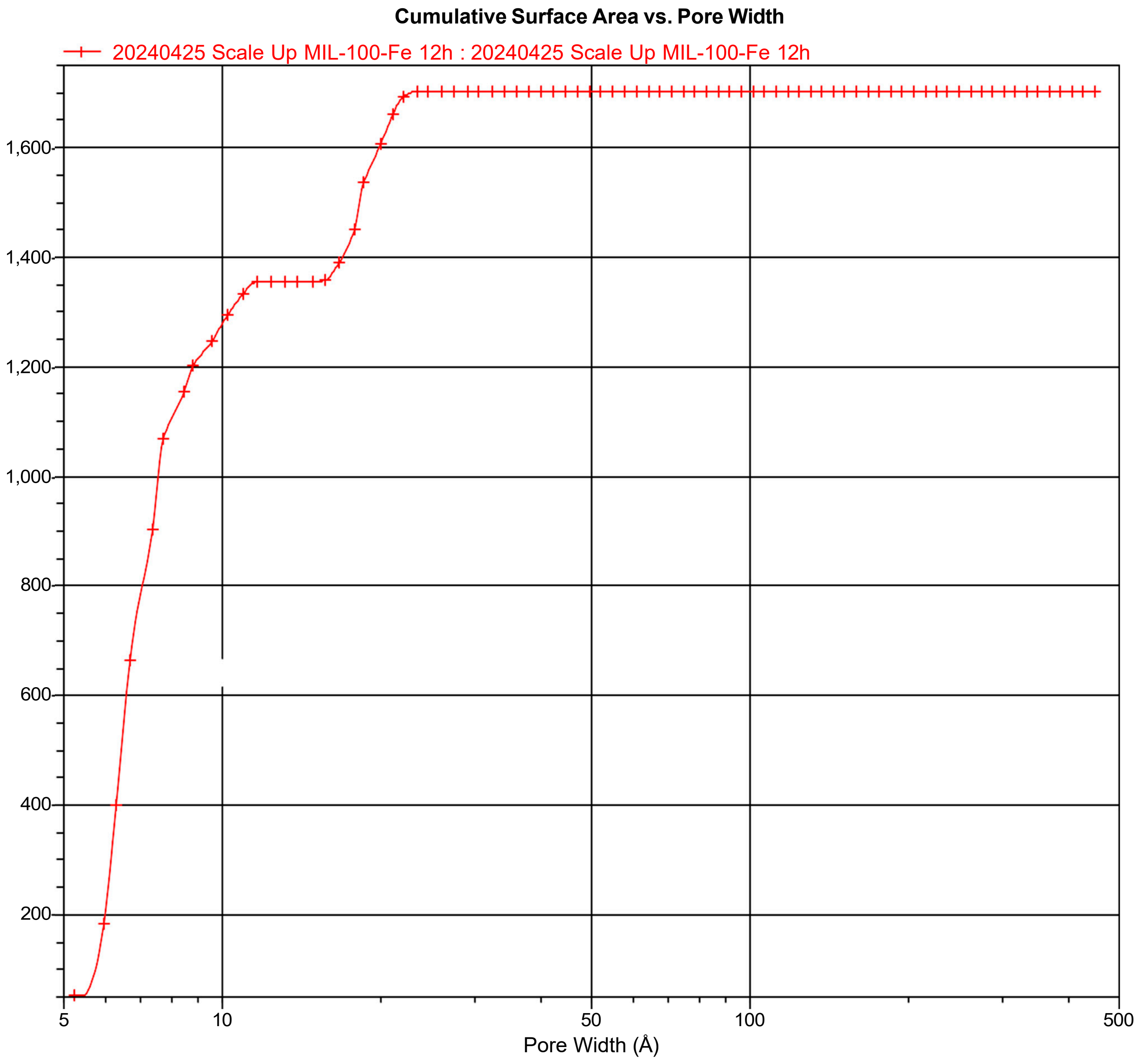

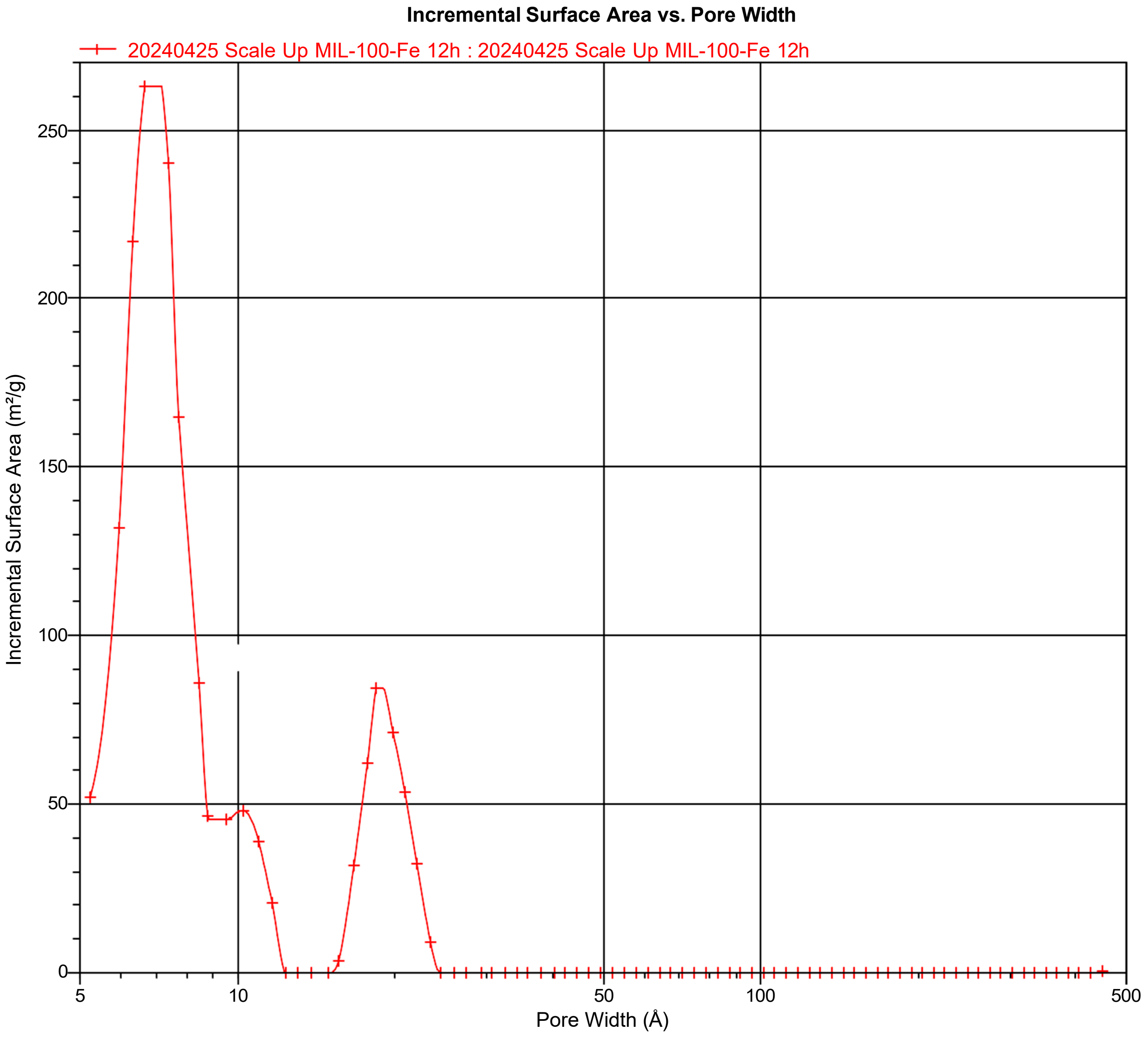
Incremental Surface Area vs. Pore Width
20240425 Scale Up MIL-100-Fe 12h : 20240425 Scale Up MIL-100-Fe 12h
Incremental Surface Area (m²/g)
250
200
150
100
50
0
5
10
50
100
500
Pore Width (Å)

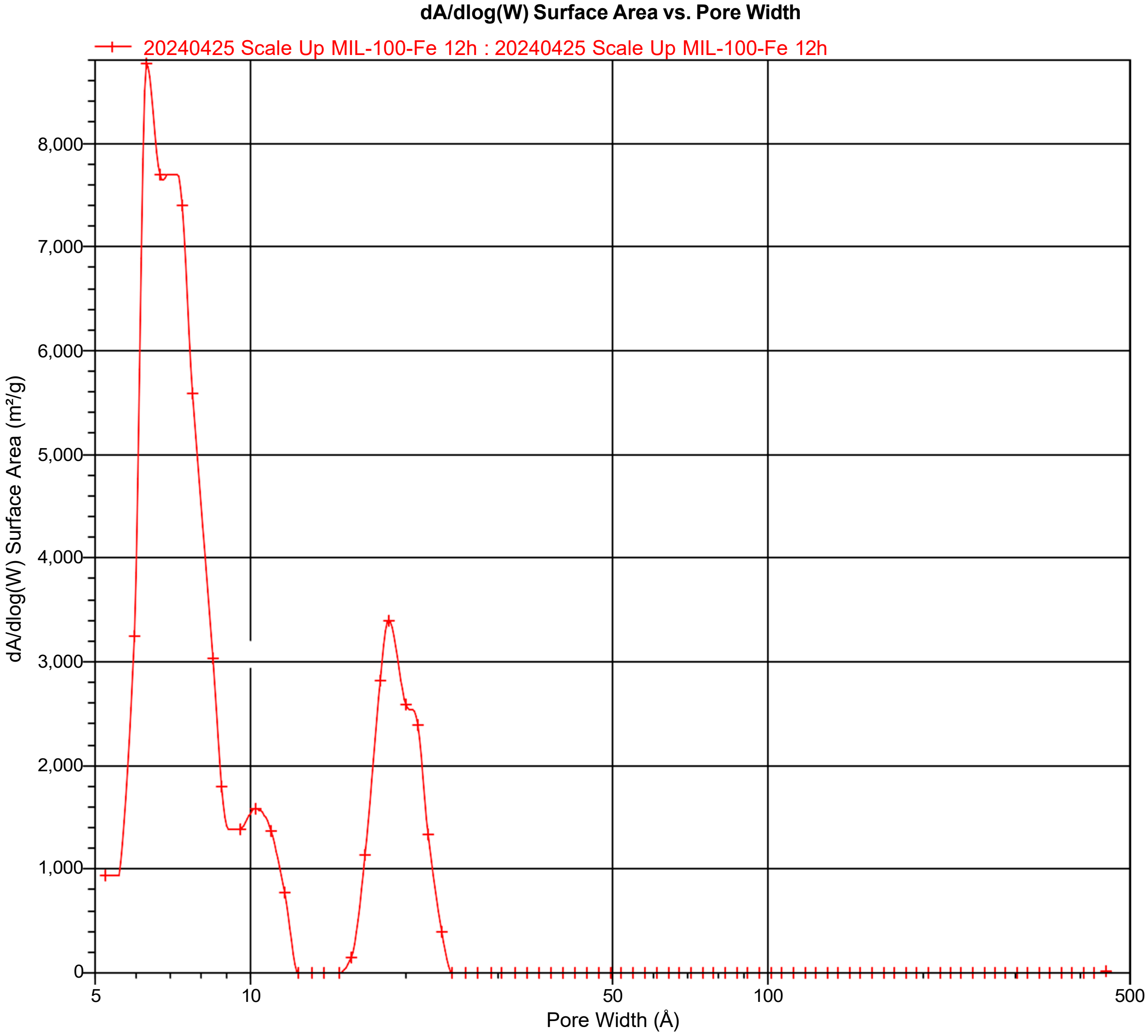
dA/dlog(W) Surface Area vs. Pore Width
20240425 Scale Up MIL-100-Fe 12h : 20240425 Scale Up MIL-100-Fe 12h
dA/dlog(W) Surface Area (m²/g)
8,000
7,000
6,000
5,000
4,000
3,000
2,000
1,000
0
5
10
50
100
500
Pore Width (Å)

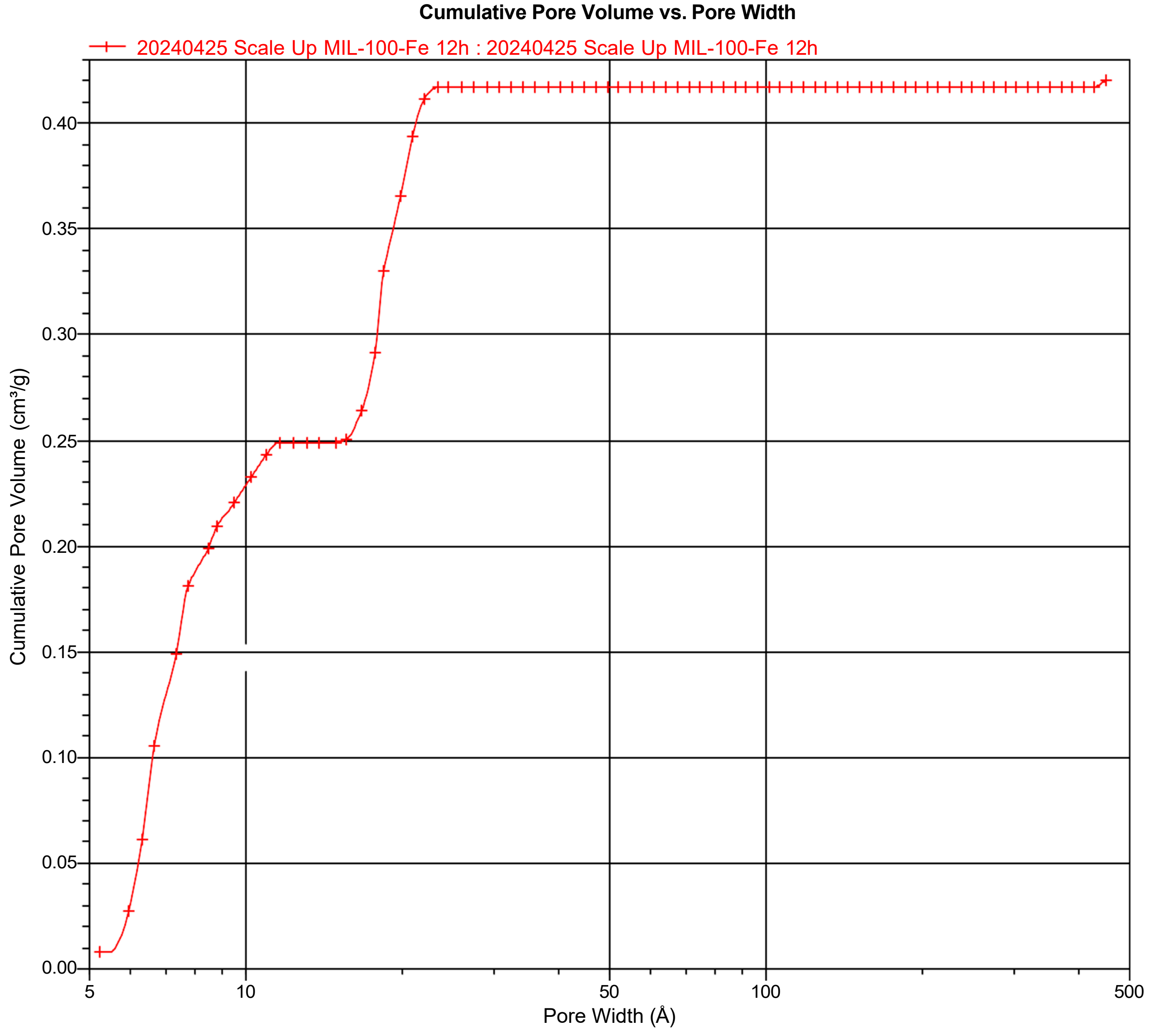
Cumulative Pore Volume vs. Pore Width
20240425 Scale Up MIL-100-Fe 12h : 20240425 Scale Up MIL-100-Fe 12h
Cumulative Pore Volume (cm³/g)
0.00
0.05
0.10
0.15
0.20
0.25
0.30
0.35
0.40
5
10
50
100
500
Pore Width (Å)

.

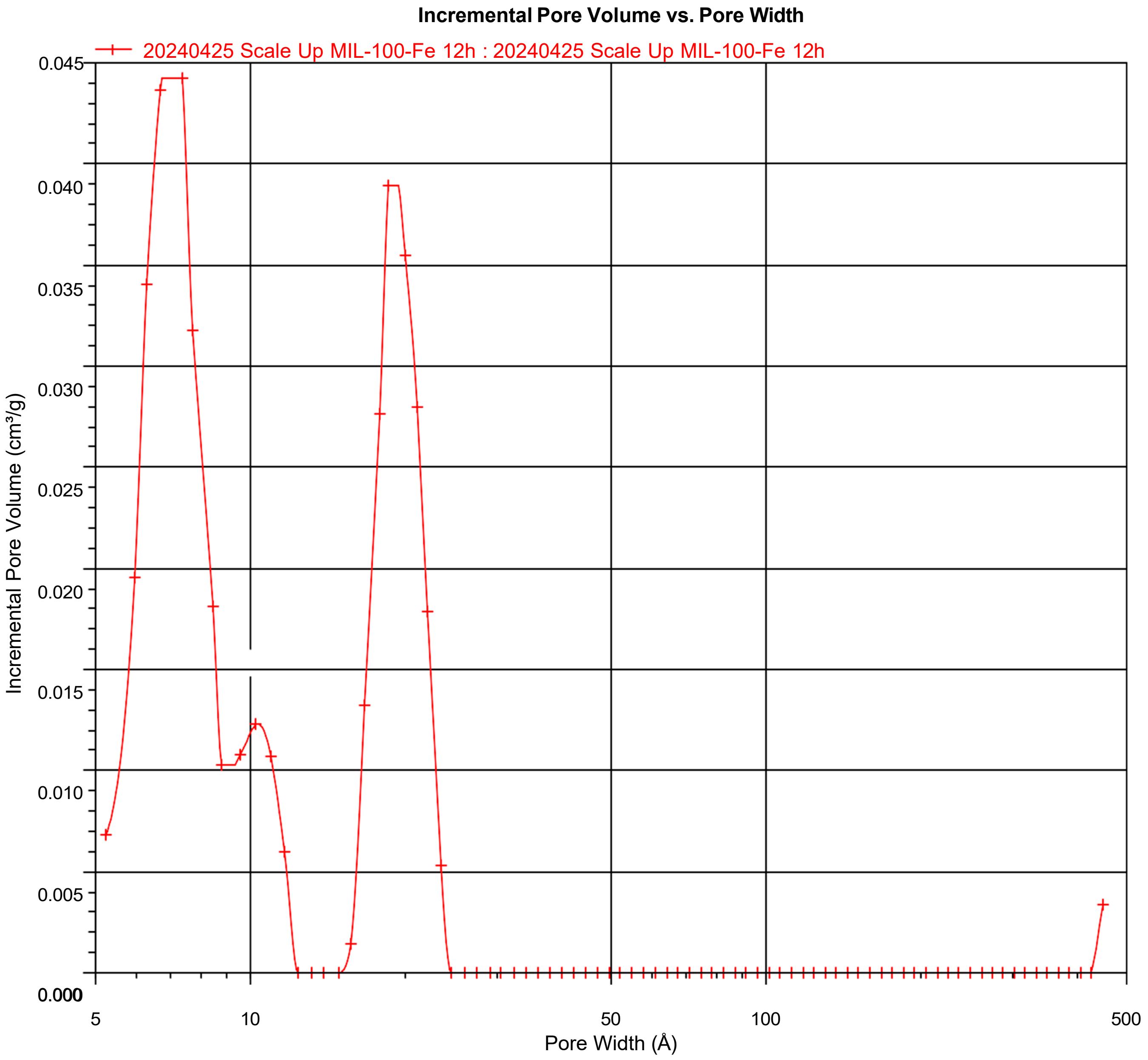
Incremental Pore Volume vs. Pore Width
20240425 Scale Up MIL-100-Fe 12h : 20240425 Scale Up MIL-100-Fe 12h
Incremental Pore Volume (cm³/g)
0.045
0.040
0.035
0.030
0.025
0.020
0.015
0.010
0.005
0.000
5
10
50
100
500
Pore Width (Å)

pharmaceutics
MDPI

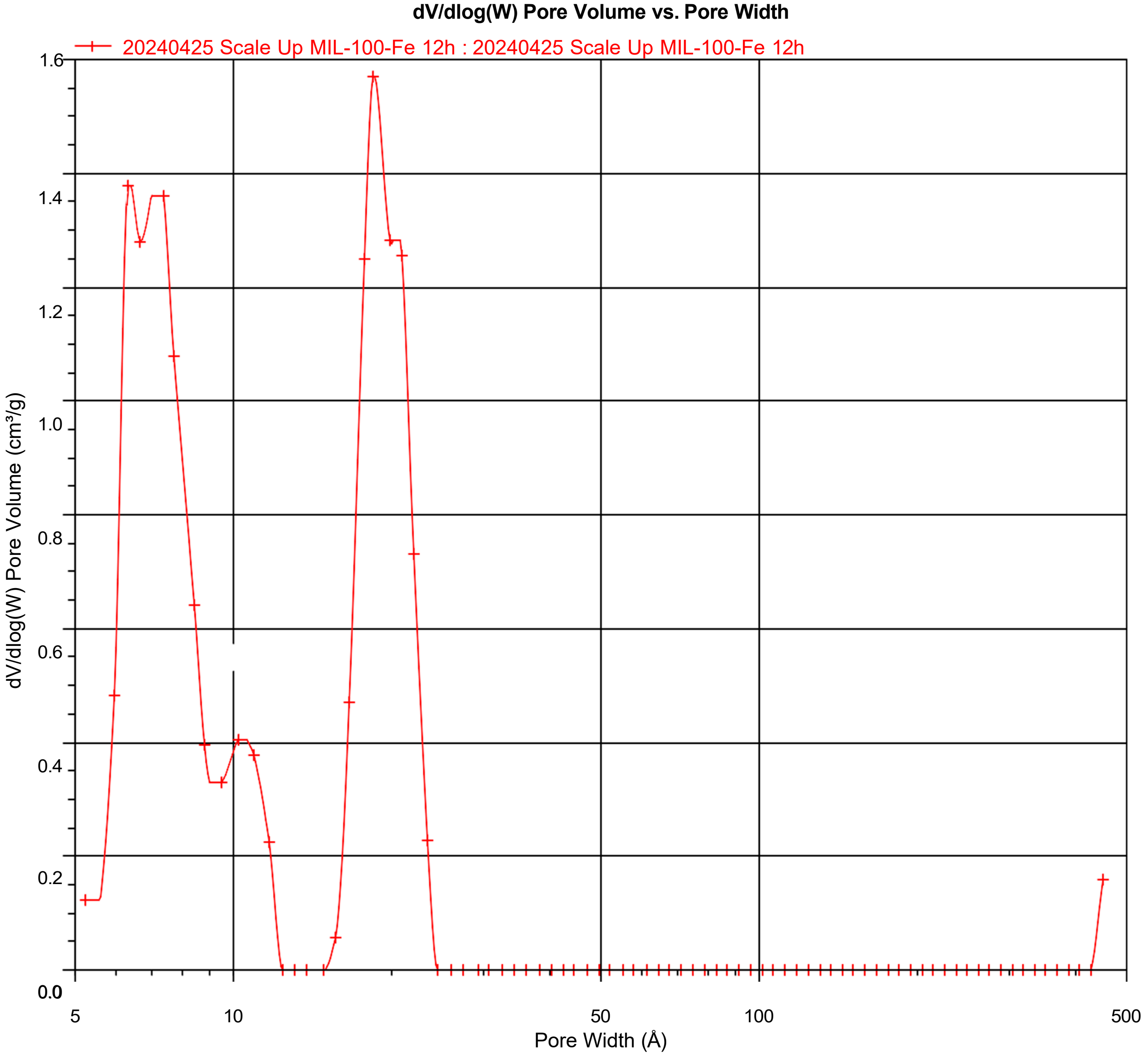

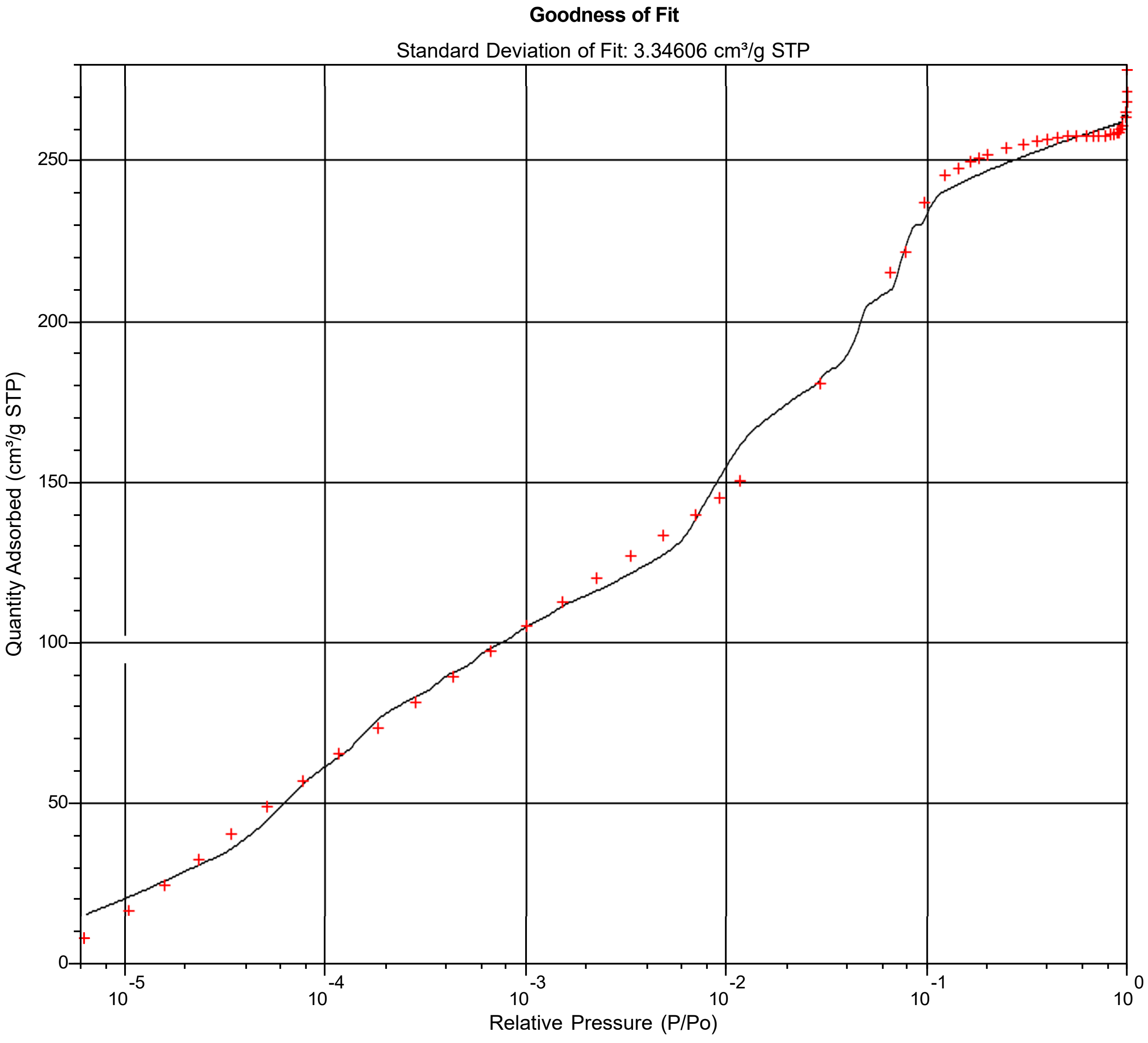
Goodness of Fit
Standard Deviation of Fit: 3.34606 cm³/g STP
Quantity Adsorbed (cm³/g STP)
Relative Pressure (P/Po)
0
50
100
150
200
250
10^-5
10^-4
10^-3
10^-2
10^-1
10^0

pharmaceutics

MDPI

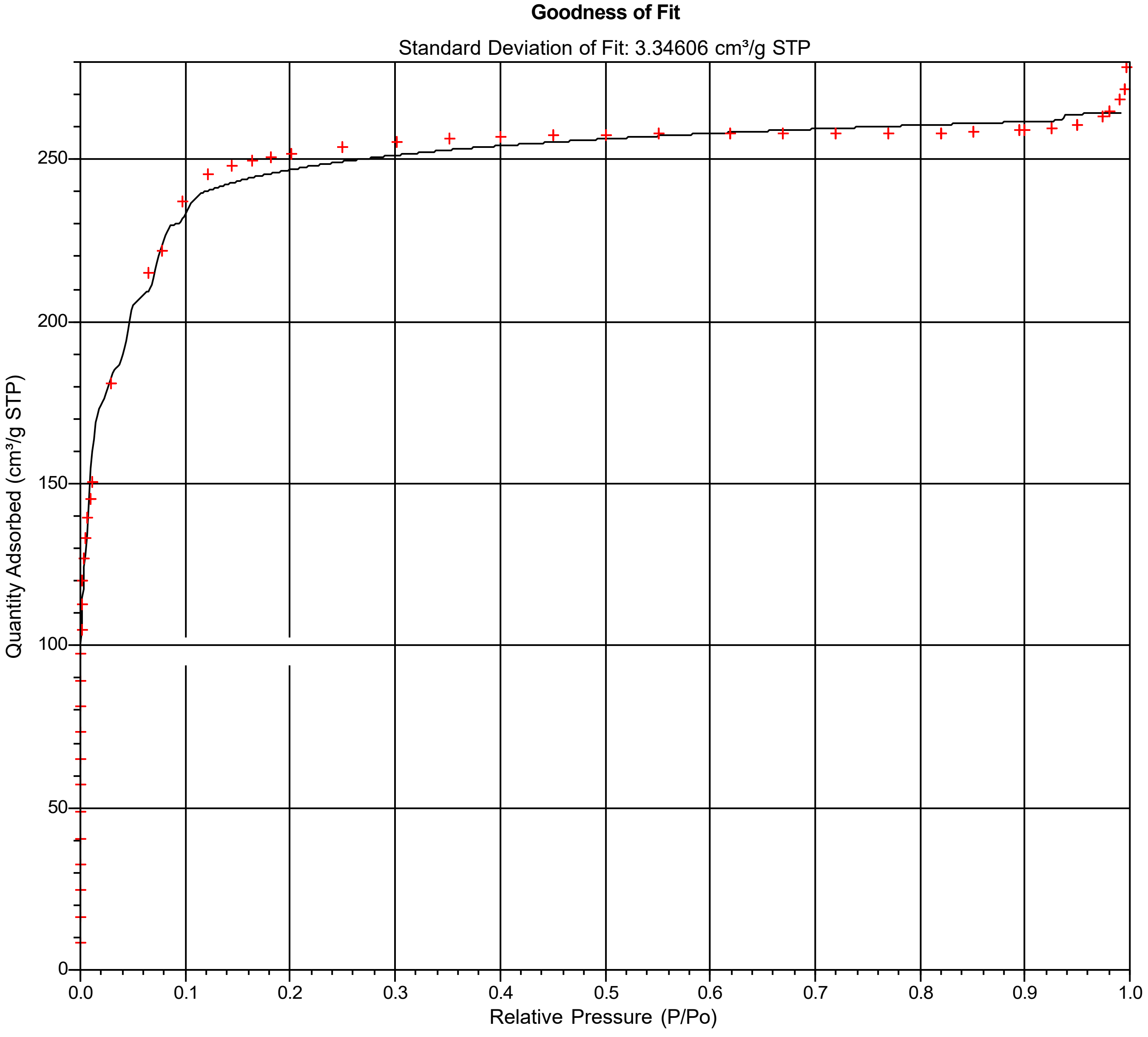

pharmaceutics

MDPI

Sample Information

Method:	Default
Sample:	20240425 Scale Up MIL-100-Fe 12h
Operator:	
Submitter:	Micromeritics
Mass type:	Calculated
Empty tube:	37.5702 g
Sample + tube:	37.6419 g
Sample mass:	0.0717 g
Density:	1.000 g/cm³
Type of data:	Automatically collected
Instrument type:	2020
Original instrument type:	2020
Comments:	Use sample tube with isothermal jacket and filler rod. Follow the instructions on the Silica-Alumina sample data sheet for sample preparation and special instructions. Be sure to use specifications for the current lot.

Sample Tube

Sample tube:	Sample Tube
Warm free space:	1.0000 cm³
Cold free space:	1.0000 cm³
Non-ideality factor:	0.0000620
Use isothermal jacket:	Yes
Use filler rod:	Yes
Vacuum seal type:	Seal Frit

Degas Conditions

Degas conditions: Degas Conditions

Evacuation Phase

Temperature ramp rate:	10.0 °C/min
Target temperature:	90 °C
Evacuation rate:	5.0 mmHg/s
Unrest. evacuation from:	5.0 mmHg
Vacuum level:	1.000000e-002 mmHg
Evacuation time:	60 min

Heading Phase

Ramp rate: 10.0 °C/min
Hold temp: 350 °C
Hold time: 360 min

Evacuation and Heating Phases

Hold pressure: 10 mmHg

Backfill

Backfill sample tube: Yes

Analysis Conditions

Analysis conditions: Silica Alumina, nitrogen @ 77.35 K
Absolute pressure dosing: No

Pressure Table

Relative Pressure (P/Po)	Rel. Pressure Increment (P/Po)
0.010000000	
0.030000000	
0.060000000	
0.080000000	
0.100000000	
0.120000000	
0.140000000	
0.160000000	
0.180000000	
0.200000000	
0.250000000	
0.300000000	
0.350000000	
0.400000000	
0.450000000	
0.500000000	
0.550000000	
0.600000000	
0.650000000	
0.700000000	
0.750000000	

pharmaceutics

MDPI

Pressure Table

Relative Pressure (P/Po)	Rel. Pressure Increment (P/Po)
0.800000000	
0.820000000	
0.850000000	
0.875000000	
0.900000000	
0.925000000	
0.950000000	
0.975000000	
0.980000000	
0.990000000	
0.995000000	
0.998000000	
0.990000000	
0.980000000	
0.975000000	
0.950000000	
0.925000000	
0.900000000	
0.875000000	
0.850000000	
0.825000000	
0.800000000	
0.750000000	
0.700000000	
0.650000000	
0.600000000	
0.550000000	
0.500000000	
0.450000000	
0.400000000	
0.350000000	
0.300000000	
0.250000000	
0.200000000	
0.140000000	

pharmaceutics

MDPI

Preparation

Fast evacuation: No
Unrestricted evacuation from: 5.0 mmHg
Vacuum setpoint: 10 µmHg
Evacuation time: 1.00 h

Leak test: No
Use TranSeal: No

Free Space

Measured before analysis
Lower Dewar for evacuation: No
Evacuation time: 2.00 h
Outgas test: No

Po and Temperature

Po type: Measured at intervals in Psat tube
Measurement interval: 120 min
Temperature type: Calculated from Po or Psat

Dosing

Use first pressure fixed dose: No
Use maximum volume increment: No
Target tolerance: 5.0% or 5.000 mmHg
Low pressure dosing: Yes
Dose amount: 8.0000 cm^3/g STP
Minimum equilibration delay: 0.00 h
Maximum equilibration delay: 2.00 h
Maximum number of decants: 6

Equilibration

	Relative Pressure (P/Po)	Equilibration Interval (s)
1	0.010000000	20
2	0.990000000	10

Minimum equilibration delay at P/Po >= 0.995: 600 s

Sample Backfill

Backfill at start of analysis: Yes
Backfill at end of analysis: Yes

pharmaceutics

MDPI

Sample Backfill

Backfill gas: N2

Adsorptive Properties

Adsorptive: Nitrogen @ 77.35 K (N2)
Non-condensing adsorptive: No
Maximum manifold pressure: 925.00 mmHg
Therm. tran. hard-sphere diameter: 3.8600 Å
Molecular cross-sectional area: 0.162 nm²
Adsorbate molecular weight: 28.01
Ideal gas law with non-ideality correction
Non-ideality factor: 0.0000660
Density conversion factor: 0.0015468
Dosing method: Normal

Psat vs. Temperature Table

	Saturation Pressure (mmHg)	Temperature (°C)
1	600.193	-197.750
2	634.512	-197.300
3	674.383	-196.800
4	720.420	-196.250
5	742.119	-196.000
6	759.833	-195.800
7	777.867	-195.600
8	805.525	-195.300
9	853.268	-194.800
10	903.122	-194.300

pharmaceutics

MDPI

Raw data for SYA@MIL-100(Fe) – 24 h

Summary Report

Surface Area

Single point surface area at P/Po = 0.249552124: 1,194.8904 m²/g

BET Surface Area: 1,451.3407 m²/g

Langmuir Surface Area: 1,729.7322 m²/g

t-Plot Micropore Area: 1,084.3122 m²/g

t-Plot external surface area: 367.0285 m²/g

BJH Adsorption cumulative surface area of pores
between 17.000 Å and 3,000.000 Å width: 118.4999 m²/g

BJH Desorption cumulative surface area of pores
between 17.000 Å and 3,000.000 Å width: 122.3236 m²/g

Pore Volume

Single point adsorption total pore volume of pores
less than 3,873.040 Å width at P/Po = 0.995000000: 0.619344 cm³/g

Single point desorption total pore volume of pores
less than 3,873.040 Å width at P/Po = 0.995000000: 0.628837 cm³/g

t-Plot micropore volume: 0.400067 cm³/g

BJH Adsorption cumulative volume of pores
between 17.000 Å and 3,000.000 Å width: 0.110793 cm³/g

BJH Desorption cumulative volume of pores
between 17.000 Å and 3,000.000 Å width: 0.126827 cm³/g

Pore Size

Adsorption average pore diameter (4V/A by BET): 17.070 Å

Desorption average pore diameter (4V/A by BET): 17.331 Å

Pore Size

BJH Adsorption average pore width (4V/A): 37.399 Å

BJH Desorption average pore width (4V/A): 41.473 Å

Horvath-Kawazoe

Maximum pore volume at P/Po = 0.096262998: 0.523374 cm^3/g

Median pore width: 14.898 Å

pharmaceutics

MDPI

Isotherm Tabular Report

Relative Pressure (P/Po)	Absolute Pressure (mmHg)	Quantity Adsorbed (cm³/g STP)	Elapsed Time (h:min)	Saturation Pressure (mmHg)
			03:44	760.479004
0.000006082	0.004624	8.3155	04:19	
0.000009021	0.006858	16.6340	04:53	
0.000012231	0.009297	24.9523	05:26	
0.000015990	0.012152	33.2700	05:59	
0.000020500	0.015577	41.5862	06:34	
0.000026192	0.019899	49.9018	07:07	
0.000033622	0.025541	58.2131	07:38	
0.000043434	0.032990	66.5223	08:09	
0.000056732	0.043084	74.8269	08:38	
0.000074939	0.056905	83.1237	09:05	
0.000100258	0.076121	91.4115	09:31	
0.000135840	0.103124	99.6845	09:57	
0.000185345	0.140691	107.9368	10:20	
0.000253486	0.192394	116.1652	10:43	
0.000346836	0.263220	124.3609	11:04	
0.000473403	0.359239	132.5357	11:25	
0.000643697	0.488420	140.6284	11:45	
0.000873276	0.662554	148.6406	12:06	
0.001182787	0.897304	156.5381	12:24	
0.001600200	1.213866	164.2781	12:42	
0.002157705	1.636636	171.8073	13:00	
0.002903232	2.201948	179.1805	13:17	
0.003874524	2.938389	186.2983	13:34	
0.005084379	3.855639	193.0446	13:50	
			13:59	758.298462
0.006734964	5.107655	199.9994	14:07	
0.008460300	6.417049	206.2119	14:18	
0.010431606	7.912998	212.1568	14:25	
0.029149453	22.115711	257.4268	14:39	
0.063489427	48.176498	306.8623	14:50	
0.077159375	58.555618	317.2988	14:58	
0.096262998	73.063850	338.3591	15:09	
0.121501486	92.228477	352.9083	15:16	
0.144900745	109.996048	356.9321	15:20	
0.165015184	125.271820	359.4420	15:24	

pharmaceutics

MDPI

Isotherm Tabular Report

Relative Pressure (P/Po)	Absolute Pressure (mmHg)	Quantity Adsorbed (cm³/g STP)	Elapsed Time (h:min)	Saturation Pressure (mmHg)
0.181953346	138.135971	361.0903	15:27	
0.201126564	152.698029	362.7336	15:30	
0.249640570	189.538055	365.8576	15:33	
0.301077784	228.600525	368.2732	15:36	
0.351341740	266.775238	369.9943	15:39	
0.399600967	303.430725	371.2703	15:42	
0.449593264	341.400635	372.2987	15:44	
0.499494717	379.308594	373.1213	15:47	
0.549410893	417.225220	373.7882	15:49	
0.599313757	455.139771	374.3185	15:52	
0.649231338	493.061981	374.8001	15:54	
0.699152463	530.988892	375.2166	15:56	
0.748988258	568.860535	375.6756	15:59	
			16:01	759.525391
0.798933371	606.810181	376.2239	16:03	
0.820125805	622.906372	376.5952	16:06	
0.850234296	645.774536	377.1719	16:08	
0.875168916	664.713013	377.8337	16:10	
0.900216521	683.737305	378.6394	16:12	
0.924923080	702.502563	379.8950	16:15	
0.950157280	721.668579	381.8917	16:17	
0.974525846	740.177124	385.9639	16:20	
0.980465144	744.688171	388.2964	16:22	
0.990393412	752.228943	393.7162	16:25	
0.994636318	755.451538	398.9995	16:28	
0.997086081	757.312195	408.4575	16:38	
0.979901662	744.260193	392.6671	16:43	
0.960213078	729.306213	385.3858	16:46	
0.933374901	708.921936	381.5805	16:49	
0.907383151	689.180542	379.6796	16:51	
0.882029215	669.923584	378.5934	16:53	
0.856846043	650.796326	377.8317	16:56	
0.831623978	631.639526	377.2583	16:59	
0.806594132	612.628723	376.8438	17:01	
0.781434425	593.519287	376.5090	17:03	
0.750146817	569.755554	376.1725	17:06	

pharmaceutics

MDPI

Isotherm Tabular Report

Relative Pressure (P/Po)	Absolute Pressure (mmHg)	Quantity Adsorbed (cm³/g STP)	Elapsed Time (h:min)	Saturation Pressure (mmHg)
0.700299195	531.895020	375.7598	17:08	
0.650339632	493.949463	375.3592	17:10	
0.600241730	455.898834	374.9537	17:12	
0.550283010	417.953918	374.4512	17:15	
0.500274107	379.970886	373.8146	17:17	
0.449719722	341.573547	372.8872	17:20	
0.399838389	303.687408	371.7643	17:22	
0.349837521	265.710480	370.3756	17:25	
0.299869821	227.758743	368.6570	17:28	
0.250108907	189.964066	366.3758	17:31	
0.200187816	152.047729	363.1894	17:35	
0.140804262	106.944412	357.1284	17:42	

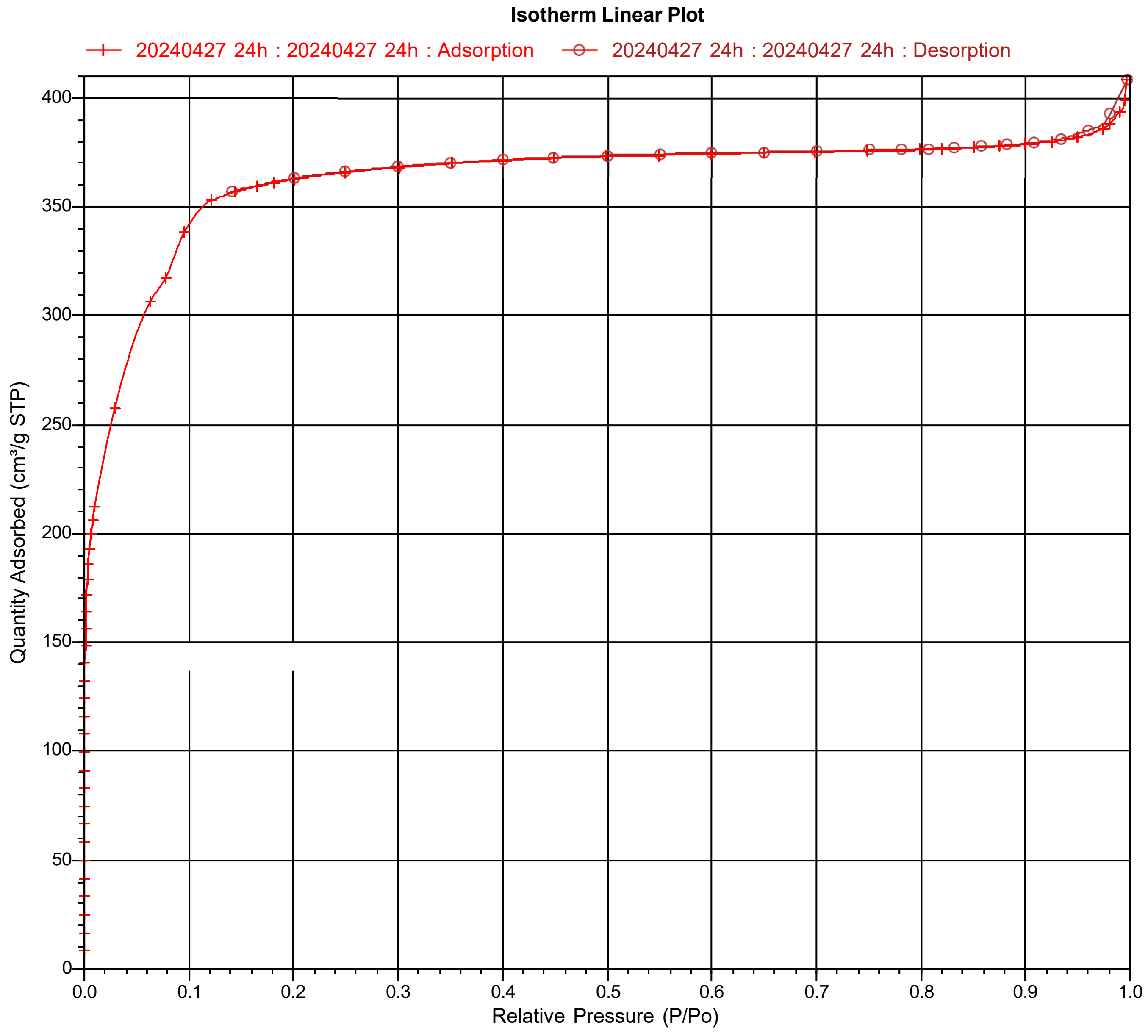

Isotherm Linear Plot
20240427 24h : 20240427 24h : Adsorption
20240427 24h : 20240427 24h : Desorption
Quantity Adsorbed (cm³/g STP)
Relative Pressure (P/Po)
0
50
100
150
200
250
300
350
400
0.0
0.1
0.2
0.3
0.4
0.5
0.6
0.7
0.8
0.9
1.0

pharmaceutics

MDPI

BET Report

BET surface area: 1451.3407 ± 16.9902 m²/g
Slope: 0.002967 ± 0.000035 g/cm³ STP
Y-intercept: 0.000032 ± 0.000003 g/cm³ STP
C: 95.089425
Qm: 333.4439 cm³/g STP
Correlation coefficient: 0.9997916
Molecular cross-sectional area: 0.1620 nm²

Relative Pressure (P/Po)	Quantity Adsorbed (cm³/g STP)	1/[Q(Po/P - 1)]
0.029149453	257.4268	0.000117
0.063489427	306.8623	0.000221
0.077159375	317.2988	0.000264
0.096262998	338.3591	0.000315
0.121501486	352.9083	0.000392

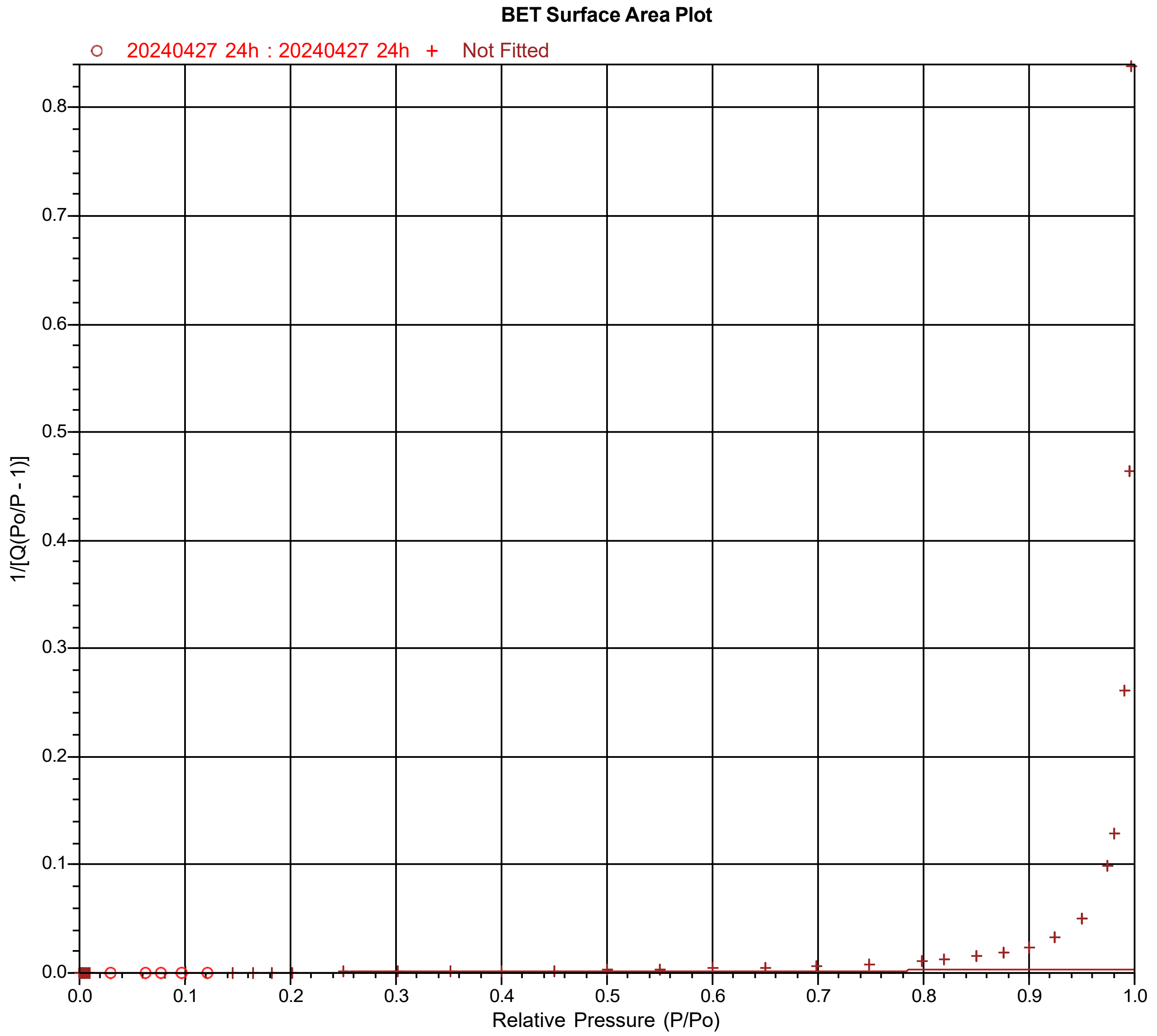
BET Surface Area Plot
20240427 24h : 20240427 24h
Not Fitted
0.8
0.7
0.6
0.5
0.4
0.3
0.2
0.1
0.0
1/[Q(Po/P - 1)]
0.0
0.1
0.2
0.3
0.4
0.5
0.6
0.7
0.8
0.9
1.0
Relative Pressure (P/Po)

pharmaceutics

MDPI

t-Plot Report

Micropore volume: 0.400067 cm³/g
Micropore area: 1084.3122 m²/g
External surface area: 367.0285 m²/g
Slope: 23.728244 ± 5.103473 cm³/g·Å STP
Y-intercept: 258.641943 ± 21.166547 cm³/g STP
Correlation coefficient: 0.901195
Surface area correction factor: 1.000
Density conversion factor: 0.0015468
Total surface area (BET): 1451.3407 m²/g
Thickness range: 3.5000 Å to 5.0000 Å
Thickness equation: Harkins and Jura

Thickness Curve

t = [13.99 / (0.034 - log(P/Po))] ^ 0.5

t-Plot Report - Data

Relative Pressure (P/Po)	Statistical Thickness (Å)	Quantity Adsorbed (cm³/g STP)	Fitted
0.000006082	1.6324	8.3155	
0.000009021	1.6597	16.6340	
0.000012231	1.6817	24.9523	
0.000015990	1.7019	33.2700	
0.000020500	1.7212	41.5862	
0.000026192	1.7409	49.9018	
0.000033622	1.7618	58.2131	
0.000043434	1.7839	66.5223	
0.000056732	1.8079	74.8269	
0.000074939	1.8340	83.1237	
0.000100258	1.8625	91.4115	
0.000135840	1.8937	99.6845	
0.000185345	1.9274	107.9368	
0.000253486	1.9631	116.1652	
0.000346836	2.0010	124.3609	
0.000473403	2.0409	132.5357	
0.000643697	2.0827	140.6284	
0.000873276	2.1268	148.6406	
0.001182787	2.1736	156.5381	
0.001600200	2.2235	164.2781	

pharmaceutics

MDPI

t-Plot Report - Data

Relative Pressure (P/Po)	Statistical Thickness (Å)	Quantity Adsorbed (cm³/g STP)	Fitted
0.002157705	2.2763	171.8073	
0.002903232	2.3326	179.1805	
0.003874524	2.3917	186.2983	
0.005084379	2.4515	193.0446	
0.006734964	2.5185	199.9994	
0.008460300	2.5770	206.2119	
0.010431606	2.6345	212.1568	
0.029149453	2.9857	257.4268	
0.063489427	3.3708	306.8623	
0.077159375	3.4930	317.2988	
0.096262998	3.6492	338.3591	*
0.121501486	3.8387	352.9083	*
0.144900745	4.0033	356.9321	*
0.165015184	4.1394	359.4420	*
0.181953346	4.2514	361.0903	*
0.201126564	4.3761	362.7336	*
0.249640570	4.6876	365.8576	*
0.301077784	5.0192	368.2732	
0.351341740	5.3528	369.9943	
0.399600967	5.6883	371.2703	
0.449593264	6.0582	372.2987	
0.499494717	6.4578	373.1213	
0.549410893	6.8970	373.7882	
0.599313757	7.3875	374.3185	
0.649231338	7.9455	374.8001	
0.699152463	8.5938	375.2166	

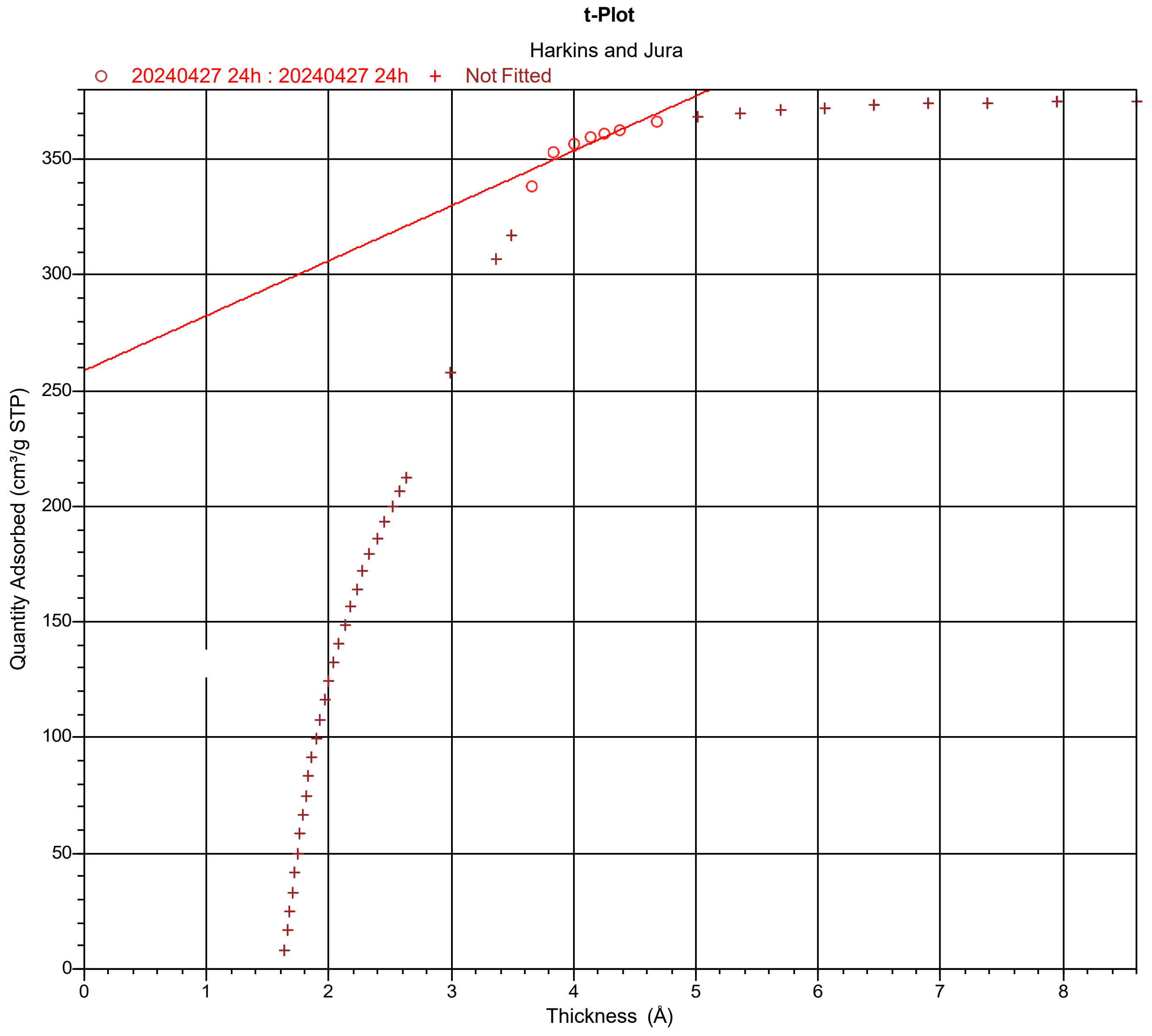
t-Plot
Harkins and Jura
20240427 24h : 20240427 24h
Not Fitted
Quantity Adsorbed (cm³/g STP)
Thickness (Å)
0
50
100
150
200
250
300
350
0
1
2
3
4
5
6
7
8

pharmaceutics

MDPI

BJH Adsorption Pore Distribution Report

Faas Correction
Halsey
t = 3.54 [-5 / ln(P/Po)] ^ 0.333

Width range: 17.000 Å to 3,000.000 Å
Adsorbate property factor: 9.53000 Å
Density conversion factor: 0.0015468
Fraction of pores open at both ends: 0.00

Pore Width Range (Å)	Average Width (Å)	Incremental Pore Volume (cm³/g)	Cumulative Pore Volume (cm³/g)	Incremental Pore Area (m²/g)	Cumulative Pore Area (m²/g)
3612.9 - 2031.3	2406.4	0.008483	0.008483	0.141	0.141
2031.3 - 1010.9	1208.5	0.008861	0.017343	0.293	0.434
1010.9 - 779.5	864.9	0.003857	0.021200	0.178	0.613
779.5 - 405.4	481.7	0.006894	0.028094	0.573	1.185
405.4 - 272.5	312.5	0.003405	0.031499	0.436	1.621
272.5 - 206.9	230.3	0.002175	0.033674	0.378	1.999
206.9 - 166.6	182.1	0.001386	0.035060	0.304	2.303
166.6 - 139.6	150.5	0.001171	0.036232	0.311	2.614
139.6 - 116.9	126.0	0.001014	0.037246	0.322	2.936
116.9 - 104.8	110.1	0.000677	0.037923	0.246	3.182
104.8 - 84.2	92.0	0.000937	0.038860	0.407	3.590
84.2 - 70.3	75.8	0.000824	0.039684	0.435	4.025
70.3 - 60.1	64.3	0.000796	0.040480	0.495	4.520
60.1 - 52.4	55.6	0.001059	0.041539	0.762	5.281
52.4 - 46.2	48.8	0.001279	0.042817	1.048	6.330
46.2 - 41.1	43.3	0.001796	0.044613	1.659	7.989
41.1 - 36.9	38.7	0.002412	0.047025	2.493	10.482
36.9 - 33.2	34.8	0.003257	0.050282	3.742	14.224
33.2 - 30.1	31.5	0.004352	0.054634	5.528	19.752
30.1 - 27.3	28.5	0.006296	0.060931	8.833	28.585
27.3 - 24.6	25.7	0.009541	0.070472	14.828	43.413
24.6 - 22.2	23.2	0.013248	0.083720	22.802	66.215
22.2 - 21.3	21.7	0.007370	0.091090	13.559	79.774
21.3 - 20.5	20.9	0.007572	0.098662	14.492	94.266
20.5 - 19.6	20.0	0.012131	0.110793	24.234	118.500

pharmaceutics

MDPI

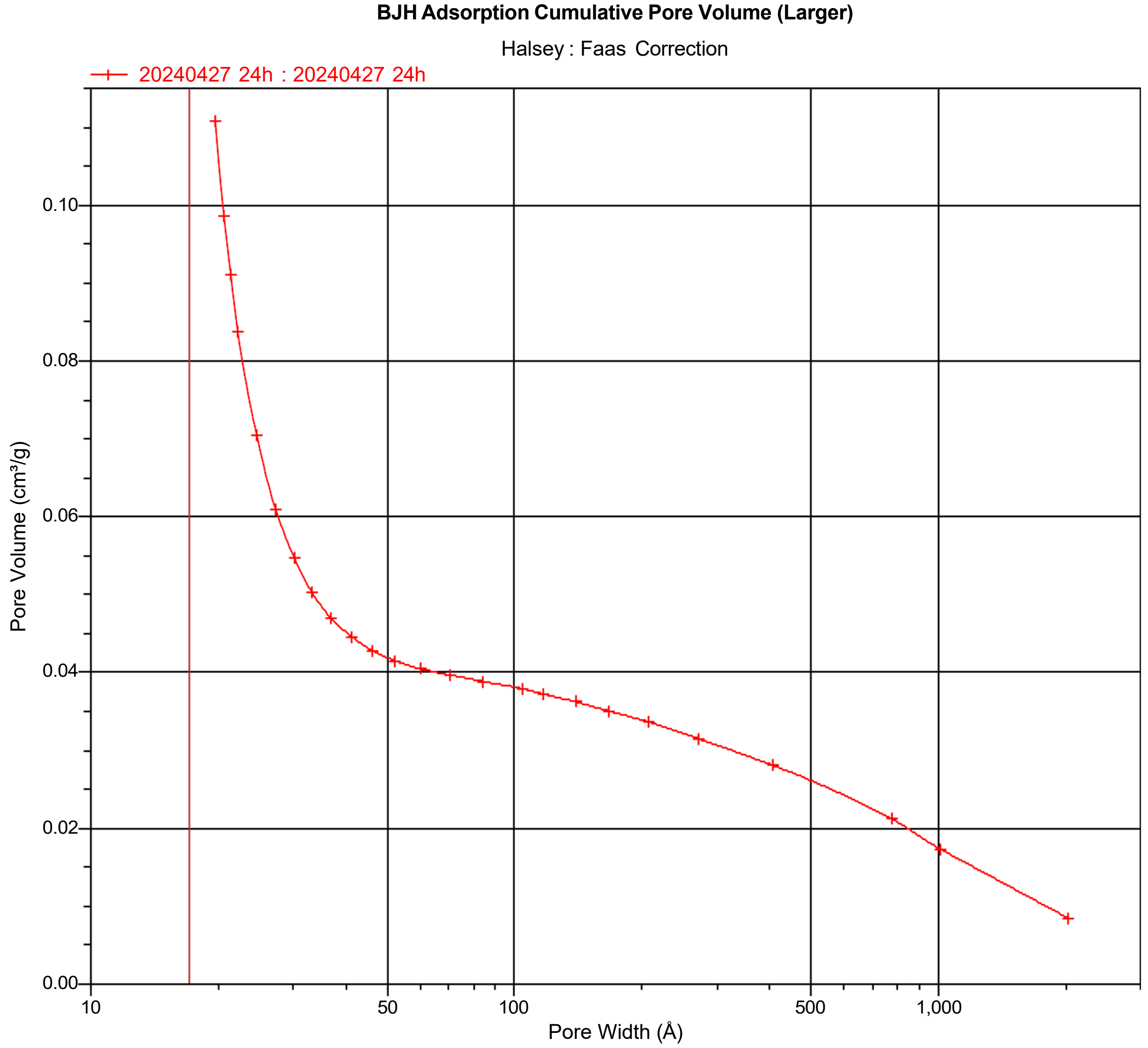

pharmaceutics
MDPI

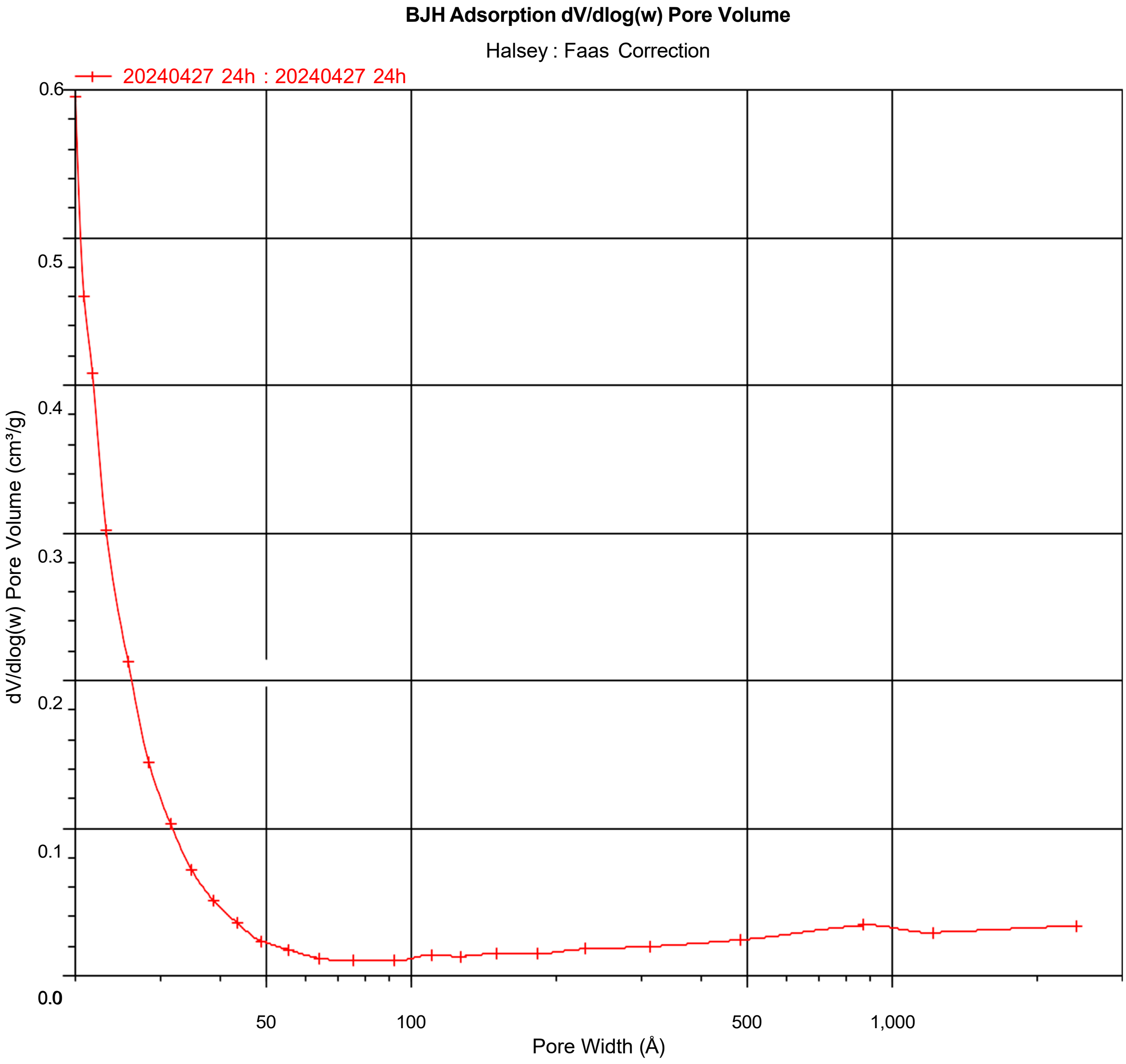

pharmaceutics

MDPI

.

BJH Desorption Pore Distribution Report

Faas Correction

Halsey

t = 3.54 [-5 / ln(P/Po)] ^ 0.333

Width range: 17.000 Å to 3,000.000 Å

Adsorbate property factor: 9.53000 Å

Density conversion factor: 0.0015468

Fraction of pores open at both ends: 0.00

Pore Width Range (Å)	Average Width (Å)	Incremental Pore Volume (cm³/g)	Cumulative Pore Volume (cm³/g)	Incremental Pore Area (m²/g)	Cumulative Pore Area (m²/g)
6616.0 - 983.1	1098.0	0.026521	0.026521	0.966	0.966
983.1 - 504.6	600.7	0.012227	0.038749	0.814	1.780
504.6 - 305.9	357.3	0.006416	0.045164	0.718	2.499
305.9 - 222.4	250.4	0.003197	0.048362	0.511	3.009
222.4 - 176.0	193.4	0.001794	0.050156	0.371	3.380
176.0 - 145.9	157.9	0.001253	0.051409	0.317	3.698
145.9 - 124.6	133.4	0.000938	0.052347	0.281	3.979
124.6 - 108.9	115.6	0.000652	0.052999	0.226	4.205
108.9 - 96.6	101.9	0.000518	0.053517	0.203	4.408
96.6 - 84.6	89.7	0.000511	0.054029	0.228	4.636
84.6 - 70.6	76.1	0.000609	0.054638	0.320	4.956
70.6 - 60.3	64.5	0.000676	0.055313	0.419	5.375
60.3 - 52.5	55.7	0.000772	0.056085	0.554	5.929
52.5 - 46.3	48.9	0.001145	0.057230	0.936	6.865
46.3 - 41.2	43.4	0.001655	0.058885	1.526	8.392
41.2 - 36.9	38.7	0.002743	0.061628	2.832	11.223
36.9 - 33.2	34.8	0.003567	0.065195	4.097	15.321
33.2 - 30.1	31.4	0.004717	0.069912	6.001	21.322
30.1 - 27.2	28.4	0.006220	0.076131	8.749	30.070
27.2 - 24.6	25.7	0.008897	0.085029	13.833	43.903
24.6 - 22.2	23.2	0.013442	0.098471	23.156	67.059
22.2 - 19.4	20.5	0.028357	0.126827	55.265	122.324

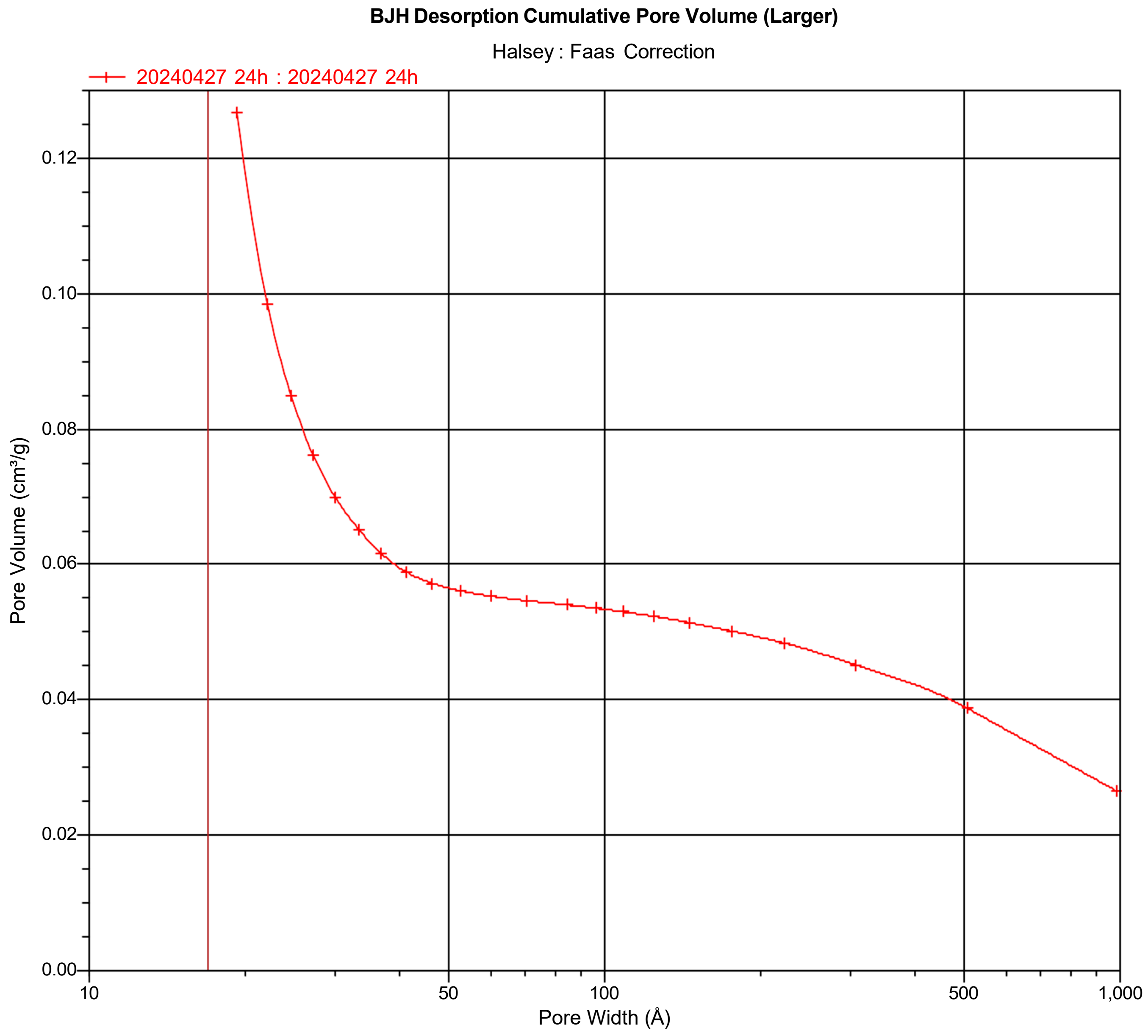
BJH Desorption Cumulative Pore Volume (Larger)
Halsey : Faas Correction
20240427 24h : 20240427 24h
Pore Volume (cm³/g)
0.00
0.02
0.04
0.06
0.08
0.10
0.12
10
50
100
500
1,000
Pore Width (Å)

pharmaceutics

MDPI

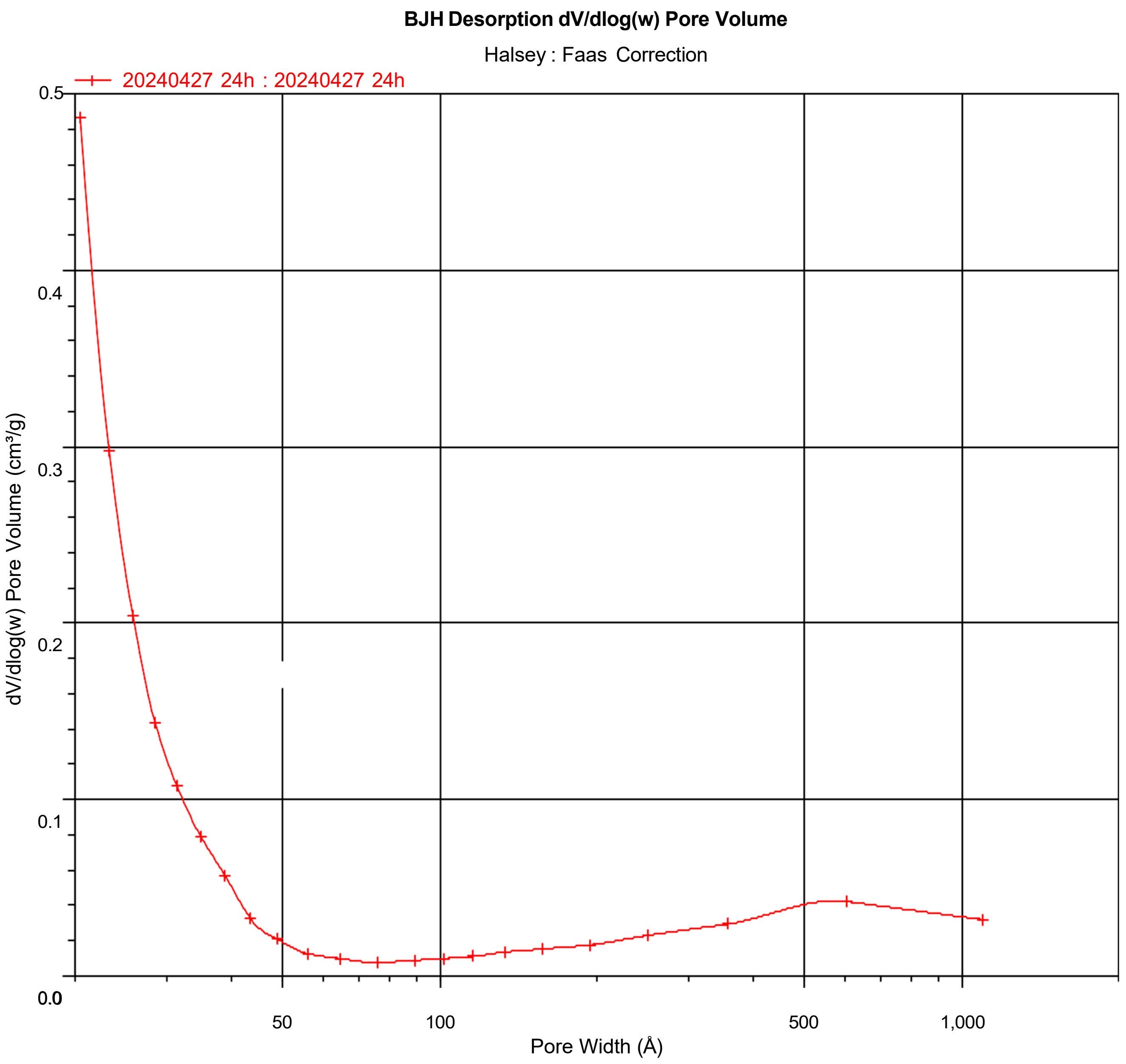

pharmaceutics

MDPI

Porosity Distribution by
Model: N2 - Tarazona NLDFT, Esf = 30.0K
Method: Non-negative Regularization: 0.01000
Standard Deviation of Fit: 4.51078 cm³/g STP

Volume in Pores	<	5.22 Å	:	0.00000 cm³/g
Total Volume in Pores	<=	448.83 Å	:	0.61528 cm³/g
Total Area in Pores	>=	5.22 Å	:	2,461.474 m²/g

Pore Size Table

Pore Width (Å)	Cumulative Volume (cm³/g)	Incremental Volume (cm³/g)	Cumulative Area (m²/g)	Incremental Area (m²/g)
5.22	0.00671	0.00671	51.427	51.427
5.93	0.03233	0.02562	224.156	172.729
6.29	0.08212	0.04978	540.681	316.525
6.65	0.14933	0.06721	945.054	404.372
7.36	0.21854	0.06921	1321.019	375.966
7.72	0.26782	0.04927	1576.290	255.271
8.44	0.29412	0.02631	1701.023	124.732
8.79	0.30738	0.01326	1761.326	60.303
9.51	0.32160	0.01422	1821.142	59.816
10.22	0.33840	0.01680	1886.878	65.737
10.94	0.35118	0.01278	1933.597	46.719
11.65	0.35730	0.00612	1954.615	21.017
12.37	0.35730	0.00000	1954.615	0.000
13.08	0.35730	0.00000	1954.615	0.000
13.80	0.35730	0.00000	1954.615	0.000
14.87	0.35730	0.00000	1954.615	0.000
15.59	0.35730	0.00000	1954.615	0.000
16.66	0.37221	0.01491	1990.424	35.809
17.73	0.41064	0.03843	2077.118	86.694
18.44	0.46515	0.05451	2195.328	118.210
19.87	0.51447	0.04932	2294.586	99.258
20.95	0.55655	0.04209	2374.954	80.369
22.02	0.58852	0.03196	2433.017	58.062
23.45	0.60464	0.01613	2460.526	27.509
24.52	0.60464	0.00000	2460.526	0.000
25.95	0.60464	0.00000	2460.526	0.000
27.38	0.60464	0.00000	2460.526	0.000

pharmaceutics

MDPI

Pore Size Table

Pore Width (Å)	Cumulative Volume (cm³/g)	Incremental Volume (cm³/g)	Cumulative Area (m²/g)	Incremental Area (m²/g)
29.17	0.60464	0.00000	2460.526	0.000
30.60	0.60464	0.00000	2460.526	0.000
32.39	0.60464	0.00000	2460.526	0.000
34.17	0.60464	0.00000	2460.526	0.000
35.96	0.60464	0.00000	2460.526	0.000
38.11	0.60464	0.00000	2460.526	0.000
40.25	0.60464	0.00000	2460.526	0.000
42.39	0.60464	0.00000	2460.526	0.000
44.54	0.60464	0.00000	2460.526	0.000
47.04	0.60464	0.00000	2460.526	0.000
49.54	0.60464	0.00000	2460.526	0.000
52.05	0.60464	0.00000	2460.526	0.000
54.91	0.60464	0.00000	2460.526	0.000
57.77	0.60464	0.00000	2460.526	0.000
60.98	0.60464	0.00000	2460.526	0.000
64.20	0.60464	0.00000	2460.526	0.000
67.42	0.60464	0.00000	2460.526	0.000
70.99	0.60464	0.00000	2460.526	0.000
74.57	0.60464	0.00000	2460.526	0.000
78.50	0.60464	0.00000	2460.526	0.000
82.79	0.60464	0.00000	2460.526	0.000
87.08	0.60464	0.00000	2460.526	0.000
91.37	0.60464	0.00000	2460.526	0.000
96.37	0.60464	0.00000	2460.526	0.000
101.38	0.60464	0.00000	2460.526	0.000
106.38	0.60464	0.00000	2460.526	0.000
112.10	0.60464	0.00000	2460.526	0.000
117.82	0.60464	0.00000	2460.526	0.000
123.90	0.60464	0.00000	2460.526	0.000
130.33	0.60464	0.00000	2460.526	0.000
136.76	0.60464	0.00000	2460.526	0.000
143.91	0.60464	0.00000	2460.526	0.000
151.06	0.60464	0.00000	2460.526	0.000
158.93	0.60464	0.00000	2460.526	0.000
167.15	0.60464	0.00000	2460.526	0.000
175.73	0.60464	0.00000	2460.526	0.000

pharmaceutics

MDPI

Pore Size Table

Pore Width (Å)	Cumulative Volume (cm³/g)	Incremental Volume (cm³/g)	Cumulative Area (m²/g)	Incremental Area (m²/g)
184.66	0.60464	0.00000	2460.526	0.000
193.96	0.60464	0.00000	2460.526	0.000
203.97	0.60464	0.00000	2460.526	0.000
214.33	0.60464	0.00000	2460.526	0.000
225.06	0.60464	0.00000	2460.526	0.000
236.50	0.60464	0.00000	2460.526	0.000
248.29	0.60464	0.00000	2460.526	0.000
261.16	0.60464	0.00000	2460.526	0.000
274.39	0.60464	0.00000	2460.526	0.000
287.97	0.60464	0.00000	2460.526	0.000
302.63	0.60464	0.00000	2460.526	0.000
318.00	0.60464	0.00000	2460.526	0.000
334.08	0.60464	0.00000	2460.526	0.000
350.88	0.60464	0.00000	2460.526	0.000
368.76	0.60464	0.00000	2460.526	0.000
387.34	0.60464	0.00000	2460.526	0.000
406.65	0.60464	0.00000	2460.526	0.000
427.38	0.60464	0.00000	2460.526	0.000
448.83	0.61528	0.01064	2461.474	0.948

pharmaceutics

MDPI

Porosity Distribution by
Model: N2 - Tarazona NLDFT, Esf = 30.0K
Method: Non-negative Regularization: 0.01000

Standard Deviation of Fit: 4.51078 cm³/g STP

Isotherm Table

Relative Pressure (P/Po)	Experimental Quantity Adsorbed (cm³/g STP)	Fitted Quantity Adsorbed (cm³/g STP)	Absolute Residual (cm³/g STP)	Relative Residual
0.000006310	8.9852	20.2492	-11.2640	-1.253609
0.000007943	13.6545	23.6869	-10.0325	-0.734738
0.000010000	19.2721	27.4536	-8.1815	-0.424523
0.000012589	25.8190	31.6136	-5.7946	-0.224430
0.000015849	32.9837	35.9587	-2.9750	-0.090195
0.000019953	40.6723	40.2192	0.4531	0.011141
0.000025119	48.5064	44.4881	4.0183	0.082841
0.000031623	56.2085	49.2321	6.9764	0.124117
0.000039811	63.7416	55.4241	8.3175	0.130488
0.000050119	71.0167	63.4705	7.5462	0.106259
0.000063096	78.0123	73.0665	4.9458	0.063398
0.000079433	84.7760	83.0346	1.7414	0.020541
0.000100000	91.3430	89.7747	1.5683	0.017169
0.000125892	97.6648	96.5204	1.1445	0.011718
0.000158490	103.7752	106.3251	-2.5498	-0.024571
0.000199526	109.8368	114.1435	-4.3067	-0.039210
0.000251188	115.9359	119.0963	-3.1604	-0.027260
0.000316228	121.9596	123.6896	-1.7300	-0.014185
0.000398107	127.9526	130.6378	-2.6852	-0.020986
0.000501187	134.0039	135.0444	-1.0405	-0.007765
0.000630958	140.1171	141.7706	-1.6535	-0.011801
0.000794328	146.1705	146.4558	-0.2852	-0.001951
0.001000000	152.1607	152.3261	-0.1654	-0.001087
0.001258925	158.1116	156.7633	1.3482	0.008527
0.001584895	164.0427	161.4736	2.5691	0.015661
0.001995263	169.8667	165.3539	4.5128	0.026567
0.002511882	175.5814	169.3238	6.2576	0.035640
0.003162276	181.2532	173.7776	7.4757	0.041244
0.003981066	186.9549	178.2205	8.7344	0.046719

pharmaceutics

MDPI

Isotherm Table

Relative Pressure (P/Po)	Experimental Quantity Adsorbed (cm³/g STP)	Fitted Quantity Adsorbed (cm³/g STP)	Absolute Residual (cm³/g STP)	Relative Residual
0.005011868	192.7074	183.4089	9.2986	0.048252
0.006309579	198.3363	191.3949	6.9414	0.034998
0.007943276	204.4453	205.3906	-0.9453	-0.004624
0.010000000	210.9353	220.7279	-9.7925	-0.046424
0.012355640	217.4706	233.7690	-16.2984	-0.074945
0.015186320	225.0591	242.3613	-17.3022	-0.076878
0.018485530	233.5193	248.3705	-14.8511	-0.063597
0.022294740	242.7073	253.2986	-10.5913	-0.043638
0.026653420	252.3586	257.6249	-5.2663	-0.020869
0.031598160	262.1311	263.8225	-1.6914	-0.006452
0.037162240	272.1300	267.6515	4.4785	0.016457
0.043374470	282.1756	277.9311	4.2445	0.015042
0.050259210	291.9559	293.3580	-1.4021	-0.004803
0.057835260	301.1084	297.2524	3.8560	0.012806
0.066115920	309.0879	300.7970	8.2909	0.026824
0.075109080	315.4965	314.9823	0.5143	0.001630
0.084815920	325.7728	327.7535	-1.9807	-0.006080
0.095232370	337.4363	330.6720	6.7643	0.020046
0.106348200	346.1114	341.3697	4.7418	0.013700
0.118147500	352.0215	348.0179	4.0035	0.011373
0.130609100	354.6832	350.1506	4.5327	0.012779
0.143706600	356.7762	352.0811	4.6951	0.013160
0.157410500	358.5633	353.8435	4.7198	0.013163
0.171685500	360.1148	355.4619	4.6528	0.012920
0.186492100	361.4997	356.9550	4.5446	0.012572
0.201792100	362.7848	358.3377	4.4471	0.012258
0.217539500	363.9226	359.6219	4.3007	0.011818
0.233689500	364.9540	360.8177	4.1363	0.011334
0.250196100	365.8873	361.9335	3.9538	0.010806
0.267011800	366.7572	362.9767	3.7805	0.010308
0.284089500	367.5668	363.9537	3.6131	0.009830
0.301380300	368.2847	364.8699	3.4148	0.009272
0.318838200	368.9322	365.7303	3.2019	0.008679
0.336417100	369.5349	366.5391	2.9957	0.008107
0.354071100	370.0730	367.3004	2.7725	0.007492

pharmaceutics

MDPI

Isotherm Table

Relative Pressure (P/Po)	Experimental Quantity Adsorbed (cm³/g STP)	Fitted Quantity Adsorbed (cm³/g STP)	Absolute Residual (cm³/g STP)	Relative Residual
0.371757900	370.5700	368.0177	2.5523	0.006888
0.389435500	371.0301	368.6941	2.3359	0.006296
0.407065800	371.4373	369.3328	2.1045	0.005666
0.424610500	371.8140	369.9363	1.8777	0.005050
0.442034200	372.1596	370.5073	1.6523	0.004440
0.459305300	372.4705	371.0487	1.4218	0.003817
0.476393400	372.7611	371.5642	1.1969	0.003211
0.493271100	373.0286	372.0624	0.9661	0.002590
0.509911800	373.2722	372.5536	0.7186	0.001925
0.526293400	373.5004	373.0003	0.5001	0.001339
0.542394700	373.7063	373.4176	0.2887	0.000773
0.558200000	373.8864	373.8108	0.0755	0.000202
0.573690800	374.0544	374.1825	-0.1281	-0.000342
0.588853900	374.2128	374.5343	-0.3215	-0.000859
0.603677600	374.3620	374.8677	-0.5056	-0.001351
0.618153900	374.5057	375.1838	-0.6781	-0.001811
0.632272400	374.6428	375.4837	-0.8409	-0.002245
0.646028900	374.7712	375.7686	-0.9974	-0.002661
0.659417100	374.8884	376.0392	-1.1508	-0.003070
0.672435500	374.9956	376.2964	-1.3008	-0.003469
0.685081600	375.0982	376.5412	-1.4430	-0.003847
0.697355300	375.2010	376.7743	-1.5733	-0.004193
0.709256600	375.3061	376.9966	-1.6905	-0.004504
0.720789500	375.4113	377.2092	-1.7979	-0.004789
0.731953900	375.5153	377.4132	-1.8980	-0.005054
0.742756600	375.6169	377.6119	-1.9950	-0.005311
0.753200000	375.7152	377.8239	-2.1086	-0.005612
0.763289500	375.8113	378.0299	-2.2186	-0.005904
0.773030300	375.9091	378.2052	-2.2962	-0.006108
0.782430300	376.0119	378.3681	-2.3562	-0.006266
0.791496100	376.1225	378.5216	-2.3991	-0.006379
0.800232900	376.2430	378.6669	-2.4239	-0.006442
0.808648700	376.3833	378.8048	-2.4215	-0.006434
0.816752600	376.5328	378.9361	-2.4033	-0.006383
0.824552600	376.6762	379.0612	-2.3850	-0.006332

Isotherm Table

Relative Pressure (P/Po)	Experimental Quantity Adsorbed (cm³/g STP)	Fitted Quantity Adsorbed (cm³/g STP)	Absolute Residual (cm³/g STP)	Relative Residual
0.832053900	376.8151	379.1807	-2.3656	-0.006278
0.839267100	376.9523	379.2951	-2.3428	-0.006215
0.846200000	377.0892	379.4050	-2.3158	-0.006141
0.852860500	377.2292	379.5115	-2.2822	-0.006050
0.859257900	377.3871	379.6164	-2.2293	-0.005907
0.865398700	377.5553	379.7301	-2.1747	-0.005760
0.871292100	377.7235	379.8737	-2.1502	-0.005692
0.876947400	377.8844	379.9715	-2.0871	-0.005523
0.882369700	378.0461	380.0600	-2.0139	-0.005327
0.887569700	378.2103	380.1427	-1.9325	-0.005109
0.892553900	378.3750	380.2210	-1.8461	-0.004879
0.897328900	378.5383	380.2956	-1.7573	-0.004642
0.901905300	378.7019	380.3672	-1.6653	-0.004397
0.906286800	378.8886	380.4362	-1.5476	-0.004085
0.910484200	379.0943	380.5038	-1.4095	-0.003718
0.914501300	379.3086	380.5722	-1.2637	-0.003332
0.918347400	379.5233	380.6949	-1.1715	-0.003087
0.922026300	379.7320	380.7797	-1.0478	-0.002759
0.925547400	379.9304	380.8433	-0.9129	-0.002403
0.928915800	380.1397	380.9014	-0.7617	-0.002004
0.932136800	380.3655	380.9563	-0.5909	-0.001553
0.935218400	380.6010	381.0097	-0.4087	-0.001074
0.938163200	380.8404	386.8698	-6.0293	-0.015832
0.940978900	381.0794	386.9075	-5.8281	-0.015294
0.943669700	381.3143	386.9435	-5.6292	-0.014763
0.946242100	381.5425	386.9777	-5.4352	-0.014245
0.948700000	381.7619	387.0103	-5.2484	-0.013748
0.951048700	381.9734	387.0414	-5.0680	-0.013268
0.953292100	382.2006	387.0711	-4.8705	-0.012743
0.955435500	382.4468	387.0992	-4.6524	-0.012165
0.957482900	382.7090	387.1262	-4.4172	-0.011542
0.959438200	382.9844	387.1517	-4.1673	-0.010881
0.961305300	383.2704	387.1761	-3.9057	-0.010190
0.963088200	383.5648	387.1993	-3.6346	-0.009476
0.964789500	383.8653	387.2214	-3.3561	-0.008743

pharmaceutics

MDPI

Isotherm Table

Relative Pressure (P/Po)	Experimental Quantity Adsorbed (cm³/g STP)	Fitted Quantity Adsorbed (cm³/g STP)	Absolute Residual (cm³/g STP)	Relative Residual
0.966414500	384.1704	387.2425	-3.0721	-0.007997
0.967965800	384.4784	387.2627	-2.7843	-0.007242
0.969447400	384.7878	387.2818	-2.4940	-0.006482
0.970860500	385.0970	387.3000	-2.2031	-0.005721
0.972209200	385.4050	387.3174	-1.9124	-0.004962
0.973496100	385.7108	387.3340	-1.6232	-0.004208
0.974725000	386.0152	387.3498	-1.3346	-0.003457
0.975897400	386.3772	387.3648	-0.9876	-0.002556
0.977015800	386.7988	387.3791	-0.5803	-0.001500
0.978082900	387.2469	387.3927	-0.1458	-0.000376
0.979101300	387.6960	387.4058	0.2902	0.000749
0.980072400	388.1259	387.4181	0.7078	0.001824
0.980998700	388.5313	387.4299	1.1014	0.002835
0.981882900	388.9430	387.4412	1.5018	0.003861
0.982726300	389.3592	387.4519	1.9074	0.004899
0.983530300	389.7748	387.4621	2.3127	0.005933
0.984297400	390.1860	387.4719	2.7141	0.006956
0.985028900	390.5896	387.4811	3.1085	0.007958
0.985727600	390.9839	387.4900	3.4939	0.008936
0.986392100	391.3653	387.4983	3.8669	0.009881
0.987027600	391.7346	387.5064	4.2282	0.010794
0.987632900	392.0894	387.5140	4.5754	0.011669
0.988209200	392.4291	387.5213	4.9077	0.012506
0.988760500	392.7547	387.5283	5.2265	0.013307
0.989285500	393.0649	387.5349	5.5299	0.014069
0.989785500	393.3596	387.5412	5.8184	0.014792
0.990263200	393.6400	387.5472	6.0928	0.015478
0.990718400	393.8777	387.5530	6.3248	0.016058
0.991151300	394.0313	387.5584	6.4729	0.016427
0.991565800	394.1573	387.5636	6.5937	0.016728
0.991959200	394.3005	387.5686	6.7319	0.017073

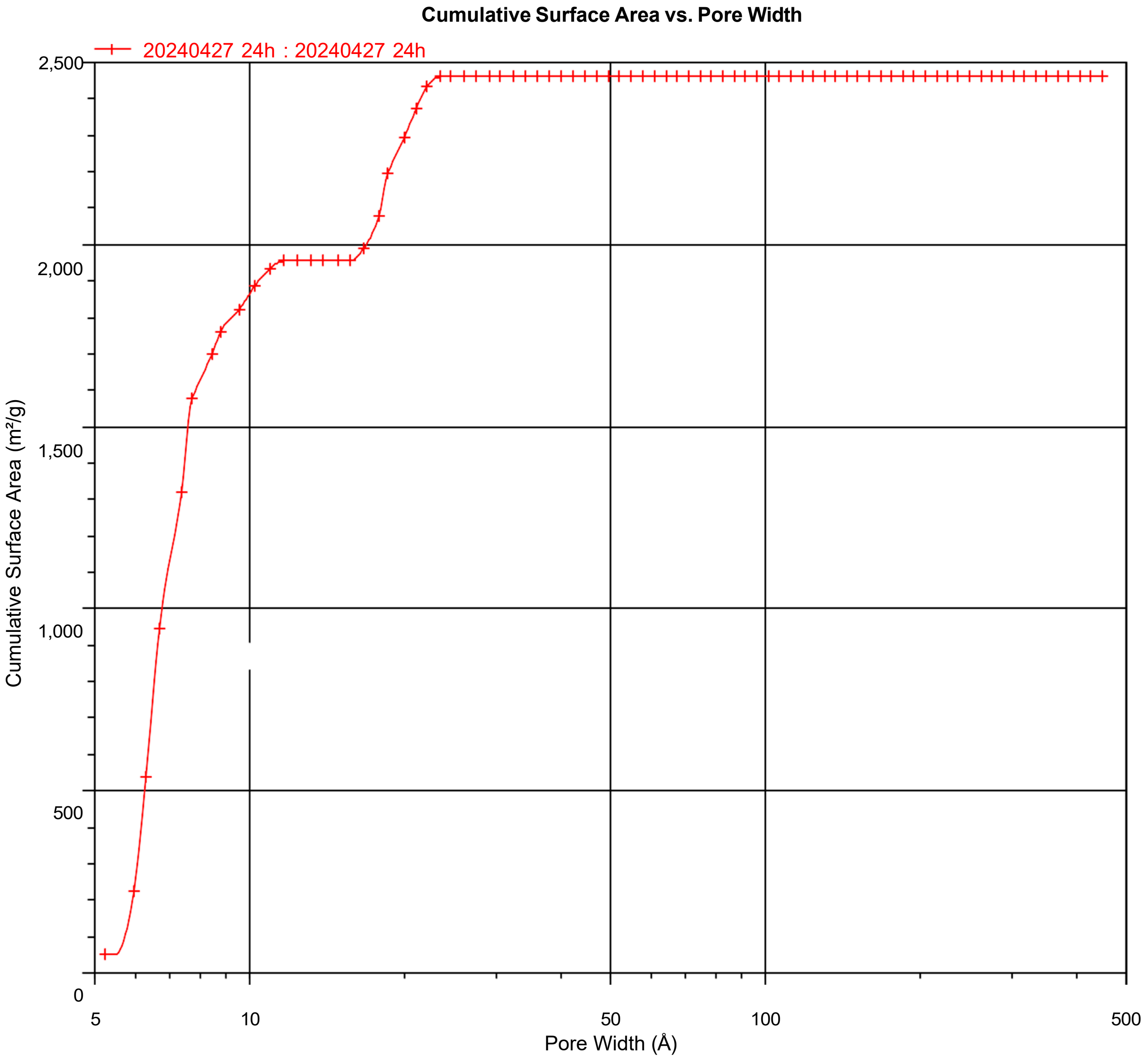
Cumulative Surface Area vs. Pore Width
20240427 24h : 20240427 24h
Cumulative Surface Area (m²/g)
2,500
2,000
1,500
1,000
500
0
5
10
50
100
500
Pore Width (Å)

pharmaceutics

MDPI

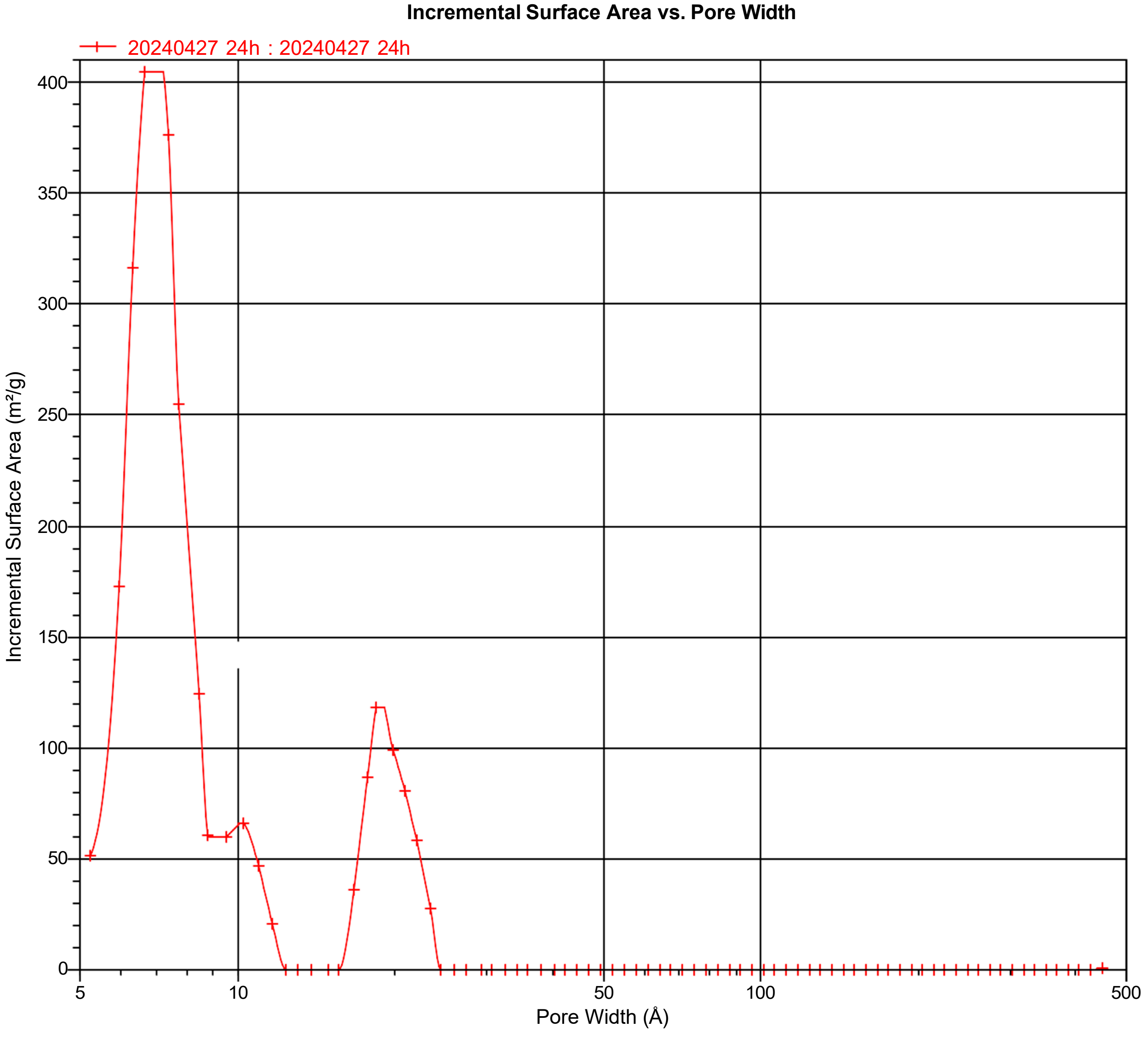

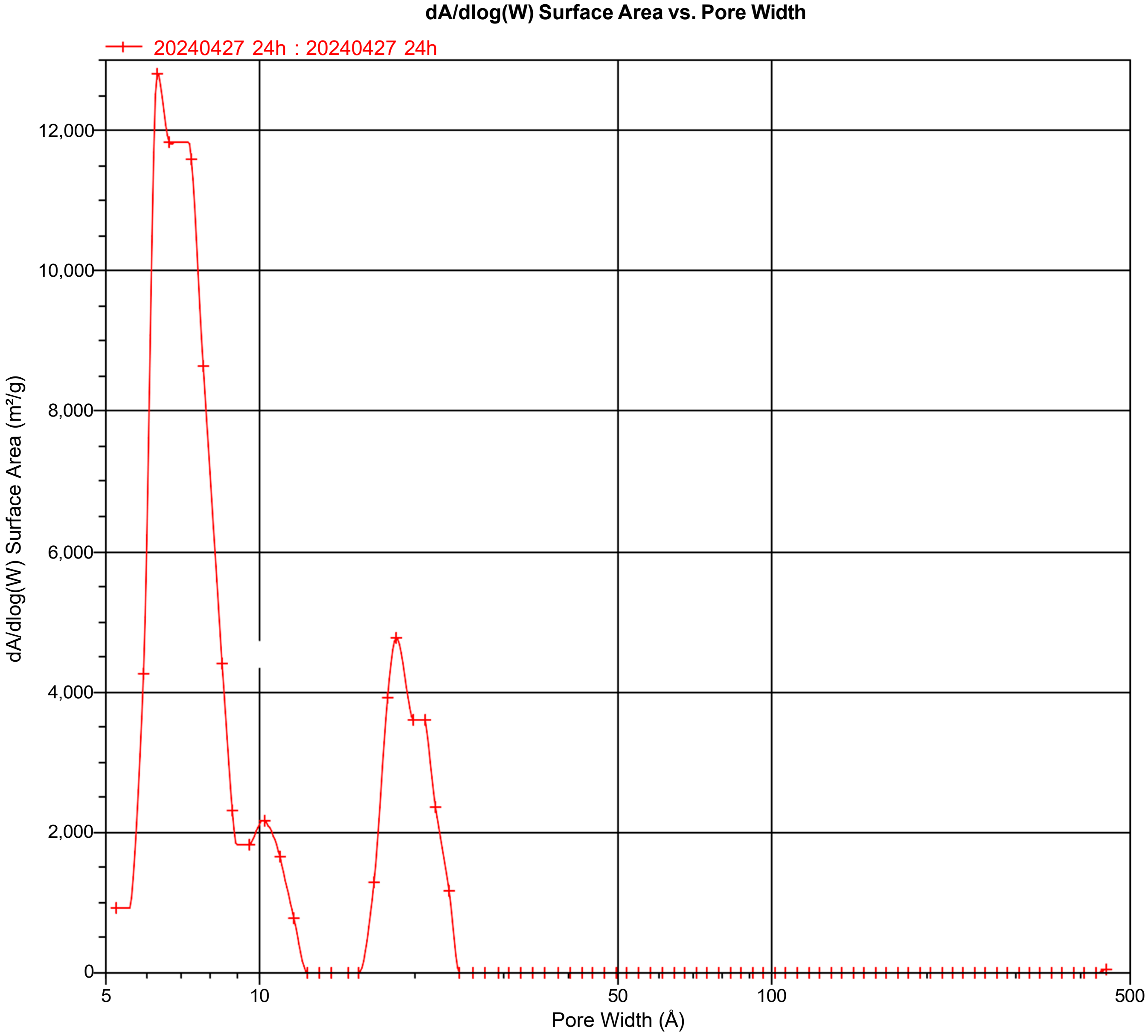
dA/dlog(W) Surface Area vs. Pore Width
20240427 24h : 20240427 24h
dA/dlog(W) Surface Area (m²/g)
12,000
10,000
8,000
6,000
4,000
2,000
0
5
10
50
100
500
Pore Width (Å)

pharmaceutics

MDPI

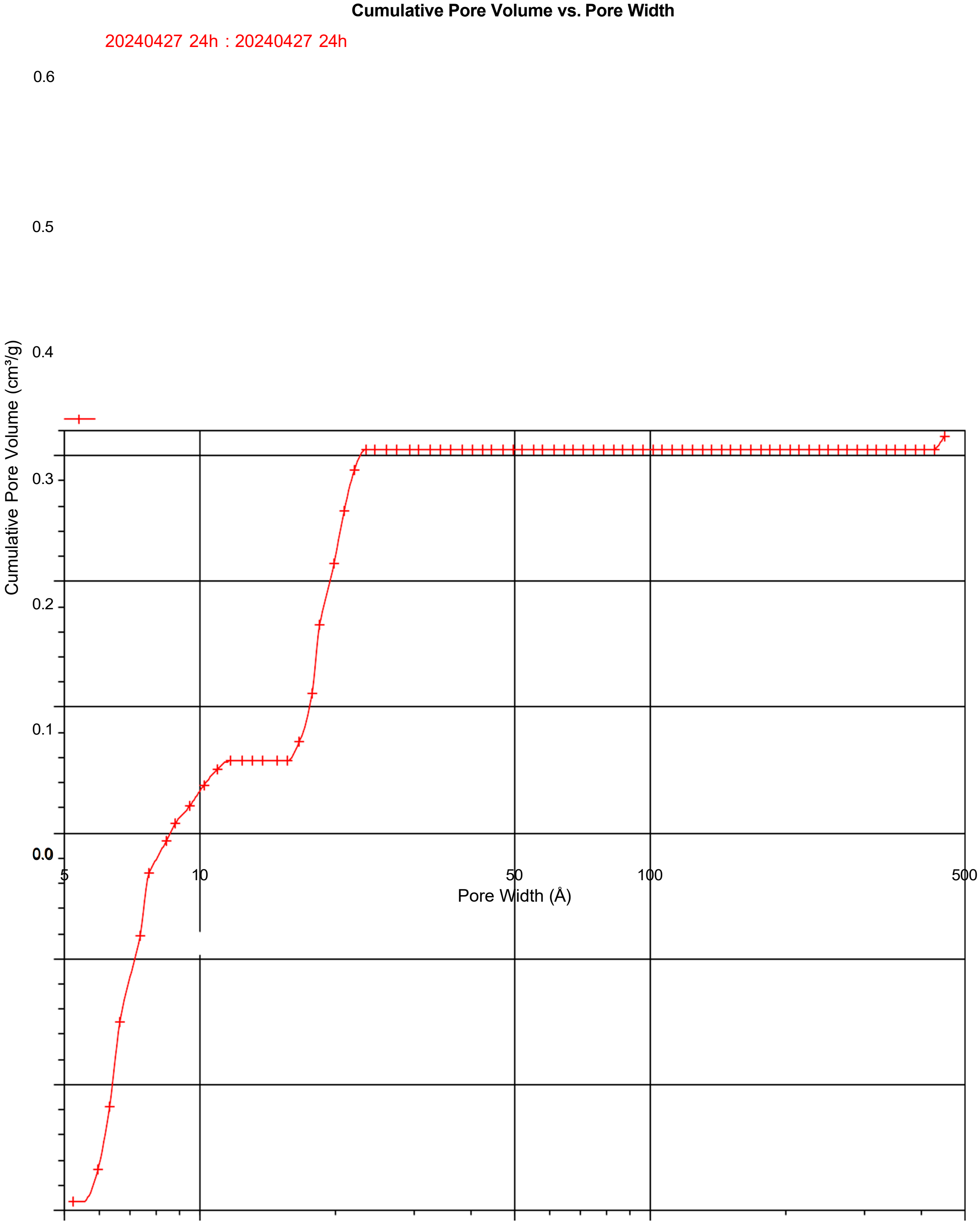

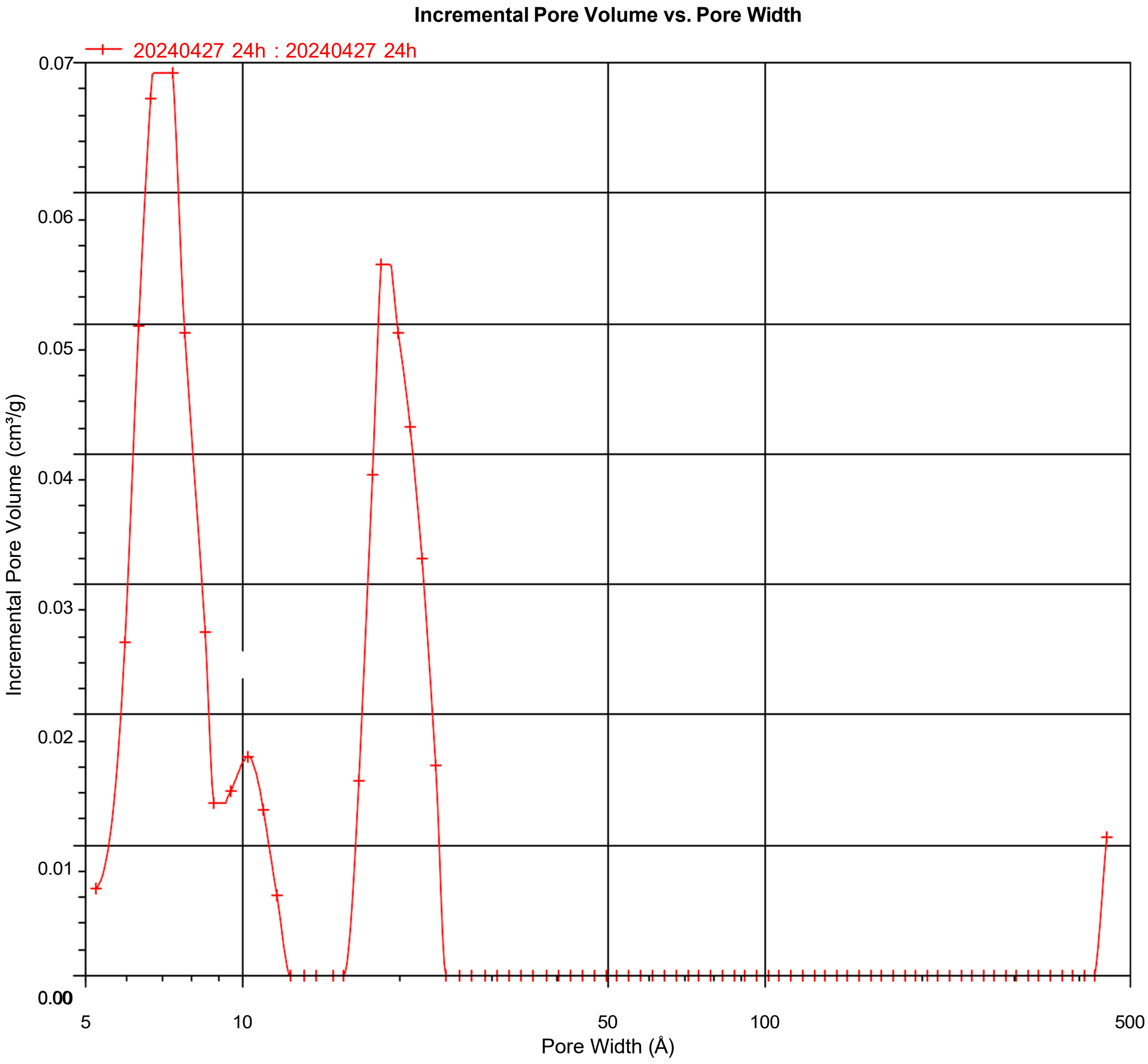
Incremental Pore Volume vs. Pore Width
20240427 24h : 20240427 24h
Incremental Pore Volume (cm³/g)
0.07
0.06
0.05
0.04
0.03
0.02
0.01
0.00
5
10
50
100
500
Pore Width (Å)

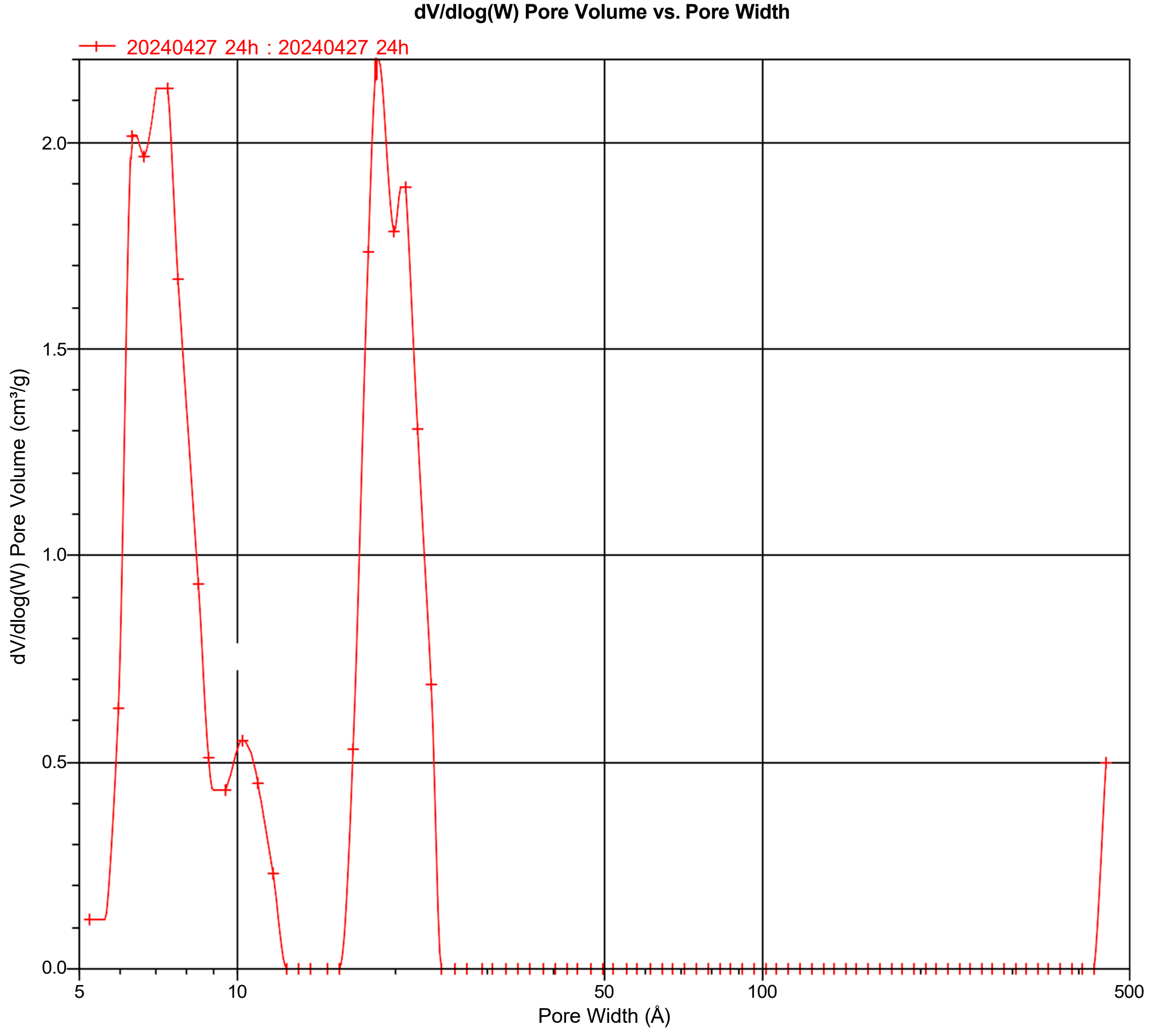
dV/dlog(W) Pore Volume vs. Pore Width
20240427 24h : 20240427 24h
dV/dlog(W) Pore Volume (cm³/g)
Pore Width (Å)
0.0
0.5
1.0
1.5
2.0
5
10
50
100
500

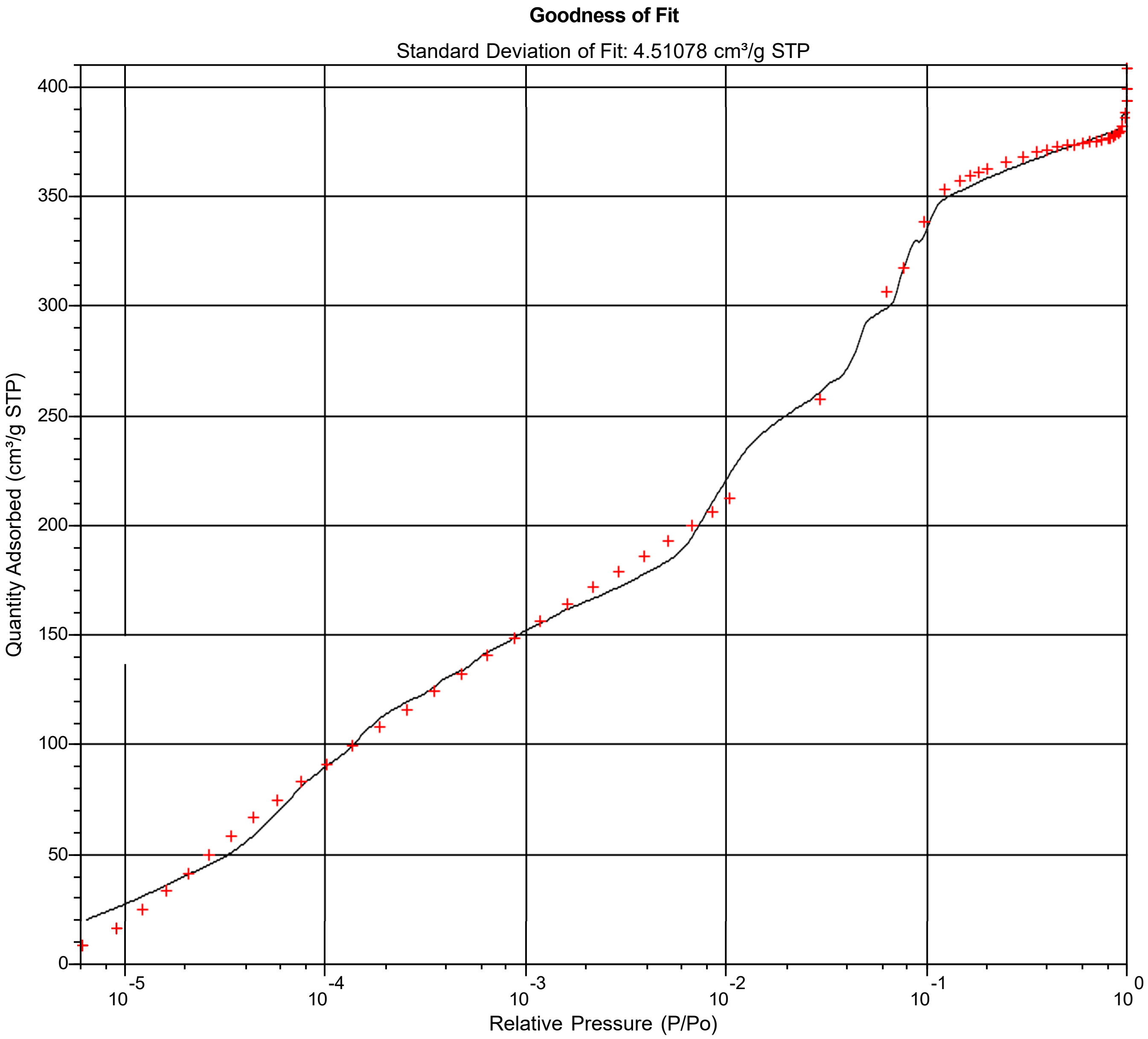
Goodness of Fit
Standard Deviation of Fit: 4.51078 cm³/g STP
Quantity Adsorbed (cm³/g STP)
400
350
300
250
200
150
100
50
0
10-5
10-4
10-3
10-2
10-1
100
Relative Pressure (P/Po)

pharmaceutics

MDPI

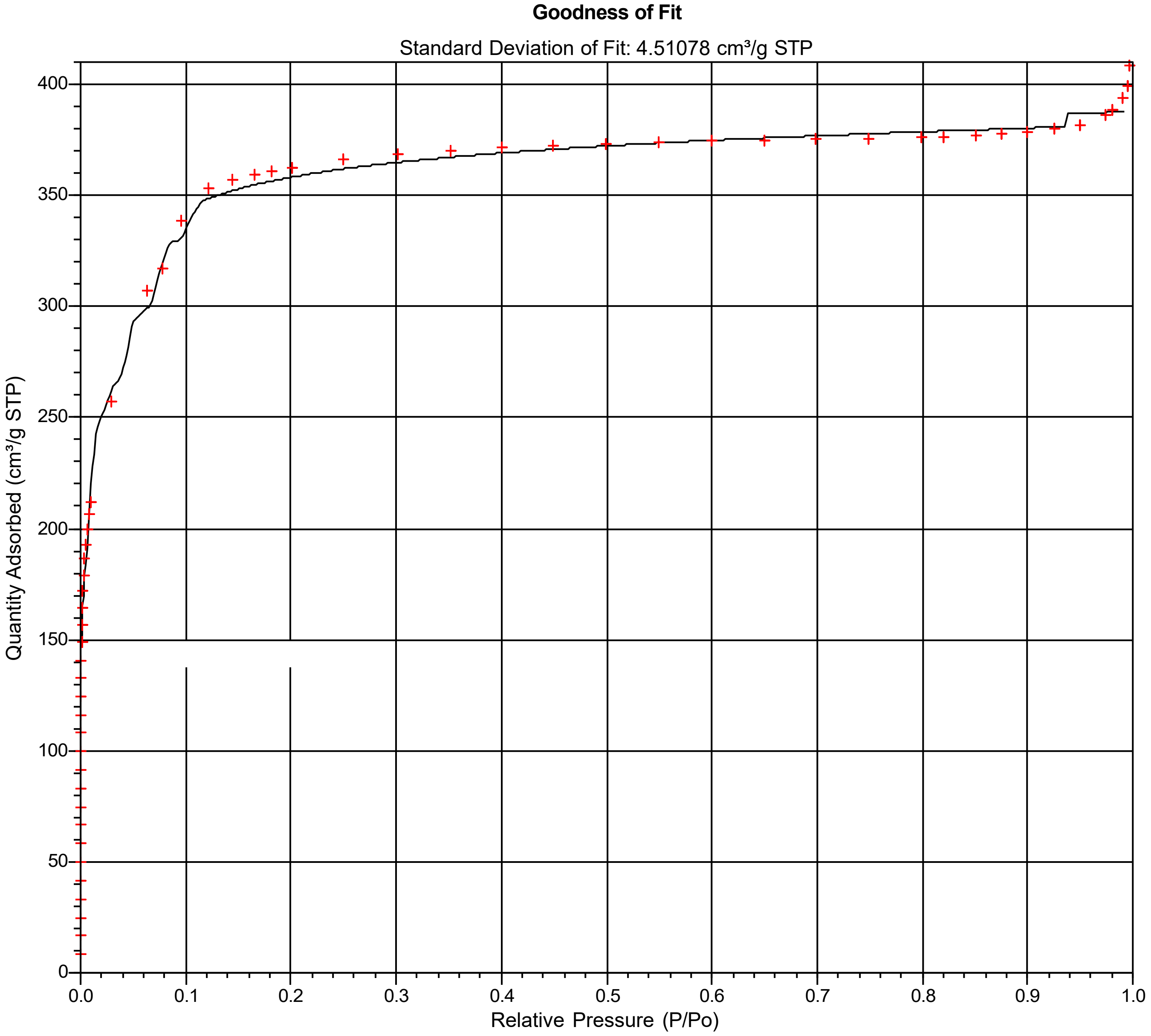

pharmaceutics

Sample Information

Method: Default
Sample: 20240427 24h
Operator:
Submitter: Micromeritics
Mass type: Calculated
Empty tube: 37.5702 g
Sample + tube: 37.6419 g
Sample mass: 0.0717 g
Density: 1.000 g/cm³
Type of data: Automatically collected
Instrument type: 2020
Original instrument type: 2020
Comments: Use sample tube with isothermal jacket and filler rod. Follow the instructions on the Silica-Alumina sample data sheet for sample preparation and special instructions. Be sure to use specifications for the current lot.

Sample Tube

Sample tube: Sample Tube
Warm free space: 1.0000 cm³
Cold free space: 1.0000 cm³
Non-ideality factor: 0.0000620
Use isothermal jacket: Yes
Use filler rod: Yes
Vacuum seal type: Seal Frit

Degas Conditions

Degas conditions: Degas Conditions

Evacuation Phase

Temperature ramp rate: 10.0 °C/min
Target temperature: 90 °C
Evacuation rate: 5.0 mmHg/s
Unrest. evacuation from: 5.0 mmHg
Vacuum level: 1.000000e-002 mmHg
Evacuation time: 60 min

Heading Phase

Ramp rate: 10.0 °C/min
Hold temp: 350 °C
Hold time: 360 min

Evacuation and Heating Phases

Hold pressure: 10 mmHg

Backfill

Backfill sample tube: Yes

Analysis Conditions

Analysis conditions: Silica Alumina, nitrogen @ 77.35 K
Absolute pressure dosing: No

Pressure Table

Relative Pressure (P/Po)	Rel. Pressure Increment (P/Po)
0.010000000	
0.030000000	
0.060000000	
0.080000000	
0.100000000	
0.120000000	
0.140000000	
0.160000000	
0.180000000	
0.200000000	
0.250000000	
0.300000000	
0.350000000	
0.400000000	
0.450000000	
0.500000000	
0.550000000	
0.600000000	
0.650000000	
0.700000000	
0.750000000	

pharmaceutics

MDPI

Pressure Table

Relative Pressure (P/Po)	Rel. Pressure Increment (P/Po)
0.800000000	
0.820000000	
0.850000000	
0.875000000	
0.900000000	
0.925000000	
0.950000000	
0.975000000	
0.980000000	
0.990000000	
0.995000000	
0.998000000	
0.990000000	
0.980000000	
0.975000000	
0.950000000	
0.925000000	
0.900000000	
0.875000000	
0.850000000	
0.825000000	
0.800000000	
0.750000000	
0.700000000	
0.650000000	
0.600000000	
0.550000000	
0.500000000	
0.450000000	
0.400000000	
0.350000000	
0.300000000	
0.250000000	
0.200000000	
0.140000000	

pharmaceutics

MDPI

Preparation

Fast evacuation: No
Unrestricted evacuation from: 5.0 mmHg
Vacuum setpoint: 10 μmHg
Evacuation time: 1.00 h

Leak test: No
Use TranSeal: No

Free Space

Measured before analysis
Lower Dewar for evacuation: No
Evacuation time: 2.00 h
Outgas test: No

Po and Temperature

Po type: Measured at intervals in Psat tube
Measurement interval: 120 min
Temperature type: Calculated from Po or Psat

Dosing

Use first pressure fixed dose: No
Use maximum volume increment: No
Target tolerance: 5.0% or 5.000 mmHg
Low pressure dosing: Yes
Dose amount: 8.0000 cm^3/g STP
Minimum equilibration delay: 0.00 h
Maximum equilibration delay: 2.00 h
Maximum number of decants: 6

Equilibration

	Relative Pressure (P/Po)	Equilibration Interval (s)
1	0.010000000	20
2	0.990000000	10

Minimum equilibration delay at P/Po >= 0.995: 600 s

Sample Backfill

Backfill at start of analysis: Yes
Backfill at end of analysis: Yes

Sample Backfill

Backfill gas: N2

Adsorptive Properties

Adsorptive: Nitrogen @ 77.35 K (N2)
Non-condensing adsorptive: No
Maximum manifold pressure: 925.00 mmHg
Therm. tran. hard-sphere diameter: 3.8600 Å
Molecular cross-sectional area: 0.162 nm²
Adsorbate molecular weight: 28.01
Ideal gas law with non-ideality correction
Non-ideality factor: 0.0000660
Density conversion factor: 0.0015468
Dosing method: Normal

Psat vs. Temperature Table

	Saturation Pressure (mmHg)	Temperature (°C)
1	600.193	-197.750
2	634.512	-197.300
3	674.383	-196.800
4	720.420	-196.250
5	742.119	-196.000
6	759.833	-195.800
7	777.867	-195.600
8	805.525	-195.300
9	853.268	-194.800
10	903.122	-194.300

pharmaceutics

MDPI

Raw data for SYA@MIL-100(Fe) – 36 h

Summary Report

Surface Area

Single point surface area at P/Po = 0.249552124: 1,086.2521 m²/g

BET Surface Area: 1,311.1947 m²/g

Langmuir Surface Area: 1,568.7056 m²/g

t-Plot Micropore Area: 999.3614 m²/g

t-Plot external surface area: 311.8334 m²/g

BJH Adsorption cumulative surface area of pores between 17.000 Å and 3,000.000 Å width: 98.5000 m²/g

BJH Desorption cumulative surface area of pores between 17.000 Å and 3,000.000 Å width: 102.6201 m²/g

Pore Volume

Single point adsorption total pore volume of pores less than 3,873.040 Å width at P/Po = 0.995000000: 0.555394 cm³/g

Single point desorption total pore volume of pores less than 3,873.040 Å width at P/Po = 0.995000000: 0.563750 cm³/g

t-Plot micropore volume: 0.373316 cm³/g

BJH Adsorption cumulative volume of pores between 17.000 Å and 3,000.000 Å width: 0.088495 cm³/g

BJH Desorption cumulative volume of pores between 17.000 Å and 3,000.000 Å width: 0.102345 cm³/g

Pore Size

Adsorption average pore diameter (4V/A by BET): 16.943 Å

Desorption average pore diameter (4V/A by BET): 17.198 Å

pharmaceutics

MDPI

Pore Size

BJH Adsorption average pore width (4V/A): 35.937 Å

BJH Desorption average pore width (4V/A): 39.893 Å

Horvath-Kawazoe

Maximum pore volume at P/Po = 0.096395595: 0.478303 cm^3/g

Median pore width: 14.946 Å

pharmaceutics

MDPI

Isotherm Tabular Report

Relative Pressure (P/Po)	Absolute Pressure (mmHg)	Quantity Adsorbed (cm³/g STP)	Elapsed Time (h:min)	Saturation Pressure (mmHg)
			03:44	760.872253
0.000006945	0.005283	8.2804	04:40	
0.000010001	0.007602	16.5640	06:15	
0.000013394	0.010176	24.8485	07:42	
0.000017323	0.013153	33.1327	09:09	
0.000022382	0.016986	41.4146	10:29	
0.000029074	0.022054	49.6955	11:47	
0.000038444	0.029150	57.9720	12:52	
0.000051719	0.039201	66.2417	13:51	
0.000070665	0.053543	74.5030	14:43	
0.000098208	0.074391	82.7520	15:29	
0.000138865	0.105163	90.9817	16:07	
0.000196913	0.149086	99.1860	16:46	
0.000279907	0.211880	107.3610	17:17	
0.000395539	0.299353	115.4962	17:47	
0.000554900	0.419884	123.5765	18:16	
0.000773169	0.584948	131.5840	18:42	
0.001070402	0.809694	139.4874	19:07	
0.001475539	1.115999	147.2471	19:29	
0.002031136	1.536011	154.9216	19:50	
0.002779210	2.101461	162.4024	20:10	
0.003756887	2.840339	169.5906	20:31	
0.004978419	3.763382	176.3872	20:51	
0.006469590	4.889996	182.9196	21:11	
			21:16	755.819214
0.008163579	6.170584	188.6492	21:27	
0.010196669	7.707868	194.7806	21:39	
0.028697839	21.695667	238.4861	21:58	
0.066248167	50.087627	282.0718	22:11	
0.077932096	58.924122	290.0489	22:19	
0.096395595	72.889793	309.2211	22:32	
0.122397593	92.555573	321.5195	22:40	
0.144787226	109.489517	324.7719	22:45	
0.165285320	124.993279	326.9825	22:49	
0.181894534	137.556015	328.4140	22:52	
0.201318532	152.247879	329.7746	22:55	

pharmaceutics

MDPI

Isotherm Tabular Report

Relative Pressure (P/Po)	Absolute Pressure (mmHg)	Quantity Adsorbed (cm³/g STP)	Elapsed Time (h:min)	Saturation Pressure (mmHg)
0.249269493	188.514252	332.4298	22:58	
0.301180595	227.778183	334.4299	23:02	
0.351515415	265.850250	335.8266	23:05	
0.399861419	302.417725	336.8091	23:07	
0.449865641	340.242157	337.5112	23:10	
0.499774829	377.993835	338.0531	23:12	
0.549882705	415.899078	338.3545	23:15	
			23:17	756.350220
0.618864268	468.078125	338.5904	23:19	
0.669169456	506.126465	338.7852	23:22	
0.700106649	529.525818	338.9105	23:24	
0.750238096	567.442749	339.0183	23:26	
0.818936459	619.402771	339.4028	23:29	
0.820167007	620.333496	339.6559	23:31	
0.850273264	643.104370	339.9009	23:33	
0.893711868	675.959167	340.6388	23:36	
0.900273821	680.922302	340.9942	23:38	
0.925341602	699.882324	341.7790	23:41	
0.949424477	718.097412	343.3137	23:43	
0.974532688	737.088013	346.5933	23:46	
0.980409040	741.532593	348.5036	23:48	
0.990265204	748.987305	353.0534	23:51	
0.994587575	752.256531	357.5773	23:54	
0.996872186	753.984497	365.7892	24:04	
0.978788645	740.307007	352.9739	24:09	
0.959743842	725.902466	347.1420	24:12	
0.933402986	705.979553	343.7885	24:15	
0.907400895	686.312866	342.0046	24:17	
0.881968040	667.076721	341.0709	24:19	
0.856703036	647.967529	340.5138	24:22	
0.831518405	628.919128	340.1968	24:24	
0.806272929	609.824707	339.9460	24:26	
0.781300774	590.937012	339.8330	24:29	
0.731260837	553.089294	339.7090	24:31	
0.681244786	515.259644	339.5333	24:33	
0.650215033	491.790283	339.3761	24:35	

pharmaceutics

MDPI

Isotherm Tabular Report

Relative Pressure (P/Po)	Absolute Pressure (mmHg)	Quantity Adsorbed (cm³/g STP)	Elapsed Time (h:min)	Saturation Pressure (mmHg)
0.600165453	453.935272	339.2808	24:38	
0.530459750	401.213348	339.0202	24:40	
0.500151952	378.290039	338.7264	24:42	
0.450263242	340.556702	338.2480	24:44	
0.399827083	302.409302	337.5001	24:47	
0.349921224	264.662994	336.5000	24:50	
0.299981371	226.890976	335.1705	24:53	
0.250106278	189.167938	333.2570	24:56	
0.200177299	151.404144	330.5053	25:01	
0.140824702	106.512794	325.1839	25:08	

pharmaceutics

MDPI

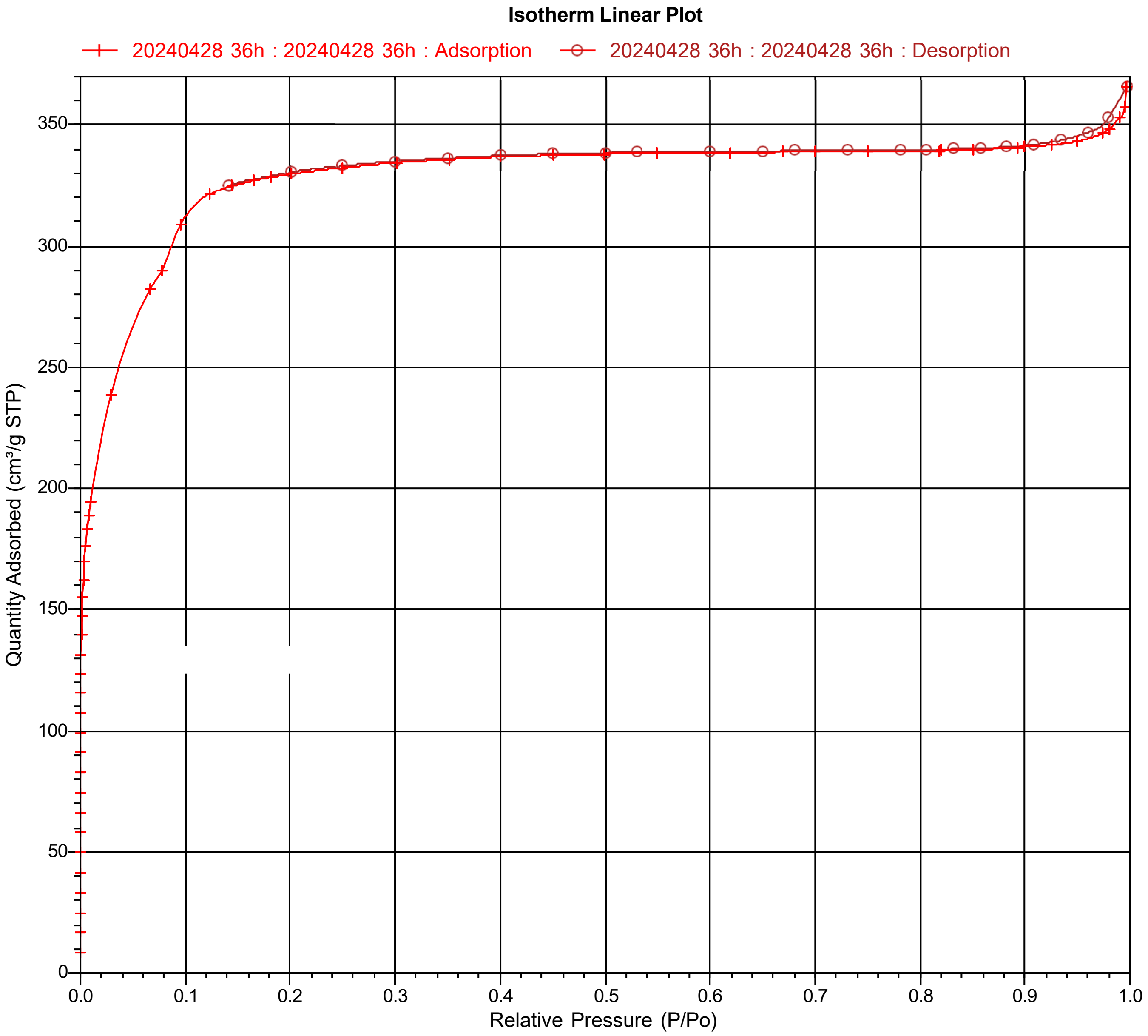

pharmaceutics

MDPI

BET Report

BET surface area: 1311.1947 ± 19.1185 m^2/g
Slope: 0.003288 ± 0.000048 g/cm^3 STP
Y-intercept: 0.000032 ± 0.000004 g/cm^3 STP
C: 105.204918
Qm: 301.2455 cm^3/g STP
Correlation coefficient: 0.9996774
Molecular cross-sectional area: 0.1620 nm^2

Relative Pressure (P/Po)	Quantity Adsorbed (cm^3/g STP)	1/[Q(Po/P - 1)]
0.028697839	238.4861	0.000124
0.066248167	282.0718	0.000252
0.077932096	290.0489	0.000291
0.096395595	309.2211	0.000345
0.122397593	321.5195	0.000434

pharmaceutics

MDPI

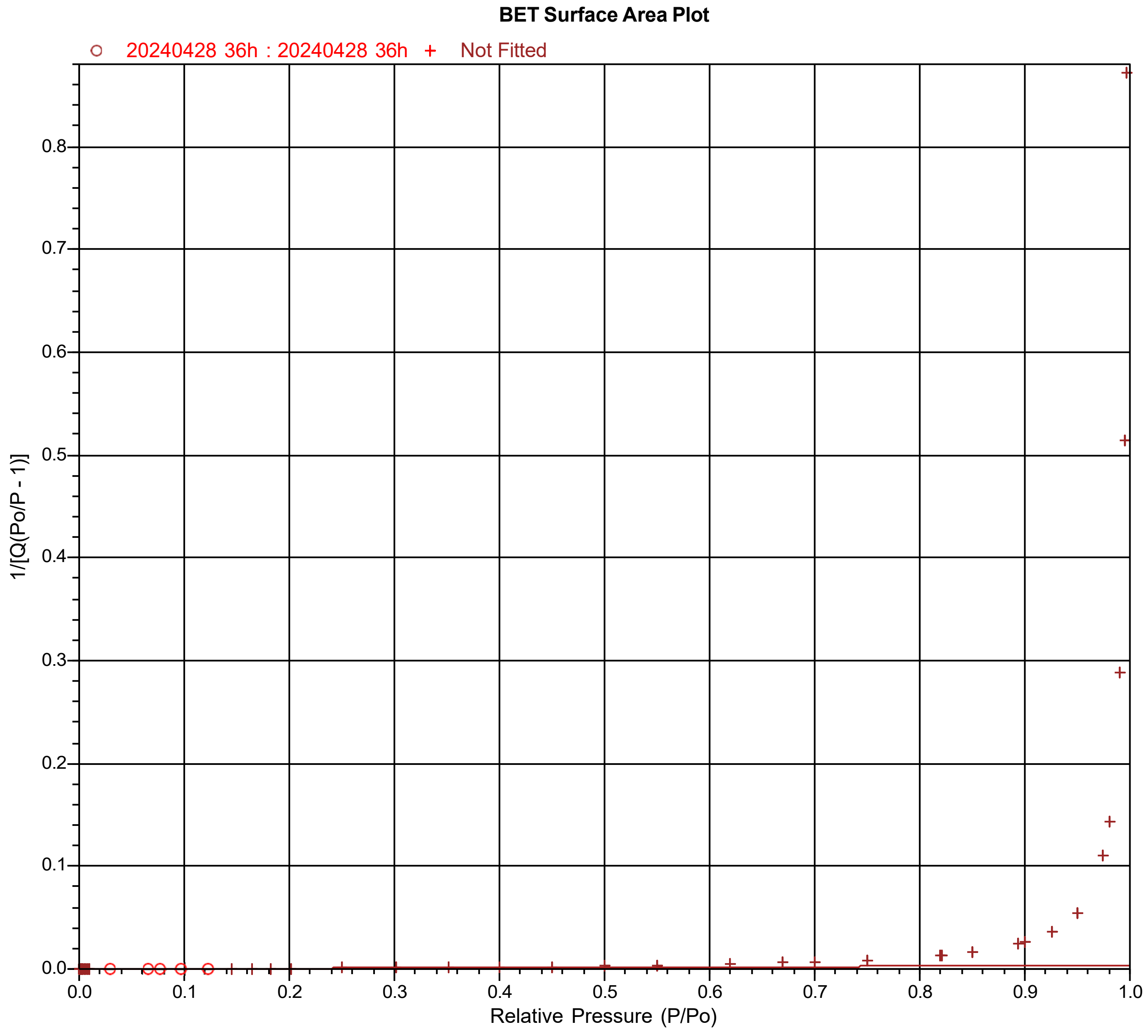

pharmaceutics

MDPI

t-Plot Report

Micropore volume: 0.373316 cm³/g
Micropore area: 999.3614 m²/g
External surface area: 311.8334 m²/g
Slope: 20.159902 ± 4.276909 cm³/g·Å STP
Y-intercept: 241.347254 ± 17.742167 cm³/g STP
Correlation coefficient: 0.903494
Surface area correction factor: 1.000
Density conversion factor: 0.0015468
Total surface area (BET): 1311.1947 m²/g
Thickness range: 3.5000 Å to 5.0000 Å
Thickness equation: Harkins and Jura

Thickness Curve

t = [13.99 / (0.034 - log(P/Po))] ^ 0.5

t-Plot Report - Data

Relative Pressure (P/Po)	Statistical Thickness (Å)	Quantity Adsorbed (cm³/g STP)	Fitted
0.000006945	1.6415	8.2804	
0.000010001	1.6671	16.5640	
0.000013394	1.6885	24.8485	
0.000017323	1.7080	33.1327	
0.000022382	1.7282	41.4146	
0.000029074	1.7496	49.6955	
0.000038444	1.7732	57.9720	
0.000051719	1.7995	66.2417	
0.000070665	1.8284	74.5030	
0.000098208	1.8605	82.7520	
0.000138865	1.8961	90.9817	
0.000196913	1.9341	99.1860	
0.000279907	1.9749	107.3610	
0.000395539	2.0176	115.4962	
0.000554900	2.0622	123.5765	
0.000773169	2.1089	131.5840	
0.001070402	2.1579	139.4874	
0.001475539	2.2097	147.2471	
0.002031136	2.2653	154.9216	
0.002779210	2.3241	162.4024	

pharmaceutics

t-Plot Report - Data

Relative Pressure (P/Po)	Statistical Thickness (Å)	Quantity Adsorbed (cm³/g STP)	Fitted
0.003756887	2.3851	169.5906	
0.004978419	2.4467	176.3872	
0.006469590	2.5086	182.9196	
0.008163579	2.5676	188.6492	
0.010196669	2.6281	194.7806	
0.028697839	2.9793	238.4861	
0.066248167	3.3963	282.0718	
0.077932096	3.4996	290.0489	
0.096395595	3.6503	309.2211	*
0.122397593	3.8451	321.5195	*
0.144787226	4.0025	324.7719	*
0.165285320	4.1412	326.9825	*
0.181894534	4.2510	328.4140	*
0.201318532	4.3774	329.7746	*
0.249269493	4.6852	332.4298	*
0.301180595	5.0199	334.4299	
0.351515415	5.3539	335.8266	
0.399861419	5.6901	336.8091	
0.449865641	6.0603	337.5112	
0.499774829	6.4601	338.0531	
0.549882705	6.9014	338.3545	
0.618864268	7.5969	338.5904	
0.669169456	8.1921	338.7852	
0.700106649	8.6073	338.9105	

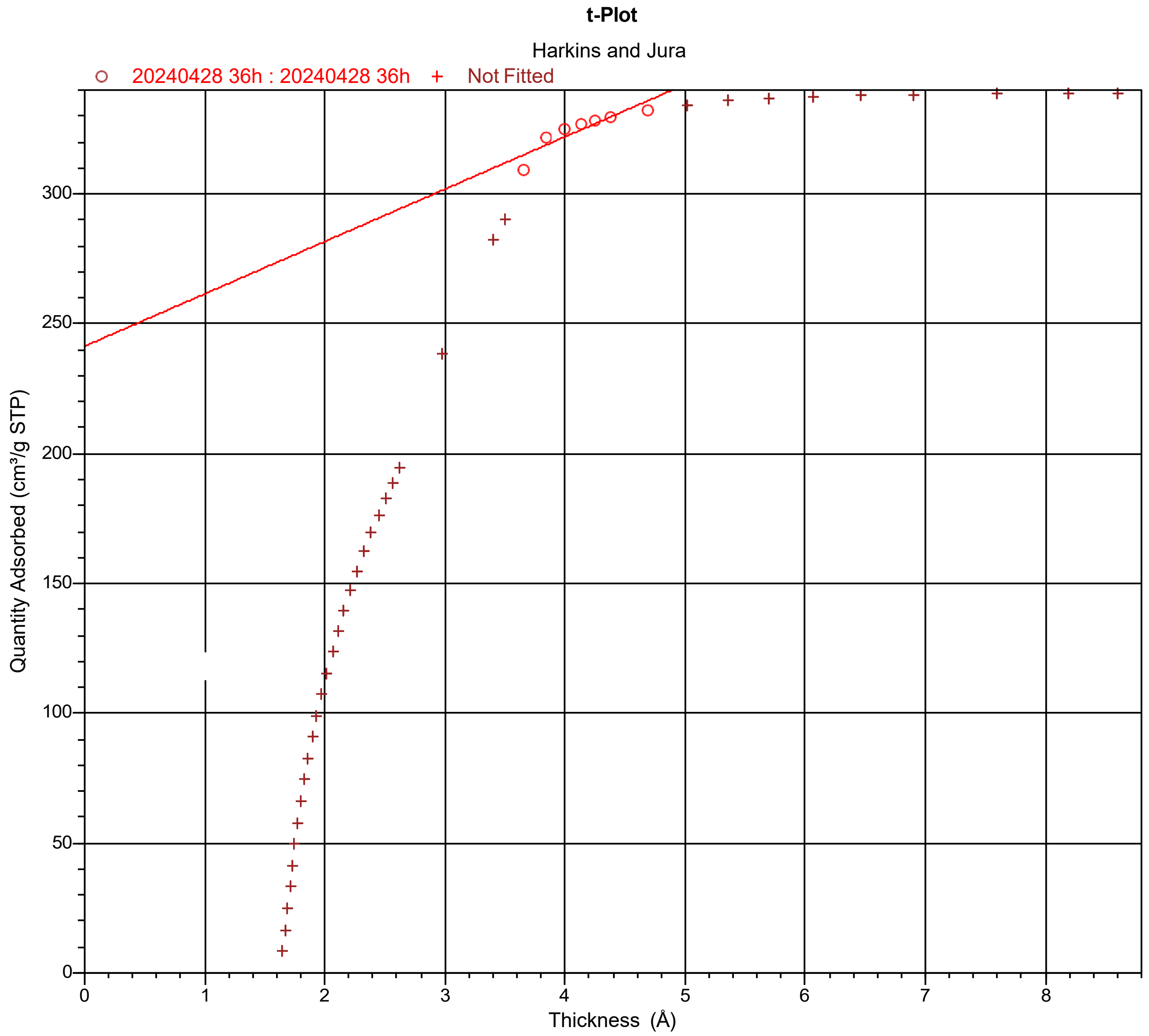
t-Plot
Harkins and Jura
20240428 36h : 20240428 36h
Not Fitted
Quantity Adsorbed (cm³/g STP)
Thickness (Å)
0
50
100
150
200
250
300
0
1
2
3
4
5
6
7
8

pharmaceutics

MDPI

BJH Adsorption Pore Distribution Report

Faas Correction

Halsey

t = 3.54 [-5 / ln(P/Po)] ^ 0.333

Width range: 17.000 Å to 3,000.000 Å

Adsorbate property factor: 9.53000 Å

Density conversion factor: 0.0015468

Fraction of pores open at both ends: 0.00

Pore Width Range (Å)	Average Width (Å)	Incremental Pore Volume (cm³/g)	Cumulative Pore Volume (cm³/g)	Incremental Pore Area (m²/g)	Cumulative Pore Area (m²/g)
3580.7 - 2004.9	2376.2	0.007264	0.007264	0.122	0.122
2004.9 - 1008.0	1204.3	0.007436	0.014700	0.247	0.369
1008.0 - 779.7	864.4	0.003156	0.017855	0.146	0.515
779.7 - 399.7	475.3	0.005538	0.023393	0.466	0.981
399.7 - 274.0	313.0	0.002610	0.026003	0.334	1.315
274.0 - 207.1	230.7	0.001300	0.027303	0.225	1.540
207.1 - 194.7	200.4	0.000660	0.027962	0.132	1.672
194.7 - 139.7	157.6	0.001214	0.029176	0.308	1.980
139.7 - 116.9	126.0	0.000340	0.029517	0.108	2.088
116.9 - 116.1	116.5	0.000571	0.030088	0.196	2.284
116.1 - 84.7	95.0	0.000507	0.030595	0.214	2.498
84.7 - 70.5	76.1	0.000021	0.030616	0.011	2.509
70.5 - 63.8	66.8	0.000197	0.030813	0.118	2.627
63.8 - 55.2	58.8	0.000334	0.031147	0.227	2.854
55.2 - 46.2	49.8	0.000426	0.031573	0.343	3.197
46.2 - 41.1	43.3	0.000775	0.032348	0.715	3.912
41.1 - 36.9	38.7	0.001655	0.034003	1.709	5.621
36.9 - 33.2	34.8	0.002301	0.036304	2.643	8.264
33.2 - 30.2	31.5	0.003489	0.039793	4.430	12.693
30.2 - 27.3	28.5	0.005296	0.045089	7.428	20.121
27.3 - 24.6	25.7	0.008112	0.053201	12.612	32.734
24.6 - 22.2	23.2	0.011554	0.064755	19.887	52.621
22.2 - 21.3	21.7	0.006160	0.070915	11.330	63.951
21.3 - 20.5	20.9	0.006740	0.077655	12.897	76.848
20.5 - 19.6	20.0	0.010840	0.088495	21.652	98.500

pharmaceutics

MDPI

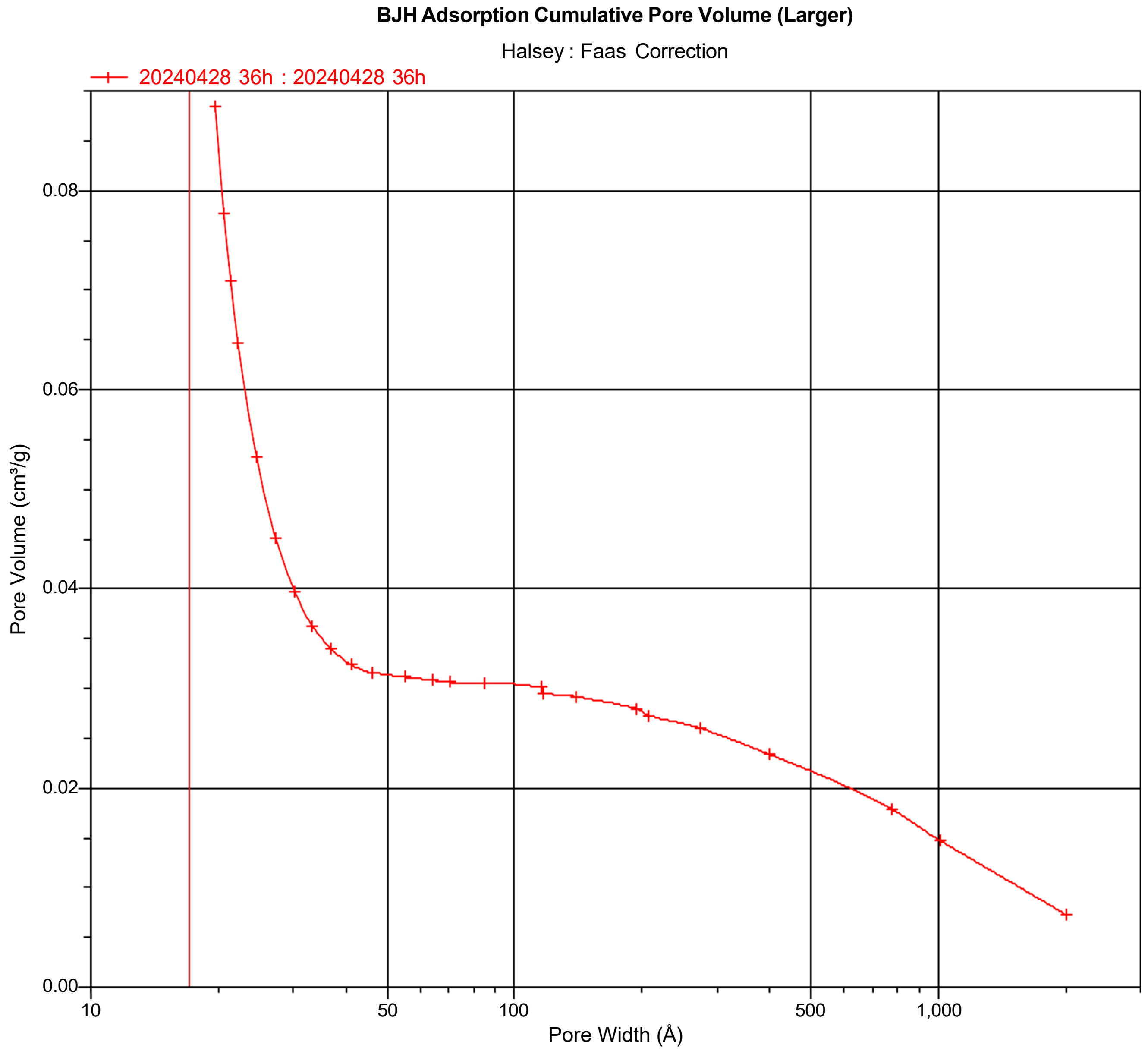

pharmaceutics

MDPI

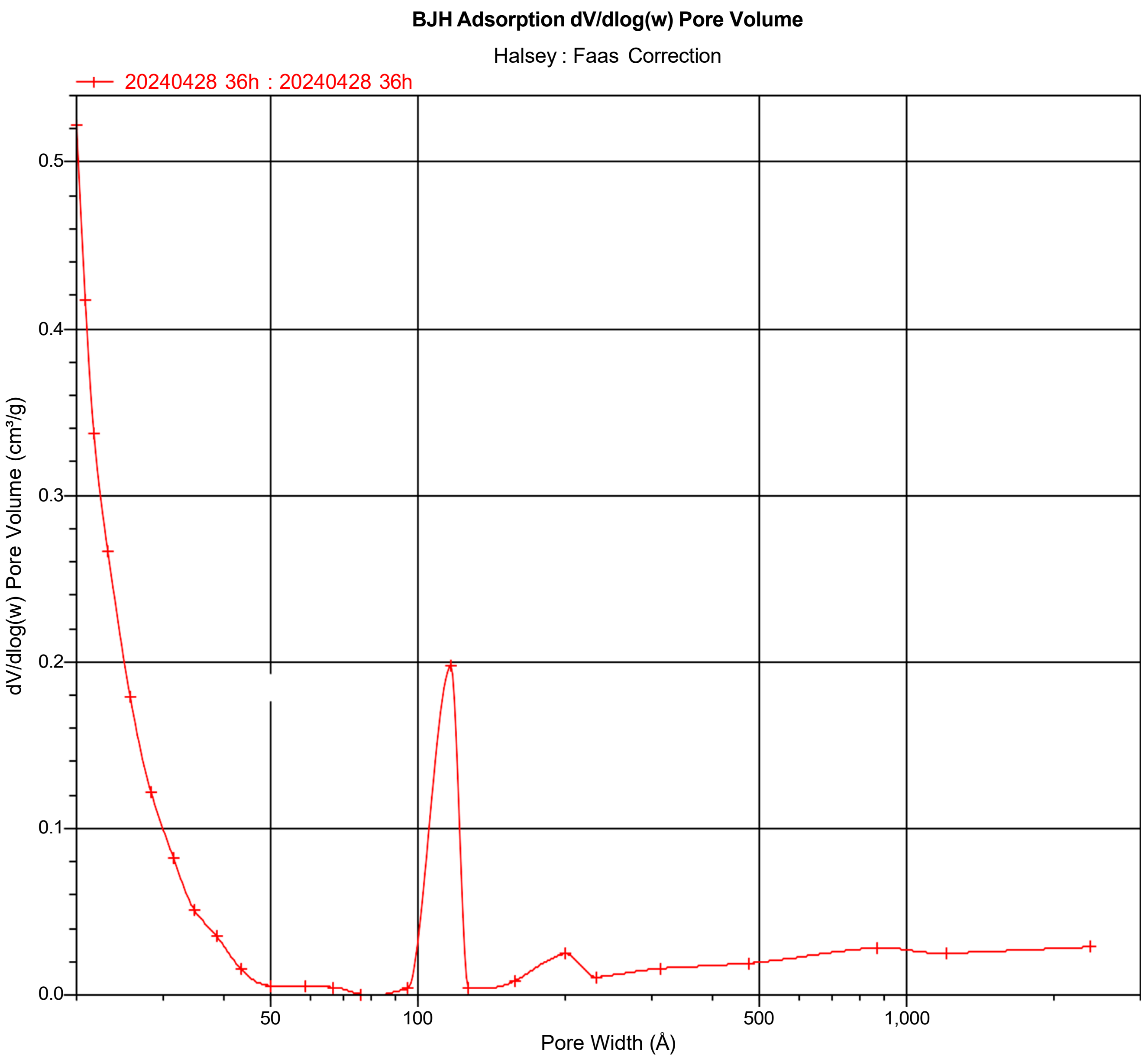

pharmaceutics

MDPI

.

BJH Desorption Pore Distribution Report

Faas Correction
Halsey
t = 3.54 [-5 / ln(P/Po)] ^ 0.333

Width range: 17.000 Å to 3,000.000 Å
Adsorbate property factor: 9.53000 Å
Density conversion factor: 0.0015468
Fraction of pores open at both ends: 0.00

Pore Width Range (Å)	Average Width (Å)	Incremental Pore Volume (cm³/g)	Cumulative Pore Volume (cm³/g)	Incremental Pore Area (m²/g)	Cumulative Pore Area (m²/g)
6166.4 - 932.2	1042.5	0.021574	0.021574	0.828	0.828
932.2 - 498.6	591.7	0.009800	0.031374	0.662	1.490
498.6 - 305.8	356.5	0.005684	0.037058	0.638	2.128
305.8 - 222.2	250.2	0.003035	0.040092	0.485	2.613
222.2 - 175.6	193.1	0.001533	0.041626	0.318	2.931
175.6 - 145.5	157.5	0.000866	0.042491	0.220	3.151
145.5 - 124.3	133.0	0.000423	0.042914	0.127	3.278
124.3 - 108.4	115.1	0.000327	0.043241	0.114	3.391
108.4 - 96.2	101.5	0.000048	0.043289	0.019	3.410
96.2 - 66.3	71.3	0.000141	0.043431	0.079	3.490
66.3 - 60.3	62.9	0.000252	0.043683	0.160	3.650
60.3 - 44.1	47.4	0.000449	0.044133	0.379	4.029
44.1 - 41.2	42.5	0.000821	0.044954	0.772	4.801
41.2 - 36.9	38.8	0.001377	0.046331	1.421	6.222
36.9 - 33.2	34.8	0.002429	0.048760	2.788	9.011
33.2 - 30.1	31.4	0.003500	0.052260	4.453	13.464
30.1 - 27.2	28.4	0.004976	0.057236	6.998	20.461
27.2 - 24.6	25.7	0.007733	0.064969	12.022	32.483
24.6 - 22.2	23.2	0.011955	0.076924	20.595	53.078
22.2 - 19.4	20.5	0.025421	0.102345	49.542	102.620

pharmaceutics

MDPI

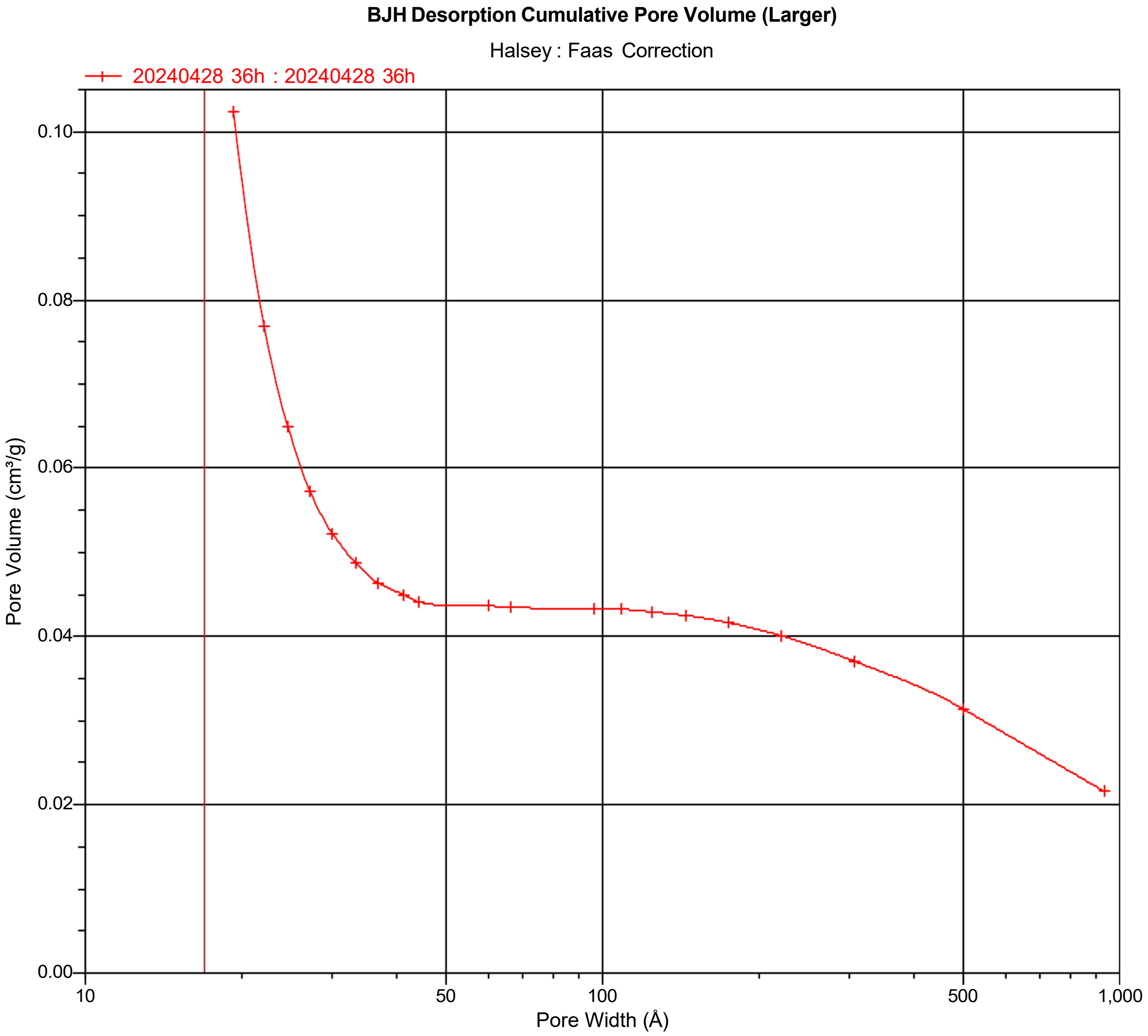

pharmaceutics
MDPI

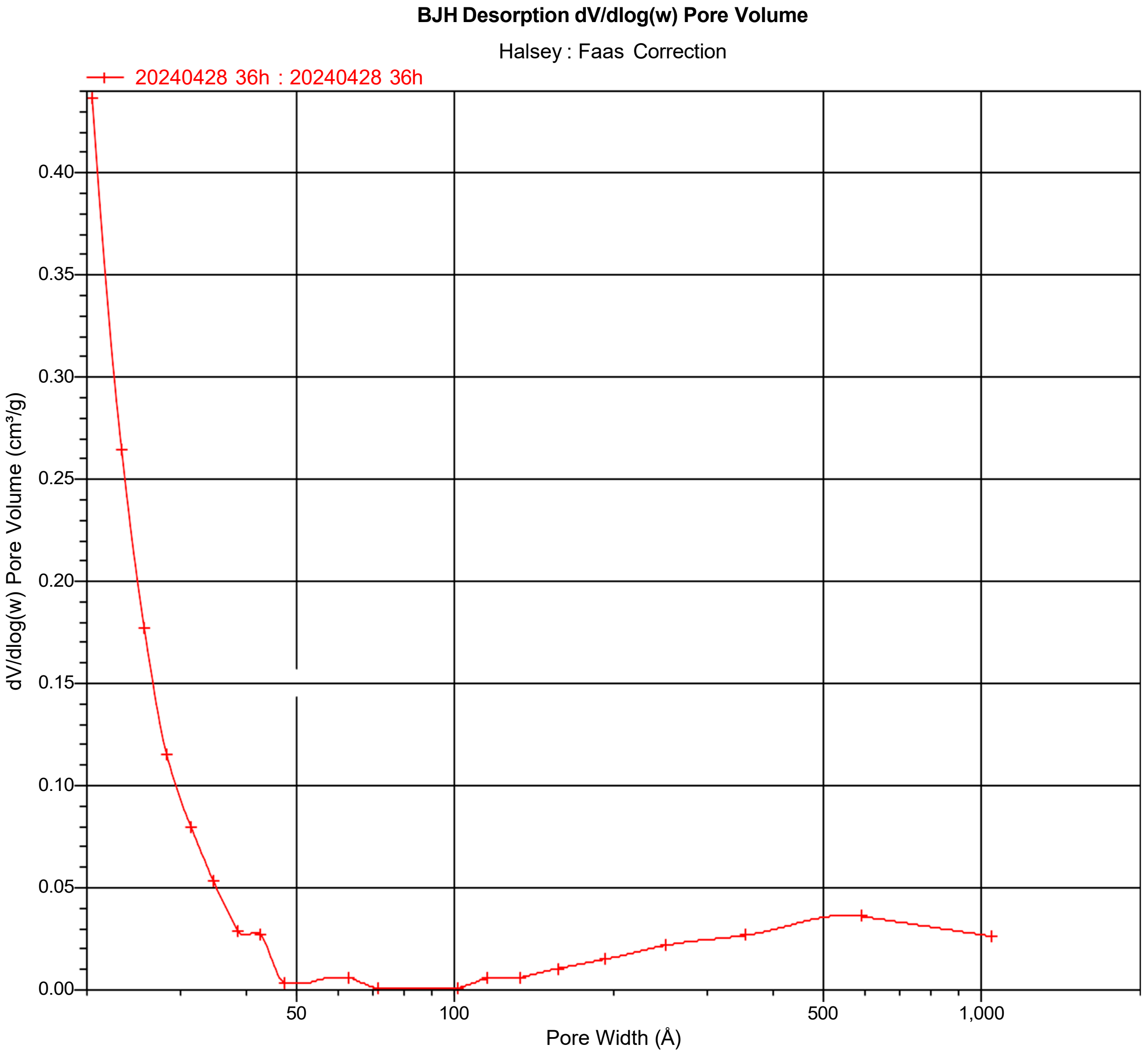

Porosity Distribution by
Model: N2 - Tarazona NLDFT, Esf = 30.0K
Method: Non-negative Regularization: 0.01000
Standard Deviation of Fit: 4.10874 cm³/g STP

Volume in Pores	<	5.22 Å	:	0.00070 cm³/g
Total Volume in Pores	<=	448.83 Å	:	0.55307 cm³/g
Total Area in Pores	>=	5.22 Å	:	2,244.262 m²/g

Pore Size Table

Pore Width (Å)	Cumulative Volume (cm³/g)	Incremental Volume (cm³/g)	Cumulative Area (m²/g)	Incremental Area (m²/g)
5.22	0.00877	0.00806	61.791	61.791
5.93	0.03382	0.02505	230.659	168.868
6.29	0.07902	0.04521	518.079	287.420
6.65	0.13850	0.05947	875.877	357.798
7.36	0.19955	0.06106	1207.555	331.678
7.72	0.24307	0.04351	1432.973	225.418
8.44	0.26620	0.02313	1542.646	109.673
8.79	0.27749	0.01130	1594.036	51.389
9.51	0.28945	0.01195	1644.311	50.275
10.22	0.30480	0.01536	1704.397	60.087
10.94	0.31971	0.01491	1758.916	54.518
11.65	0.32878	0.00907	1790.054	31.139
12.37	0.32878	0.00000	1790.054	0.000
13.08	0.32878	0.00000	1790.054	0.000
13.80	0.32878	0.00000	1790.054	0.000
14.87	0.32878	0.00000	1790.054	0.000
15.59	0.33349	0.00470	1802.125	12.070
16.66	0.35208	0.01859	1846.777	44.652
17.73	0.38584	0.03376	1922.946	76.169
18.44	0.43209	0.04625	2023.241	100.294
19.87	0.47591	0.04382	2111.424	88.184
20.95	0.51291	0.03701	2182.088	70.664
22.02	0.53835	0.02544	2228.297	46.209
23.45	0.54741	0.00906	2243.758	15.461
24.52	0.54741	0.00000	2243.758	0.000
25.95	0.54741	0.00000	2243.758	0.000
27.38	0.54741	0.00000	2243.758	0.000

pharmaceutics

MDPI

Pore Size Table

Pore Width (Å)	Cumulative Volume (cm³/g)	Incremental Volume (cm³/g)	Cumulative Area (m²/g)	Incremental Area (m²/g)
29.17	0.54741	0.00000	2243.758	0.000
30.60	0.54741	0.00000	2243.758	0.000
32.39	0.54741	0.00000	2243.758	0.000
34.17	0.54741	0.00000	2243.758	0.000
35.96	0.54741	0.00000	2243.758	0.000
38.11	0.54741	0.00000	2243.758	0.000
40.25	0.54741	0.00000	2243.758	0.000
42.39	0.54741	0.00000	2243.758	0.000
44.54	0.54741	0.00000	2243.758	0.000
47.04	0.54741	0.00000	2243.758	0.000
49.54	0.54741	0.00000	2243.758	0.000
52.05	0.54741	0.00000	2243.758	0.000
54.91	0.54741	0.00000	2243.758	0.000
57.77	0.54741	0.00000	2243.758	0.000
60.98	0.54741	0.00000	2243.758	0.000
64.20	0.54741	0.00000	2243.758	0.000
67.42	0.54741	0.00000	2243.758	0.000
70.99	0.54741	0.00000	2243.758	0.000
74.57	0.54741	0.00000	2243.758	0.000
78.50	0.54741	0.00000	2243.758	0.000
82.79	0.54741	0.00000	2243.758	0.000
87.08	0.54741	0.00000	2243.758	0.000
91.37	0.54741	0.00000	2243.758	0.000
96.37	0.54741	0.00000	2243.758	0.000
101.38	0.54741	0.00000	2243.758	0.000
106.38	0.54741	0.00000	2243.758	0.000
112.10	0.54741	0.00000	2243.758	0.000
117.82	0.54741	0.00000	2243.758	0.000
123.90	0.54741	0.00000	2243.758	0.000
130.33	0.54741	0.00000	2243.758	0.000
136.76	0.54741	0.00000	2243.758	0.000
143.91	0.54741	0.00000	2243.758	0.000
151.06	0.54741	0.00000	2243.758	0.000
158.93	0.54741	0.00000	2243.758	0.000
167.15	0.54741	0.00000	2243.758	0.000
175.73	0.54741	0.00000	2243.758	0.000

pharmaceutics

MDPI

Pore Size Table

Pore Width (Å)	Cumulative Volume (cm^3/g)	Incremental Volume (cm^3/g)	Cumulative Area (m^2/g)	Incremental Area (m^2/g)
184.66	0.54741	0.00000	2243.758	0.000
193.96	0.54741	0.00000	2243.758	0.000
203.97	0.54741	0.00000	2243.758	0.000
214.33	0.54741	0.00000	2243.758	0.000
225.06	0.54741	0.00000	2243.758	0.000
236.50	0.54741	0.00000	2243.758	0.000
248.29	0.54741	0.00000	2243.758	0.000
261.16	0.54741	0.00000	2243.758	0.000
274.39	0.54741	0.00000	2243.758	0.000
287.97	0.54741	0.00000	2243.758	0.000
302.63	0.54741	0.00000	2243.758	0.000
318.00	0.54741	0.00000	2243.758	0.000
334.08	0.54741	0.00000	2243.758	0.000
350.88	0.54741	0.00000	2243.758	0.000
368.76	0.54741	0.00000	2243.758	0.000
387.34	0.54741	0.00000	2243.758	0.000
406.65	0.54741	0.00000	2243.758	0.000
427.38	0.54741	0.00000	2243.758	0.000
448.83	0.55307	0.00566	2244.262	0.504

pharmaceutics

MDPI

Porosity Distribution by
Model: N2 - Tarazona NLDFT, Esf = 30.0K
Method: Non-negative Regularization: 0.01000

Standard Deviation of Fit: 4.10874 cm³/g STP

Isotherm Table

Relative Pressure (P/Po)	Experimental Quantity Adsorbed (cm³/g STP)	Fitted Quantity Adsorbed (cm³/g STP)	Absolute Residual (cm³/g STP)	Relative Residual
0.000007943	11.0733	22.8590	-11.7857	-1.064340
0.000010000	16.5623	26.2667	-9.7044	-0.585936
0.000012589	22.9627	30.0130	-7.0503	-0.307031
0.000015849	30.2218	33.9152	-3.6934	-0.122211
0.000019953	37.7437	37.7360	0.0077	0.000203
0.000025119	45.1074	41.5584	3.5490	0.078679
0.000031623	52.2216	45.7953	6.4263	0.123058
0.000039811	58.9374	51.3061	7.6313	0.129482
0.000050119	65.4116	58.4504	6.9612	0.106422
0.000063096	71.5646	66.9612	4.6034	0.064325
0.000079433	77.4468	75.7995	1.6474	0.021271
0.000100000	83.1671	81.7851	1.3820	0.016617
0.000125892	88.6953	87.7696	0.9257	0.010437
0.000158490	94.0449	96.4374	-2.3925	-0.025440
0.000199526	99.4781	103.2872	-3.8092	-0.038291
0.000251188	104.8570	107.6811	-2.8241	-0.026933
0.000316228	110.1702	111.7630	-1.5928	-0.014458
0.000398107	115.6430	117.8274	-2.1844	-0.018889
0.000501187	121.1500	121.7775	-0.6275	-0.005180
0.000630958	126.6343	127.8971	-1.2628	-0.009972
0.000794328	132.2173	132.2205	-0.0032	-0.000024
0.001000000	137.8593	138.1323	-0.2730	-0.001980
0.001258925	143.4024	142.4956	0.9068	0.006324
0.001584895	148.9294	147.4667	1.4627	0.009821
0.001995263	154.5100	151.2647	3.2453	0.021004
0.002511882	160.0057	155.1776	4.8280	0.030174
0.003162276	165.4515	159.7122	5.7393	0.034689
0.003981066	170.9460	164.0643	6.8817	0.040257
0.005011868	176.5479	169.4428	7.1051	0.040245

pharmaceutics

MDPI

Isotherm Table

Relative Pressure (P/Po)	Experimental Quantity Adsorbed (cm³/g STP)	Fitted Quantity Adsorbed (cm³/g STP)	Absolute Residual (cm³/g STP)	Relative Residual
0.006309579	182.3223	177.4230	4.8993	0.026872
0.007943276	187.9469	190.1183	-2.1713	-0.011553
0.010000000	194.2145	203.7704	-9.5558	-0.049202
0.012355640	200.8788	215.0817	-14.2029	-0.070704
0.015186320	208.5824	222.4255	-13.8431	-0.066368
0.018485530	217.0487	227.7313	-10.6826	-0.049218
0.022294740	225.9857	232.8058	-6.8201	-0.030179
0.026653420	234.8797	236.7424	-1.8627	-0.007930
0.031598160	243.1823	243.1271	0.0552	0.000227
0.037162240	251.4338	246.6231	4.8107	0.019133
0.043374470	259.5747	255.7431	3.8316	0.014761
0.050259210	267.4415	269.0085	-1.5670	-0.005859
0.057835260	274.9173	272.4621	2.4552	0.008931
0.066115920	281.9670	275.6095	6.3575	0.022547
0.075109080	287.9135	288.2068	-0.2933	-0.001019
0.084815920	297.1488	299.4507	-2.3019	-0.007747
0.095232370	308.3005	302.0171	6.2834	0.020381
0.106348200	315.6794	310.7409	4.9385	0.015644
0.118147500	320.5811	315.2181	5.3630	0.016729
0.130609100	322.8463	317.0871	5.7592	0.017839
0.143706600	324.6503	318.7838	5.8665	0.018070
0.157410500	326.1827	320.3361	5.8466	0.017924
0.171685500	327.5644	321.7640	5.8005	0.017708
0.186492100	328.7595	323.0828	5.6766	0.017267
0.201792100	329.8044	324.3055	5.4989	0.016673
0.217539500	330.7613	325.4420	5.3194	0.016082
0.233689500	331.6617	326.5010	5.1607	0.015560
0.250196100	332.4720	327.4897	4.9823	0.014986
0.267011800	333.1981	328.4146	4.7835	0.014356
0.284089500	333.8559	329.2812	4.5747	0.013703
0.301380300	334.4361	330.0941	4.3421	0.012983
0.318838200	334.9633	330.8577	4.1056	0.012257
0.336417100	335.4529	331.5757	3.8772	0.011558
0.354071100	335.8854	332.2516	3.6338	0.010819
0.371757900	336.2783	332.8884	3.3900	0.010081

pharmaceutics

MDPI

Isotherm Table

Relative Pressure (P/Po)	Experimental Quantity Adsorbed (cm³/g STP)	Fitted Quantity Adsorbed (cm³/g STP)	Absolute Residual (cm³/g STP)	Relative Residual
0.389435500	336.6303	333.4889	3.1414	0.009332
0.407065800	336.9218	334.0557	2.8661	0.008507
0.424610500	337.1797	334.5912	2.5885	0.007677
0.442034200	337.4132	335.0976	2.3157	0.006863
0.459305300	337.6262	335.5771	2.0491	0.006069
0.476393400	337.8249	336.0324	1.7925	0.005306
0.493271100	337.9966	336.4688	1.5278	0.004520
0.509911800	338.1320	336.8923	1.2397	0.003666
0.526293400	338.2411	337.2835	0.9576	0.002831
0.542394700	338.3244	337.6509	0.6735	0.001991
0.558200000	338.3840	337.9979	0.3861	0.001141
0.573690800	338.4359	338.3263	0.1096	0.000324
0.588853900	338.4853	338.6374	-0.1520	-0.000449
0.603677600	338.5351	338.9322	-0.3971	-0.001173
0.618153900	338.5876	339.2118	-0.6242	-0.001843
0.632272400	338.6420	339.4771	-0.8351	-0.002466
0.646028900	338.6952	339.7290	-1.0338	-0.003052
0.659417100	338.7472	339.9682	-1.2211	-0.003605
0.672435500	338.7981	340.1956	-1.3974	-0.004125
0.685081600	338.8495	340.4117	-1.5622	-0.004610
0.697355300	338.8996	340.6173	-1.7178	-0.005069
0.709256600	338.9412	340.8130	-1.8718	-0.005523
0.720789500	338.9677	340.9998	-2.0321	-0.005995
0.731953900	338.9864	341.1784	-2.1919	-0.006466
0.742756600	339.0038	341.3505	-2.3467	-0.006922
0.753200000	339.0252	341.5266	-2.5013	-0.007378
0.763289500	339.0553	341.6963	-2.6410	-0.007789
0.773030300	339.0938	341.8467	-2.7529	-0.008118
0.782430300	339.1400	341.9877	-2.8477	-0.008397
0.791496100	339.1928	342.1210	-2.9282	-0.008633
0.800232900	339.2515	342.2475	-2.9960	-0.008831
0.808648700	339.3153	342.3676	-3.0523	-0.008996
0.816752600	339.3834	342.4819	-3.0986	-0.009130
0.824552600	339.7143	342.5908	-2.8765	-0.008467
0.832053900	339.7679	342.6946	-2.9267	-0.008614

Isotherm Table

Relative Pressure (P/Po)	Experimental Quantity Adsorbed (cm³/g STP)	Fitted Quantity Adsorbed (cm³/g STP)	Absolute Residual (cm³/g STP)	Relative Residual
0.839267100	339.7997	342.7938	-2.9941	-0.008811
0.846200000	339.8491	342.8888	-3.0397	-0.008944
0.852860500	339.9402	342.9801	-3.0399	-0.008942
0.859257900	340.0303	343.0689	-3.0386	-0.008936
0.865398700	340.1132	343.1608	-3.0476	-0.008960
0.871292100	340.1957	343.2671	-3.0714	-0.009028
0.876947400	340.2829	343.3476	-3.0647	-0.009006
0.882369700	340.3788	343.4218	-3.0430	-0.008940
0.887569700	340.4865	343.4916	-3.0052	-0.008826
0.892553900	340.6077	343.5579	-2.9501	-0.008661
0.897328900	340.8261	343.6209	-2.7948	-0.008200
0.901905300	341.0597	343.6813	-2.6216	-0.007687
0.906286800	341.2104	343.7392	-2.5288	-0.007411
0.910484200	341.3317	343.7954	-2.4636	-0.007218
0.914501300	341.4391	343.8510	-2.4119	-0.007064
0.918347400	341.5450	343.9345	-2.3895	-0.006996
0.922026300	341.6587	343.9971	-2.3385	-0.006844
0.925547400	341.7872	344.0475	-2.2603	-0.006613
0.928915800	341.9398	344.0943	-2.1544	-0.006301
0.932136800	342.1143	344.1386	-2.0243	-0.005917
0.935218400	342.3026	344.1813	-1.8788	-0.005489
0.938163200	342.4978	347.3108	-4.8130	-0.014053
0.940978900	342.6950	347.3439	-4.6489	-0.013566
0.943669700	342.8899	347.3755	-4.4856	-0.013082
0.946242100	343.0792	347.4055	-4.3263	-0.012610
0.948700000	343.2605	347.4341	-4.1735	-0.012158
0.951048700	343.4381	347.4614	-4.0232	-0.011715
0.953292100	343.6280	347.4873	-3.8594	-0.011231
0.955435500	343.8295	347.5120	-3.6825	-0.010710
0.957482900	344.0410	347.5357	-3.4947	-0.010158
0.959438200	344.2607	347.5581	-3.2974	-0.009578
0.961305300	344.4871	347.5795	-3.0923	-0.008977
0.963088200	344.7190	347.5999	-2.8809	-0.008357
0.964789500	344.9547	347.6192	-2.6645	-0.007724
0.966414500	345.1934	347.6377	-2.4444	-0.007081

Isotherm Table

Relative Pressure (P/Po)	Experimental Quantity Adsorbed (cm³/g STP)	Fitted Quantity Adsorbed (cm³/g STP)	Absolute Residual (cm³/g STP)	Relative Residual
0.967965800	345.4338	347.6554	-2.2216	-0.006431
0.969447400	345.6752	347.6722	-1.9970	-0.005777
0.970860500	345.9162	347.6882	-1.7720	-0.005123
0.972209200	346.1563	347.7034	-1.5472	-0.004470
0.973496100	346.3946	347.7179	-1.3233	-0.003820
0.974725000	346.6321	347.7318	-1.0997	-0.003172
0.975897400	346.9236	347.7450	-0.8213	-0.002367
0.977015800	347.2718	347.7575	-0.4857	-0.001399
0.978082900	347.6459	347.7695	-0.1236	-0.000356
0.979101300	348.0220	347.7809	0.2411	0.000693
0.980072400	348.3816	347.7917	0.5899	0.001693
0.980998700	348.7214	347.8021	0.9193	0.002636
0.981882900	349.0691	347.8120	1.2571	0.003601
0.982726300	349.4219	347.8214	1.6006	0.004581
0.983530300	349.7750	347.8303	1.9446	0.005560
0.984297400	350.1248	347.8389	2.2859	0.006529
0.985028900	350.4683	347.8470	2.6213	0.007479
0.985727600	350.8039	347.8548	2.9491	0.008407
0.986392100	351.1283	347.8621	3.2662	0.009302
0.987027600	351.4422	347.8692	3.5730	0.010167
0.987632900	351.7434	347.8759	3.8675	0.010995
0.988209200	352.0312	347.8823	4.1489	0.011786
0.988760500	352.3067	347.8884	4.4183	0.012541
0.989285500	352.5685	347.8942	4.6742	0.013258
0.989785500	352.8167	347.8997	4.9170	0.013936
0.990263200	353.0524	347.9050	5.1473	0.014580
0.990718400	353.2177	347.9100	5.3076	0.015026
0.991151300	353.3019	347.9148	5.3871	0.015248
0.991565800	353.3646	347.9194	5.4452	0.015410
0.991959200	353.4499	347.9237	5.5262	0.015635

pharmaceutics

MDPI

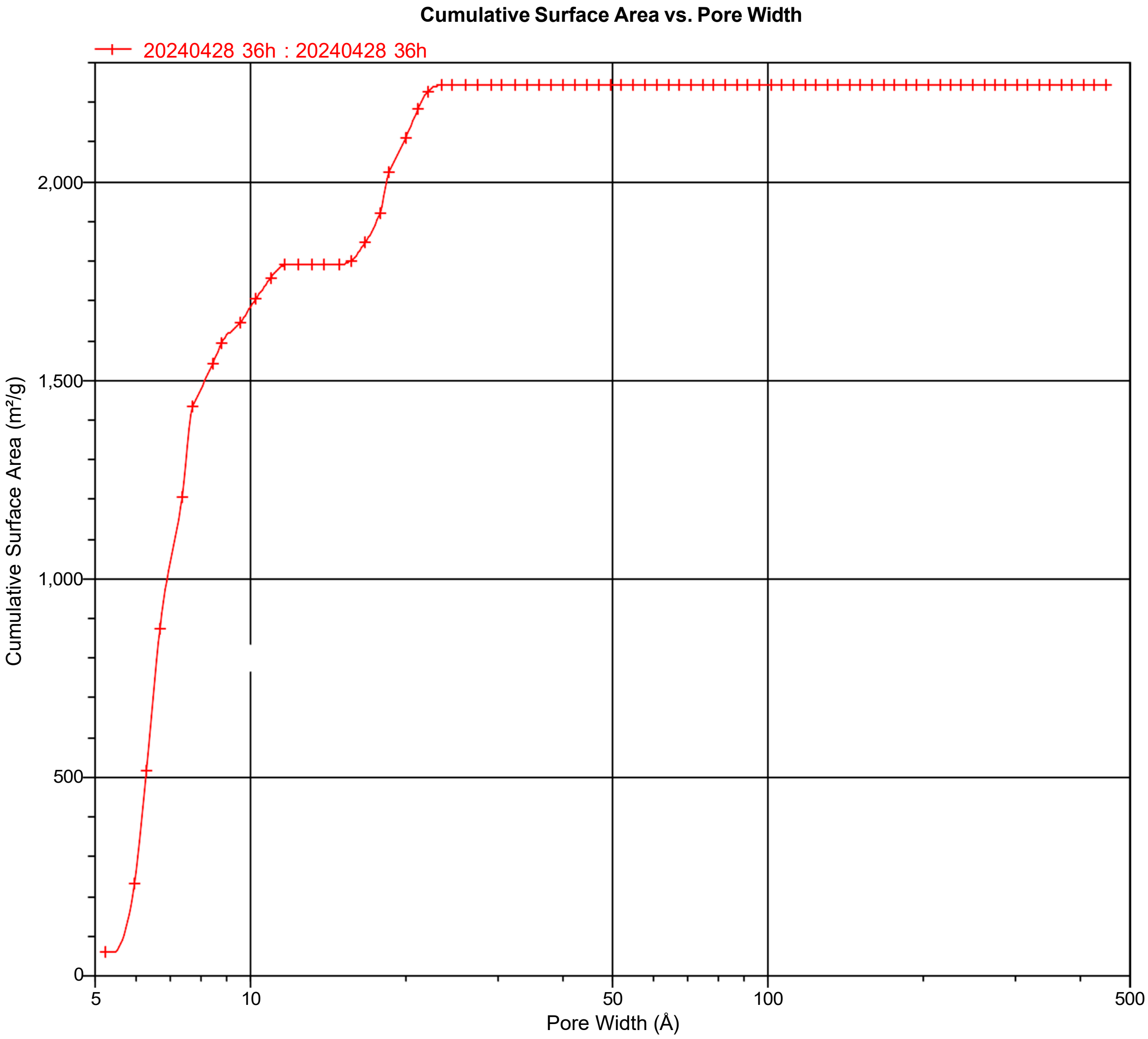

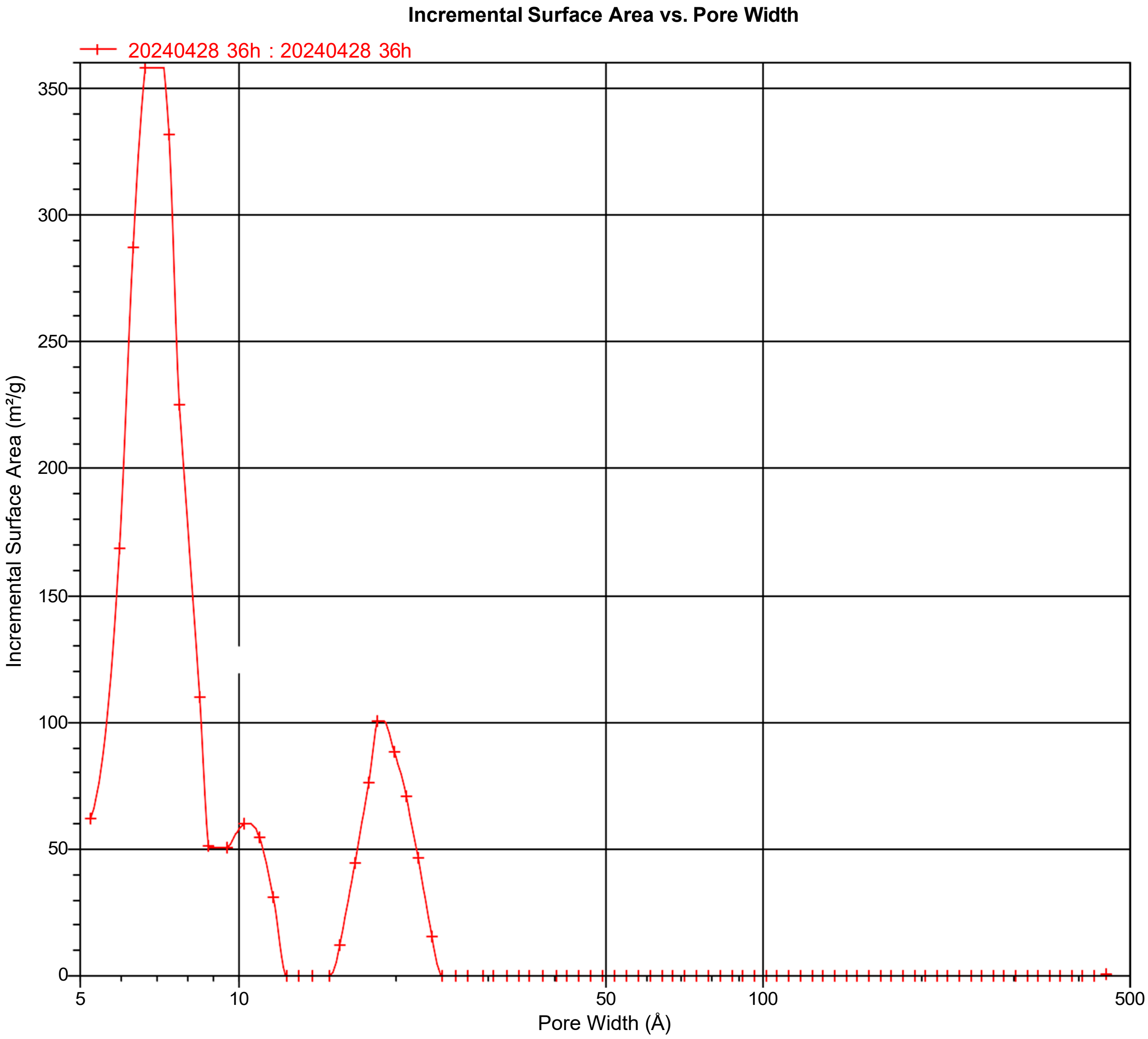
Incremental Surface Area vs. Pore Width
20240428 36h : 20240428 36h
Incremental Surface Area (m²/g)
350
300
250
200
150
100
50
0
5
10
50
100
500
Pore Width (Å)

pharmaceutics

MDPI

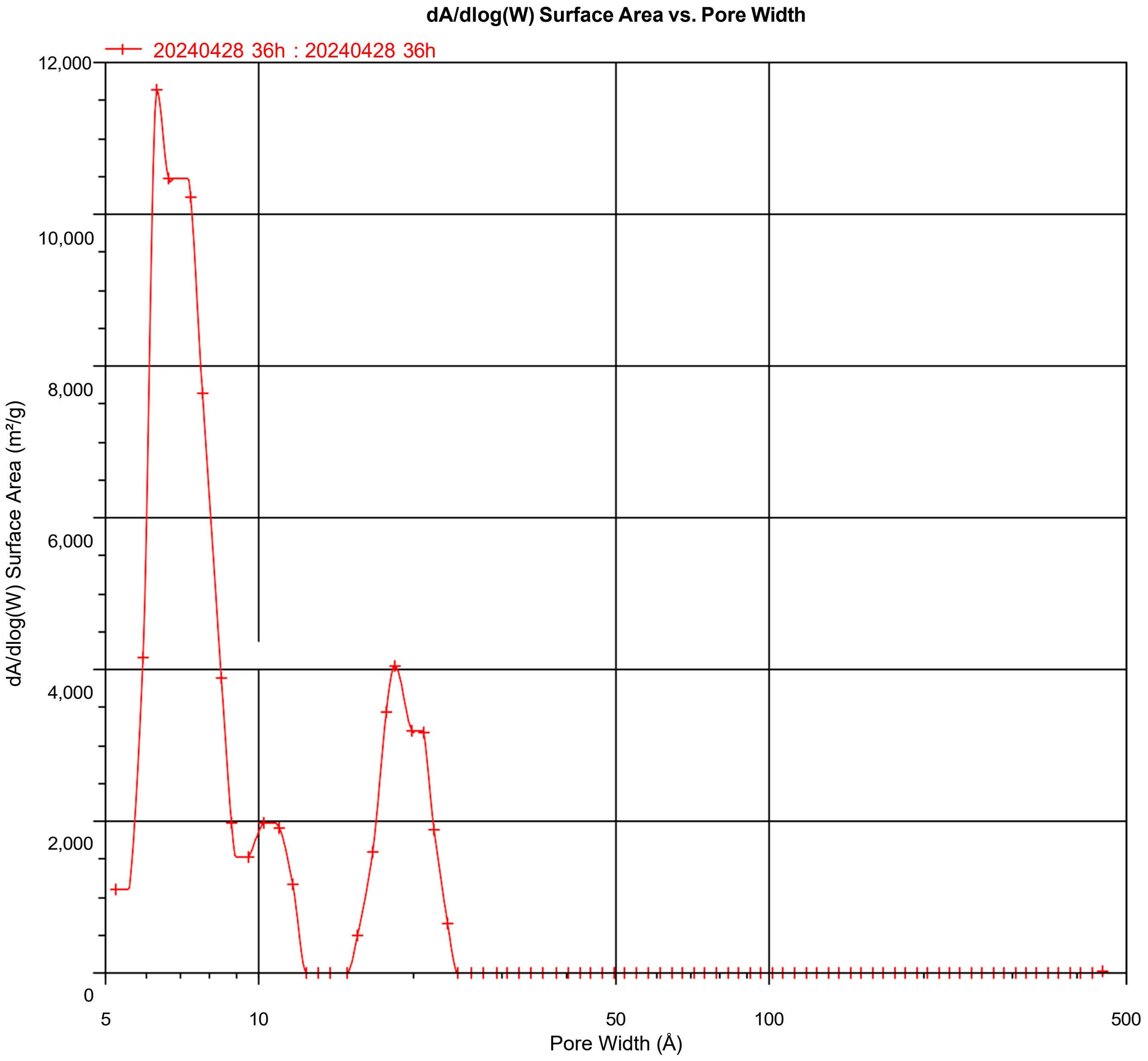

pharmaceutics

MDPI

.

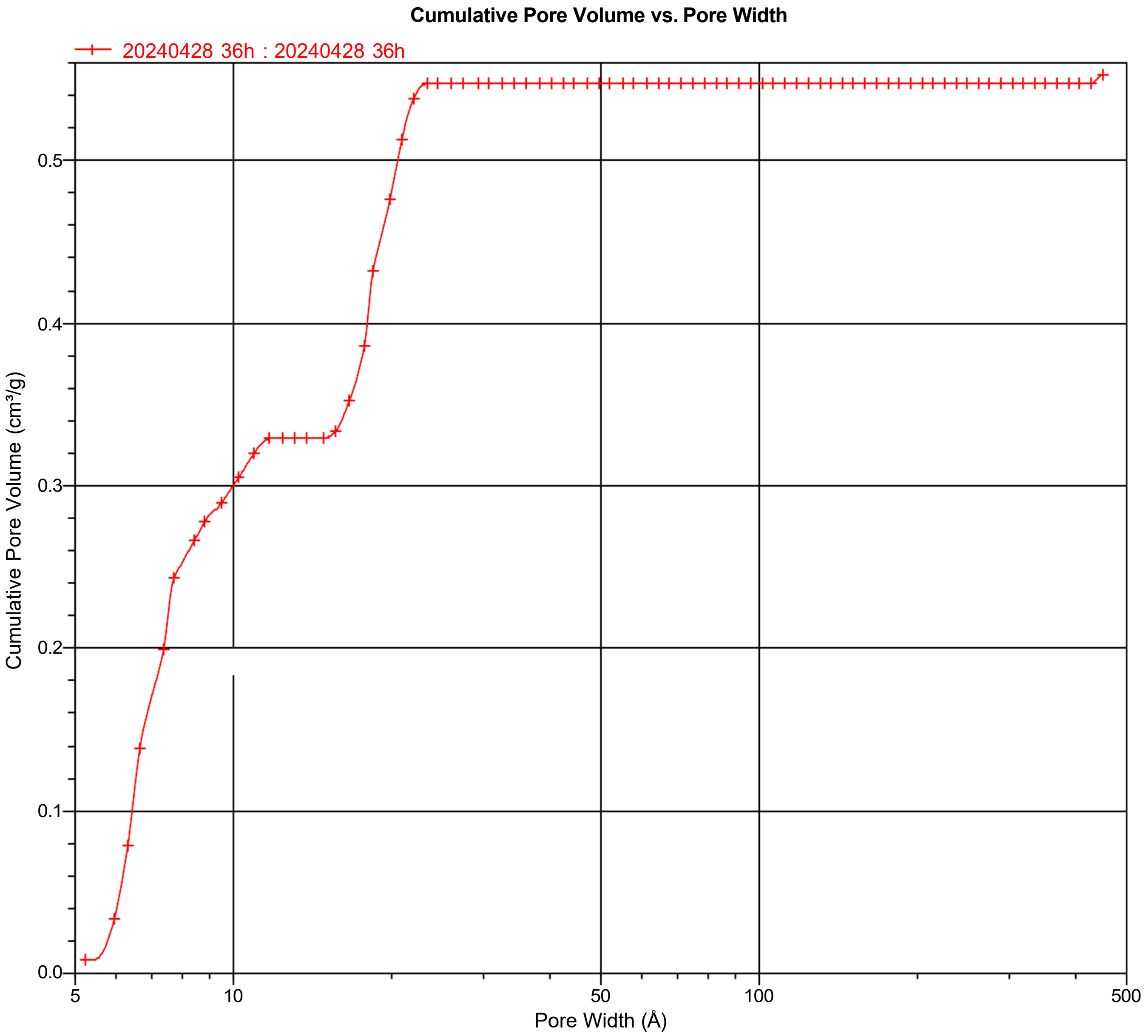

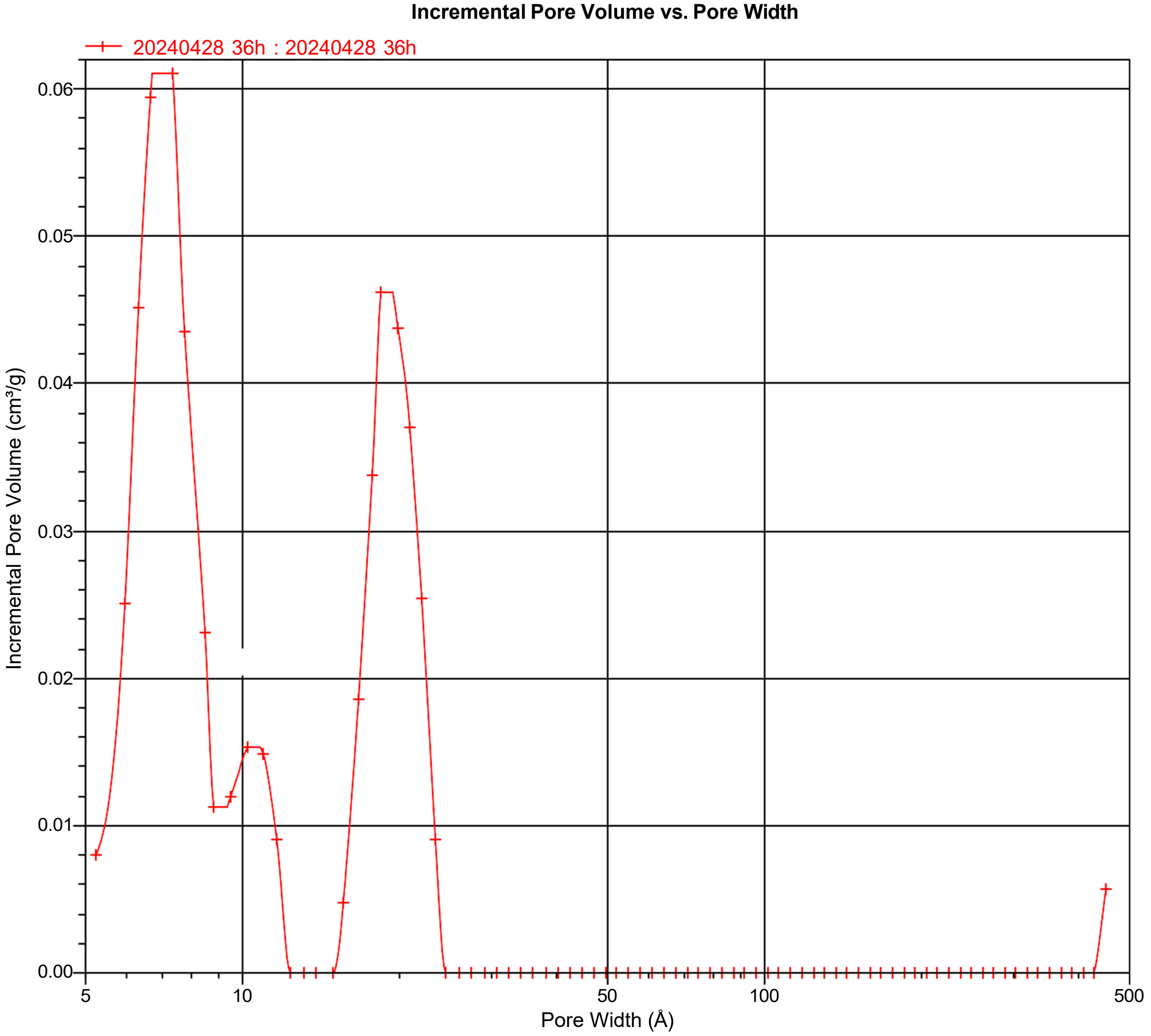
Incremental Pore Volume vs. Pore Width
20240428 36h : 20240428 36h
Incremental Pore Volume (cm³/g)
0.06
0.05
0.04
0.03
0.02
0.01
0.00
5
10
50
100
500
Pore Width (Å)

pharmaceutics

MDPI

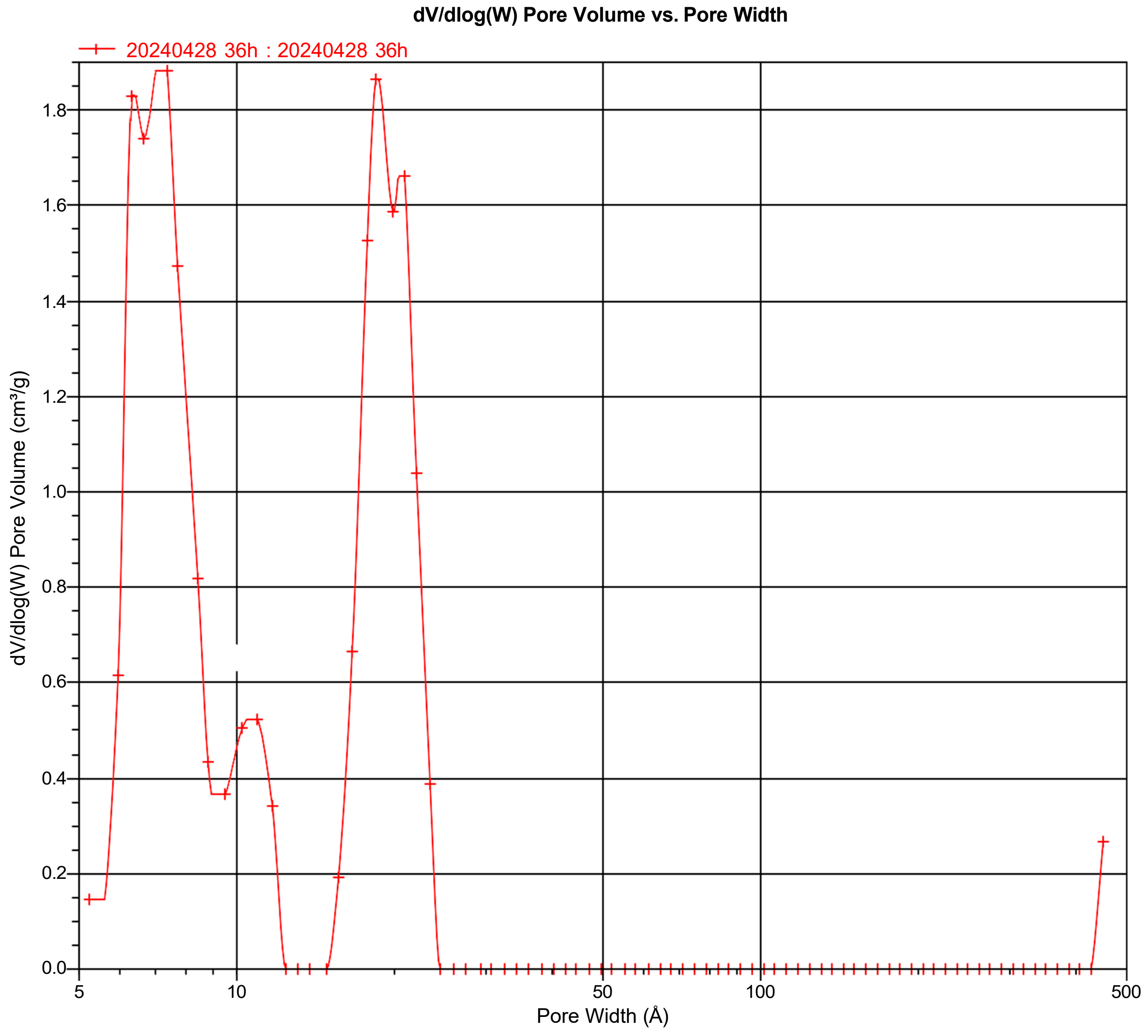

pharmaceutics

MDPI

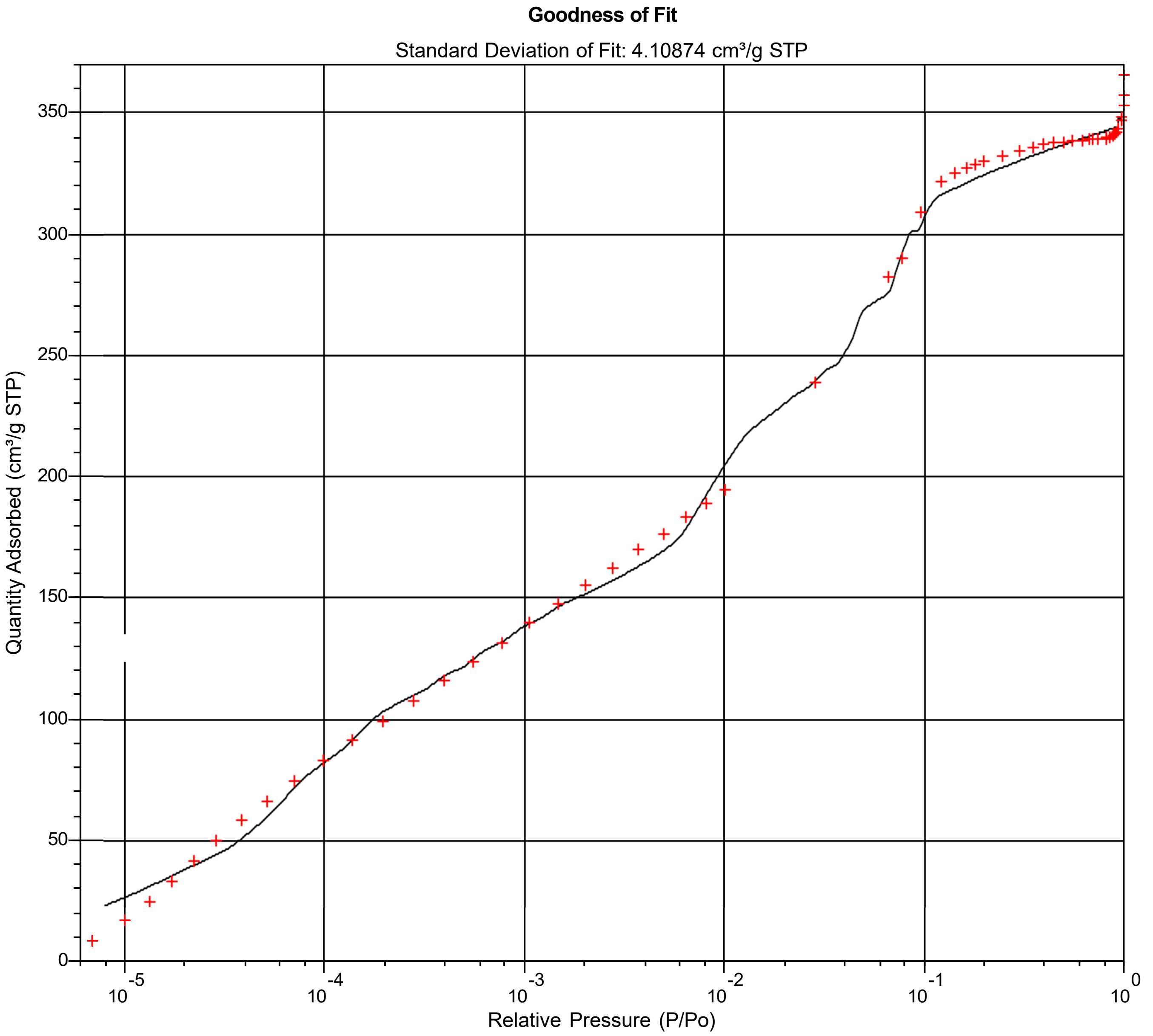

pharmaceutics

MDPI

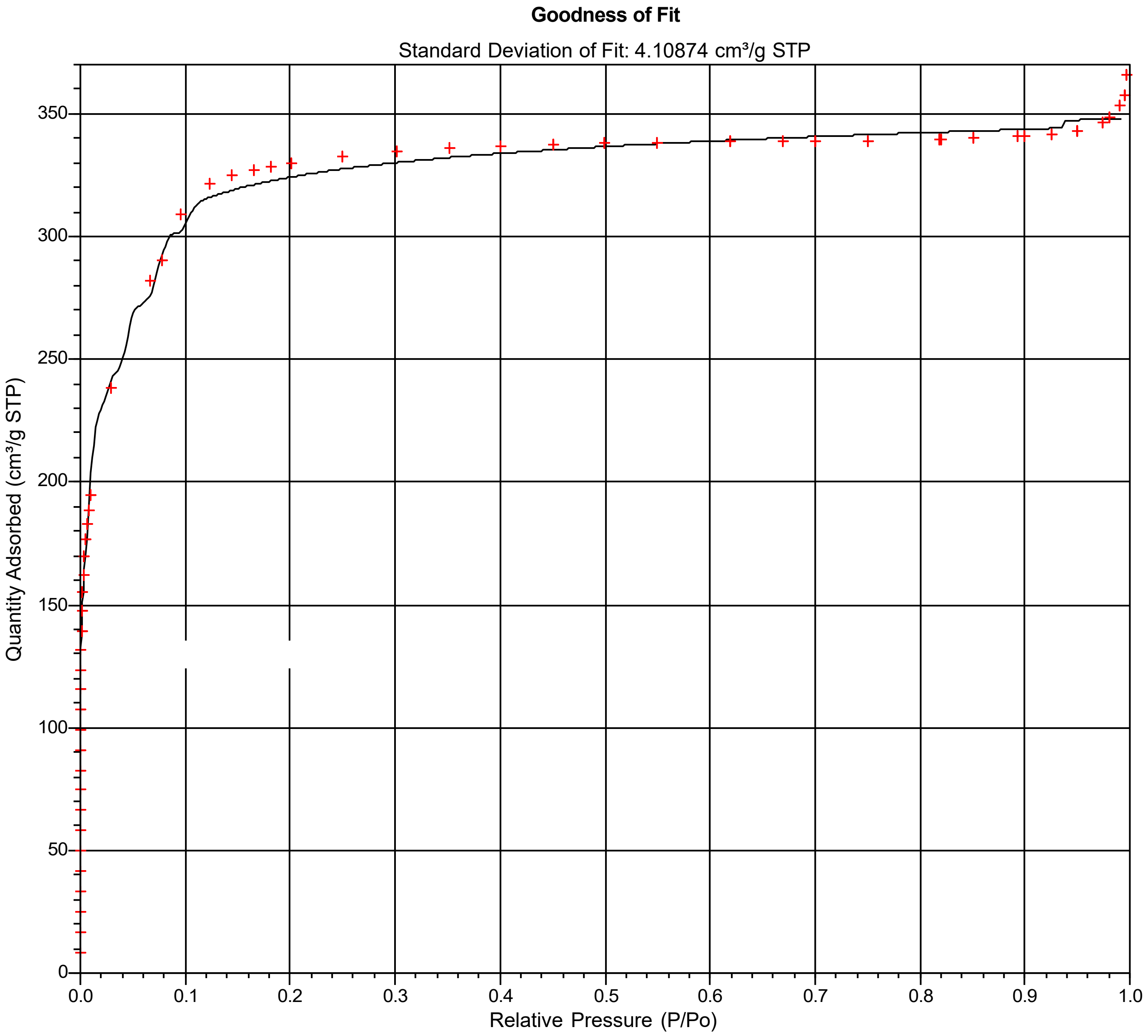

pharmaceutics

MDPI

Sample Information

Method: Default
Sample: 20240428 36h
Operator:
Submitter: Micromeritics
Mass type: Calculated
Empty tube: 35.8507 g
Sample + tube: 35.9327 g
Sample mass: 0.0820 g
Density: 1.000 g/cm³
Type of data: Automatically collected
Instrument type: 2020
Original instrument type: 2020
Comments: Use sample tube with isothermal jacket and filler rod. Follow the instructions on the Silica-Alumina sample data sheet for sample preparation and special instructions. Be sure to use specifications for the current lot.

Sample Tube

Sample tube: Sample Tube
Warm free space: 1.0000 cm³
Cold free space: 1.0000 cm³
Non-ideality factor: 0.0000620
Use isothermal jacket: Yes
Use filler rod: Yes
Vacuum seal type: Seal Frit

Degas Conditions

Degas conditions: Degas Conditions

Evacuation Phase

Temperature ramp rate: 10.0 °C/min
Target temperature: 90 °C
Evacuation rate: 5.0 mmHg/s
Unrest. evacuation from: 5.0 mmHg
Vacuum level: 1.000000e-002 mmHg
Evacuation time: 60 min

pharmaceutics

MDPI

Heading Phase

Ramp rate: 10.0 °C/min
Hold temp: 350 °C
Hold time: 360 min

Evacuation and Heating Phases

Hold pressure: 10 mmHg

Backfill

Backfill sample tube: Yes

Analysis Conditions

Analysis conditions: Silica Alumina, nitrogen @ 77.35 K
Absolute pressure dosing: No

Pressure Table

Relative Pressure (P/Po)	Rel. Pressure Increment (P/Po)
0.010000000	
0.030000000	
0.060000000	
0.080000000	
0.100000000	
0.120000000	
0.140000000	
0.160000000	
0.180000000	
0.200000000	
0.250000000	
0.300000000	
0.350000000	
0.400000000	
0.450000000	
0.500000000	
0.550000000	
0.600000000	
0.650000000	
0.700000000	
0.750000000	

pharmaceutics

Pressure Table

Relative Pressure (P/Po)	Rel. Pressure Increment (P/Po)
0.800000000	
0.820000000	
0.850000000	
0.875000000	
0.900000000	
0.925000000	
0.950000000	
0.975000000	
0.980000000	
0.990000000	
0.995000000	
0.998000000	
0.990000000	
0.980000000	
0.975000000	
0.950000000	
0.925000000	
0.900000000	
0.875000000	
0.850000000	
0.825000000	
0.800000000	
0.750000000	
0.700000000	
0.650000000	
0.600000000	
0.550000000	
0.500000000	
0.450000000	
0.400000000	
0.350000000	
0.300000000	
0.250000000	
0.200000000	
0.140000000	

Preparation

Fast evacuation: No
Unrestricted evacuation from: 5.0 mmHg
Vacuum setpoint: 10 μmHg
Evacuation time: 1.00 h

Leak test: No
Use TranSeal: No

Free Space

Measured before analysis
Lower Dewar for evacuation: No
Evacuation time: 2.00 h
Outgas test: No

Po and Temperature

Po type: Measured at intervals in Psat tube
Measurement interval: 120 min
Temperature type: Calculated from Po or Psat

Dosing

Use first pressure fixed dose: No
Use maximum volume increment: No
Target tolerance: 5.0% or 5.000 mmHg
Low pressure dosing: Yes
Dose amount: 8.0000 cm^3/g STP
Minimum equilibration delay: 0.00 h
Maximum equilibration delay: 2.00 h
Maximum number of decants: 6

Equilibration

	Relative Pressure (P/Po)	Equilibration Interval (s)
1	0.010000000	20
2	0.990000000	10

Minimum equilibration delay at P/Po >= 0.995: 600 s

Sample Backfill

Backfill at start of analysis: Yes
Backfill at end of analysis: Yes

pharmaceutics

MDPI

Sample Backfill

Backfill gas: N2

Adsorptive Properties

Adsorptive: Nitrogen @ 77.35 K (N2)
Non-condensing adsorptive: No
Maximum manifold pressure: 925.00 mmHg
Therm. tran. hard-sphere diameter: 3.8600 Å
Molecular cross-sectional area: 0.162 nm²
Adsorbate molecular weight: 28.01
Ideal gas law with non-ideality correction
Non-ideality factor: 0.0000660
Density conversion factor: 0.0015468
Dosing method: Normal

Psat vs. Temperature Table

	Saturation Pressure (mmHg)	Temperature (°C)
1	600.193	-197.750
2	634.512	-197.300
3	674.383	-196.800
4	720.420	-196.250
5	742.119	-196.000
6	759.833	-195.800
7	777.867	-195.600
8	805.525	-195.300
9	853.268	-194.800
10	903.122	-194.300

pharmaceutics

MDPI

Raw data for SYA@MIL-100(Fe) – 48 h

Summary Report

Surface Area

Single point surface area at P/Po = 0.249552124: 1,048.0071 m²/g

BET Surface Area: 1,274.4686 m²/g

Langmuir Surface Area: 1,527.6766 m²/g

t-Plot Micropore Area: 926.5638 m²/g

t-Plot external surface area: 347.9048 m²/g

BJH Adsorption cumulative surface area of pores between 17.000 Å and 3,000.000 Å width: 111.5455 m²/g

BJH Desorption cumulative surface area of pores between 17.000 Å and 3,000.000 Å width: 110.6912 m²/g

Pore Volume

Single point adsorption total pore volume of pores less than 3,873.040 Å width at P/Po = 0.995000000: 0.546359 cm³/g

Single point desorption total pore volume of pores less than 3,873.040 Å width at P/Po = 0.995000000: 0.555032 cm³/g

t-Plot micropore volume: 0.339412 cm³/g

BJH Adsorption cumulative volume of pores between 17.000 Å and 3,000.000 Å width: 0.103465 cm³/g

BJH Desorption cumulative volume of pores between 17.000 Å and 3,000.000 Å width: 0.111511 cm³/g

Pore Size

Adsorption average pore diameter (4V/A by BET): 17.148 Å

Desorption average pore diameter (4V/A by BET): 17.420 Å

pharmaceutics

MDPI

Pore Size

BJH Adsorption average pore width (4V/A): 37.102 Å

BJH Desorption average pore width (4V/A): 40.296 Å

Horvath-Kawazoe

Maximum pore volume at P/Po = 0.096003756: 0.456021 cm³/g

Median pore width: 14.888 Å

Isotherm Tabular Report

Relative Pressure (P/Po)	Absolute Pressure (mmHg)	Quantity Adsorbed (cm³/g STP)	Elapsed Time (h:min)	Saturation Pressure (mmHg)
			03:43	757.982910
0.000006125	0.004643	8.1511	04:32	
0.000009812	0.007438	16.3024	05:22	
0.000013820	0.010476	24.4542	06:19	
0.000018599	0.014100	32.6060	07:13	
0.000024534	0.018600	40.7569	08:06	
0.000032687	0.024781	48.9049	08:52	
0.000043979	0.033343	57.0510	09:33	
0.000059768	0.045316	65.1937	10:13	
0.000082332	0.062426	73.3283	10:52	
0.000115273	0.087405	81.4516	11:26	
0.000163640	0.124081	89.5597	11:59	
0.000233662	0.177179	97.6458	12:26	
0.000333481	0.252874	105.7068	12:54	
0.000474637	0.359918	113.7315	13:24	
0.000673900	0.511030	121.7027	13:51	
0.000953790	0.723290	129.5934	14:18	
0.001346385	1.021026	137.3707	14:43	
0.001911013	1.449238	145.1300	15:10	
0.002694305	2.043298	152.6786	15:39	
0.003755672	2.848267	159.9041	16:04	
0.005144199	3.901389	166.8934	16:31	
			16:40	758.410522
0.006908186	5.239604	173.3893	16:53	
0.008970420	6.804241	179.9645	17:07	
0.011370064	8.624925	186.1359	17:18	
0.029198544	22.151218	223.6896	17:37	
0.065137126	49.419926	267.4539	17:53	
0.077033000	58.448513	275.8275	18:03	
0.096003756	72.847946	294.8158	18:17	
0.121348690	92.084671	308.9527	18:27	
0.145527494	110.436134	312.7541	18:33	
0.166168141	126.103020	315.1039	18:38	
0.183076102	138.936493	316.6898	18:41	
			18:44	758.912354
0.201265000	152.741898	318.1352	18:47	

pharmaceutics

MDPI

Isotherm Tabular Report

Relative Pressure (P/Po)	Absolute Pressure (mmHg)	Quantity Adsorbed (cm³/g STP)	Elapsed Time (h:min)	Saturation Pressure (mmHg)
0.249960742	189.696564	321.0211	18:51	
0.301135671	228.532303	323.2589	18:55	
0.350069859	265.667145	324.9215	18:59	
0.400215520	303.721375	326.2169	19:02	
0.450011034	341.509583	327.2638	19:05	
0.500237455	379.625000	328.1194	19:07	
0.550158233	417.508270	328.8631	19:09	
0.600304446	455.561829	329.4800	19:12	
0.650115625	493.361481	330.0373	19:14	
0.700107732	531.297546	330.5838	19:17	
0.750267709	569.361450	331.1401	19:19	
0.800134626	607.202759	331.7978	19:21	
0.820259409	622.472534	332.2720	19:24	
0.850402607	645.345703	332.9010	19:26	
0.875097627	664.083435	333.5758	19:29	
0.900336335	683.234497	334.3934	19:31	
0.924996082	701.946106	335.5302	19:33	
0.949991060	720.911072	337.3296	19:36	
0.974549738	739.544800	340.8186	19:39	
0.980597033	744.131897	342.8304	19:41	
0.990557644	751.686646	347.4205	19:45	
0.994618148	754.765015	351.7059	19:48	
0.996704674	756.337524	359.9716	19:59	
0.979519685	743.292053	348.4198	20:04	
0.960974054	729.215210	341.9815	20:08	
0.934128261	708.841064	338.2695	20:11	
0.907645096	688.742249	336.4490	20:14	
0.882293811	669.503357	335.3882	20:16	
0.856992818	650.301880	334.6282	20:19	
0.831839266	631.213257	334.1301	20:21	
0.806733522	612.161011	333.7257	20:23	
0.781604455	593.090393	333.4094	20:26	
0.750280678	569.320068	333.0999	20:28	
0.700353827	531.433716	332.7138	20:30	
0.650465087	493.575836	332.2920	20:33	
0.600279972	455.493958	331.8076	20:35	

pharmaceutics

MDPI

Isotherm Tabular Report

Relative Pressure (P/Po)	Absolute Pressure (mmHg)	Quantity Adsorbed (cm³/g STP)	Elapsed Time (h:min)	Saturation Pressure (mmHg)
0.550153027	417.455872	331.3167	20:38	
0.499831144	379.270660	330.7099	20:40	
0.449918165	341.395477	329.8710	20:43	
0.399652602	303.253448	328.8809	20:45	
			20:47	758.790649
0.350028628	265.598450	327.6191	20:51	
0.299879983	227.546127	326.0552	20:54	
0.250111245	189.782074	323.9923	20:58	
0.200213364	151.920029	321.1198	21:03	
0.140929483	106.935974	315.6479	21:09	

pharmaceutics

MDPI

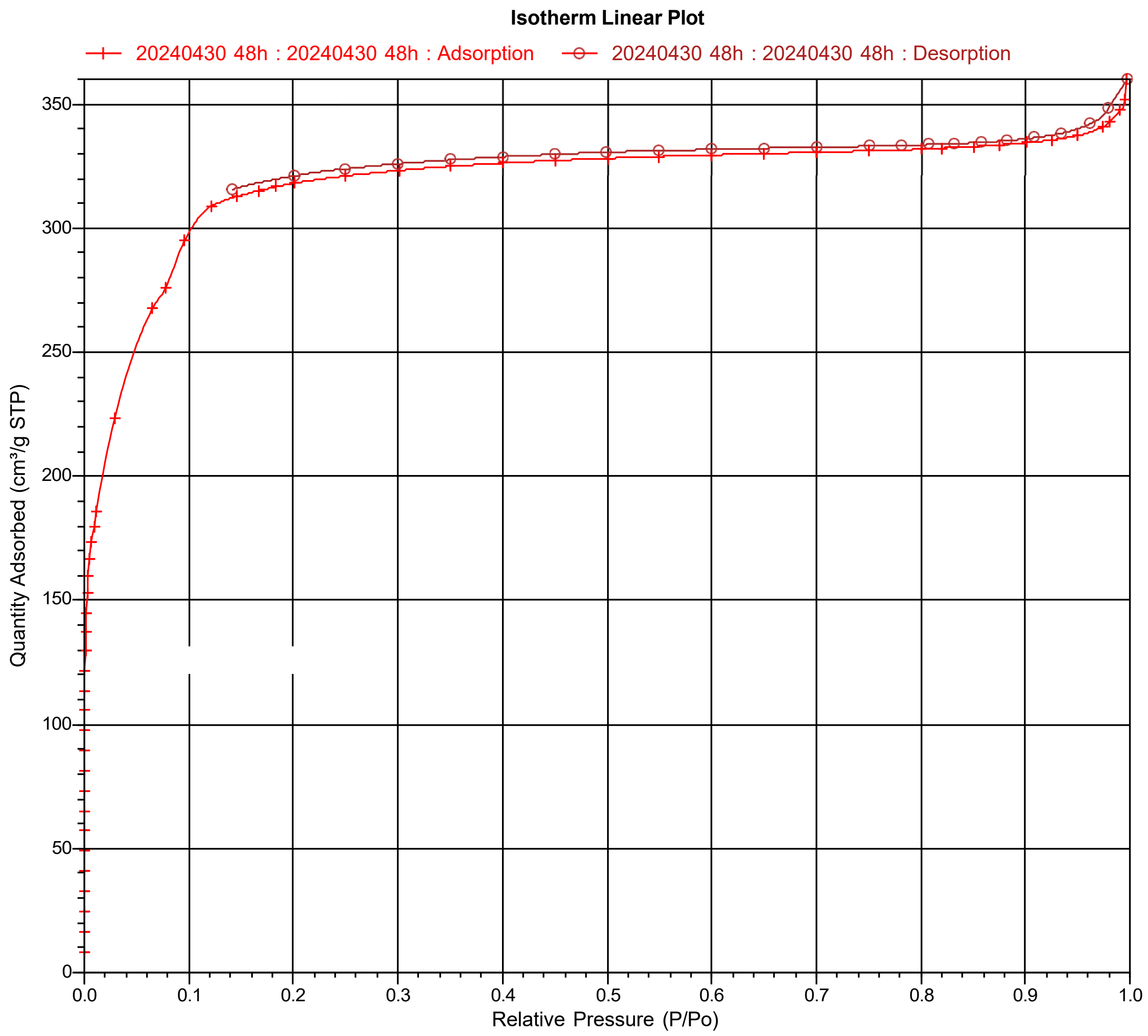

pharmaceutics

MDPI

BET Report

BET surface area: 1274.4686 ± 18.4329 m²/g
Slope: 0.003377 ± 0.000049 g/cm³ STP
Y-intercept: 0.000038 ± 0.000004 g/cm³ STP
C: 88.854690
Qm: 292.8077 cm³/g STP
Correlation coefficient: 0.9996814
Molecular cross-sectional area: 0.1620 nm²

Relative Pressure (P/Po)	Quantity Adsorbed (cm³/g STP)	1/[Q(Po/P - 1)]
0.029198544	223.6896	0.000134
0.065137126	267.4539	0.000261
0.077033000	275.8275	0.000303
0.096003756	294.8158	0.000360
0.121348690	308.9527	0.000447

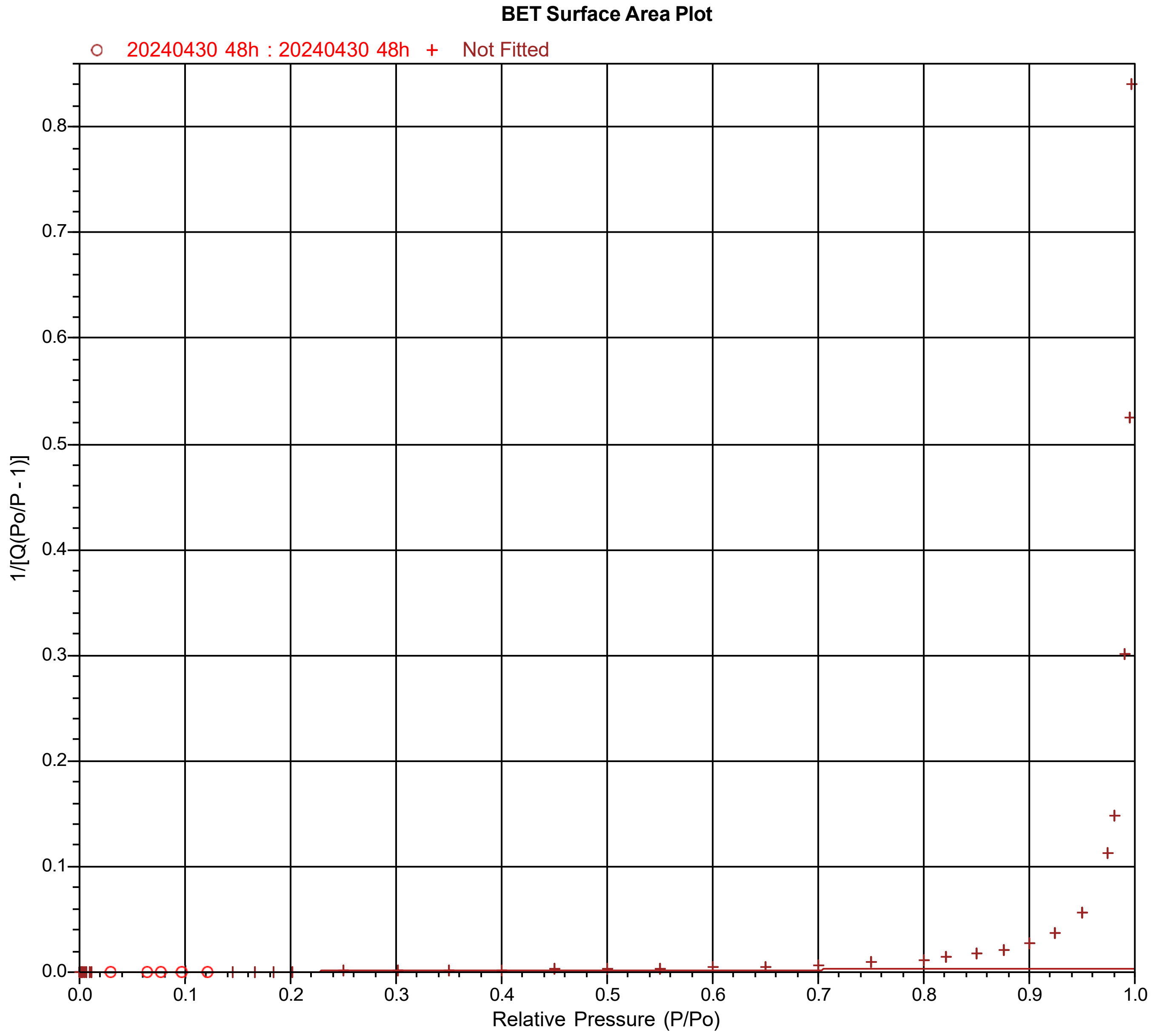
BET Surface Area Plot
20240430 48h : 20240430 48h
Not Fitted
1/[Q(Po/P - 1)]
0.0
0.1
0.2
0.3
0.4
0.5
0.6
0.7
0.8
0.9
1.0
Relative Pressure (P/Po)

pharmaceutics

MDPI

t-Plot Report

Micropore volume: 0.339412 cm³/g
Micropore area: 926.5638 m²/g
External surface area: 347.9048 m²/g
Slope: 22.491904 ± 4.921887 cm³/g·Å STP
Y-intercept: 219.428425 ± 20.427363 cm³/g STP
Correlation coefficient: 0.898233
Surface area correction factor: 1.000
Density conversion factor: 0.0015468
Total surface area (BET): 1274.4686 m²/g
Thickness range: 3.5000 Å to 5.0000 Å
Thickness equation: Harkins and Jura

Thickness Curve

t = [13.99 / (0.034 - log(P/Po))] ^ 0.5

t-Plot Report - Data

Relative Pressure (P/Po)	Statistical Thickness (Å)	Quantity Adsorbed (cm³/g STP)	Fitted
0.000006125	1.6329	8.1511	
0.000009812	1.6657	16.3024	
0.000013820	1.6908	24.4542	
0.000018599	1.7136	32.6060	
0.000024534	1.7356	40.7569	
0.000032687	1.7594	48.9049	
0.000043979	1.7850	57.0510	
0.000059768	1.8127	65.1937	
0.000082332	1.8431	73.3283	
0.000115273	1.8767	81.4516	
0.000163640	1.9137	89.5597	
0.000233662	1.9537	97.6458	
0.000333481	1.9962	105.7068	
0.000474637	2.0412	113.7315	
0.000673900	2.0891	121.7027	
0.000953790	2.1401	129.5934	
0.001346385	2.1946	137.3707	
0.001911013	2.2544	145.1300	
0.002694305	2.3181	152.6786	
0.003755672	2.3851	159.9041	

pharmaceutics

MDPI

t-Plot Report - Data

Relative Pressure (P/Po)	Statistical Thickness (Å)	Quantity Adsorbed (cm^3/g STP)	Fitted
0.005144199	2.4542	166.8934	
0.006908186	2.5248	173.3893	
0.008970420	2.5927	179.9645	
0.011370064	2.6593	186.1359	
0.029198544	2.9864	223.6896	
0.065137126	3.3861	267.4539	
0.077033000	3.4919	275.8275	
0.096003756	3.6472	294.8158	*
0.121348690	3.8376	308.9527	*
0.145527494	4.0076	312.7541	*
0.166168141	4.1471	315.1039	*
0.183076102	4.2587	316.6898	*
0.201265000	4.3770	318.1352	*
0.249960742	4.6896	321.0211	*
0.301135671	5.0196	323.2589	
0.350069859	5.3442	324.9215	
0.400215520	5.6927	326.2169	
0.450011034	6.0614	327.2638	
0.500237455	6.4640	328.1194	
0.550158233	6.9039	328.8631	
0.600304446	7.3978	329.4800	
0.650115625	7.9562	330.0373	
0.700107732	8.6073	330.5838	

pharmaceutics

MDPI

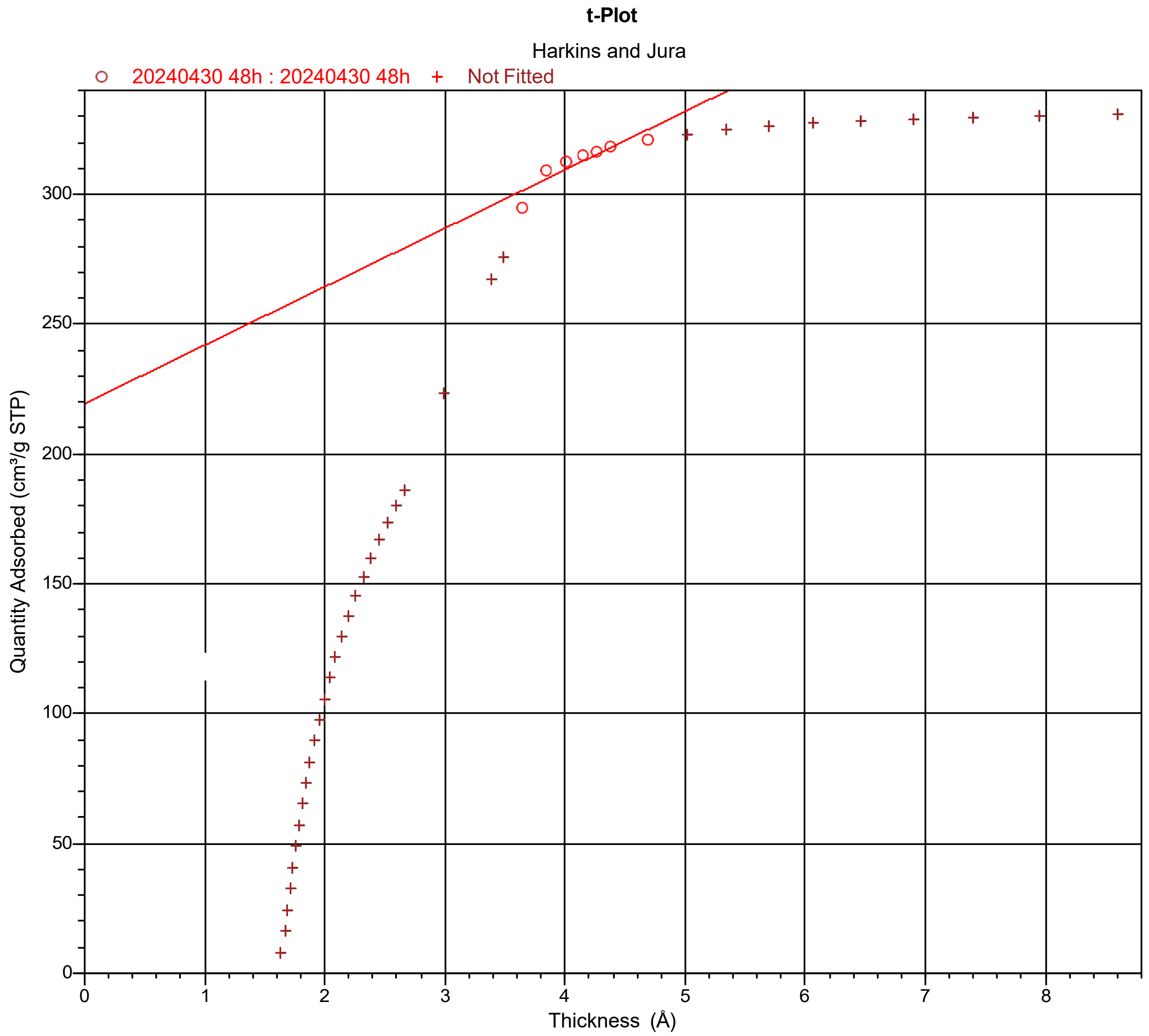

pharmaceutics

MDPI

BJH Adsorption Pore Distribution Report

Faas Correction
Halsey
t = 3.54 [-5 / ln(P/Po)] ^ 0.333

Width range: 17.000 Å to 3,000.000 Å
Adsorbate property factor: 9.53000 Å
Density conversion factor: 0.0015468
Fraction of pores open at both ends: 0.00

Pore Width Range (Å)	Average Width (Å)	Incremental Pore Volume (cm³/g)	Cumulative Pore Volume (cm³/g)	Incremental Pore Area (m²/g)	Cumulative Pore Area (m²/g)
3600.9 - 2066.1	2441.7	0.006873	0.006873	0.113	0.113
2066.1 - 1017.6	1217.2	0.007499	0.014372	0.246	0.359
1017.6 - 780.3	867.2	0.003326	0.017698	0.153	0.512
780.3 - 404.1	480.2	0.005910	0.023608	0.492	1.005
404.1 - 272.8	312.6	0.003087	0.026696	0.395	1.400
272.8 - 207.2	230.6	0.001981	0.028676	0.344	1.743
207.2 - 166.5	182.1	0.001442	0.030118	0.317	2.060
166.5 - 139.8	150.6	0.001226	0.031344	0.326	2.386
139.8 - 116.9	126.1	0.001153	0.032497	0.366	2.752
116.9 - 105.4	110.5	0.000934	0.033431	0.338	3.090
105.4 - 84.7	92.5	0.001213	0.034644	0.524	3.614
84.7 - 70.5	76.1	0.001072	0.035716	0.563	4.177
70.5 - 60.3	64.5	0.001142	0.036858	0.709	4.886
60.3 - 52.5	55.7	0.001258	0.038116	0.903	5.789
52.5 - 46.3	48.9	0.001516	0.039632	1.240	7.029
46.3 - 41.2	43.4	0.002010	0.041642	1.854	8.883
41.2 - 36.9	38.8	0.002477	0.044119	2.556	11.439
36.9 - 33.3	34.9	0.003278	0.047396	3.762	15.201
33.3 - 30.1	31.5	0.004348	0.051744	5.528	20.729
30.1 - 27.3	28.5	0.006030	0.057774	8.467	29.196
27.3 - 24.6	25.7	0.008685	0.066459	13.492	42.688
24.6 - 22.2	23.2	0.012060	0.078519	20.749	63.437
22.2 - 21.4	21.8	0.006424	0.084943	11.799	75.237
21.4 - 20.6	21.0	0.007292	0.092235	13.920	89.157
20.6 - 19.6	20.1	0.011230	0.103465	22.388	111.545

pharmaceutics

MDPI

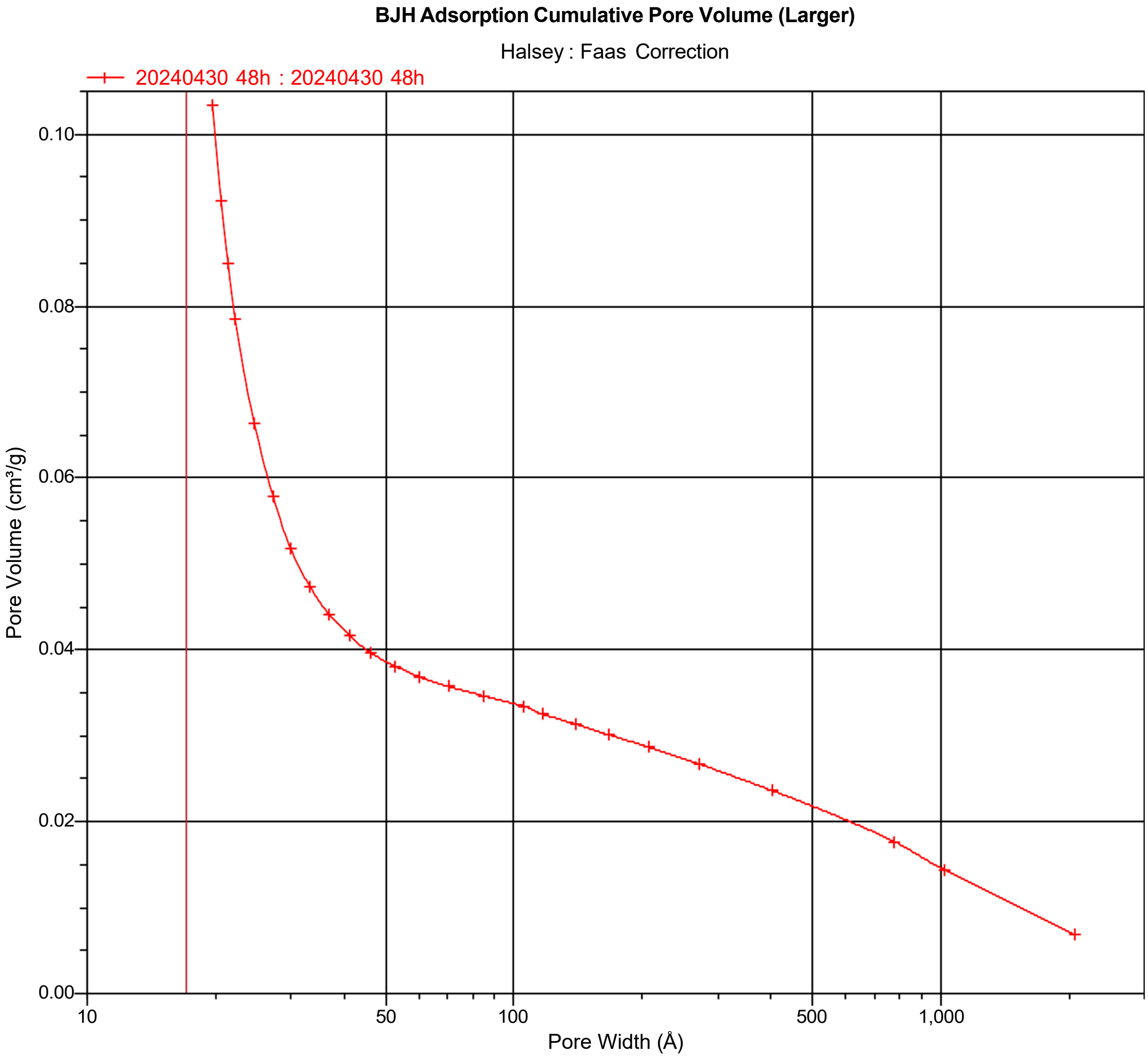

pharmaceutics

MDPI

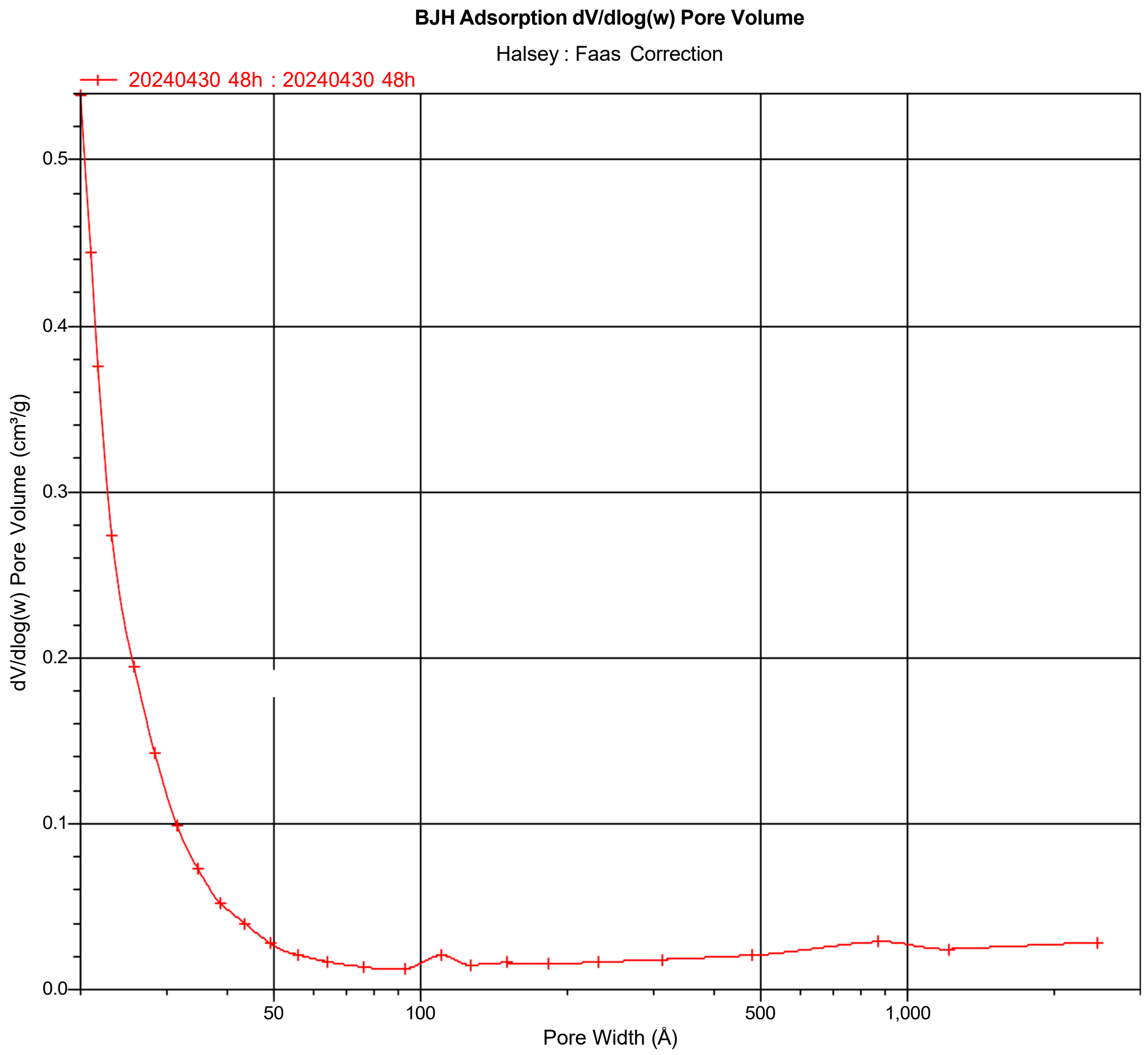

pharmaceutics

MDPI

BJH Desorption Pore Distribution Report

Faas Correction
Halsey
t = 3.54 [-5 / ln(P/Po)] ^ 0.333

Width range: 17.000 Å to 3,000.000 Å
Adsorbate property factor: 9.53000 Å
Density conversion factor: 0.0015468
Fraction of pores open at both ends: 0.00

Pore Width Range (Å)	Average Width (Å)	Incremental Pore Volume (cm³/g)	Cumulative Pore Volume (cm³/g)	Incremental Pore Area (m²/g)	Cumulative Pore Area (m²/g)
5855.5 - 965.1	1087.4	0.019408	0.019408	0.714	0.714
965.1 - 514.2	610.5	0.010889	0.030296	0.713	1.427
514.2 - 309.3	361.7	0.006340	0.036637	0.701	2.129
309.3 - 223.0	251.7	0.003099	0.039736	0.493	2.621
223.0 - 176.4	193.9	0.001793	0.041529	0.370	2.991
176.4 - 146.0	158.1	0.001288	0.042817	0.326	3.317
146.0 - 124.8	133.6	0.000807	0.043624	0.242	3.559
124.8 - 108.9	115.7	0.000659	0.044284	0.228	3.787
108.9 - 96.6	102.0	0.000502	0.044786	0.197	3.984
96.6 - 84.7	89.8	0.000475	0.045261	0.212	4.196
84.7 - 70.6	76.2	0.000584	0.045845	0.307	4.503
70.6 - 60.3	64.5	0.000774	0.046620	0.480	4.982
60.3 - 52.5	55.8	0.001033	0.047653	0.741	5.723
52.5 - 46.3	48.9	0.001127	0.048779	0.922	6.645
46.3 - 41.2	43.3	0.001574	0.050353	1.453	8.098
41.2 - 36.9	38.7	0.002461	0.052815	2.542	10.639
36.9 - 33.2	34.8	0.003100	0.055915	3.561	14.200
33.2 - 30.1	31.4	0.004276	0.060190	5.439	19.639
30.1 - 27.2	28.4	0.005640	0.065830	7.932	27.572
27.2 - 24.6	25.7	0.008022	0.073852	12.472	40.043
24.6 - 22.2	23.2	0.012087	0.085939	20.821	60.864
22.2 - 19.4	20.5	0.025572	0.111511	49.827	110.691

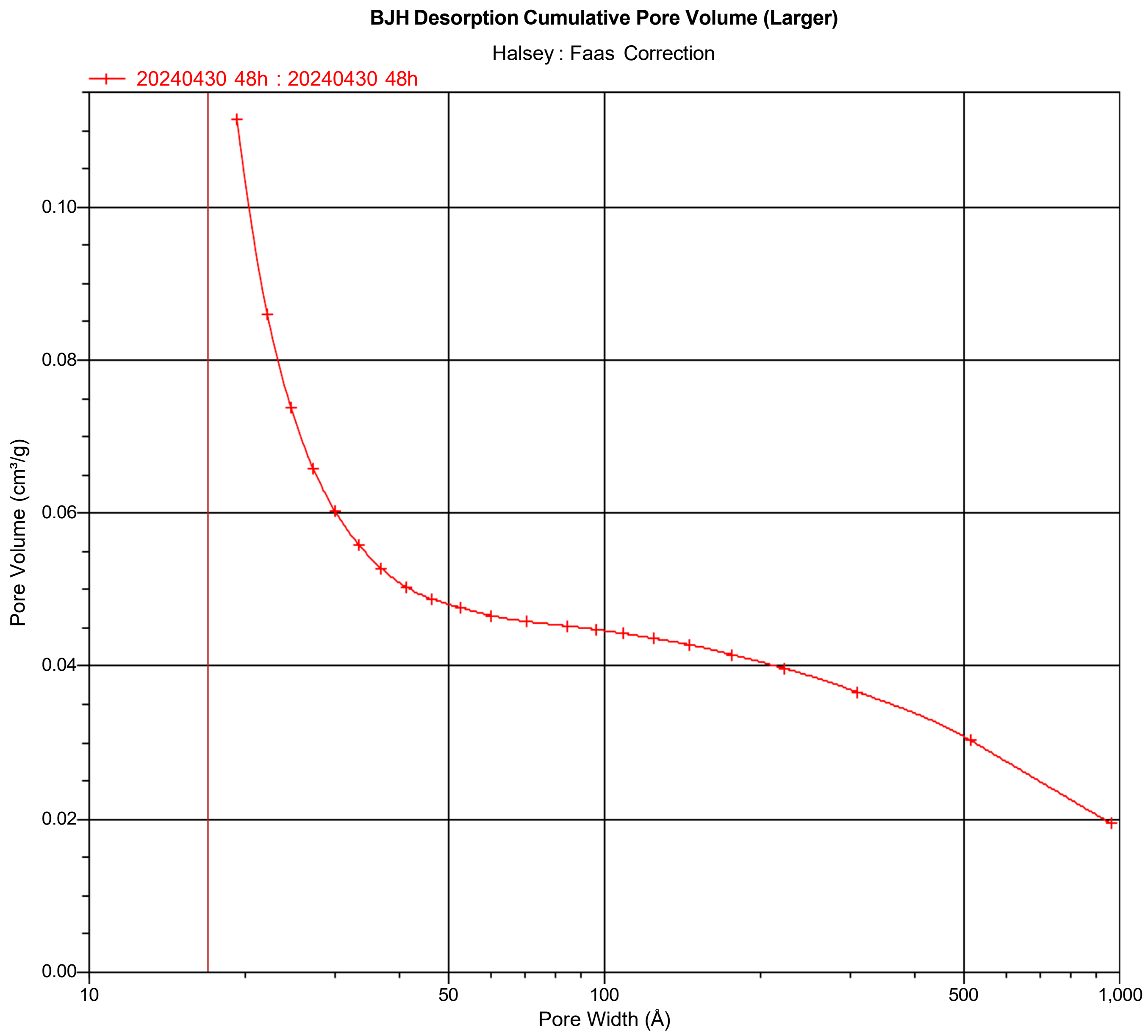
BJH Desorption Cumulative Pore Volume (Larger)
Halsey : Faas Correction
20240430 48h : 20240430 48h
Pore Volume (cm³/g)
0.10
0.08
0.06
0.04
0.02
0.00
10
50
100
500
1,000
Pore Width (Å)

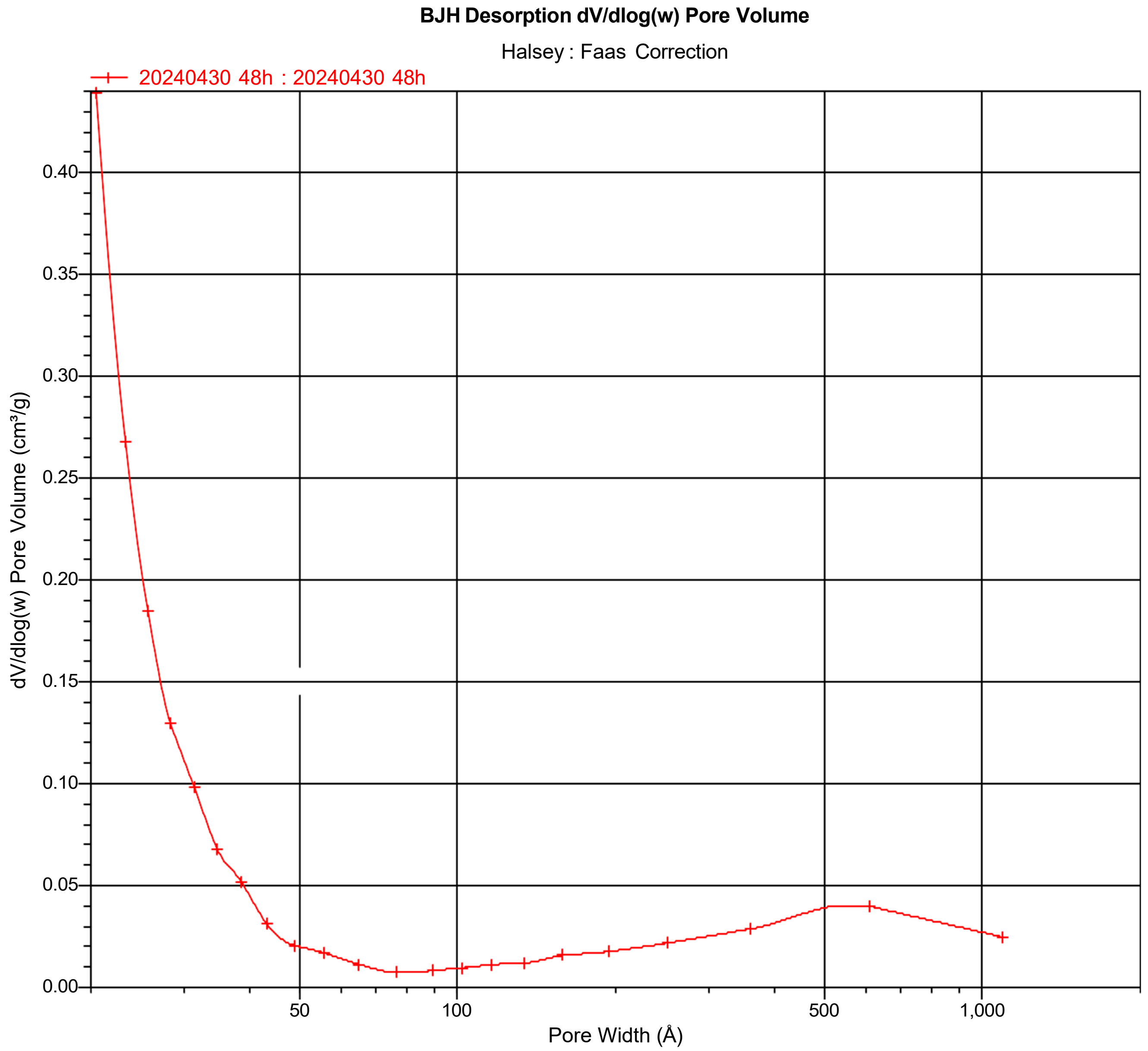
BJH Desorption dV/dlog(w) Pore Volume
Halsey : Faas Correction
20240430 48h : 20240430 48h
dV/dlog(w) Pore Volume (cm³/g)
0.00
0.05
0.10
0.15
0.20
0.25
0.30
0.35
0.40
50
100
500
1,000
Pore Width (Å)

pharmaceutics

MDPI

Porosity Distribution by
Model: N2@77 Cyl Pores in Pillared Clay, NLDFT
Method: Non-negative Regularization: 0.03160
Standard Deviation of Fit: 2.75307 cm³/g STP

Volume in Pores	<	8.79 Å	:	0.00000 cm³/g
Total Volume in Pores	<=	387.34 Å	:	0.47824 cm³/g
Area in Pores	>	387.34 Å	:	5.621 m²/g
Total Area in Pores	>=	8.79 Å	:	1,288.838 m²/g

Pore Size Table

Pore Width (Å)	Cumulative Volume (cm³/g)	Incremental Volume (cm³/g)	Cumulative Area (m²/g)	Incremental Area (m²/g)
8.79	0.00000	0.00000	0.000	0.000
9.15	0.00000	0.00000	0.000	0.000
9.51	0.00249	0.00249	10.493	10.493
9.87	0.01438	0.01189	58.692	48.200
10.22	0.03778	0.02340	150.238	91.545
10.58	0.06930	0.03152	269.392	119.154
10.94	0.10275	0.03345	391.718	122.326
11.30	0.13221	0.02947	496.059	104.341
11.65	0.15426	0.02205	571.746	75.687
12.01	0.16820	0.01393	618.152	46.406
12.37	0.17508	0.00688	640.400	22.248
12.73	0.17707	0.00199	646.670	6.271
13.08	0.17707	0.00000	646.670	0.000
13.44	0.17707	0.00000	646.670	0.000
13.80	0.17707	0.00000	646.670	0.000
14.16	0.17707	0.00000	646.670	0.000
14.51	0.17707	0.00000	646.670	0.000
14.87	0.17746	0.00039	647.710	1.039
15.23	0.18014	0.00268	654.746	7.037
15.59	0.18691	0.00677	672.134	17.387
15.94	0.19886	0.01195	702.113	29.979
16.30	0.21505	0.01619	741.846	39.734
16.66	0.23283	0.01778	784.533	42.687
17.02	0.24965	0.01682	824.078	39.545
17.37	0.26498	0.01533	859.366	35.288
17.73	0.28030	0.01532	893.934	34.568

pharmaceutics

MDPI

Pore Size Table

Pore Width (Å)	Cumulative Volume (cm³/g)	Incremental Volume (cm³/g)	Cumulative Area (m²/g)	Incremental Area (m²/g)
18.09	0.29675	0.01645	930.318	36.384
18.44	0.31452	0.01777	968.849	38.532
18.80	0.33222	0.01770	1006.501	37.652
19.16	0.34791	0.01569	1039.260	32.758
19.52	0.36067	0.01276	1065.407	26.147
19.87	0.37078	0.01011	1085.763	20.357
20.23	0.37951	0.00872	1103.012	17.249
20.59	0.38900	0.00950	1121.459	18.447
20.95	0.40114	0.01213	1144.629	23.170
21.30	0.41690	0.01577	1174.229	29.600
21.66	0.43548	0.01858	1208.532	34.302
22.38	0.45464	0.01916	1242.783	34.252
23.09	0.47013	0.01549	1269.613	26.830
23.81	0.47773	0.00761	1282.394	12.780
24.52	0.47824	0.00050	1283.217	0.823
25.24	0.47824	0.00000	1283.217	0.000
25.95	0.47824	0.00000	1283.217	0.000
26.67	0.47824	0.00000	1283.217	0.000
27.38	0.47824	0.00000	1283.217	0.000
28.10	0.47824	0.00000	1283.217	0.000
28.81	0.47824	0.00000	1283.217	0.000
29.53	0.47824	0.00000	1283.217	0.000
30.24	0.47824	0.00000	1283.217	0.000
30.96	0.47824	0.00000	1283.217	0.000
31.67	0.47824	0.00000	1283.217	0.000
32.39	0.47824	0.00000	1283.217	0.000
33.10	0.47824	0.00000	1283.217	0.000
33.82	0.47824	0.00000	1283.217	0.000
34.53	0.47824	0.00000	1283.217	0.000
35.25	0.47824	0.00000	1283.217	0.000
35.96	0.47824	0.00000	1283.217	0.000
36.68	0.47824	0.00000	1283.217	0.000
37.39	0.47824	0.00000	1283.217	0.000
38.11	0.47824	0.00000	1283.217	0.000
38.82	0.47824	0.00000	1283.217	0.000
39.54	0.47824	0.00000	1283.217	0.000

Pore Size Table

Pore Width (Å)	Cumulative Volume (cm³/g)	Incremental Volume (cm³/g)	Cumulative Area (m²/g)	Incremental Area (m²/g)
40.25	0.47824	0.00000	1283.217	0.000
40.96	0.47824	0.00000	1283.217	0.000
41.68	0.47824	0.00000	1283.217	0.000
42.39	0.47824	0.00000	1283.217	0.000
43.11	0.47824	0.00000	1283.217	0.000
43.82	0.47824	0.00000	1283.217	0.000
44.54	0.47824	0.00000	1283.217	0.000
45.25	0.47824	0.00000	1283.217	0.000
45.97	0.47824	0.00000	1283.217	0.000
46.68	0.47824	0.00000	1283.217	0.000
47.40	0.47824	0.00000	1283.217	0.000
48.11	0.47824	0.00000	1283.217	0.000
48.83	0.47824	0.00000	1283.217	0.000
49.54	0.47824	0.00000	1283.217	0.000
50.26	0.47824	0.00000	1283.217	0.000
52.05	0.47824	0.00000	1283.217	0.000
54.91	0.47824	0.00000	1283.217	0.000
57.77	0.47824	0.00000	1283.217	0.000
60.98	0.47824	0.00000	1283.217	0.000
64.20	0.47824	0.00000	1283.217	0.000
67.42	0.47824	0.00000	1283.217	0.000
70.99	0.47824	0.00000	1283.217	0.000
74.57	0.47824	0.00000	1283.217	0.000
78.50	0.47824	0.00000	1283.217	0.000
82.79	0.47824	0.00000	1283.217	0.000
87.08	0.47824	0.00000	1283.217	0.000
91.37	0.47824	0.00000	1283.217	0.000
96.37	0.47824	0.00000	1283.217	0.000
101.38	0.47824	0.00000	1283.217	0.000
106.38	0.47824	0.00000	1283.217	0.000
112.10	0.47824	0.00000	1283.217	0.000
117.82	0.47824	0.00000	1283.217	0.000
123.90	0.47824	0.00000	1283.217	0.000
130.33	0.47824	0.00000	1283.217	0.000
136.76	0.47824	0.00000	1283.217	0.000
143.91	0.47824	0.00000	1283.217	0.000

pharmaceutics

MDPI

Pore Size Table

Pore Width (Å)	Cumulative Volume (cm³/g)	Incremental Volume (cm³/g)	Cumulative Area (m²/g)	Incremental Area (m²/g)
151.06	0.47824	0.00000	1283.217	0.000
158.93	0.47824	0.00000	1283.217	0.000
167.15	0.47824	0.00000	1283.217	0.000
175.73	0.47824	0.00000	1283.217	0.000
184.66	0.47824	0.00000	1283.217	0.000
193.96	0.47824	0.00000	1283.217	0.000
203.97	0.47824	0.00000	1283.217	0.000
214.33	0.47824	0.00000	1283.217	0.000
225.06	0.47824	0.00000	1283.217	0.000
236.50	0.47824	0.00000	1283.217	0.000
248.29	0.47824	0.00000	1283.217	0.000
261.16	0.47824	0.00000	1283.217	0.000
274.39	0.47824	0.00000	1283.217	0.000
287.97	0.47824	0.00000	1283.217	0.000
302.63	0.47824	0.00000	1283.217	0.000
318.00	0.47824	0.00000	1283.217	0.000
334.08	0.47824	0.00000	1283.217	0.000
350.88	0.47824	0.00000	1283.217	0.000
368.76	0.47824	0.00000	1283.217	0.000
387.34	0.47824	0.00000	1283.217	0.000

pharmaceutics

MDPI

Porosity Distribution by
Model: N2@77 Cyl Pores in Pillared Clay, NLDFT
Method: Non-negative Regularization: 0.03160

Standard Deviation of Fit: 2.75307 cm³/g STP

Isotherm Table

Relative Pressure (P/Po)	Experimental Quantity Adsorbed (cm³/g STP)	Fitted Quantity Adsorbed (cm³/g STP)	Absolute Residual (cm³/g STP)	Relative Residual
0.000006310	8.5743	25.6721	-17.0978	-1.994069
0.000007943	12.2407	27.0896	-14.8490	-1.213084
0.000010000	16.7057	28.7325	-12.0268	-0.719923
0.000012589	22.0487	30.1485	-8.0998	-0.367360
0.000015849	28.1358	33.9321	-5.7963	-0.206011
0.000019953	34.6559	35.9523	-1.2964	-0.037408
0.000025119	41.4336	41.7884	-0.3548	-0.008563
0.000031623	48.0064	45.3673	2.6391	0.054974
0.000039811	54.3592	52.3604	1.9987	0.036769
0.000050119	60.5321	58.6675	1.8647	0.030804
0.000063096	66.5586	64.4447	2.1139	0.031760
0.000079433	72.4673	71.6415	0.8258	0.011396
0.000100000	78.0691	76.5931	1.4760	0.018906
0.000125892	83.4581	83.3236	0.1346	0.001612
0.000158490	88.8575	88.1636	0.6939	0.007809
0.000199526	94.0531	93.6243	0.4288	0.004560
0.000251188	99.2319	98.5445	0.6874	0.006928
0.000316228	104.5304	103.3229	1.2075	0.011552
0.000398107	109.7101	109.7823	-0.0722	-0.000658
0.000501187	114.9289	117.0558	-2.1269	-0.018506
0.000630958	120.2337	122.5730	-2.3393	-0.019456
0.000794328	125.4147	127.8019	-2.3872	-0.019035
0.001000000	130.6307	132.1879	-1.5571	-0.011920
0.001258925	135.8964	136.4452	-0.5488	-0.004039
0.001584895	140.9700	140.3508	0.6192	0.004392
0.001995263	146.0455	144.2252	1.8203	0.012464
0.002511882	151.1752	148.2636	2.9116	0.019260
0.003162276	156.1278	153.0544	3.0735	0.019686
0.003981066	161.1617	158.5478	2.6139	0.016219

pharmaceutics

MDPI

Isotherm Table

Relative Pressure (P/Po)	Experimental Quantity Adsorbed (cm³/g STP)	Fitted Quantity Adsorbed (cm³/g STP)	Absolute Residual (cm³/g STP)	Relative Residual
0.005011868	166.3483	164.4519	1.8963	0.011400
0.006309579	171.3131	170.9547	0.3584	0.002092
0.007943276	176.8115	177.8470	-1.0355	-0.005857
0.010000000	182.7608	184.9536	-2.1928	-0.011998
0.012355640	188.4754	191.8350	-3.3597	-0.017825
0.015186320	195.0933	198.8817	-3.7884	-0.019418
0.018485530	202.5505	205.4710	-2.9205	-0.014419
0.022294740	210.6925	212.2266	-1.5341	-0.007281
0.026653420	219.2041	218.9450	0.2591	0.001182
0.031598160	227.6438	225.3527	2.2911	0.010064
0.037162240	236.1664	234.0284	2.1380	0.009053
0.043374470	244.6802	244.5408	0.1394	0.000570
0.050259210	252.9870	252.0470	0.9400	0.003716
0.057835260	260.8987	261.6457	-0.7470	-0.002863
0.066115920	268.2372	268.8329	-0.5957	-0.002221
0.075109080	274.2912	273.8422	0.4490	0.001637
0.084815920	283.6006	283.0589	0.5417	0.001910
0.095232370	294.1735	295.0335	-0.8599	-0.002923
0.106348200	302.4195	301.4163	1.0032	0.003317
0.118147500	308.1486	305.5250	2.6236	0.008514
0.130609100	310.6249	307.5392	3.0857	0.009934
0.143706600	312.5366	309.2372	3.2994	0.010557
0.157410500	314.1550	310.7860	3.3690	0.010724
0.171685500	315.6425	312.2074	3.4351	0.010883
0.186492100	316.9825	313.5189	3.4636	0.010927
0.201792100	318.1720	314.7340	3.4380	0.010805
0.217539500	319.2135	315.8630	3.3505	0.010496
0.233689500	320.1705	316.9152	3.2553	0.010168
0.250196100	321.0326	317.8987	3.1339	0.009762
0.267011800	321.8293	318.8200	3.0093	0.009351
0.284089500	322.5812	319.6840	2.8972	0.008981
0.301380300	323.2680	320.4964	2.7716	0.008574
0.318838200	323.9069	321.2624	2.6444	0.008164
0.336417100	324.5056	321.9839	2.5216	0.007771
0.354071100	325.0349	322.6659	2.3691	0.007289

pharmaceutics

MDPI

.

Isotherm Table

Relative Pressure (P/Po)	Experimental Quantity Adsorbed (cm³/g STP)	Fitted Quantity Adsorbed (cm³/g STP)	Absolute Residual (cm³/g STP)	Relative Residual
0.371757900	325.5177	323.3104	2.2073	0.006781
0.389435500	325.9647	323.9195	2.0452	0.006274
0.407065800	326.3712	324.4983	1.8729	0.005738
0.424610500	326.7566	325.0462	1.7104	0.005234
0.442034200	327.1133	325.5662	1.5471	0.004730
0.459305300	327.4315	326.0617	1.3698	0.004184
0.476393400	327.7291	326.5318	1.1973	0.003653
0.493271100	328.0085	326.9811	1.0273	0.003132
0.509911800	328.2714	327.4118	0.8596	0.002619
0.526293400	328.5237	327.8211	0.7025	0.002138
0.542394700	328.7577	328.2151	0.5426	0.001651
0.558200000	328.9681	328.5939	0.3742	0.001138
0.573690800	329.1644	328.9546	0.2098	0.000637
0.588853900	329.3479	329.3022	0.0457	0.000139
0.603677600	329.5181	329.6389	-0.1208	-0.000367
0.618153900	329.6814	329.9600	-0.2786	-0.000845
0.632272400	329.8397	330.2668	-0.4271	-0.001295
0.646028900	329.9923	330.5688	-0.5764	-0.001747
0.659417100	330.1391	330.8569	-0.7178	-0.002174
0.672435500	330.2814	331.1319	-0.8505	-0.002575
0.685081600	330.4195	331.3975	-0.9780	-0.002960
0.697355300	330.5537	331.6554	-1.1017	-0.003333
0.709256600	330.6844	331.9016	-1.2172	-0.003681
0.720789500	330.8120	332.1367	-1.3247	-0.004004
0.731953900	330.9361	332.3626	-1.4264	-0.004310
0.742756600	331.0565	332.5819	-1.5254	-0.004608
0.753200000	331.1726	332.7913	-1.6187	-0.004888
0.763289500	331.2857	332.9914	-1.7057	-0.005149
0.773030300	331.4008	333.1825	-1.7817	-0.005376
0.782430300	331.5225	333.3657	-1.8432	-0.005560
0.791496100	331.6545	333.5414	-1.8869	-0.005689
0.800232900	331.7996	333.7108	-1.9112	-0.005760
0.808648700	331.9854	333.8766	-1.8912	-0.005697
0.816752600	332.1904	334.0349	-1.8445	-0.005552
0.824552600	332.3637	334.1861	-1.8224	-0.005483

pharmaceutics

MDPI

Isotherm Table

Relative Pressure (P/Po)	Experimental Quantity Adsorbed (cm³/g STP)	Fitted Quantity Adsorbed (cm³/g STP)	Absolute Residual (cm³/g STP)	Relative Residual
0.832053900	332.5164	334.3388	-1.8224	-0.005481
0.839267100	332.6614	334.4851	-1.8237	-0.005482
0.846200000	332.8069	334.6249	-1.8180	-0.005463
0.852860500	332.9594	334.7653	-1.8058	-0.005424
0.859257900	333.1243	334.9059	-1.7816	-0.005348
0.865398700	333.2952	335.0403	-1.7451	-0.005236
0.871292100	333.4653	335.1687	-1.7034	-0.005108
0.876947400	333.6300	335.3048	-1.6749	-0.005020
0.882369700	333.7953	335.4422	-1.6469	-0.004934
0.887569700	333.9618	335.5735	-1.6117	-0.004826
0.892553900	334.1272	335.6990	-1.5718	-0.004704
0.897328900	334.2897	335.8217	-1.5320	-0.004583
0.901905300	334.4496	335.9748	-1.5252	-0.004560
0.906286800	334.6239	336.1211	-1.4972	-0.004474
0.910484200	334.8105	336.2610	-1.4504	-0.004332
0.914501300	335.0023	336.3946	-1.3923	-0.004156
0.918347400	335.1937	336.5223	-1.3287	-0.003964
0.922026300	335.3800	336.6927	-1.3128	-0.003914
0.925547400	335.5584	336.8730	-1.3147	-0.003918
0.928915800	335.7477	337.0454	-1.2977	-0.003865
0.932136800	335.9531	337.2100	-1.2569	-0.003741
0.935218400	336.1681	337.3674	-1.1993	-0.003568
0.938163200	336.3867	337.5176	-1.1309	-0.003362
0.940978900	336.6049	337.6612	-1.0563	-0.003138
0.943669700	336.8191	337.7983	-0.9793	-0.002907
0.946242100	337.0266	337.9962	-0.9695	-0.002877
0.948700000	337.2255	338.1976	-0.9720	-0.002882
0.951048700	337.4171	338.4012	-0.9840	-0.002916
0.953292100	337.6201	338.5989	-0.9789	-0.002899
0.955435500	337.8363	338.8137	-0.9774	-0.002893
0.957482900	338.0637	339.0501	-0.9865	-0.002918
0.959438200	338.3003	339.3618	-1.0614	-0.003137
0.961305300	338.5445	339.6867	-1.1422	-0.003374
0.963088200	338.7946	339.9969	-1.2023	-0.003549
0.964789500	339.0490	340.2929	-1.2440	-0.003669

pharmaceutics

MDPI

Isotherm Table

Relative Pressure (P/Po)	Experimental Quantity Adsorbed (cm³/g STP)	Fitted Quantity Adsorbed (cm³/g STP)	Absolute Residual (cm³/g STP)	Relative Residual
0.966414500	339.3065	340.5756	-1.2691	-0.003740
0.967965800	339.5659	340.8455	-1.2796	-0.003768
0.969447400	339.8261	341.1032	-1.2771	-0.003758
0.970860500	340.0859	341.3489	-1.2630	-0.003714
0.972209200	340.3446	341.5835	-1.2389	-0.003640
0.973496100	340.6012	341.8072	-1.2060	-0.003541
0.974725000	340.8564	342.0209	-1.1645	-0.003416
0.975897400	341.1594	342.2247	-1.0653	-0.003123
0.977015800	341.5139	342.4191	-0.9052	-0.002650
0.978082900	341.8921	342.6046	-0.7125	-0.002084
0.979101300	342.2722	342.7816	-0.5094	-0.001488
0.980072400	342.6372	342.9504	-0.3132	-0.000914
0.980998700	342.9792	343.1113	-0.1321	-0.000385
0.981882900	343.3247	343.2650	0.0597	0.000174
0.982726300	343.6749	343.4116	0.2634	0.000766
0.983530300	344.0252	343.5512	0.4740	0.001378
0.984297400	344.3721	343.6845	0.6876	0.001997
0.985028900	344.7128	343.8116	0.9012	0.002614
0.985727600	345.0455	343.9330	1.1125	0.003224
0.986392100	345.3672	344.0484	1.3188	0.003819
0.987027600	345.6785	344.1588	1.5197	0.004396
0.987632900	345.9772	344.2639	1.7133	0.004952
0.988209200	346.2628	344.3640	1.8988	0.005484
0.988760500	346.5363	344.4598	2.0765	0.005992
0.989285500	346.7962	344.5510	2.2453	0.006474
0.989785500	347.0429	344.6378	2.4051	0.006930
0.990263200	347.2771	344.7208	2.5563	0.007361
0.990718400	347.4880	344.7998	2.6882	0.007736
0.991151300	347.5853	344.8749	2.7103	0.007798
0.991565800	347.6133	344.9470	2.6663	0.007670
0.991959200	347.6368	345.0152	2.6216	0.007541

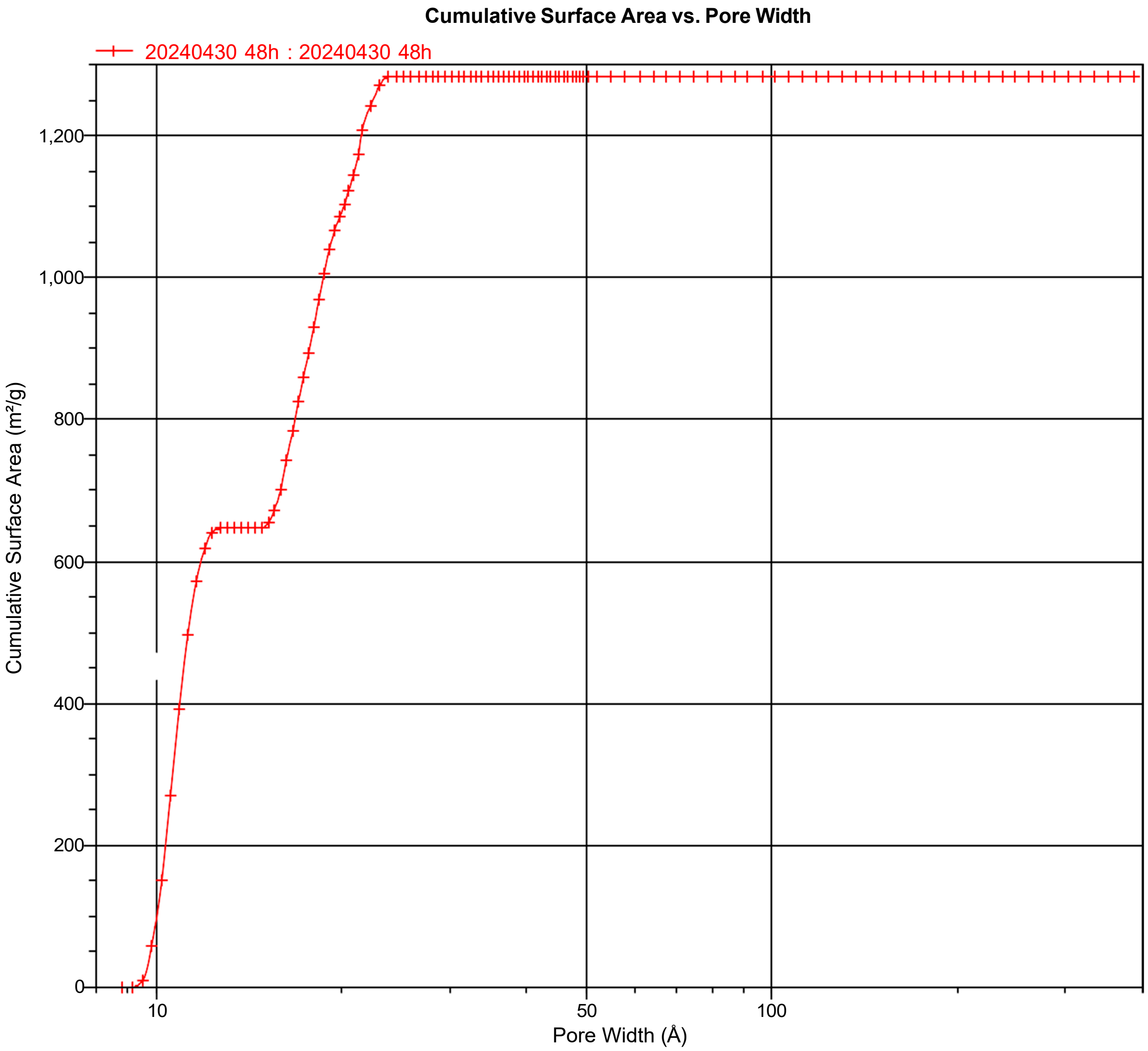
Cumulative Surface Area vs. Pore Width
20240430 48h : 20240430 48h
Cumulative Surface Area (m²/g)
1,200
1,000
800
600
400
200
0
10
50
100
Pore Width (Å)

pharmaceutics

MDPI

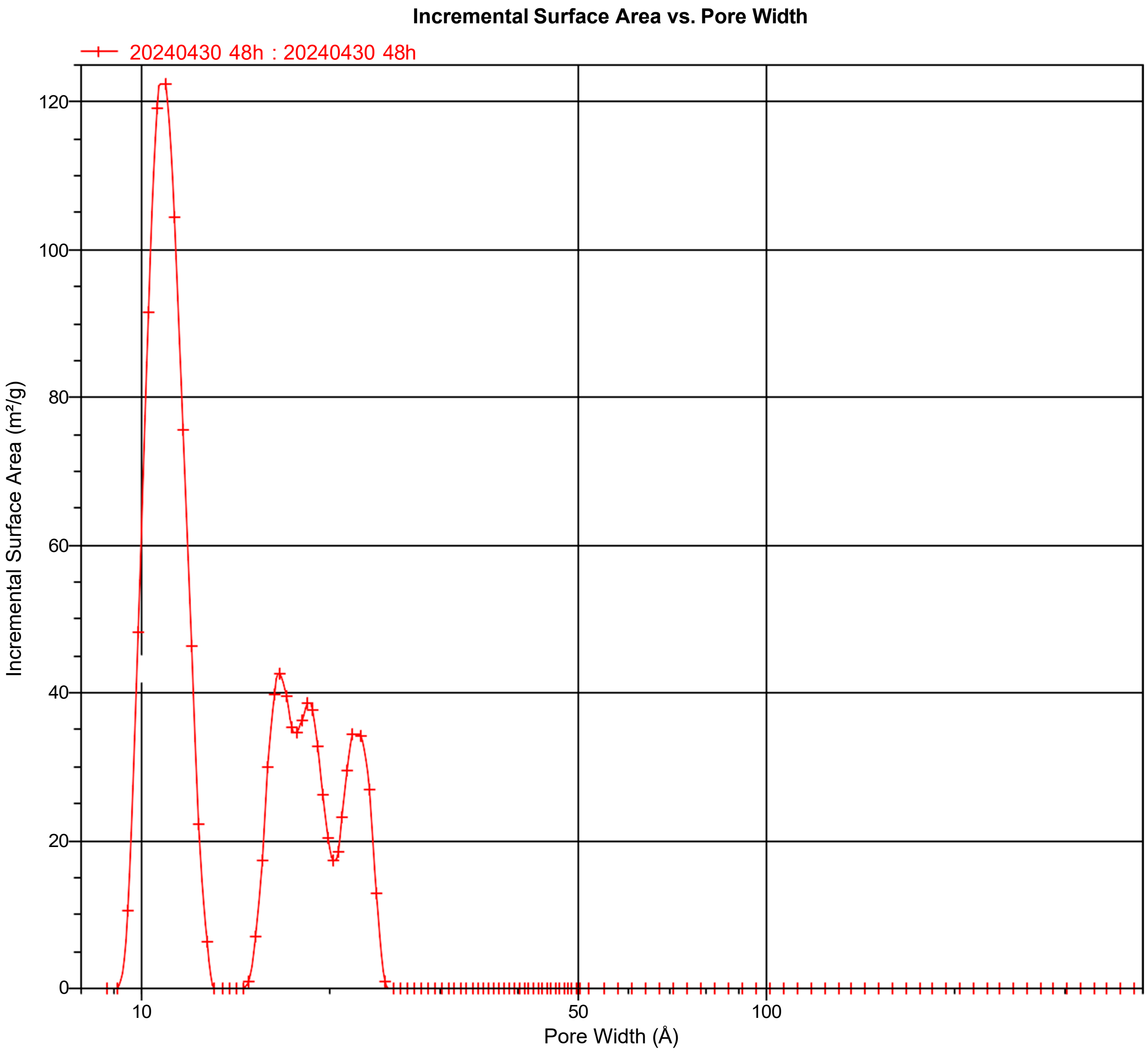

pharmaceutics

MDPI

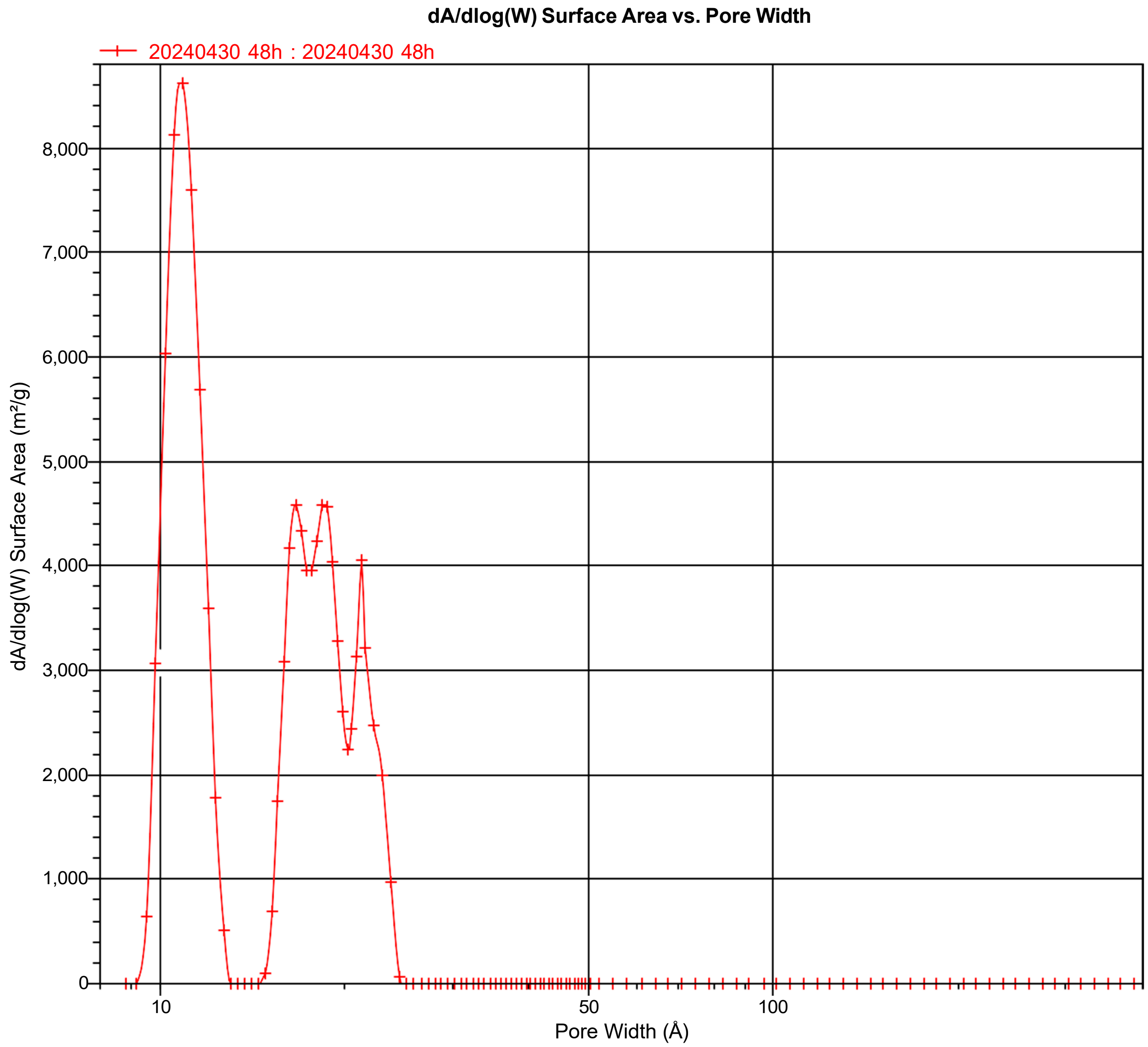

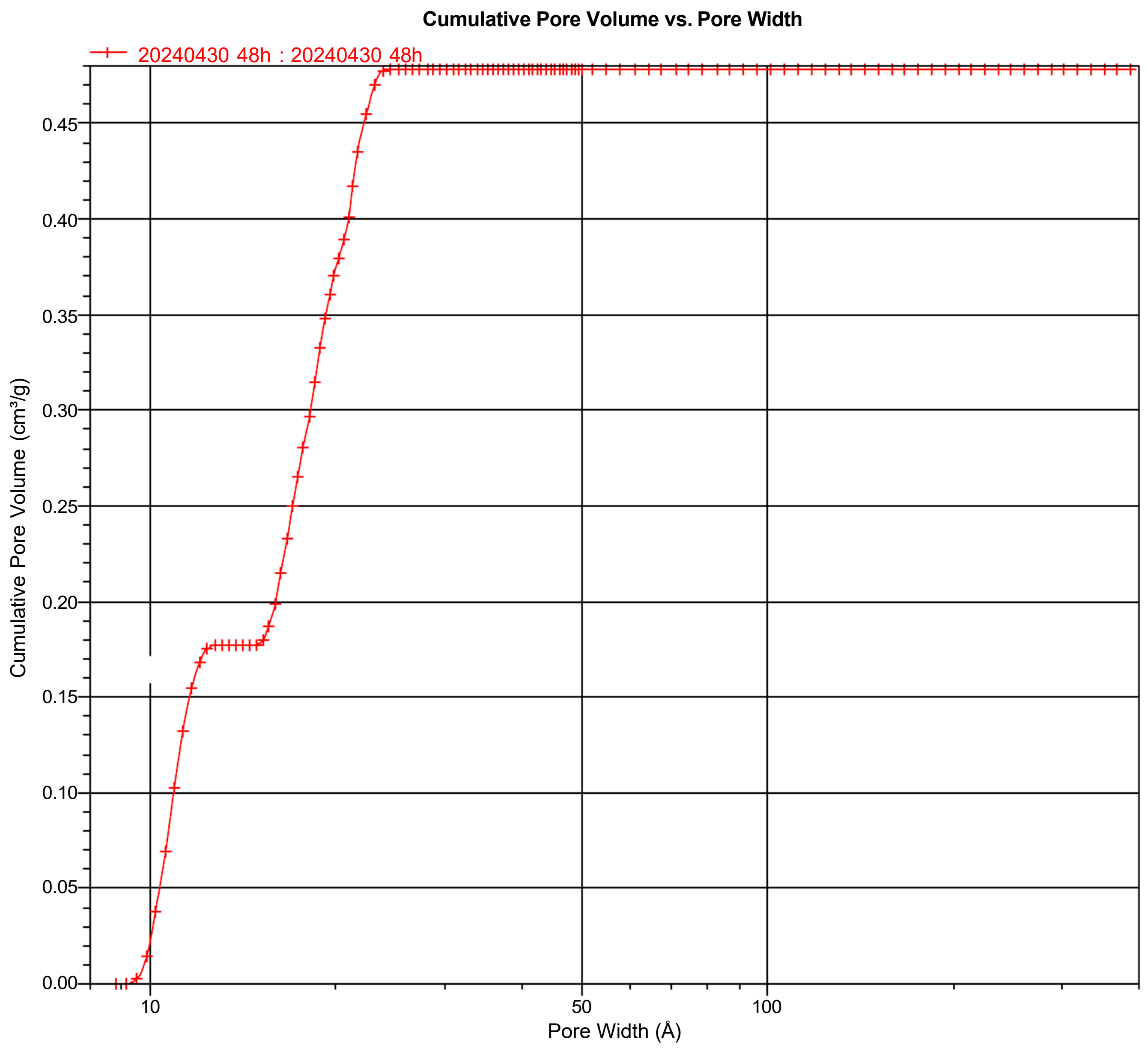
Cumulative Pore Volume vs. Pore Width
20240430 48h : 20240430 48h
Cumulative Pore Volume (cm³/g)
0.00
0.05
0.10
0.15
0.20
0.25
0.30
0.35
0.40
0.45
10
50
100
Pore Width (Å)

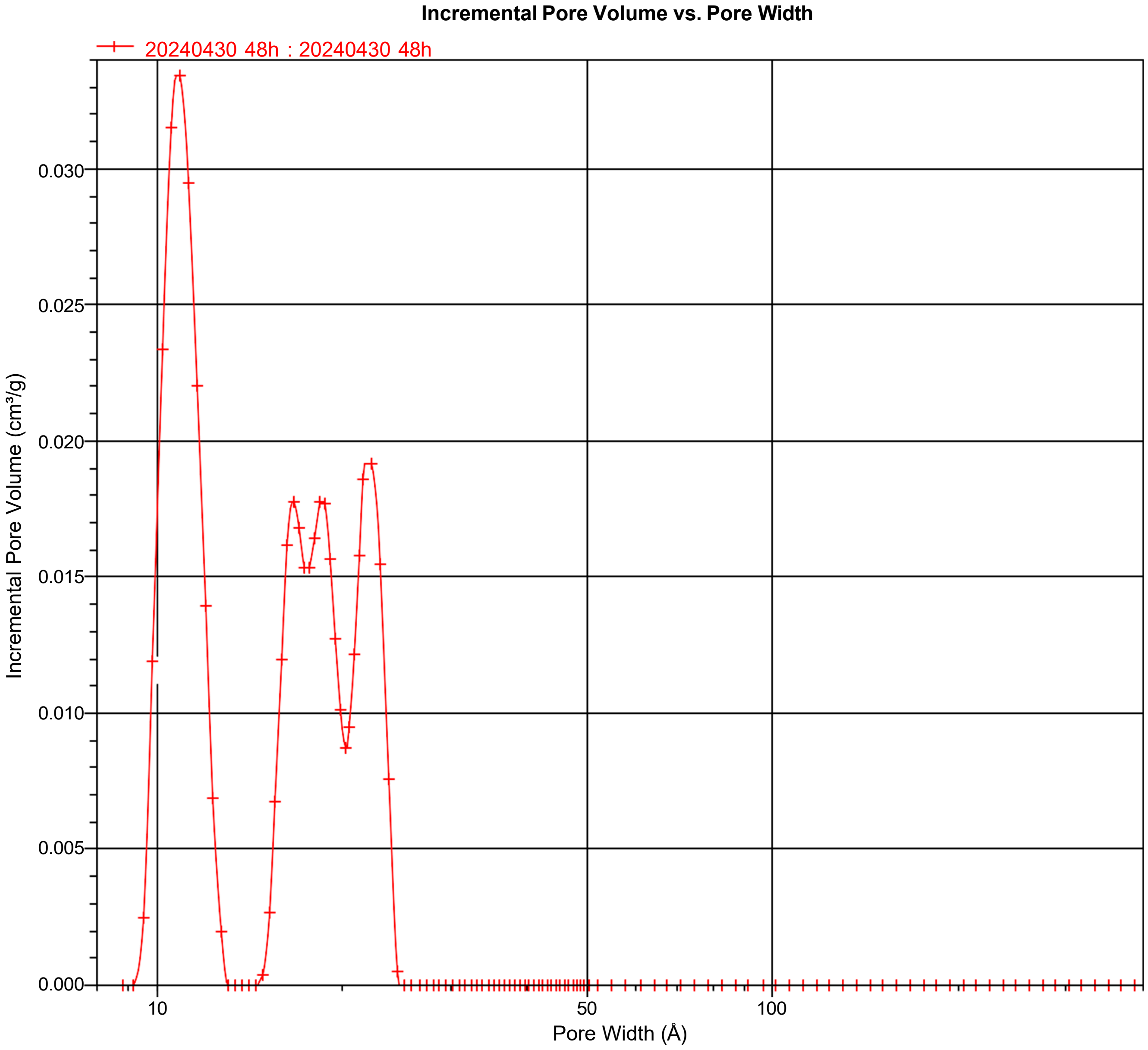
Incremental Pore Volume vs. Pore Width
20240430 48h : 20240430 48h
Incremental Pore Volume (cm³/g)
0.030
0.025
0.020
0.015
0.010
0.005
0.000
10
50
100
Pore Width (Å)

pharmaceutics

MDPI

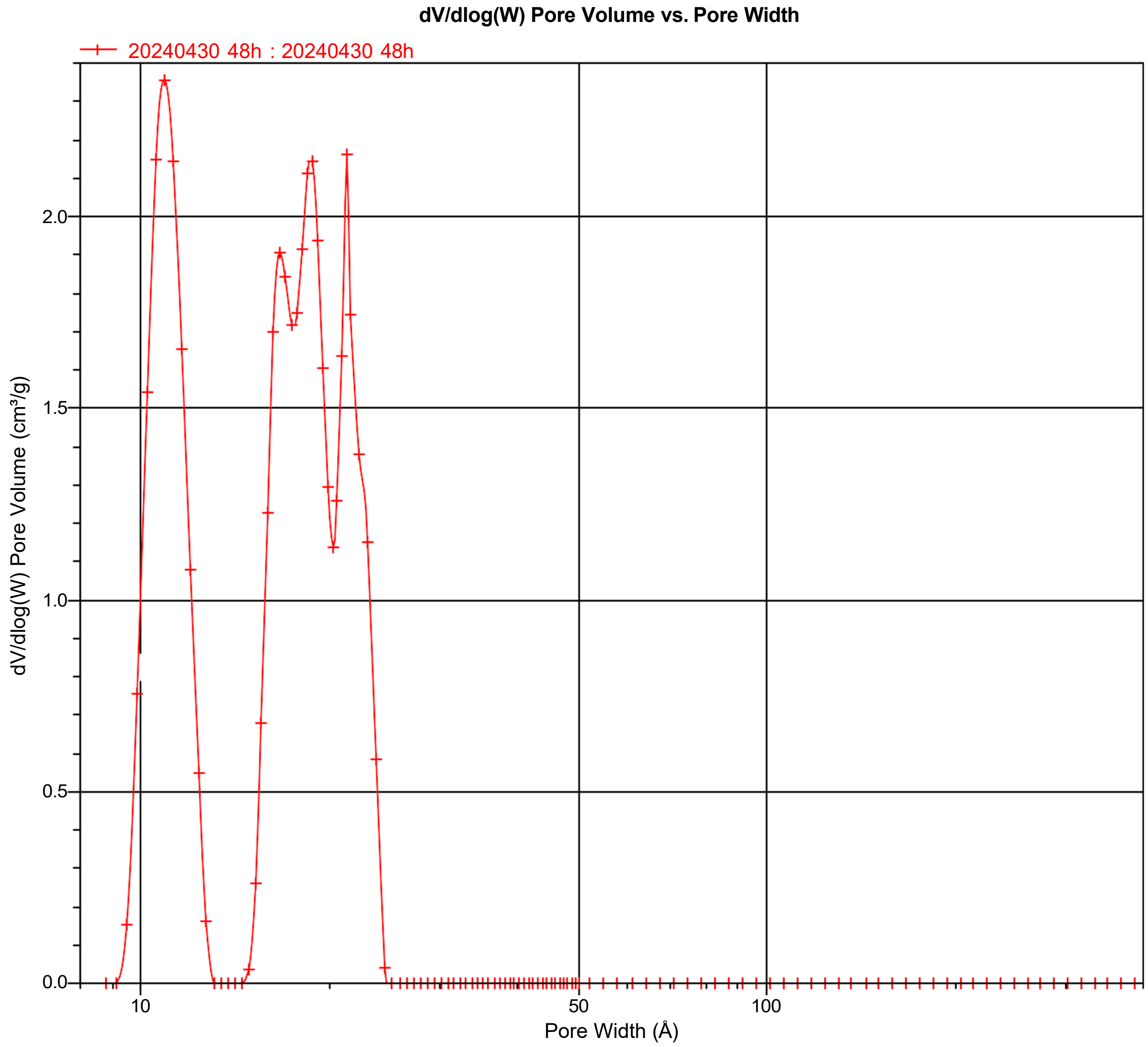

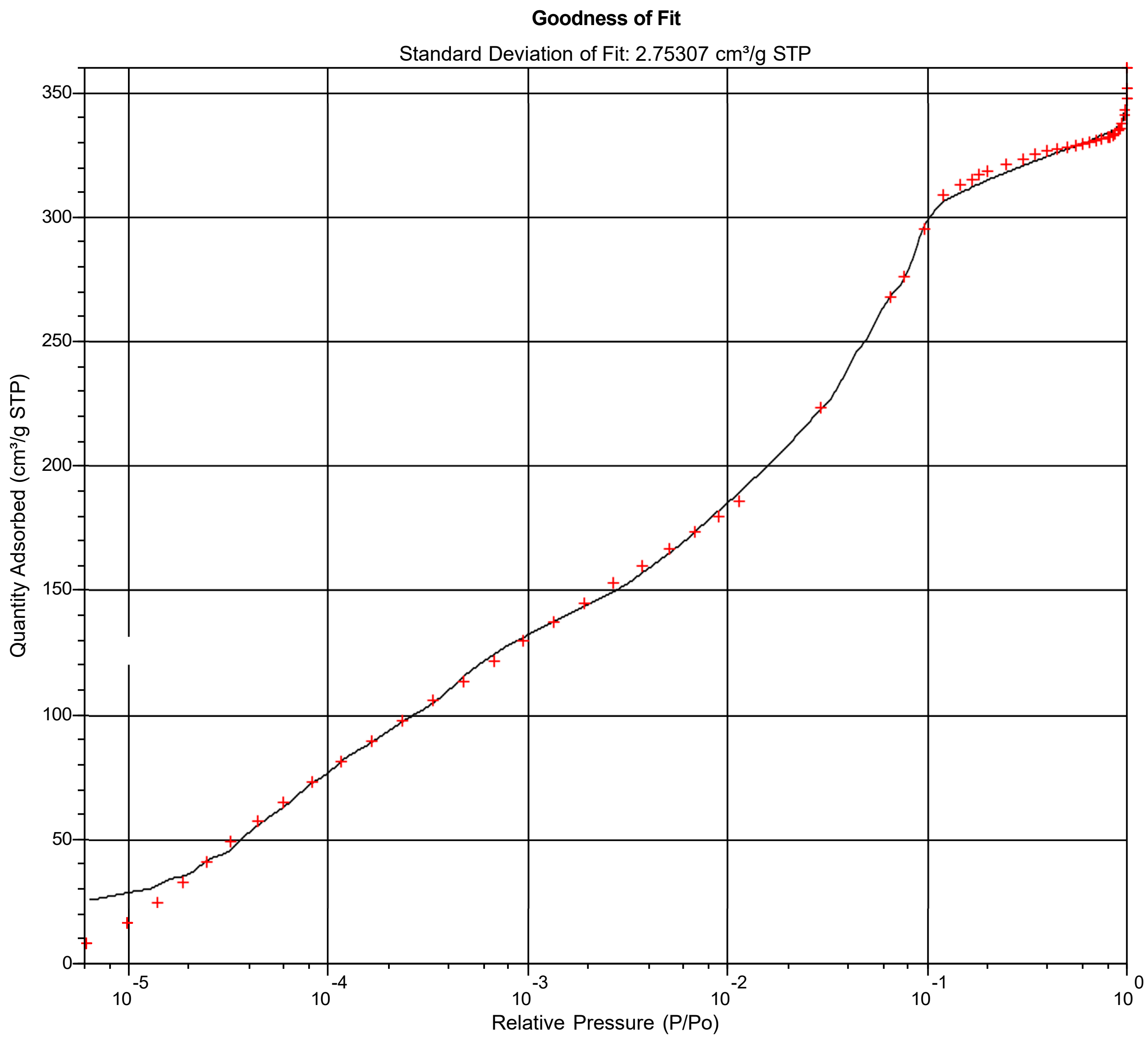
Goodness of Fit
Standard Deviation of Fit: 2.75307 cm³/g STP
Quantity Adsorbed (cm³/g STP)
350
300
250
200
150
100
50
0
10 -5
10 -4
10 -3
10 -2
10 -1
10 0
Relative Pressure (P/Po)

pharmaceutics

MDPI

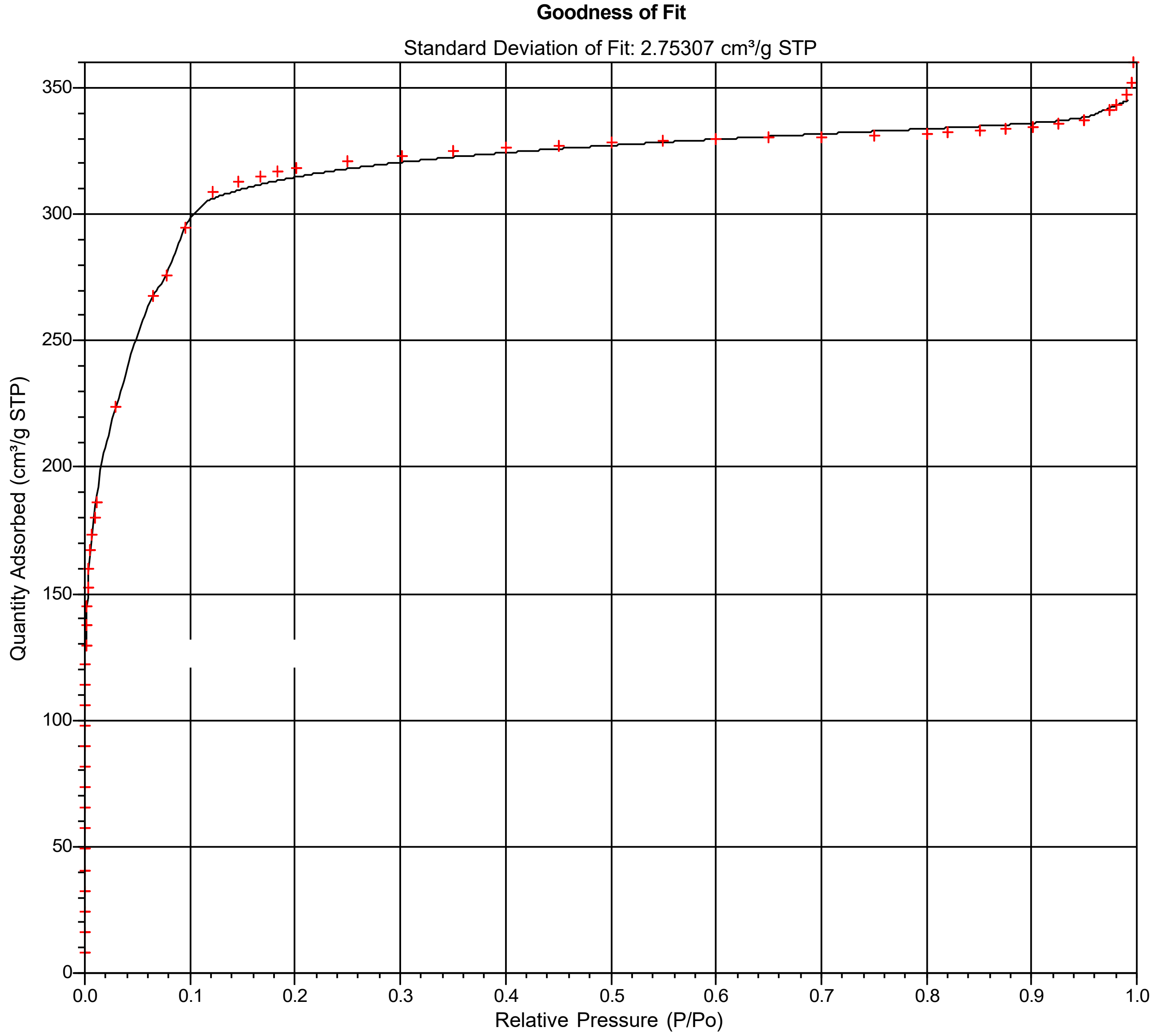

pharmaceutics

MDPI

Sample Information

Method:	Default
Sample:	20240430 48h
Operator:	
Submitter:	Micromeritics
Mass type:	Calculated
Empty tube:	38.3652 g
Sample + tube:	38.4582 g
Sample mass:	0.0930 g
Density:	1.000 g/cm³
Type of data:	Automatically collected
Instrument type:	2020
Original instrument type:	2020
Comments:	Use sample tube with isothermal jacket and filler rod. Follow the instructions on the Silica-Alumina sample data sheet for sample preparation and special instructions. Be sure to use specifications for the current lot.

Sample Tube

Sample tube:	Sample Tube
Warm free space:	1.0000 cm³
Cold free space:	1.0000 cm³
Non-ideality factor:	0.0000620
Use isothermal jacket:	Yes
Use filler rod:	Yes
Vacuum seal type:	Seal Frit

Degas Conditions

Degas conditions: Degas Conditions

Evacuation Phase

Temperature ramp rate:	10.0 °C/min
Target temperature:	90 °C
Evacuation rate:	5.0 mmHg/s
Unrest. evacuation from:	5.0 mmHg
Vacuum level:	1.000000e-002 mmHg
Evacuation time:	60 min

pharmaceutics

MDPI

Heating Phase

Ramp rate: 10.0 °C/min
Hold temp: 350 °C
Hold time: 360 min

Evacuation and Heating Phases

Hold pressure: 10 mmHg

Backfill

Backfill sample tube: Yes

Analysis Conditions

Analysis conditions: Silica Alumina, nitrogen @ 77.35 K
Absolute pressure dosing: No

Pressure Table

Relative Pressure (P/Po)	Rel. Pressure Increment (P/Po)
0.010000000	
0.030000000	
0.060000000	
0.080000000	
0.100000000	
0.120000000	
0.140000000	
0.160000000	
0.180000000	
0.200000000	
0.250000000	
0.300000000	
0.350000000	
0.400000000	
0.450000000	
0.500000000	
0.550000000	
0.600000000	
0.650000000	
0.700000000	
0.750000000	

pharmaceutics

MDPI

Pressure Table

Relative Pressure (P/Po)	Rel. Pressure Increment (P/Po)
0.800000000	
0.820000000	
0.850000000	
0.875000000	
0.900000000	
0.925000000	
0.950000000	
0.975000000	
0.980000000	
0.990000000	
0.995000000	
0.998000000	
0.990000000	
0.980000000	
0.975000000	
0.950000000	
0.925000000	
0.900000000	
0.875000000	
0.850000000	
0.825000000	
0.800000000	
0.750000000	
0.700000000	
0.650000000	
0.600000000	
0.550000000	
0.500000000	
0.450000000	
0.400000000	
0.350000000	
0.300000000	
0.250000000	
0.200000000	
0.140000000	

pharmaceutics

MDPI

Preparation

Fast evacuation: No
Unrestricted evacuation from: 5.0 mmHg
Vacuum setpoint: 10 µmHg
Evacuation time: 1.00 h

Leak test: No
Use TranSeal: No

Free Space

Measured before analysis
Lower Dewar for evacuation: No
Evacuation time: 2.00 h
Outgas test: No

Po and Temperature

Po type: Measured at intervals in Psat tube
Measurement interval: 120 min
Temperature type: Calculated from Po or Psat

Dosing

Use first pressure fixed dose: No
Use maximum volume increment: No
Target tolerance: 5.0% or 5.000 mmHg
Low pressure dosing: Yes
Dose amount: 8.0000 cm³/g STP
Minimum equilibration delay: 0.00 h
Maximum equilibration delay: 2.00 h
Maximum number of decants: 6

Equilibration

	Relative Pressure (P/Po)	Equilibration Interval (s)
1	0.010000000	20
2	0.990000000	10

Minimum equilibration delay at P/Po >= 0.995: 600 s

Sample Backfill

Backfill at start of analysis: Yes
Backfill at end of analysis: Yes

pharmaceutics

MDPI

Sample Backfill

Backfill gas: N2

Adsorptive Properties

Adsorptive: Nitrogen @ 77.35 K (N2)
Non-condensing adsorptive: No
Maximum manifold pressure: 925.00 mmHg
Therm. tran. hard-sphere diameter: 3.8600 Å
Molecular cross-sectional area: 0.162 nm²
Adsorbate molecular weight: 28.01
Ideal gas law with non-ideality correction
Non-ideality factor: 0.0000660
Density conversion factor: 0.0015468
Dosing method: Normal

Psat vs. Temperature Table

	Saturation Pressure (mmHg)	Temperature (°C)
1	600.193	-197.750
2	634.512	-197.300
3	674.383	-196.800
4	720.420	-196.250
5	742.119	-196.000
6	759.833	-195.800
7	777.867	-195.600
8	805.525	-195.300
9	853.268	-194.800
10	903.122	-194.300
